# Quantitative Proteomics Analysis of *Plasmodium vivax* Induced Alterations in Human Serum during the Acute and Convalescent Phases of Infection

**DOI:** 10.1038/s41598-017-04447-5

**Published:** 2017-06-30

**Authors:** Sandipan Ray, Sandip K. Patel, Apoorva Venkatesh, Gangadhar Chatterjee, Naziya N. Ansari, Nithya J. Gogtay, Urmila M. Thatte, Prajakta Gandhe, Santosh G. Varma, Swati Patankar, Sanjeeva Srivastava

**Affiliations:** 1 Department of Biosciences and Bioengineering, Indian Institute of Technology Bombay, Powai, Mumbai 400076 India; 2 Departments of Clinical Pharmacology, Seth GS Medical College & KEM Hospital, Parel, Mumbai 400012 India; 3 Dept of Biochemistry, Grant Govt Medical College and Sir JJ Group of Hospitals, Byculla, Mumbai 400008 India; 4 Department of Clinical Biochemistry, Metabolic Research Laboratories, Wellcome Trust-Medical Research Council Institute of Metabolic Science, University of Cambridge, Addenbrooke’s Hospital, Cambridge, CB2 0QQ United Kingdom

## Abstract

The radial distribution of *Plasmodium vivax* malaria burden has evoked enormous concern among the global research community. In this study, we have investigated the serum proteome alterations in non-severe vivax malaria patients before and during patient recuperation starting from the early febrile to the defervescence and convalescent stages of the infection. We have also performed an extensive quantitative proteomics analysis to compare the serum proteome profiles of vivax malaria patients with low (LPVM) and moderately-high (MPVM) parasitemia with healthy community controls. Interestingly, some of the serum proteins such as Serum amyloid A, Apolipoprotein A1, C-reactive protein, Titin and Haptoglobin, were found to be sequentially altered with respect to increased parasite counts. Analysis of a longitudinal cohort of malaria patients indicated reversible alterations in serum levels of some proteins such as Haptoglobin, Apolipoprotein E, Apolipoprotein A1, Carbonic anhydrase 1, and Hemoglobin subunit alpha upon treatment; however, the levels of a few other proteins did not return to the baseline even during the convalescent phase of the infection. Here we present the first comprehensive serum proteomics analysis of vivax malaria patients with different levels of parasitemia and during the acute and convalescent phases of the infection.

## Introduction

*Plasmodium vivax* is the most widely distributed species among the five parasites responsible for malaria in humans. Even though this plasmodial infection was generally considered as benign, reported case series appeared mostly during the last 15 years from different endemic countries evidently indicated severe manifestations associated with *P*. *vivax* mono-infection^1^. Worrisomely, apart from the incidence of a very high level of parasitemia all the other complications of severe falciparum malaria, including cerebral syndromes and fatal outcomes have been observed in acute *P*. *vivax* infections^2, 3^. Importantly, *P*. *vivax* causes severe and fatal manifestations even at a very low-grade parasitemia^4, 5^ and elicits a greater host response than *P*. *falciparum* ^6^. Insufficient knowledge about the invasion biology of *P*. *vivax* ^7^, and poor understanding of host-parasite interactions are primarily due to the lack of an enduring *in vitro* culture system for this malaria parasite. The increasing global burden of vivax malaria, especially in infants and young children^8–11^ and emerging resistance of this pathogen against commonly used anti-malarials^12^ suggest an urgent need for intensive research in vivax malaria^4, 13–16^.

Early diagnosis and effective treatment against both the blood and liver stages of the parasite is an absolute necessity for vivax malaria control. To this end, microscopic examination of thick and thin blood smears often leads to inaccurate diagnosis, since *P*. *vivax* preferentially invades reticulocytes, resulting in low levels of parasitemia, requiring the need for trained experts with good microscopic skills for proper diagnosis^16^. Polymerase chain reaction (PCR)-based molecular diagnostics are sensitive but not easy to use in point-of-care settings; while rapid diagnostic tests (RDTs) are routinely used for malaria diagnosis due to their ease and simplicity^17, 18^. However, overall sensitivity of RDTs for low-parasite density in *P*. *vivax* samples are much lower than that of *P*. *falciparum* ^19, 20^, actuating the need for development of new diagnostic approaches. In this context, blood biomarkers and surrogate host markers for malaria could be used for early diagnosis, prognosis, monitoring responses to therapy and predicting outcomes.

In recent years, proteome level analyses are found to be informative to comprehend different aspects of malaria pathogenesis^21, 22^. Earlier studies on the serum/plasma proteome of *P*. *falciparum* infected patients have led to the identification of multiple surrogate protein markers of infection and severity^23–27^. Presence of various muscle proteins in plasma samples of children with cerebral falciparum malaria identified through affinity proteomics indicate that plasma levels of carbonic anhydrase III, creatine kinase and myoglobin could serve as the indicators of cerebral malaria in children^26^. Compared to the studies on *P*. *falciparum*, research on *P*. *vivax* is awfully limited, necessitating further investigation in this front. Previously, we have reported serum proteome analysis of vivax malaria patients with identification of several differentially abundant proteins and associated physiological pathways to provide some imperative insights into disease pathogenesis and host immune responses in *P*. *vivax* infection^28^, while proteomic analyses of the parasite directly isolated from the human blood and *Saimiri boliviensis* monkey host have further strengthened our understanding of pathogenesis and host-parasite interactions in vivax malaria^29, 30^. In this present study we investigated serum proteome profiles in low and moderately-high parasitemic vivax malaria patients to evaluate whether there is any possible correlation between serum abundance of diverse classes of proteins and parasite levels in peripheral blood. Furthermore, we aimed to explore the alterations in the host serum proteome profiles during the acute and remission phases (pre- and post-treatment time points) of the infection through analysis of a longitudinal cohort of vivax malaria patients.

## Methods

### Ethics statement

This study was approved by the Institutional Ethics Committees of Seth GS Medical College & King Edward Memorial (KEM) Hospital, Mumbai and Grant Govt. Medical College and Sir JJ Group of Hospitals, Mumbai. After providing detailed explanations about the experimental procedure in the language best understood by the potential participants, written informed consent was obtained from each individual before recruitment. Experiments involving human subjects were carried out in accordance with the relevant guidelines and regulations.

### Subject recruitment and sample collection

Twenty-three low parasitemic (LPVM, parasite count <200/μL of blood) and 40 moderately-high parasitemic (MPVM, parasite count >2000/µL of blood) non-severe vivax malaria patients classified according to the World Health Organization (WHO) guidelines^19^ along with 40 age and gender-matched healthy controls (HC) were enrolled for the study from Seth GS Medical College & King Edward VII Memorial Hospital. The cross-sectional vivax malaria cohorts were treated with Coartem [artemether + lumefantrine (20 mg + 120 mg)]; total course over 3 days, 24 tablets). Patients exhibiting symptoms for severe malaria in accordance with the WHO standard guidelines were excluded from this study. In addition, 7 patients suffering from dengue fever (DF) were also enrolled in this study to serve as a non-malaria febrile infection for a comparative analysis.

In order to perform a longitudinal study, 15 non-severe vivax malaria patients admitted at Grant Govt. Medical College and Sir JJ Group of Hospitals were followed up. Serum samples were collected during early febrile phase immediately after diagnosis (FEB; day 0), defervescence (DEF; day 2) and convalescence (CON; day 15 ± 3) stages. We also recruited age and gender-matched 15 HC participants for a comparative analysis. Diagnosis was confirmed by microscopic examination of thick and thin peripheral blood smear by trained microscopists and RDT. Those patients who were recruited for the longitudinal analysis were treated with intravenous Artesunate (2.4 mg/kg) given after confirmation of diagnosis (time = 0), which was repeated after 12 and 24 h, and then once a day from day 1–3. This was followed by an oral Artemesinin-based Combination Therapy (ACT), which includes Artemether (20 mg) and Lumefantrine (120 mg) for three days. Additionally, 14 daily doses of primaquine (0.50 mg/kg) were provided to the vivax malaria patients as a therapy for radical cure of *P*. *vivax*. Demographic, epidemiological and clinicopathological details, together with past history of diseases of all the malaria patients and controls enrolled for this study were documented. Sample collection and storage was performed as described previously^31, 32^.

### Analysis of clinicopathological parameters

Hematological parameters analyzed in the blood samples collected from LPVM, MPVM, DF patients and HC subjects included hemoglobin level (g/dL), platelet count (thousand/µL), erythrocyte sedimentation rate (mm in 1^st^ hr) and RBC count (millions/µL). Biochemical parameters including liver function tests [alanine aminotransferase (ALT) (IU/L), aspartate aminotransferase (AST) (IU/L), total bilirubin (mg%) and alkaline phosphatase (ALP) (IU/L)] were measured in serum samples. In the longitudinal study, hematological parameters such as hemoglobin (g/dL), platelets (thousand/µL), erythrocyte sedimentation rate (mm in 1^st^ hr), RBC (millions/µL), total WBC (counts/µL), neutrophils (%), monocytes (%), eosinophils (%) and lymphocytes (%), and biochemical parameters such as renal function tests [urea (mg/dL), creatinine (mg/dL), uric acid (mg/dL)] and liver function tests [ALT (IU/L) and AST (IU/L), total protein (g/dL), globulin (g/dL) and albumin (g/dL)] were measured in non-severe vivax patients at three different time points. Hematological investigations were carried out in a fully automated cell counter (Abacus^R^ 5 CT, Diatron, USA), ESR was measured by Westergreen’s method^33^, and biochemical tests were carried out using a fully automated chemical analyzer (Advia1800^R^, Siemens Inc. Germany). Kruskal–Wallis test was carried out to find out any statistically significant difference among the multiple study cohorts; if this multiple comparison test exhibited a significant difference, further statistical analysis was performed using Mann Whitney U test at 5% significance. GraphPad Prism software package (*version* 6.0) was used to generate graphical representations of the datasets.

### Sample processing and gel-based proteomics (2D-DIGE)

The optimal sample size required to present sufficient statistical power at our selected level of significance in two-dimensional difference in-gel electrophoresis (2D-DIGE) analysis was calculated following the protocol as described by Hunt *et al*. 2005^34^. Protein extraction from serum samples for 2D-DIGE was performed as described earlier^32^. In brief, the high abundant proteins were depleted using Albumin & IgG Depletion SpinTrap (GE Healthcare) following the manufacturer’s instructions. Protein extraction from depleted serum samples was performed using trichloroacetic acid (TCA)-acetone precipitation method. Extracted serum proteins (LPVM/MPVM and HC; n = 8) were labeled with fluorescent dyes Cy3 and Cy5, while a mixture of equal amounts from each sample to be analyzed was regarded as an internal standard and was labeled with Cy2 according to the manufacturer’s instructions (GE Healthcare). After labeling, protein samples were pooled, diluted with rehydration buffer and loaded onto 18 cm, 4–7 pH immobilized pH gradient (IPG) strips. Subsequently, isoelectric focusing (IEF) and SDS-PAGE separation were performed following the same protocol as reported earlier^32^.

In order to perform a comparative serum proteomic analysis of a longitudinal cohort of vivax malaria patients at three different time points against healthy controls, three different sets of experiments were carried out. Sera from HC were pooled and labeled with Cy3 and sera obtained from patients during the FEB, DEF and CON stages were each labeled with Cy5, and individually used in different sets of experiments in comparison with HC (in three technical replicates). In every DIGE experiment, dye swapping was carried out while labeling the malaria and control samples to avoid labeling bias.

### Image acquisition and software analysis

A Typhoon 9400 variable mode imager (GE Healthcare) employing suitable excitation/emission wavelengths for CyDyes [(Cy3 (523/580 nm), Cy5 (633/670 nm), Cy2 (488/520 nm)] was used to scan the 2D-DIGE gels, which were further analyzed by DeCyder 2D software, version 7.0 (GE Healthcare). Initially, a comparative analysis of LPVM *vs*. HC and LPVM *vs*. MPVM was performed separately, and subsequently the multiple biological variation analysis (BVA) modules were combined for a cross comparison of the abundances of serum proteins across HC, LPVM and MPVM study populations. In the longitudinal analysis, comparison of the three stages (FEB, DEF and CON) individually against the HC was performed using differential in-gel analysis (DIA) and biological variation analysis (BVA) modules. Differentially abundant protein spots for subsequent mass spectrometric analysis (MS) were selected on the basis of statistical significance (*p* < 0.05) of their differential abundance using Student’s t-test and one-way ANOVA.

### In-gel digestion, MALDI-TOF/TOF analysis and protein identification

Differentially abundant protein spots (*p* < 0.05) were manually excised from GelCode Blue stained preparative gels and subjected to in-gel digestion and matrix-assisted laser desorption/ionization–time-of-flight tandem mass spectrometry (MALDI-TOF/TOF) analysis as described earlier^32^. Protein identification was performed by MS/MS ion search using MASCOT version 2.1 (http://www.martixscience.com) search engine against the Swiss-Prot database with the following parameters: all entries taxonomy, trypsin digestion with one missed cleavage, fixed modifications: carbamidomethylation of cysteine residues, variable modifications: oxidation of methionine residues, mass tolerance 75 ppm for MS and 0.4 Da for MS/MS. Identified proteins having at least two unique matched peptides were selected for further analysis. Only those proteins with a protein identification confidence interval of ≥95% were considered for further analysis.

### In-solution digestion, iTRAQ labeling and OFFGEL fractionation

Further to the gel-based proteomics, gel-free isobaric tag for relative and absolute quantitation (iTRAQ)-based quantitative proteomics analysis was performed on HC, LPVM and MPVM cohorts using the pooled samples (each pool consists of 20 samples). Sample labeling strategy for differential proteomic analysis was; HC-114, LPVM-115 and MPVM-116. Comparative serum proteome analysis of vivax malaria patients at three different time points against healthy controls was also performed using iTRAQ. 10 selected serum samples from each of the study cohorts (FEB, DEF, CON and HC) were split into three pools- Set 1(n = 4); Set 2 (n = 3) and Set 3 (n = 3). Apart from these biological replicates, a pool containing all 10 samples (FEB, DEF, CON and HC) was also analyzed. HC samples were labeled with the 114 iTRAQ reagent, while the three different time-point samples (FEB, DEF and CON) were labeled with 115, 116 and 117 labels, respectively. Buffer exchange (from rehydration solution to TEAB buffer) for all the samples was performed using Amicon Ultra 0.5 mL centrifugal 3 kDa filters (Millipore, Watford, UK) prior to in-solution digestion.

In-solution digestion, iTRAQ labeling and OFFGEL fractionation were performed as previously described^35^. Briefly, 75 μg of protein from each sample was digested using Trypsin (Trypsin Gold, mass spectrometry grade: Promega, Madison, WI, USA) at a 1: 20 trypsin: protein ratio. The resulting peptides were iTRAQ labelled following the manufacturer’s instructions (AB Sciex UK Limited, UK). All the labeled samples were pooled and concentrated using a speed vacuum centrifuge. Pre-fractionation of the labeled peptides was carried out using a 3100 OFFGEL fractionator (Agilent Technologies, Santa Clara, CA) with high-resolution (pH 4–7, 24 cm) IPG strips.

### LC-MS/MS analysis for protein identification and quantitation

Analysis of the iTRAQ-labelled samples was performed using two mass spectrometry (MS) platforms; Agilent 6550 Quadrupole Time-of-Flight (Q-TOF) and Thermo Scientific Q-Exactive. Agilent 6550 iFunnel Q-TOF LC-MS/MS instrument (Agilent Technologies, USA) equipped with a Chip-Cube controlled by the Mass hunter Acquisition software was operated in a positive ion mode for data acquisition. Details of the liquid chromatography (LC) and MS parameters have been described previously elsewhere^35^. The data files obtained were processed by the Spectrum Mill Protein Identification software (Agilent Technologies, USA) using the Paragon algorithm and Mascot *v*2.2 (Matrix Science, London, UK) and were searched against the UniProt database (Proteome ID: UP000005640; Organism ID: 9606; Protein count: 70225) using the following parameters: Data extraction was carried out between MH+ 600 and 4000, IAA for cysteine and iTRAQ (N-term, K) were specified as the fixed modifications and oxidized methionine as a variable modification. The mass spectrometry proteomics data have been deposited to the ProteomeXchange Consortium via the PRIDE^36^ partner repository with the dataset identifier PXD005267.

Normalization and statistical analysis of the quantitative proteomics datasets was carried out using the Perseus workstation (*version* 1.5.5.3)^37^. Reverse and contaminant database hits were removed before executing subsequent statistical analyses. Reporter ion intensity values were log2 transformed, and were normalized by “subtract (mean)” followed by Z score normalization. Proteins groups were selected for valid values, and p-values obtained from a paired t-test were used to estimate significance of differences in the protein abundances between HC and different study cohorts (FEB, DEF and CON stages of malaria). P-values (adjusted) ≤0.05 were considered to be statistically significant.

The iTRAQ labeled samples were also analyzed using a Q-Exactive mass spectrometer (Thermo Fisher Scientific, Waltham, MA, USA) for increasing the proteome coverage. Chromatographic separation and MS parameters were specified following the same method as described earlier^38^. Proteome discoverer 1.4 (Thermo Fisher Scientific) was applied for processing of the raw. msf files; MASCOT 2.2.4 and SEQUEST were used for database searching against the Uniprot *Homo sapiens* FASTA. Database searching parameters included precursor ion mass tolerance of 5 ppm and fragment mass tolerance of 0.02 Da. N-terminal modifications were selected as iTRAQ 4-plex reaction, with dynamic modifications at oxidation (M), deamination (N,Q) and iTRAQ 4-plex (K) in addition to the static modifications at Methylthio (C).

### Enzyme-linked immunosorbent assay (ELISA)

Five selected targets namely, Serum amyloid A (SAA; P0DJI8), Hemopexin (HPX; P02790), Apolipoprotein E (Apo E; P02649), Haptoglobin (HP; P00738), and Apolipoprotein A1 (Apo A1; P02647) were quantified using AssayMax ELISA kits (AssayPro, USA) in serum samples from LPVM, MPVM and DF patients, and healthy controls following the manufacturer’s instructions. Serum abundances of these five proteins along with three other proteins Ceruloplasmin (CP; P00450), Plasma retinol binding protein (RBP4; P02753), and Plasminogen (PLS; P00747) were measured in the FEB, DEF, and CON stages of the infection and HC following the same assay protocol as described earlier^39^.

### Receiver operating characteristic (ROC) analysis

ROC curves [plot of true positives (sensitivity) *vs*. false positives (1- specificity) for each possible cutoff] were used to analyze the efficiency of the differentially abundant serum proteins (only for those above mentioned candidates for which absolute serum concentration values were measured by ELISA) in prediction of low and moderately-high parasitemic cohorts as well as the longitudinal cohorts of vivax malaria patients. ROC curves were plotted using GraphPad Prism software package (*version* 6). Sensitivity and specificity values for these serum proteins were calculated at different threshold points. Two-sided *p*-values less than 0.05 were considered statistically significant. The area under the ROC curve (AUC) was also calculated as a measure of the accuracy of the test.

### Proteins networks and bioinformatics analysis

Differentially abundant serum proteins identified in the comparative quantitative proteomics analysis in the longitudinal cohort and different parasitemic vivax malaria patients were subjected to further bioinformatics analysis using Ingenuity Pathway Analysis (IPA) version 9.0 (Ingenuity® Systems, www.ingenuity.com). Pathway analysis was also performed using DAVID (Database for Annotation, Visualization and Integrated Discovery) database version 6.7 (http://david.abcc.ncifcrf.gov/home.jsp)^40^, and PANTHER (Protein ANalysis THrough Evolutionary Relationships) system, version 7 (http://www.pantherdb.org)^41^.

## Results

### Alterations in clinicopathological parameters in different parasitemic and longitudinal cohort of vivax malaria patients

**Figure 1:**
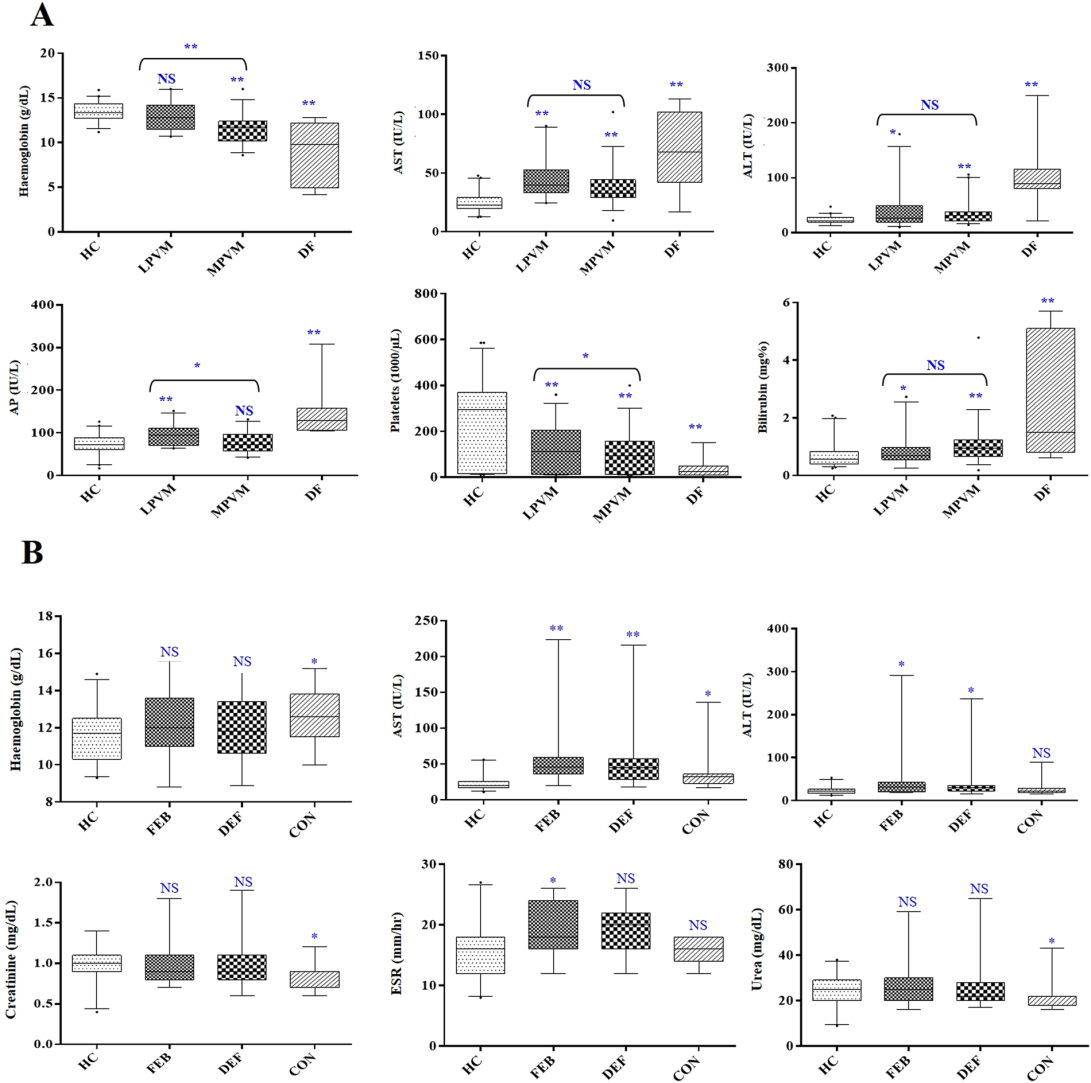
Measurement of clinical laboratory parameters. (**A**) Clinical details of healthy control subjects and low and moderately high parasitemic vivax malaria and dengue fever patients. HC (n = 40), LPVM (n = 23), MPVM (n = 40) and DF (n = 7). (**B**) Clinical laboratory parameters in a longitudinal cohort of vivax malaria patients. HC (n = 15) and a longitudinal cohort (FEB, DEF and CON stages) of vivax malaria patients (n = 15). **Indicates *p* < 0.001, *indicates 0.001 < *p* < 0.05 and NS indicates *p* > 0.05 based on a Mann-Whitney test. Complete lists of demographic and clinical details are provided under supplementary information (Tables S1 and S2).

Parasitemia range for the vivax malaria patients enrolled in this study was 80–9000 (parasite count/µL blood) (Fig. S1). 6–8% of the total number of patients screened were malaria positive (including vivax and falciparum malaria); of which over 75% were infected with *P*. *vivax*, while less than 1% were found to have mixed infections. Comparative analysis of HC and different parasitemic vivax malaria patients indicated that platelet levels, ESR and RBC counts were lower (*p* ≤ 0.05) in the malaria patients (both LPVM and MPVM), while hemoglobin (Hb) levels were found to be significantly lower (*p* < 0.0001) only in MPVM and DF patients (Fig. 1A and Table S1A). Interestingly, ESR, Hb and platelet levels exhibited a significant correlation with parasite counts. ESR was found to be significantly higher, while Hb and platelets were lower in MPVM when compared to LPVM (Table S1A). Similarly, the liver enzymes, ALT and AST were found to be significantly up-regulated in malaria and DF patients compared to HC (*p* < 0.05), but did not exhibit any notable correlation with the parasite counts. Total bilirubin was also found to be significantly higher (*p* < 0.05) in MPVM and DF as compared to HC (Fig. 1A and Table S1A). Since multiple comparison (Kruskal–Wallis) test exhibited a significant difference for most of these parameters (Tables S1B and S2B), further statistical analysis for pair-wise comparison was performed using Mann Whitney U test.

Clinicopathological parameters were also analyzed in the longitudinal cohort of non-severe vivax malaria patients at three time points (Fig. 1B and Table S2A). Among all the hematological parameters measured, WBC count was found to be significantly lower in all three time points (FEB, DEF and CON) of vivax malaria compared to the HC, while ESR was found to be higher only during the early febrile stage, and slowly reduced towards normal with recovery (during the defervescence and convalescent stages). Amongst the various renal and liver function parameters; AST, ALT, total protein, uric acid, albumin and globulin were found to be significantly altered in all the three time points of malaria compared to HC, while creatinine and urea were found to be slightly decreased only in the convalescent stage of the disease (Table S2A).

### Alterations in serum proteome in low and moderately-high parasitemic vivax malaria patients

**Figure 2:**
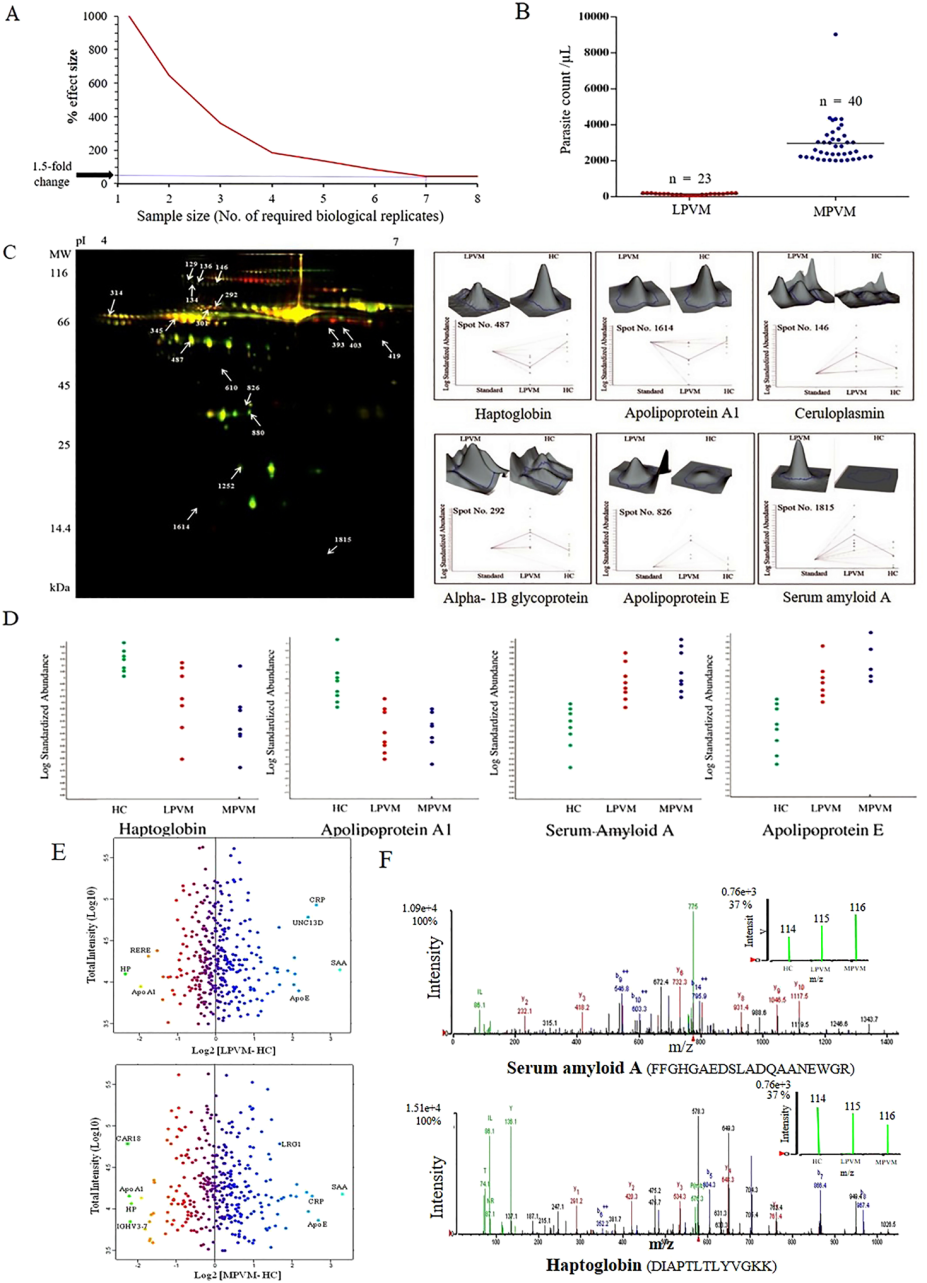
Quantitative proteomic analysis of low and moderately-high parasitemic vivax malaria patients. (**A**) Power calculation for determination of minimum number of required biological variants for 2D-DIGE analysis. Power curve exhibiting the minimum % effect size (fold-change) measurable as a function of sample size with 80% power at *p* < 0.05 level of statistical significance. (**B**) Dot plots representing the parasitemia range for both low and moderately-high parasitemic cohorts of vivax malaria patients (LPVM and MPVM) in terms of parasite counts/µL. (**C**) Representative 2D-DIGE image to compare serum proteome of HC and LP/MPVM patients. Graphical and 3D fluorescence intensity representations of a few selected statistically significant (*p* < 0.05; paired t-test) differentially abundant proteins such as HP, Apo A1, CP, Alpha-1B glycoprotein, Apo E and SAA in LPVM patients. (**D**) Trend of differential abundance for some serum proteins in LPVM and MPVM patients compared to HC identified in 2D-DIGE analysis. Data are represented as standardized log abundance of spot intensity measured in the biological variation analysis (BVA). Serum levels of HP and Apo A1 were found to be consistently lower in vivax malaria patients, while increased abundance for Apo E and SAA was observed in LPVM and MPVM patients compared to HC. (**E**) Graphical representation of the (normalized) protein abundance ratios between the samples (LPVM *vs*. HC and MPVM *vs*. HC), plotted against the total iTRAQ reporter ion intensities for a particular protein. A few selected differentially abundant proteins are labeled. (**F**) Representative MS/MS spectrum for two selected differentially abundant serum proteins identified in different parasitemic vivax malaria patients. Inset presenting the iTRAQ reporter ion intensities for representative peptides in healthy community controls (HC), and LPVM and MPVM patients.

Power calculation was carried out to determine the minimum number of biological replicates required for obtaining statistical significance in our 2D-DIGE analysis. According to our power calculations a minimum of 7 samples from each group was required in DIGE experiment to obtain confidence for 1.5-fold difference at the *p* < 0.05 significance level (Fig. 2A). Consequently, we performed the 2D-DIGE analysis involving 8 subjects from each experimental group (HC, LPVM and MPVM). With an intension to investigate the alterations in human serum proteome in vivax malaria patients, we performed differential proteomics analysis of low and moderately-high parasitemic vivax malaria patients and healthy control subjects (Fig. 2B). Comparative proteomic analysis of LPVM patients and HC by 2D-DIGE indicated differential abundance of 18 protein spots, which were processed further for in-gel digestion (Table S3). In the subsequent MALDI-TOF/TOF mass spectrometric analysis, 11 proteins were identified, among which 10 were up-regulated and 1 was down-regulated (Table S4A). Figure 2C depicts a representative overlapped DIGE gel image of HC and LPVM, and 3D views and graphical representations of a few selected differentially abundant protein spots in LPVM (compared to HC). 19 differentially abundant protein spots were identified in the comparative analysis of LPVM and MPVM, among which 8 were identified by mass spectrometric analysis (Tables S3 and S4B). Interestingly, some of the proteins such as SAA, HP, Apo E and Apo A1 exhibited sequential alterations in their serum abundances with the increase in parasitemia (Fig. 2D and Table 1).

**Table 1:** Differentially abundant serum proteins identified in the low and moderately-high parasitemic vivax malaria patients^#^.

Sl No.	Protein	Uniprot Accession ID	Unique Peptides (iTRAQ /DIGE)	Fold change HC *vs*. LPVM (iTRAQ/DIGE)	Fold change HC *vs*. MPVM (iTRAQ)	Fold change MPVM *vs*. LPVM (iTRAQ/DIGE)	Associated Pathways^^^
1	Apolipoprotein E*	P02649	12/21	1.01/3.18	1.13	1.12	Chylomicron-mediated lipid transport, HDL-mediated lipid transport, Scavenging by Class A Receptors, Retinoid metabolism and transport
2	Alpha-2-macroglobulin^$^	P01023	63/39	0.79	0.78	0.98/0.38	HDL-mediated lipid transport. Platelet degranulation. Intrinsic pathway of Fibrin Clot Formation. Degradation of the extracellular matrix. Rho GTPase cycle
3	Apolipoprotein A-II	P02652	7	0.72	0.48	0.66	HDL-mediated lipid transport, Chylomicron-mediated lipid transport, Scavenging by Class A Receptors, Retinoid metabolism and transport
4	Apolipoprotein A1*^$^	P02647	37/24	0.63	0.46	0.73/0.28–0.42	ABC transporters in lipid homeostasis, Platelet degranulation, Chylomicron-mediated lipid transport, HDL-mediated lipid transport, PPARA activates gene expression, Scavenging of heme from plasma, Scavenging by Class B Receptors, Scavenging by Class A Receptors, Retinoid metabolism and transport, Amyloids
5	Serum albumin^†$^	P02768	26/30	0.47	0.35	0.73/0.33–0.40	HDL-mediated lipid transport, Platelet degranulation, Recycling of bile acids and salts, Scavenging of heme from plasma and Transport of organic anions
6	Titin	Q8WZ42	2	1.69	3.77	2.23	Striated muscle contraction, Platelet degranulation
7	Gelsolin	P06396	18	0.76	0.78	1.02	Caspase-mediated cleavage of cytoskeletal proteins. Amyloid fiber formation
8	C-reactive protein	P02741	5	1.61	8.57	5.31	Classical antibody-mediated complement activation
9	Complement component C9^$^	P02748	5/11	1.11/3.5	1.43	1.29	Terminal pathway of complement. Regulation of Complement cascade
10	Vitronectin	P04004	6	1.05	1.12	1.07	Molecules associated with elastic fibres, Integrin cell surface interactions, Syndecan interactions, ECM proteoglycans, Regulation of Complement cascade
11	Hemoglobin subunit beta	P68871	10	1.17	1.76	1.5	Erythrocytes take up carbon dioxide and release oxygen; Erythrocytes take up oxygen and release carbon dioxide, Scavenging of heme from plasma, Factors involved in megakaryocyte development and platelet production
12	Hemopexin*	P02790	18/17	1.05	1.14	1.09/1.56	Scavenging of heme from plasma
13	Hemoglobin subunit alpha	P69905	7	1.05	1.52	1.45	Erythrocytes take up carbon dioxide and release oxygen. Erythrocytes take up oxygen and release carbon dioxide. Scavenging of heme from plasma
14	Glutathione peroxidase 3	P22352	2	1.03	0.69	0.67	Detoxification of reactive oxygen species
15	Haptoglobin*^$^	P00738	22/11	1.01/0.54	0.53	0.53/0.25–0.35	Scavenging of heme from plasma
16	Alpha-1-antitrypsin^$^	P01009	37/20	1.14/2.29	1.5	1.31	Platelet degranulation
17	Clusterin^$^	P10909	11/8	0.85	0.98	1.16/0.56–0.62	Platelet degranulation
18	Serum amyloid A-1 protein*	P0DJI8	7	1.47	1.38	0.94	RIP-mediated NFkB activation via ZBP1, Scavenging by Class B Receptors, DEx/H-box helicases activate type I IFN and inflammatory cytokines production, G alpha (q) signalling events, G alpha (i) signalling events, Formyl peptide receptors bind formyl peptides and many other ligands,TAK1 activates NFkB by phosphorylation and activation of IKKs complex, Advanced glycosylation endproduct receptor signaling, TRAF6 mediated NF-kB activation, Amyloids
19	Ig mu chain C region^$^	P01871	15/8	1.33/2.48	1.34	1.01	CD22 mediated BCR regulation. Antigen activates B Cell Receptor (BCR) leading to generation of second messengers
20	Ceruloplasmin^$^	P00450	28/18	1.17/2.33	1.37	1.17	Metal ion SLC transporters, Iron uptake and transport
21	Leucine-rich alpha-2-glycoprotein	P02750	10	1.46	1.7	1.16	—
22	Alpha-1-antichymotrypsin^$^	P01011	24/18	1.39/3.48	1.79	1.29	—
23	Inter-alpha-trypsin inhibitor heavy chain H4^$^	Q14624	24/21	1.11/2.95	1.21	1.08	—
24	Alpha-1B-glycoprotein^$^	P04217	10/16	1.00/1.89–5.1	1.15	1.15	—
25	Vacuolar protein sorting-associated protein 33B	Q9H267	2	0.92	1.93	2.11	—
26	Myelin basic protein	P02686	2	0.8	2.06	2.58	—

^#^This is a partial list for a few selected candidates identified in iTRAQ and 2D-DIGE -based quantitative proteomics analysis; complete lists of the identified differentially abundant proteins are provided under supplementary information (Tables S4 and S5). ^^^Associated pathways obtained from Uniprot database. ^$^Differential abundances for these candidates are also identified in 2D-DIGE (details are provided in Table S4).^*^Differential serum abundances of these proteins are validated by ELISA (details are provided in Table S11A). ^†^Differential abundance of serum albumin indicates the measurement of the residual HSA remained after immunodepletion.

In order to enhance the coverage of serum proteome and increase the possibilities for detection of the low abundant proteins in serum, which may not be identified by the gel-based methods; further differential proteomics analysis was performed using iTRAQ-based quantitative approach (Fig. 2E and Table S5A). In the iTRAQ-based quantitative proteomics analysis, we identified increase in the serum levels of 24 proteins in LPVM, and 30 proteins in MPVM; whereas serum levels of 26 and 28 proteins was found to be reduced in LPVM and MPVM, respectively, compared to HC (Table S5B). 24 differentially abundant candidates were found to be common between LPVM and MPVM. Figure 2F represents the MS/MS spectra for some selected proteins with the insets depicting the iTRAQ reporter ion intensities for representative peptides in HC, LPVM and MPVM. As identified in 2D-DIGE; iTRAQ analysis also revealed a gradual alteration in serum abundance of multiple proteins including SAA, HP, Apo E and Apo A1. Moreover, a few sequentially altered candidates such as Titin (Q8WZ42), C-reactive protein (CRP) (P02741), Hemoglobin subunit alpha (P69905) & beta (P68871), which were not detected in DIGE, were identified in iTRAQ analysis (Table 1). We also identified some proteins such as Glutathione peroxidase 3 (P22352), Hemoglobin subunit beta, Myelin basic protein (P02686) and HPX, which exhibited detectable differential abundances only in MPVM, while in LPVM patients their serum levels were found to be nearly comparable with HC.

### Changes in human serum proteome profile during the acute and convalescent phases of vivax malaria

**Figure 3:**
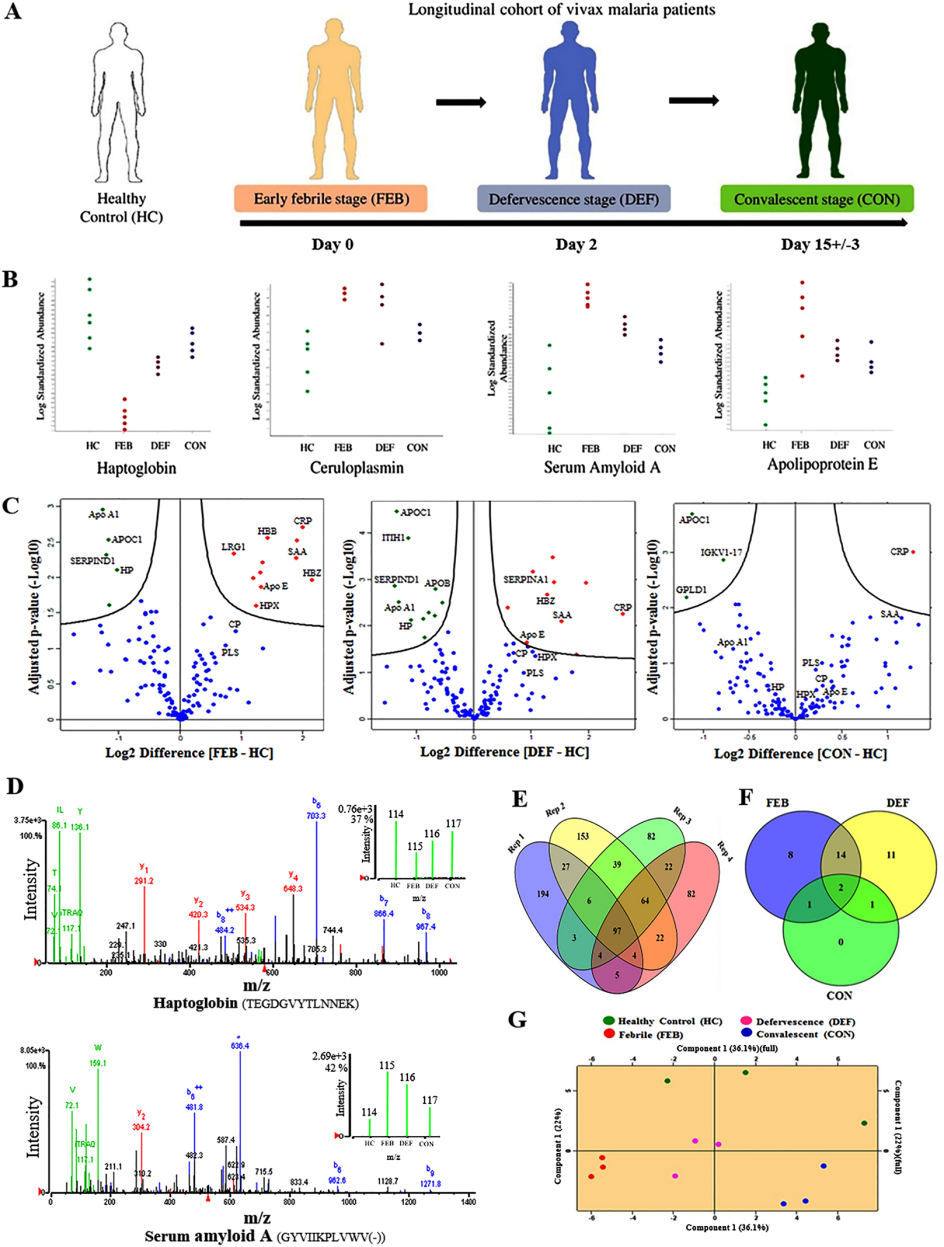
Quantitative proteomic analysis of a longitudinal cohort of vivax malaria patients. (**A**) Schematic representation of a longitudinal cohort of vivax malaria patients analyzed in this study. Blood samples were collected during the early febrile (FEB; D0), defervescence (DEF; D2) and convalescent (CON; D15 ± 3) stages (Drawn by S.R.). (**B**) Trend of a few selected differentially abundant serum proteins such as HP, CP, SAA and Apo E in FEB, DEF and CON stages of vivax malaria identified in 2D-DIGE analysis. Data are represented as standardized log abundance of spot intensity. (**C**) Volcano plots showing *p*-values (−log10) versus difference of group means of FEB-HC, DEF-HC and CON-HC (log2). Red, up-regulated; Green, down-regulated; and Blue, remained unaltered (adjusted *p*-value > 0.05) proteins. A few selected differentially abundant proteins are labeled. (**D**) Representative MS/MS spectrum for two selected differentially abundant serum proteins (HP; down-regulated and SAA; up-regulated) identified in the longitudinal cohort of vivax malaria patients. Inset presenting the iTRAQ reporter ion intensities for representative peptides in HC and vivax malaria patients at different phases of disease progression. (**E**) Venn diagram depicting the overlap of proteins identified by iTRAQ measurements among four biological replicates. (**F**) Distinctive and overlapping differentially abundant proteins in FEB, DEF and CON stages of vivax malaria identified in iTRAQ-based quantitative proteomics analysis. (**G**) 2D-PCA plot showing discrimination between HC and FEB, DEF and CON stages of vivax malaria on the basis of proteome profiles.

Quantitative proteomics analysis was performed on a longitudinal cohort of non-severe vivax malaria patients using the same gel-based and gel-free proteomics approaches to capture the snapshots of dynamic serum proteome profiles during the acute and remission phases of the disease (Fig. 3A). In 2D-DIGE and MALDI-TOF/TOF analysis, 12 proteins were found to be significantly differentially regulated in the FEB stage of the infection (Student’s t-test and 1-way ANOVA; *p* ≤ 0.05). Among the differentially abundant proteins, 5 were up-regulated, and the remaining 7 were down-regulated (Table S6A). In HC *vs*. DEF analysis 9 proteins were found to be differentially abundant (7 up-regulated and 2 down-regulated) (Table S6B). Details of the differentially abundant protein spots and results obtained from their subsequent MS analysis have been summarized in the supplementary information (Table S6). Interestingly, some of the identified differentially abundant proteins such as HP, CP, SAA, and Apo E exhibited reversible fluctuations in their serum levels with the remission of the disease (Fig. 3B). Many of the protein spots detected in the DIGE gels remained unidentified in the MALDI TOF/TOF analysis and generated almost empty spectra, possibly due to their extremely low abundance in serum.

In the gel-free quantitative proteomics analysis, the iTRAQ labelled samples were analyzed using Q-TOF and Q-Exactive mass spectrometers. The iTRAQ ratios for all the proteins identified in Q-TOF and Q-Exactive mass spectrometric analysis along with their sequence coverage, protein score and unique peptide information are provided in supplementary information (Table S7). Combining four replicates, total 804 proteins were identified in the Q-TOF analysis at 1% false discovery rate (FDR) (Table S7A). Volcano plots showing *p* values versus difference of group means of FEB-HC, DEF-HC and CON-HC obtained in Q-TOF analysis are represented in Fig. 3C. MS/MS spectra of a few selected proteins with the insets depicting the iTRAQ reporter ion intensities for representative peptides in healthy controls and in vivax malaria during the three different phases of the infection (FEB, DEF and CON) are shown in the Fig. 3D. In the Q-Exactive analysis, a total of 342 proteins were identified at 1% FDR, out of which 153 were with ≥2 peptides (Table S7B). Proteins with ≥2 unique peptide matches as well as those with 1 peptide match, but detected in multiple replicates were selected for differential proteomics analysis. Comparative analysis of the proteins identified by Q-TOF and Q-Exactive mass spectrometers indicated an overlap of 62 proteins, among which 51 were found to be with ≥2 peptides. Of note, similar trend of differential abundance was observed for majority of the quantified proteins in both the mass spectrometric analyses independently. Normal distribution of total proteome, S-curve distributions of the differentially abundant proteins, and correlations among the different iTRAQ data sets for the longitudinal cohort of vivax malaria patients are represented in the Figure S2. In the Q-TOF analysis, different pooled samples were analysed as multiple biological replicates, and 97 proteins were found to be common in all the four replicates (Fig. 3E). Quantitative proteomic analysis based on the iTRAQ ratios indicated differential abundance (fold-change ≥1.2; adjusted *p*-value ≤0.05) of 25 proteins in FEB (12 up-regulated and 13 down-regulated), 28 proteins in DEF (8 up-regulated and 20 down-regulated), and 4 proteins in CON (1 up-regulated and 3 down-regulated) stages of the disease (Fig. 3F and Table S8). Principal component analysis (PCA) revealed distinct clustering among the different experimental groups (HC, FEB, DEF and CON) (Fig. 3G).

Eight proteins were found to be exhibiting differential abundance (*p*-value ≤0.05) only in the FEB stage, while serum abundances for two proteins were found to be dysregulated across all the three stages (Fig. 3F and Table S8A). Quite a few serum proteins, including Apo E, SAA, Leucine-rich alpha-2-glycoprotein (P02750) and Hemoglobin subunit zeta (P02008) were found to be up-regulated during the early febrile and defervescence stages, but seemed to return almost to normal levels during the convalescent stage (Table 2 and Table S8A). On the contrary, serum abundances for a few proteins such as HP and Apo A1 were found to be reduced in the FEB stage and/or DEF stages, but arrived nearly to the normal levels in the CON stage (Tables 2 and S8A). However, a number of proteins also showed similar trends of differential abundances across the three stages (either up-regulated or down-regulated), and a few serum proteins showed no significant alterations in their serum levels in any of the three stages as compared to the healthy controls. Interestingly, serum level of Immunoglobulin kappa variable 1–17 (P01610) was found to be altered only during the DEF and CON stages (*p* < 0.05), but not in the FEB stage of the infection (Table S8).

**Table 2:** Differentially abundant serum proteins identified in longitudinal cohorts of vivax malaria patients.

Sl NO.	Protein name	Uniprot Accession ID	Gene Name	Unique peptides^†^	(FEB/HC)		(DEF/HC)		(CON/HC)	
					Fold-change	Adjusted *p-*value	Fold-change	Adjusted *p-*value	Fold-change	Adjusted *p-*value
1	Apolipoprotein A-II	P02652	APOA2	8	0.41	0.047	0.47	0.073	0.64	0.244
2	Apolipoprotein A-I^$*^	P02647	APOA1	36	0.42	0.001	0.48	0.008	0.64	0.054
3	Heparin cofactor 2	P05546	SERPIND1	7	0.44	0.005	0.39	0.001	0.50	0.019
4	Apolipoprotein C-I	P02654	APOC1	3	0.45	0.003	0.40	0.00003	0.46	0.0002
5	Haptoglobin^$*^	P00738	HP	17	0.47	0.024	0.68	0.049	1.09	0.704
6	Inter-alpha-trypsin inhibitor heavy chain H2^$^	P19823	ITIH2	18	0.49	0.008	0.41	0.003	0.51	0.025
7	Serum paraoxonase/ arylesterase 1	P27169	PON1	5	0.64	0.074	0.54	0.007	0.69	0.122
8	Inter-alpha-trypsin inhibitor heavy chain H1	P19827	ITIH1	15	0.65	0.021	0.45	0.000	0.66	0.013
9	Conserved oligomeric Golgi complex subunit 4	Q9H9E3	COG4	8	0.66	0.148	0.55	0.018	0.85	0.360
10	Afamin	P43652	AFM	13	0.67	0.033	0.73	0.131	0.64	0.021
11	Apolipoprotein C-III	P02656	APOC3	5	0.68	0.031	0.68	0.041	0.73	0.209
12	Kininogen-1	P01042	KNG1	6	0.69	0.081	0.63	0.006	0.68	0.113
13	Fibronectin	P02751	FN1	22	0.72	0.030	0.58	0.005	0.63	0.009
14	Inter-alpha-trypsin inhibitor heavy chain H4	Q14624	ITIH4	24	0.72	0.043	0.63	0.056	0.71	0.035
15	Complement C5	P01031	C5	8	0.73	0.067	0.67	0.038	0.68	0.023
16	Complement C3	P01024	C3	89	0.74	0.159	0.66	0.032	0.79	0.171
17	Clusterin	P10909	CLU	12	0.75	0.106	0.68	0.003	0.71	0.092
18	Apolipoprotein B-100	P04114	APOB	145	0.77	0.030	0.63	0.002	0.76	0.029
19	Serum amyloid P-component	P02743	APCS	3	0.78	0.216	0.62	0.025	0.70	0.086
20	Complement C4-A	P0C0L4	C4A	7	0.85	0.104	0.73	0.014	1.10	0.703
21	Hemopexin^$*^	P02790	HPX	12	1.22	0.05	1.12	0.163	1.00	0.295
22	Apolipoprotein E^*^	P02649	APOE	11	1.24	0.05	1.16	0.045	0.87	0.715
23	Alpha-1-acid glycoprotein 1	P02763	ORM1	8	1.25	0.139	1.49	0.041	1.46	0.088
24	Biotinidase	P43251	BTD	2	1.30	0.045	1.12	0.555	1.10	0.685
25	Alpha-1-antichymotrypsin^$^	P01011	SERPINA3	26	1.42	0.036	1.28	0.379	1.22	0.096
26	Alpha-1-antitrypsin^$^	P01009	SERPINA1	40	1.47	0.115	1.51	0.004	1.50	0.164
27	Leucine-rich alpha-2-glycoprotein^$^	P02750	LRG1	7	1.83	0.005	1.63	0.037	1.49	0.047
28	Cell growth-regulating nucleolar protein	Q9NX58	LYAR	13	1.85	0.056	2.10	0.044	2.26	0.157
29	Serum amyloid A-1^$*^	P0DJI8	SAA1	6	2.49	0.008	1.60	0.023	2.15	0.018
30	Carbonic anhydrase 1	P00915	CA1	4	2.61	0.014	1.37	0.207	1.43	0.048
31	Hemoglobin subunit alpha	P69905	HBA1	7	2.63	0.006	1.49	0.025	1.33	0.032
32	Hemoglobin subunit beta	P68871	HBB	8	2.70	0.003	1.66	0.057	1.47	0.047
33	Hemoglobin subunit delta	P02042	HBD	7	3.77	0.005	1.89	0.101	1.83	0.036
34	C-reactive protein	P02741	CRP	4	3.91	0.003	6.69	0.006	2.45	0.001
35	Hemoglobin subunit zeta	P02008	HBZ	5	4.83	0.011	1.88	0.023	2.21	0.041

^#^This is a partial list for some selected candidates (*p* < 0.05 in at least one comparison) identified in iTRAQ and 2D-DIGE-based quantitative proteomics analysis; complete lists of the identified differentially abundant proteins are provided under supplementary information (Tables S6, S7 and S8). ^†^Median value for the identified unique peptides in different biological replicates is represented. ^$^Differential abundances for these candidates are also identified in 2D-DIGE (details are provided in Table S6). *Differential serum abundances of these proteins are validated by ELISA (details are provided in Table S13A).

### Modulation of diverse physiological pathways in *P*. *vivax* infection

**Figure 4:**
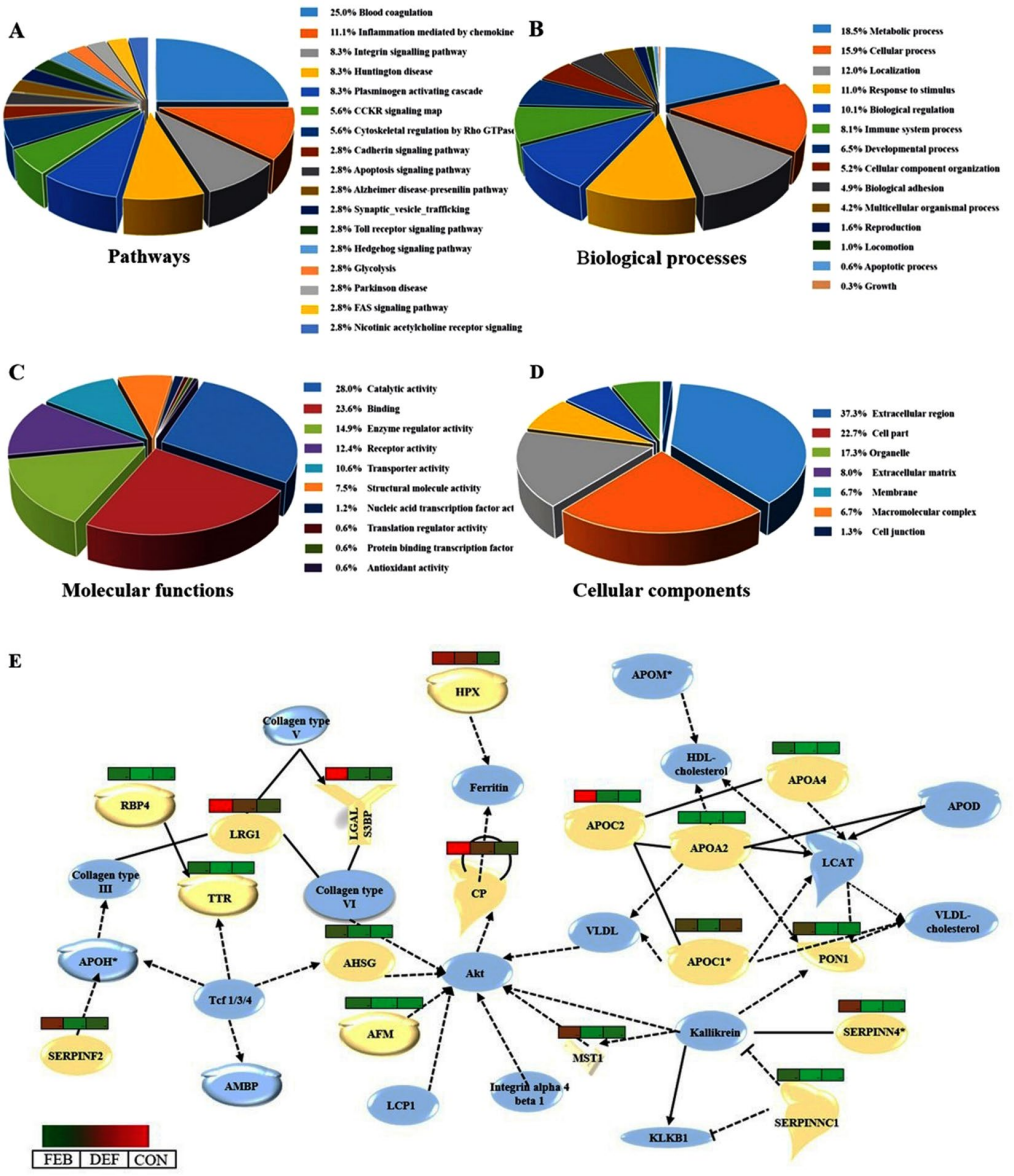
Functional clustering and physiological pathways associated with the differentially abundant proteins identified in vivax malaria. Pie charts showing the physiological pathways (**A**), biological process (**B**), molecular functions (**C**) and cellular components (**D**) related to the differentially abundant proteins identified in both LPVM and MPVM patients (combined list). Separate analyses of the two classes of vivax malaria patients (i.e. LPVM and MPVM) are provided in supplementary information (Fig. S3A and B). (**E**) Differential abundance of the serum proteins (light yellow) involved in lipid metabolism and molecular transport are depicted in a longitudinal cohort of vivax malaria patients (FEB, DEF and CON stages). The node color represents up (red) and down-regulated (green) proteins within the categories, and the color intensity demonstrates the magnitude of differential abundances. Light blue symbols represent the associated proteins identified in the functional analysis for which the differences in serum levels have not achieved statistical significance (*p* > 0.05) in our study.

After identifying the differentially abundant serum proteins in the low and moderate parasitemic malaria patients, we were interested to find out their molecular and biological functions and association with different biological processes and physiological pathways (Table S9 and Fig. S3). Our bioinformatics analysis indicates that blood coagulation and plasminogen activating cascade are the main physiological pathways associated with the differentially abundant serum proteins identified in the vivax malaria patients (Fig. 4A). Many of the altered proteins were found to be associated with metabolic and cellular processes, localization, response to stimulus, and biological regulations (Fig. 4B). Molecular function analysis specified that the differentially abundant proteins were related with diverse types of molecular functions; including catalytic activity, binding, enzyme regulator activity, and receptor and transporter activities (Fig. 4C). Most of the proteins were found to be resided at the extracellular regions and within the cell parts, while some were organelle specific or components of macromolecular complexes (Fig. 4D).

Differentially abundant serum proteins identified during the FEB, DEF, and CON stages of the disease were also subjected to bioinformatics analysis for functional clustering. Results are summarized in details under the supplementary information (Table S10 and Fig. S4). According to IPA analysis, the differentially abundant proteins identified in the longitudinal cohort of vivax malaria patients provide evidences of alterations in multiple physiological pathways, mostly during the acute phase of the infection. The most prominent canonical pathways included acute phase response signaling, Liver X receptor/Retinoid X receptor (LXR/RXR) activation, complement and coagulation systems (Table S10). Lipid metabolism and molecular transport was identified as one of the top-scoring network (Fig. 4E). In the FEB and DEF stages of the infection maximum numbers of differentially abundant serum proteins involved in various physiological pathways/networks were indentified, while both the number of candidates and their respective levels of alterations (fold-change) were found to be reduced during abatement of the fever (CON stage). Many of the differentially altered proteins identified in the FEB stage, exhibited nearly normal level during the CON stage, when the patients gradually return to health after illness.

### Measurement of serum concentrations of differentially abundant proteins by ELISA

**Figure 5:**
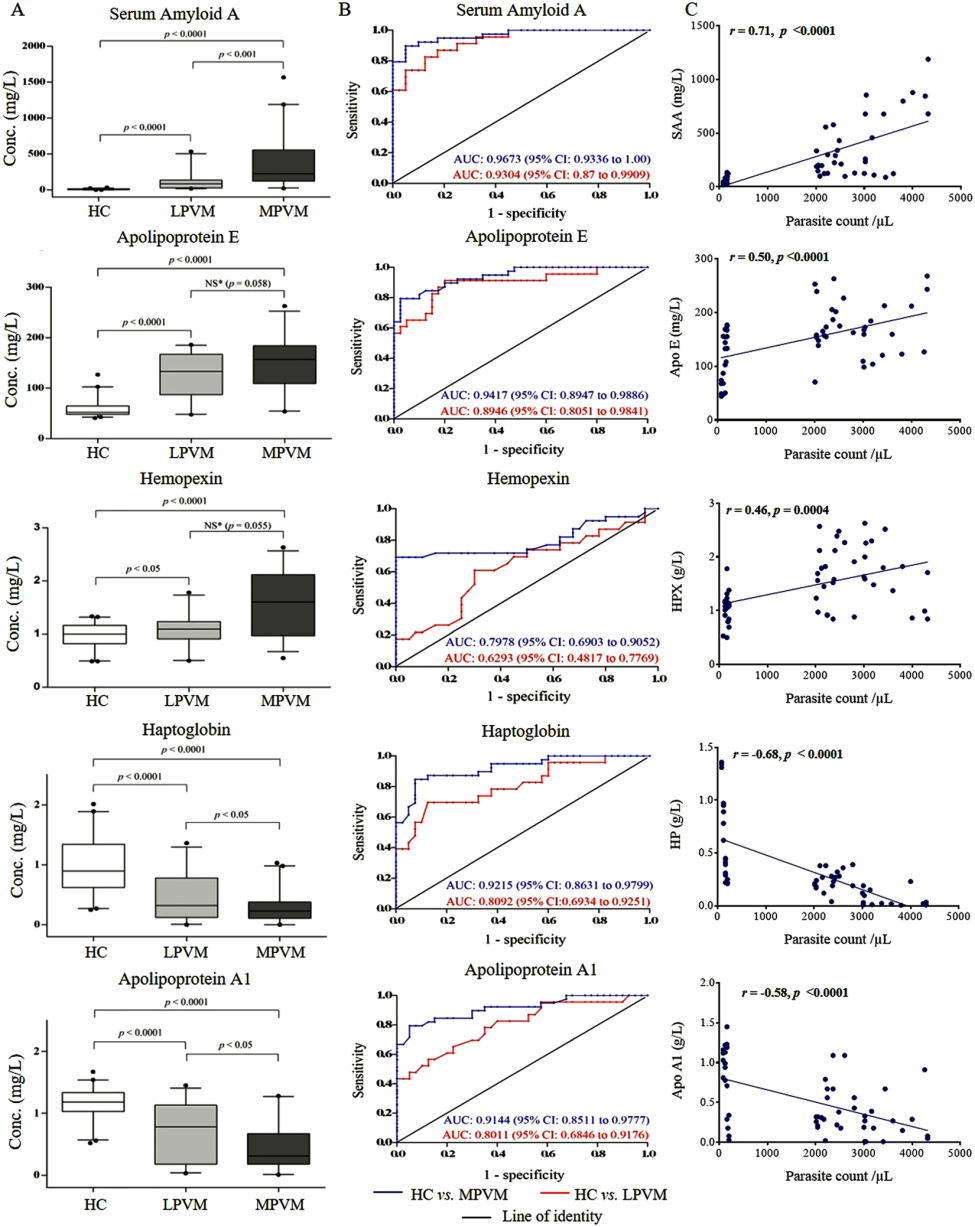
ELISA-based measurement of serum proteins in different parasitemic vivax malaria patients. (**A**) Measurement of serum levels of different proteins in healthy controls (n = 40) and low and moderately-high parasitemic vivax malaria patients (LPVM (n = 23) and MPVM (n = 40)) by ELISA. SAA exhibited a steady increase (*p* < 0.05) in its serum abundance, whereas the serum levels of HP and ApoA1 were found to be sequentially decreased (*p* < 0.05) with respect to the increase in parasite count. **Indicates *p* < 0.001, *indicates 0.001 < *p* < 0.05, and NS indicates *p* > 0.05 based on a Mann-Whitney test. (**B**) Receiver operating characteristics (ROC) curves for evaluation of the sensitivity and specificity of different serum proteins for LPVM (red lines) and MPVM (blue lines). ROC curves demonstrating that SAA, Apo A1 and HP can predict vivax malaria efficiently (AUC > 0.80) even at a low-parasitemic level. (**C**) Correlation analysis between parasitemia and concentration of different serum proteins in combined groups consists of both LPVM and MPVM patients. Serum levels of SAA and HP exhibited substantial correlation with parasitic count in malaria patients (r > 0.6).

In order to validate the findings obtained in our quantitative proteomics analysis, serum abundances of 5 selected proteins were measured in the sera of LPVM and MPVM patients and HC study cohorts by ELISA (Table S11A). In the validation study, candidates were selected on the basis of their level of differential abundances observed in the proteomics analysis, possible connection of the proteins with vivax malaria pathogenesis, and availability of the required ELISA kits and reagents. Seeing that multiple comparison (Kruskal–Wallis) test exhibited a significant difference for most of the proteins (Table S11B), additional statistical analysis for pair-wise comparison was performed using Mann Whitney U test (Table S11C). SAA exhibited a gradual increase in its serum abundance, while the serum levels of HP and ApoA1 were found to be sequentially decreased with respect to the increase in parasite load. Serum abundance of HPX and ApoE were found to be higher in both LPVM and MPVM patients compared to the HC. However, differential abundance of Apo E, Apo A1, and HP between the LPVM and MPVM patient cohorts was found to be statistically insignificant (*p* > 0.05) (Fig. 5A). ROC curves indicate SAA, Apo A1 and HP are efficient predictor proteins (AUC > 0.80) for vivax malaria even at a low-parasitemic level (Fig. 5B and Table S12). Serum levels of SAA and HP exhibited correlation (negative or positive) with the parasitic burden in malaria patients showing a Pearson’s correlation coefficient (r) >0.6 at *p* < 0.0001 (Fig. 5C). Serum levels of these proteins were also measured in DF patients to evaluate their specificity towards malaria. Interestingly, serum abundance of HP was found to be higher in DF patients compared to HC, while its serum level was substantially low in malaria patients. SAA, Apo E, Apo A1 and HPX exhibited similar trends of differential abundance in malaria and DF patients (compared to HC); however, the levels of their dysregulation were found to be much higher in the malaria patients (Fig. S5). Additionally, we have compared these dysregulated proteins identified in the LPVM and MPVM cohorts of our study with their serum abundance in severe vivax malaria patients (measurements of these proteins in severe malaria have been re-analyzed from a recently published article from our research group)^39^. Importantly, some of these differentially abundant proteins such as HP, HPX, Apo A1 and Apo E, which exhibited gradual alterations in serum with increase in parasitemia, also exhibited significant perturbations in the severe malaria patients (significant increase/decrease as compared to non-severe malaria) (Fig. S6).

**Figure 6:**
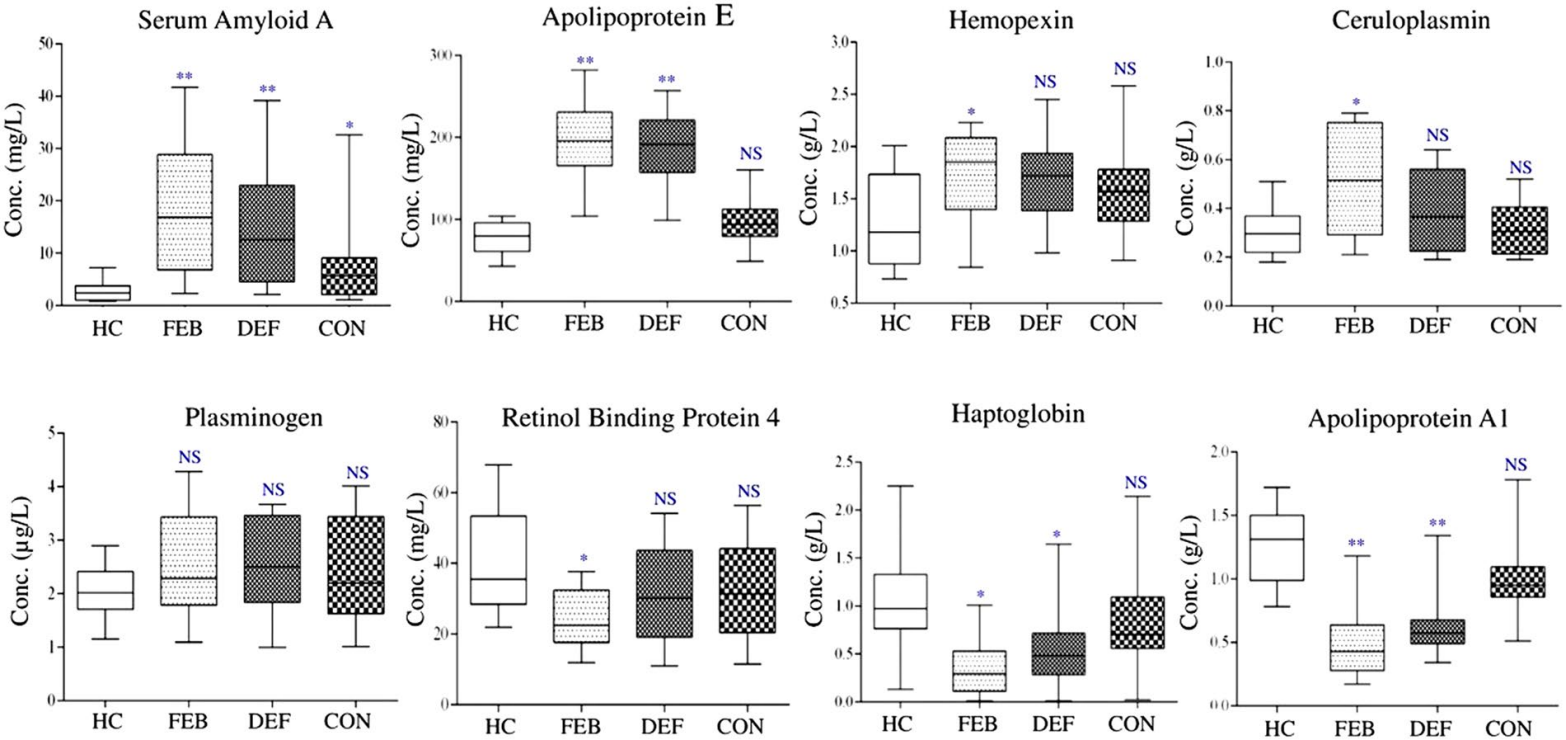
ELISA-based measurement of serum proteins in a longitudinal cohort of vivax malaria patients. Measurement of serum levels of eight differentially abundant proteins in HC (n = 10) and a longitudinal cohort (FEB, DEF and CON stages) of vivax malaria patients (n = 10) performed by ELISA. Maximum levels of dysregulation in the serum abundance of these proteins were observed during the acute phase of the infection (FEB), while the amplitude of alteration for these proteins was gradually decreased with the remission of the disease. **Indicates *p* < 0.001, *indicates 0.001 < *p* < 0.05, and NS indicates *p* > 0.05 based on a Mann-Whitney test.

Analysis of the longitudinal cohort of vivax malaria patients by ELISA indicated reversible alterations (compared to the normal serum levels) in the serum abundances of HP, ApoE and Apo A1 during the acute and remission phases of the infection (Table S13A). Serum levels of these proteins were highly altered during the FEB stage of the disease. Interestingly, the scale of alteration for these proteins was gradually decreased during remission of the disease; their serum levels still remained high/low (compared to the normal range) in the DEF stage, but reached almost the basal level during the CON stage (Fig. 6). For HPX, CP and RBP4 differential abundance was observed only in the FEB stage of the infection, while alterations in their serum abundances during the DEF and CON stages were found to be statistically insignificant (*p* > 0.05) (Tables S13B and S13C). Consequently, discrimination accuracy of most of the differentially abundant proteins for healthy control and malaria patients were highest at the FEB stage and reduced during the DEF and CON stages of the disease (Table S14 and Fig. S7). Taken together, the findings obtained from the ELISA-based measurements validated the observations obtained in our discovery-phase quantitative proteomics analyses.

## Discussion

Identification of serum/plasma proteins, which exhibit altered abundance at the onset and during the acute phase of any infection, could be informative to understand the pathobiology of different infectious diseases and host responses against the invading pathogens^42–44^. To this end, in recent years, several research groups including ours have investigated alterations in serum/plasma proteome in severe and non-severe falciparum^23, 24, 27, 32^ and vivax malaria^28, 39, 45^ to study malaria pathogenesis. In all these studies, serum/plasma proteome of the malaria patients have been analyzed during the febrile stages of the infection, either at the onset of the disease or at the fastigium stage. However, temporal profiling of serum/plasma proteome during acute and remission stages in malaria, which can provide snapshots of the transient and enduring alterations in serum proteome during the FEB, DEF and CON stages, has not been reported hitherto. Here, for the first time we report serum proteomic alterations in a longitudinal cohort of *P*. *vivax* infected patients to elucidate host responses when fever is established (temperature of the body reaches above higher normal level), during the stage when the temperature comes down to normal, and also during the gradual recovery of health after the illness. The three stages discussed in our study have been categorically chosen depending upon the clinical course of uncomplicated vivax malaria. Analysis of the early febrile stage represents host proteome profile immediately after onset of the infection, before administering any anti-malarial drugs. The second, defervescence stage, reflects any immediate change in blood proteome at early recovery phase, while the convalescent stage indicates a phase after administration of 14 days radical cure treatment with primaquine and a complete recovery, when none of the patients displayed any apparent symptoms of malaria.

In this study, we have analyzed correlations of dysregulated serum proteins with different clinicopathological parameters and investigated their involvements in diverse physiological pathways and biological processes. Reduction in the hemoglobin level during the early stage of plasmodial infection and its gradual recovery to the normal level with the disease remission, as well as its reduction with the increase in parasitemia are suggestive of increased hemolysis and decreased rate of erythrocyte production in malaria patients. This observation is consistent with earlier reports^46, 47^. Likewise, platelet counts, which were reported earlier to be consistently low in vivax malaria^48–50^, were also found to be reduced in malaria patients in our study, reflecting the possibilities of sequestration of platelets by macrophages in the spleen due to immune mediated injury as well as platelet clump formation with the infected erythrocytes^51, 52^. Increased levels of liver enzymes were observed with an increase in parasitemia and degree of hemolysis, as reflected by the higher AST level in MPVM compared to the HC and LPVM cohorts. Liver function derangements have been studied earlier in malaria^53^. Other biochemical parameters such as bilirubin and ALP were also found to be elevated in vivax malaria, but returned to the normal levels during the convalescent stage indicating possibilities of liver involvement in malaria that could be attributed to mononuclear infiltration of the liver leading to an intrahepatic cholestasis.

Comparative analysis of the serum proteome profiles of non-severe vivax malaria patients with varying levels of parasitemia indicated some prominent differences in the serum proteome patterns of low and moderately-high parasitemic patients. Some of the differentially abundant proteins such as SAA, CRP, Titin, Apo E exhibited gradual alterations in their serum abundances with an increase in parasitemia. However, some of the identified proteins such as HPX, Vitronectin, Clusterin and Apo E exhibited nearly equal levels of differential serum abundance in both patient groups as compared to healthy controls, indicating some possibilities of differential host responses due to the varying levels of parasitemia.

Many of the dysregulated proteins were found to be acute phase reactants or acute phase proteins (APPs), followed by the proteins involved in complement and coagulation cascades. Previously, a time course analysis of falciparum malaria patients during antiparasitic therapy demonstrated complex interactions of inflammatory and coagulatory factors during the acute phase of the disease^54^. In this direction, an earlier study describing proteomics analysis of longitudinal cohorts of dengue fever and dengue hemorrhagic fever patients also reported altered serum levels of a large number of acute phase reactants and cytokines^55^. Non-specific resistance against the pre-erythrocytic stages of *Plasmodium* can be generated by APPs^56^. In malaria patients, the parasite selectively invades the red blood cells, multiplies within them and ultimately ruptures to release merozoites into circulation^57, 58^. In this process, several proteins such as hemoglobin subunits, which remain confined in the interior of RBCs, are also released in the bloodstream. Consequently, inflammatory responses are triggered by the body against the parasites^59^, which can lead to the activation of various complement proteins^60, 61^. Extreme dysregulation in the serum levels of several APPs including SAA, CRP, Leucine-rich alpha-2-glycoprotein (P02750), Alpha-1-antichymotrypsin, and Alpha-1-antitrypsin (P01009) observed during the FEB stage of the infection clearly indicates generation of strong inflammatory responses against the malaria parasites almost immediately after onset of the infection.

Bioinformatics analysis on the basis of our identified differentially abundant serum proteins indicates modulations in lipid metabolism and transport in the vivax malaria patients (Fig. 4E). Lipids are synthesized within liver, and exo-erythrocytic stage of the malaria parasites also happens in hepatocytes. Initiation of erythrocytic stage with multiplication of a single merozoite to multiple copies (8 to 32) requires a considerable amount of cholesterol for membrane formation. Malarial parasites lack *de novo* cholesterol synthetic pathway, and therefore need uptake of cholesterol and other nutrients through parasitophorous vacuolar membrane (PVM) to ensure their survival and propagation^62^. Our study indicates that apolipoproteins of high-density lipoproteins-cholesterol (HDL-C) such as Apolipoprotein CI (APOC1; P02654), Apolipoprotein C2 (APOC2; P02655), Apolipoprotein A4 (APOA4; P06727) and Paraoxonase 1(PON1; P27169) were down-regulated along with the alterations in HDL-C transport (LCAT) in vivax malaria. To this end, lower HDL level in malaria patients has been reported earlier^63, 64^. An earlier report on the meta-analysis of serum lipid and lipoprotein changes indicates that the normalization of lipid profiles happens quite slowly in malaria patients, and takes over one to six months to reach the basal levels following the infection^65^. Intriguingly, we observed that serum levels of the proteins involved in HDL metabolism and transport remained lower not only in the FEB stage, but modulations in their serum levels were also observed during the DEF and CON stages of malaria.

This study provided a comprehensive representation of the diverse alterations in serum proteome profiles of vivax malaria patients with low and moderately-high parasitemia, as well as regarding the phase-specific temporal protein profiles during the acute and convalescent phases of the infection. More importantly, our bioinformatics analyses provided evidences of intricate associations of many of the identified dysregulated proteins with crucial biological processes and physiological pathways such as blood coagulation and plasminogen activating cascade, complement systems, lipid metabolism and molecular transport, and acute phase response signalling. However, it is certainly difficult to speculate the exact mechanisms behind such diverse alterations in blood proteome as the factors introducing these alterations could be partly host related, or might be parasite-related (secondary to parasite metabolism), and there could also be some cumulative effect of interactions between the host and the parasite. Therefore, it remains challenging to unravel the precise mechanisms behind such observations through analysis of clinical specimens due to the presence of an entangled web of physiological networks that are controlled by both host and parasite under the complicated diseased conditions. Specific functional assays with the *ex vivo* grown malaria parasites may provide some further insights, and could be an interesting continuation of this present study. Taken together, we are able to get a glimpse of the composite depictions of vivax malaria pathogenesis through a proteome level analysis. This study may pave the way for future proteomics and integrated multi-omics investigations on both the host and the parasite for obtaining a better perspective of vivax malaria pathogenesis.

## Electronic supplementary material

Supplementary Information

## References

[1] Rahimi BA (2014). Severe vivax malaria: a systematic review and meta-analysis of clinical studies since 1900. Malar. J..

[2] Baird JK (2013). Evidence and implications of mortality associated with acute *Plasmodium vivax* malaria. Clin. Microbiol. Rev..

[3] Tjitra, E. *et al*. Multidrug-Resistant *Plasmodium vivax* Associated with Severe and Fatal Malaria: A Prospective Study in Papua, Indonesia. *PLoS Med*. **5** (2008). 10.1371/journal.pmed.0050128 PMC2429950 18563962

[4] Anstey NM, Russell B, Yeo TW, Price RN (2009). The pathophysiology of vivax malaria. Trends Parasitol..

[5] Price RN (2007). Vivax malaria: neglected and not benign. Am. J. Trop. Med. Hyg..

[6] Hemmer CJ (2006). Stronger host response per parasitized erythrocyte in *Plasmodium vivax* or ovale than in *Plasmodium falciparum* malaria. Trop. Med. Int. Heal. TM IH.

[7] Prajapati, S. K. & Singh, O. P. Insights into the invasion biology of *Plasmodium vivax*. *Front. Cell. Infect. Microbiol*. **3** (2013). 10.3389/fcimb.2013.00008 PMC3587795 23469364

[8] Poespoprodjo JR (2009). Vivax malaria: a major cause of morbidity in early infancy. Clin. Infect. Dis. An Off. Publ. Infect. Dis. Soc. Am..

[9] Karyana M (2008). Malaria morbidity in Papua Indonesia, an area with multidrug resistant *Plasmodium vivax* and *Plasmodium falciparum*. Malar. J..

[10] Maitland, K. *et al*. The interaction between *Plasmodium falciparum* and *P. vivax* in children on Espiritu Santo island, Vanuatu. *Trans. R. Soc. Trop. Med. Hyg*. **90**, 614–20. 10.1016/s0035-9203(96)90406-x 9015495

[11] Smith, T. *et al*. Prospective risk of morbidity in relation to malaria infection in an area of high endemicity of multiple species of Plasmodium. *Am. J. Trop. Med. Hyg*. **64**, 262–7. 10.4269/ajtmh.2001.64.262 11463113

[12] WHO|World Malaria Report 2013.

[13] Galinski MR, Barnwell JW (2008). *Plasmodium vivax*: who cares?. Malar. J..

[14] Mendis, K., Sina, B. J., Marchesini, P. & Carter, R. The neglected burden of *Plasmodium vivax* malaria. *Am. J. Trop. Med. Hyg*. **64**, 97–106. 10.4269/ajtmh.2001.64.97 11425182

[15] Gething PW (2012). A long neglected world malaria map: *Plasmodium vivax* endemicity in 2010. PLoS Negl. Trop. Dis..

[16] Carlton JM, Sina BJ, Adams JH (2011). Why Is *Plasmodium vivax* a Neglected Tropical Disease?. PLoS Negl Trop Dis.

[17] Broek IVD (2006). Evaluation of Three Rapid Tests for Diagnosis of *P. Falciparum* and *P. Vivax* Malaria in Colombia. Am. J. Trop. Med. Hyg..

[18] Murray CK, Bennett JW (2009). Rapid Diagnosis of Malaria. Interdiscip. Perspect. Infect. Dis..

[19] WHO|Malaria rapid diagnostic test performance: results of WHO product testing of malaria RDTs: round 6 (2014–2015).

[20] Wongsrichanalai C, Barcus MJ, Muth S, Sutamihardja A, Wernsdorfer WH (2007). A Review of Malaria Diagnostic Tools: Microscopy and Rapid Diagnostic Test (RDT). Am. J. Trop. Med. Hyg..

[21] Bautista JM, Marín-García P, Diez A, Azcárate IG, Puyet A (2014). Malaria proteomics: Insights into the parasite–host interactions in the pathogenic space. J. Proteomics.

[22] Venkatesh A (2016). Proteomics of *Plasmodium vivax* malaria: new insights, progress and potential. Expert Rev. Proteomics.

[23] Kassa FA (2011). New inflammation-related biomarkers during malaria infection. PLoS One.

[24] Burté F (2012). Severe childhood malaria syndromes defined by plasma proteome profiles. PLoS One.

[25] Gitau EN, Kokwaro GO, Karanja H, Newton CRJC, Ward SA (2013). Plasma and cerebrospinal proteomes from children with cerebral malaria differ from those of children with other encephalopathies. J. Infect. Dis..

[26] Bachmann J (2014). Affinity proteomics reveals elevated muscle proteins in plasma of children with cerebral malaria. PLoS Pathog..

[27] Ray, S. *et al*. Proteomic analysis of *Plasmodium falciparum* induced alterations in humans from different endemic regions of India to decipher malaria pathogenesis and identify surrogate markers of severity. *J. Proteomics*, **127**, 103–13 (2015). 10.1016/j.jprot.2015.04.032 25982387

[28] Ray S (2012). Serum proteome analysis of vivax malaria: An insight into the disease pathogenesis and host immune response. J. Proteomics.

[29] Acharya P (2011). Clinical proteomics of the neglected human malarial parasite *Plasmodium vivax*. PLoS One.

[30] Anderson DC (2015). *Plasmodium vivax* trophozoite-stage proteomes. J. Proteomics.

[31] Srivastava R (2012). Serum profiling of leptospirosis patients to investigate proteomic alterations. J. Proteomics.

[32] Ray S (2012). Proteomic investigation of falciparum and vivax malaria for identification of surrogate protein markers. PLoS One.

[33] Thomas RD, Westengard JC, Hay KL, Bull BS (1993). Calibration and validation for erythrocyte sedimentation tests. Role of the International Committee on Standardization in Hematology reference procedure. Arch. Pathol. Lab. Med..

[34] Hunt, S. M. N. *et al*. Optimal replication and the importance of experimental design for gel-based quantitative proteomics. *J. Proteome Res*. **4**, 809–19. 10.1021/pr049758y 15952727

[35] Sharma S, Ray S, Moiyadi A, Sridhar E, Srivastava S (2014). Quantitative proteomic analysis of meningiomas for the identification of surrogate protein markers. Sci. Rep..

[36] Deutsch, E. W. *et al*. The ProteomeXchange consortium in 2017: supporting the cultural change in proteomics public data deposition. *Nucleic Acids Res*. **45**, D1100–D1106 (2017). 10.1093/nar/gkw936 PMC5210636 27924013

[37] Tyanova S (2016). The Perseus computational platform for comprehensive analysis of (prote)omics data. Nat. Methods.

[38] Sharma S (2015). Multipronged quantitative proteomic analyses indicate modulation of various signal transduction pathways in human meningiomas. Proteomics.

[39] Ray S (2016). Clinicopathological analysis and multipronged quantitative proteomics reveal oxidative stress and cytoskeletal proteins as possible markers for severe vivax malaria. Sci. Rep..

[40] Huang DW, Sherman BT, Lempicki RA (2009). Systematic and integrative analysis of large gene lists using DAVID bioinformatics resources. Nat. Protoc..

[41] Mi H, Muruganujan A, Casagrande JT, Thomas PD (2013). Large-scale gene function analysis with the PANTHER classification system. Nat. Protoc..

[42] Anderson NL (2010). The clinical plasma proteome: a survey of clinical assays for proteins in plasma and serum. Clin. Chem..

[43] Ray S, Patel SK, Kumar V, Damahe J, Srivastava S (2014). Differential expression of serum/plasma proteins in various infectious diseases: specific or nonspecific signatures. Proteomics. Clin. Appl..

[44] Ray S (2011). Proteomic technologies for the identification of disease biomarkers in serum: advances and challenges ahead. Proteomics.

[45] Bahk YY (2010). Proteomic analysis of haptoglobin and amyloid A protein levels in patients with vivax malaria. Korean J. Parasitol..

[46] Castro-Gomes, T. *et al*. Potential immune mechanisms associated with anemia in *Plasmodium vivax* malaria: a puzzling question. *Infect. Immun*. **82**, 3990–4000 (2014). 10.1128/IAI.01972-14 PMC4187874 25092911

[47] Douglas NM (2012). The anaemia of *Plasmodium vivax* malaria. Malar. J..

[48] Kueh YK, Yeo KL (1982). Haematological alterations in acute malaria. Scand. J. Haematol..

[49] Oh MD (2001). Clinical features of vivax malaria. Am. J. Trop. Med. Hyg..

[50] Rojanasthien S, Surakamolleart V, Boonpucknavig S, Isarangkura P (1992). Hematological and coagulation studies in malaria. J. Med. Assoc. Thai..

[51] Makkar RPS, Monga SMA, Gupta AK (2002). *Plasmodium vivax* malaria presenting with severe thrombocytopenia. Brazilian J. Infect. Dis..

[52] Rodríguez-Morales AJ (2005). Occurrence of Thrombocytopenia in *Plasmodium vivax* Malaria. Clin. Infect. Dis..

[53] Tangpukdee N (2006). Minor liver profile dysfunctions in *Plasmodium vivax*, P. malaria and P. ovale patients and normalization after treatment. Korean J. Parasitol..

[54] Vogetseder A, Ospelt C, Reindl M, Schober M, Schmutzhard E (2004). Time course of coagulation parameters, cytokines and adhesion molecules in *Plasmodium falciparum* malaria. Trop. Med. Int. Health.

[55] Kumar Y (2012). Serum Proteome and Cytokine Analysis in a Longitudinal Cohort of Adults with Primary Dengue Infection Reveals Predictive Markers of DHF. PLoS Negl Trop Dis.

[56] Taylor-Robinson AW (2000). Increased production of acute-phase proteins corresponds to the peak parasitaemia of primary malaria infection. Parasitol. Int..

[57] Miller LH, Baruch DI, Marsh K, Doumbo OK (2002). The pathogenic basis of malaria. Nature.

[58] Florens L (2002). A proteomic view of the *Plasmodium falciparum* life cycle. Nature.

[59] Artavanis-Tsakonas K, Tongren JE, Riley EM (2003). The war between the malaria parasite and the immune system: immunity, immunoregulation and immunopathology. Clin. Exp. Immunol..

[60] Roestenberg M (2007). Complement activation in experimental human malaria infection. Trans. R. Soc. Trop. Med. Hyg..

[61] Biryukov S, Stoute JA (2014). Complement activation in malaria: friend or foe?. Trends Mol. Med..

[62] Lingelbach K, Joiner KA (1998). The parasitophorous vacuole membrane surrounding Plasmodium and Toxoplasma: an unusual compartment in infected cells. J. Cell Sci..

[63] Khovidhunkit W, Memon RA, Feingold KR, Grunfeld C (2000). Infection and inflammation-induced proatherogenic changes of lipoproteins. J. Infect. Dis..

[64] P.C, O. R. T. C. Serum Lipid Profile and Hepatic Dysfunction in Moderate *Plasmodium Falciparum* Infection. *Global Journal of Medical Research* **13** (2013).

[65] Visser BJ, Wieten RW, Nagel IM, Grobusch MP (2013). Serum lipids and lipoproteins in malaria–a systematic review and meta-analysis. Malar. J..

