# Supplementary material for: Quantitative Proteomics Analysis of Plasmodium vivax Induced Alterations in Human Serum during the Acute and Convalescent Phases of Infection
Source: Sci Rep. 2017 Jun 30;7:4400. doi: 10.1038/s41598-017-04447-5 (PMC5493610; doi:10.1038/s41598-017-04447-5)
Supplement: Supplementary file 1 — Supplementary Information [file 41598_2017_4447_MOESM1_ESM.pdf]

Original Research Article

Quantitative Proteomics Analysis of *Plasmodium vivax* Induced Alterations in Human Serum during the Acute and Convalescent Phases of Infection

Sandipan Ray[1†*], Sandip K. Patel[1*], Apoorva Venkatesh[1], Gangadhar Chatterjee[3], Naziya N. Ansari[1], Nithya J. Gogtay[2], Urmila M. Thatte[2], Prajakta Gandhe[2], Santosh G. Varma[3], Swati Patankar[1] and Sanjeeva Srivastava[1]*

[1]Department of Biosciences and Bioengineering, Indian Institute of Technology Bombay, Powai, Mumbai 400076, India

[2]Departments of Clinical Pharmacology, Seth GS Medical College & KEM Hospital, Parel, Mumbai 400012, India

[3]Dept of Biochemistry, Grant Govt Medical College and Sir JJ Group of Hospitals, Byculla, Mumbai 400008, India

*These authors contributed equally to this work

†Present address: Department of Clinical Biochemistry, Metabolic Research Laboratories, Wellcome Trust-Medical Research Council Institute of Metabolic Science, University of Cambridge, Addenbrooke's Hospital, Cambridge CB2 0QQ, United Kingdom

[*]Correspondence: Dr. Sanjeeva Srivastava; E-mail: sanjeeva@iitb.ac.in

Phone: +91-22-2576-7779, Fax: +91-22-2572-3480

A. Supplementary Figures

Figure S1. Parasitemia distribution plot for the vivax malaria patients enrolled in this study.

Figure S2A. Histograms showing normal distribution of iTRAQ data sets (after normalization) for HC and different parasitemic vivax malaria patients.

Figure S2B. Distribution of the differentially abundant proteins in low and moderately high parasitemic vivax malaria patients identified in iTRAQ-based quantitative proteomics analysis.

Figure S2C. Scatter plots exhibiting correlations among the serum proteome profiles of HC and different parasitemic vivax malaria patients (LPVM and MPVM).

Figure S2D. Histograms showing normal distribution of different iTRAQ data sets (after normalization) for longitudinal cohorts of vivax malaria patients.

Figure S2E. Distribution of the differentially abundant proteins in a longitudinal cohort of vivax malaria patients identified in iTRAQ-based quantitative proteomics analysis.

Figure S2F. Scatter plots exhibiting correlations (Pearson correlation coefficient) among the different iTRAQ data sets (analyzed by using Q-TOF) for longitudinal cohorts of vivax malaria patients.

Figure S3. Functional clustering, biological functions and physiological pathways associated with the differentially abundant proteins identified in low (A) and moderately high (B) parasitemic vivax malaria patients.

Figure S4. Different physiological pathways associated with the differentially abundant proteins identified in the longitudinal cohort of vivax malaria patients (The [networks, functional analyses, etc.] were generated through the use of QIAGEN's Ingenuity Pathway Analysis (IPA®, QIAGEN Redwood City, www.qiagen.com/ingenuity)).

Figure S5. ELISA-based measurement of serum levels of some selected differentially abundant proteins in healthy controls, vivax malaria and dengue fever patients.

Figure S6. ELISA-based measurement of serum levels of some selected differentially abundant proteins in low and moderately high parasitemic non-severe vivax malaria and severe vivax malaria patients.

Figure S7. Receiver operating characteristic (ROC) curves depicting the accuracy of a few differentially abundant serum proteins for prediction of FEB, DEF and CON stages of vivax malaria.

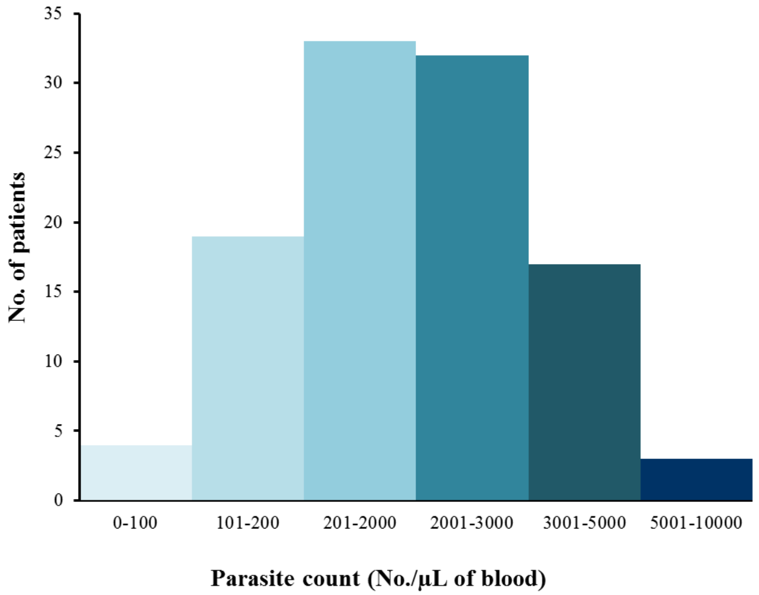

Figure S1. Parasitemia distribution plot for the vivax malaria patients enrolled in this study.

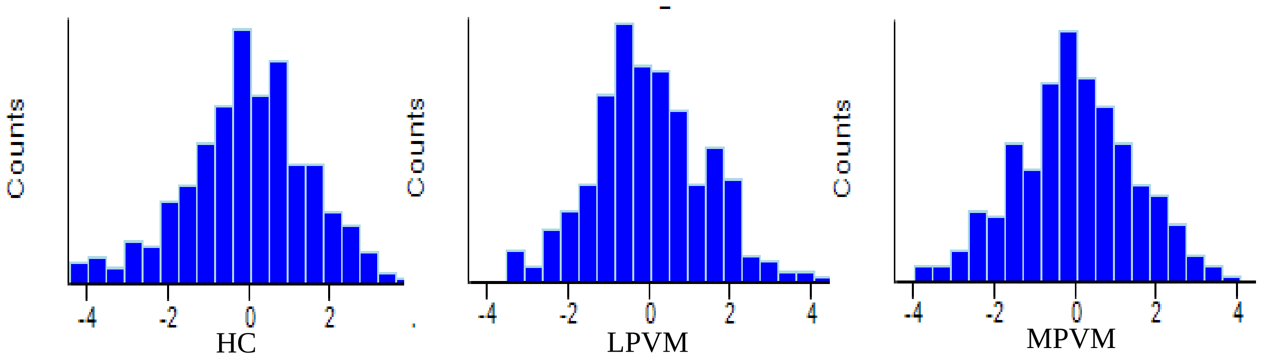

Figure S2A. Histograms showing normal distribution of iTRAQ data sets (after normalization) for HC and different parasitemic vivax malaria patients.

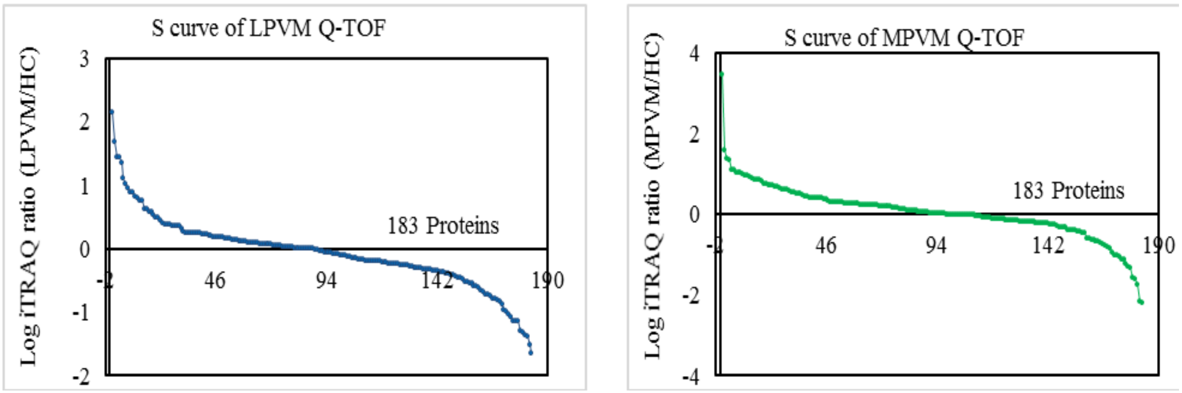

Figure S2B. Distribution of the differentially abundant proteins in low and moderately high parasitemic vivax malaria patients identified in iTRAQ-based quantitative proteomics analysis.

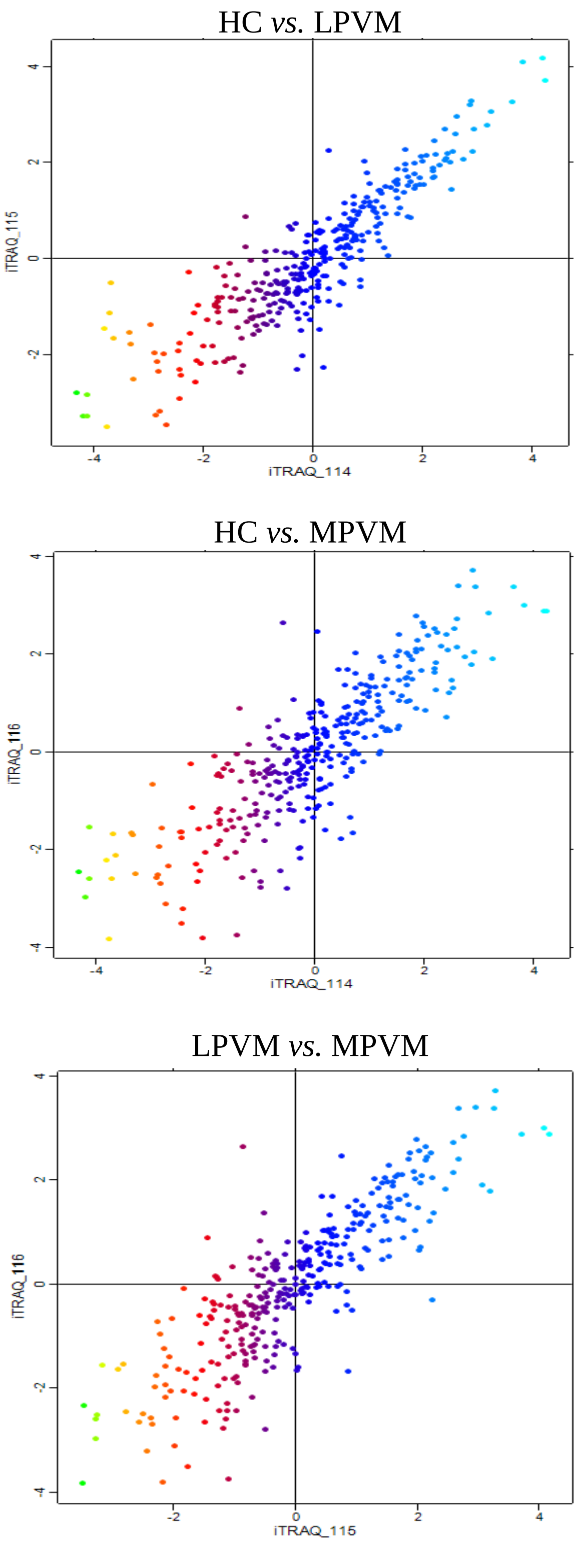

Figure S2C. Scatter plots exhibiting correlations among the serum proteome profiles of HC and different parasitemic vivax malaria patients (LPVM and MPVM).

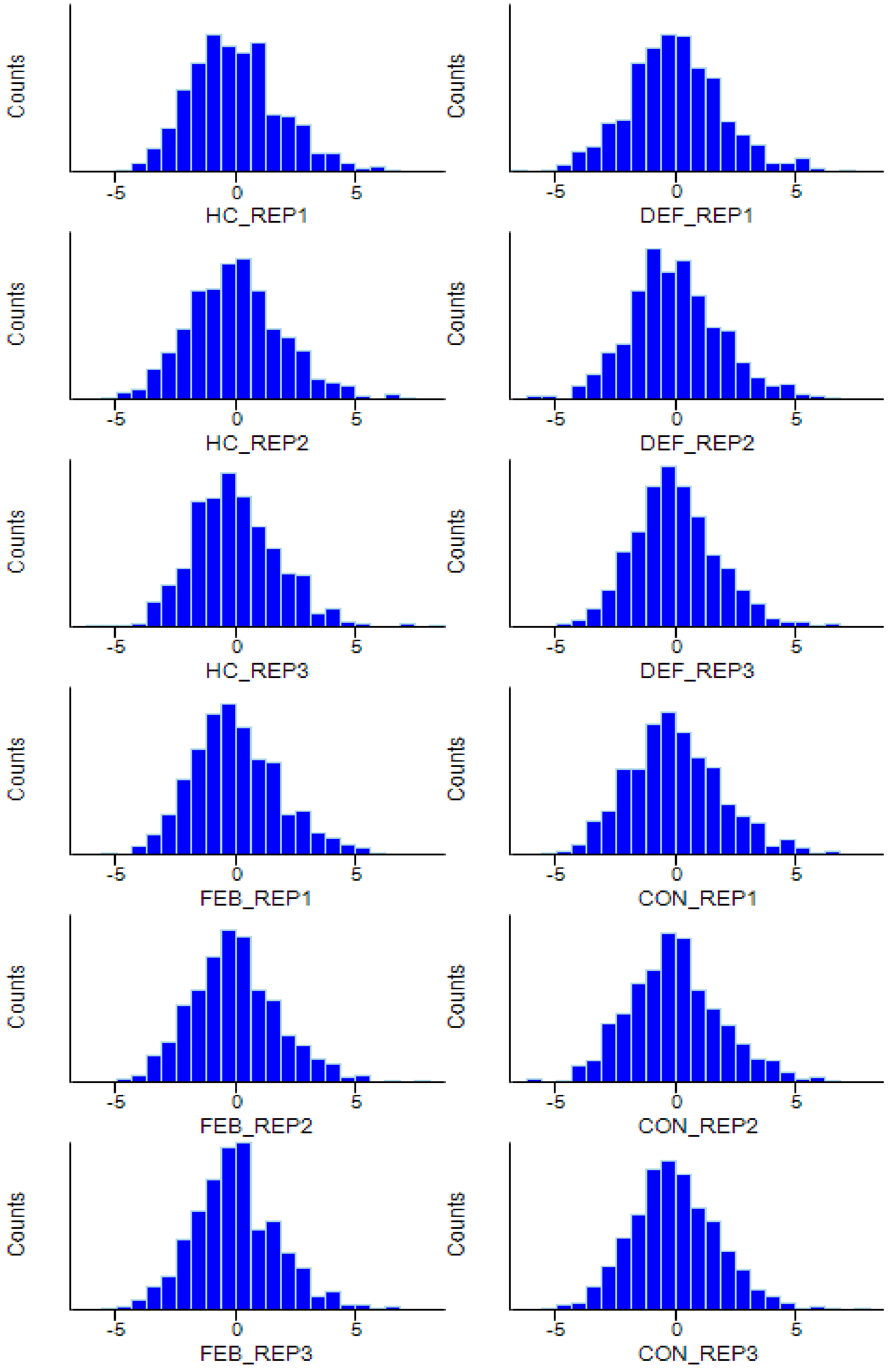

Figure S2D. Histograms showing normal distribution of different iTRAQ data sets (after normalization) for longitudinal cohorts of vivax malaria patients.

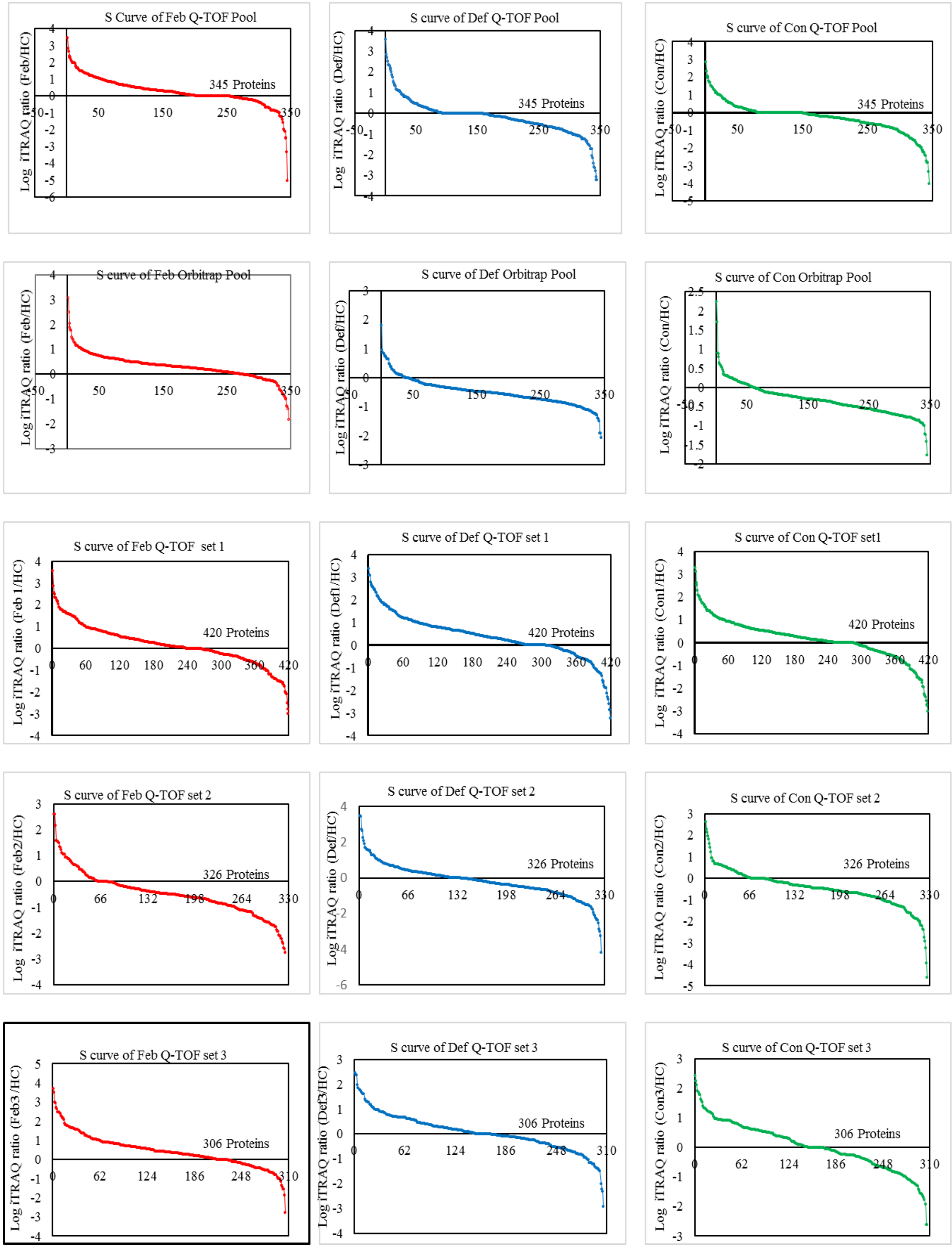

Figure S2E. Distribution of the differentially abundant proteins in a longitudinal cohort of vivax malaria patients identified in iTRAQ-based quantitative proteomics analysis.

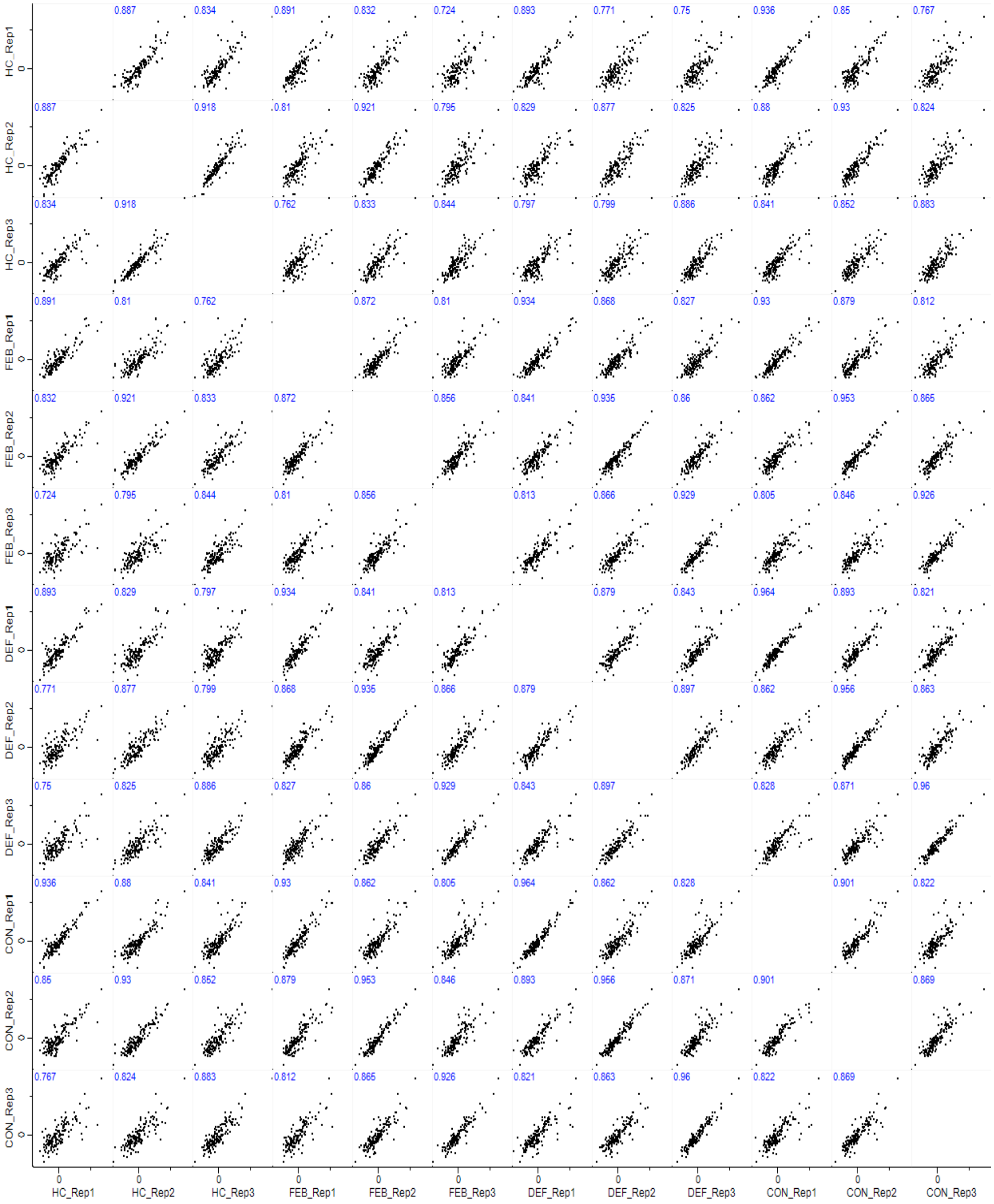

Figure S2F. Scatter plots exhibiting correlations (Pearson correlation coefficient) among the different iTRAQ data sets (analyzed by using Q-TOF) for longitudinal cohorts of vivax malaria patients.

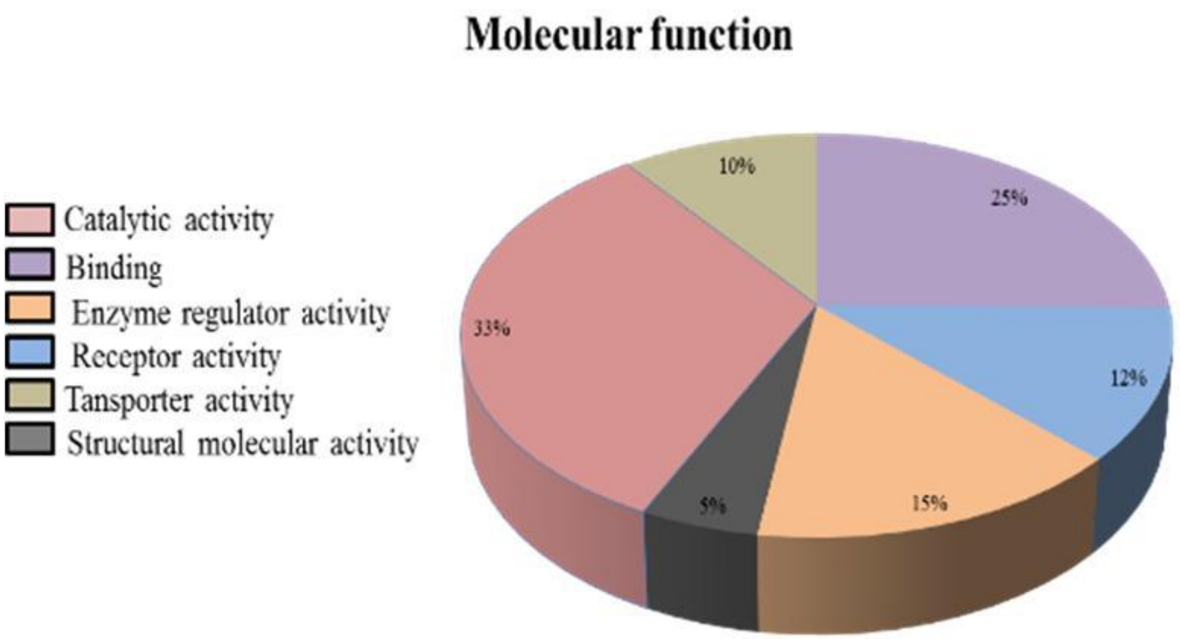
Molecular function
Catalytic activity
Binding
Enzyme regulator activity
Receptor activity
Tansporter activity
Structural molecular activity
10%
25%
33%
12%
5%
15%

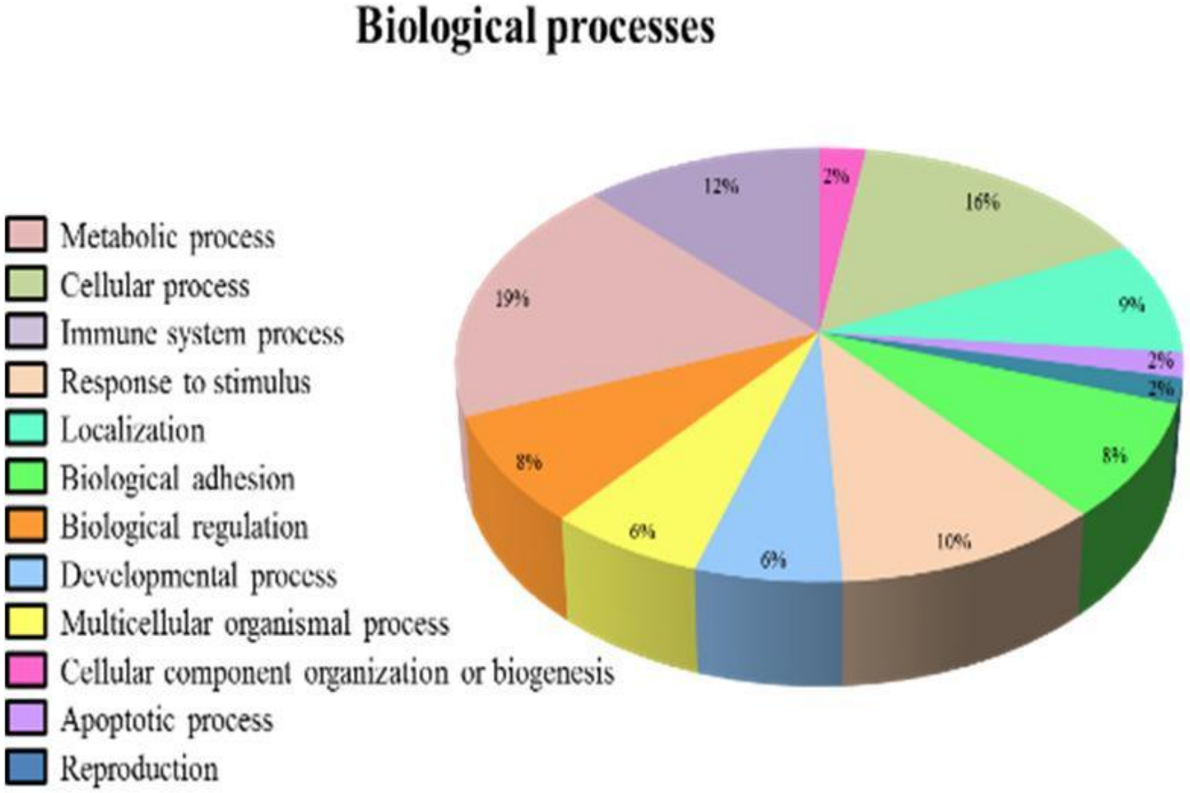
Biological processes
Metabolic process
Cellular process
Immune system process
Response to stimulus
Localization
Biological adhesion
Biological regulation
Developmental process
Multicellular organismal process
Cellular component organization or biogenesis
Apoptotic process
Reproduction
12%
2%
16%
19%
9%
2%
2%
8%
8%
6%
6%
10%

Protein class

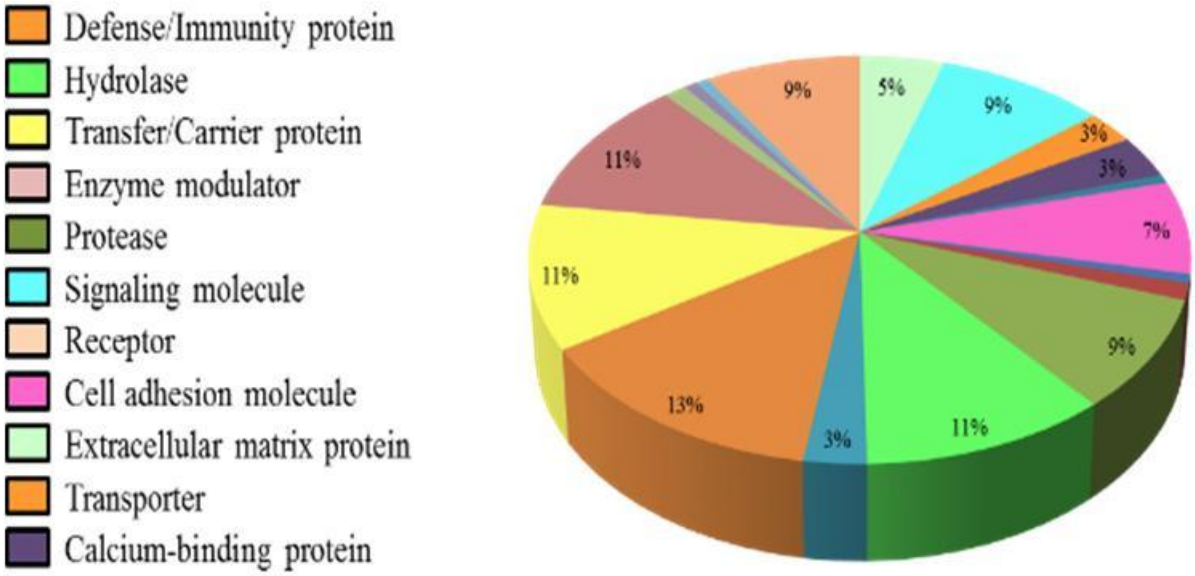

Cellular component

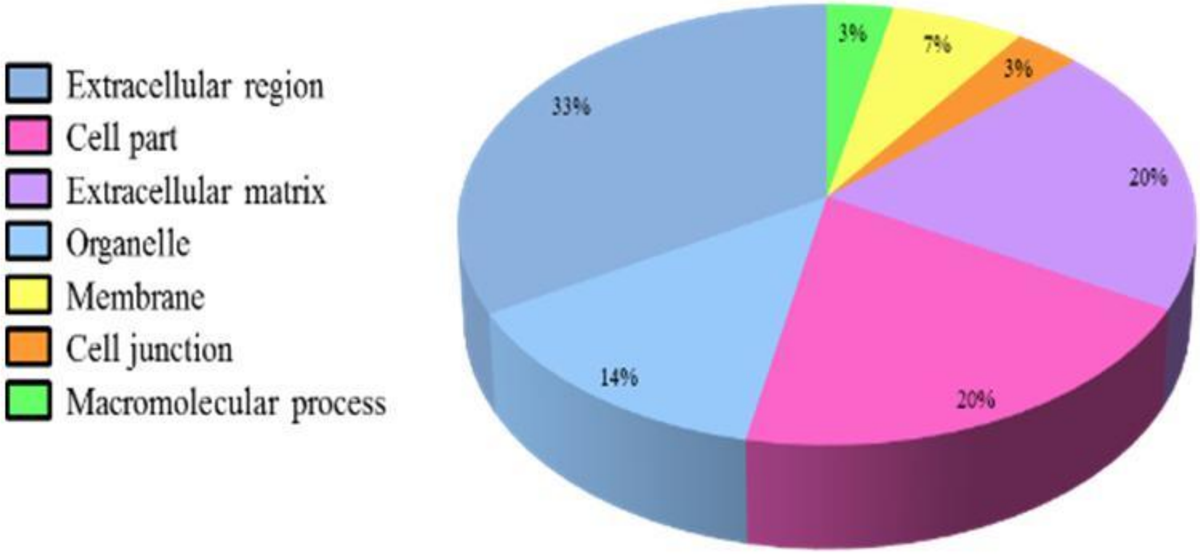

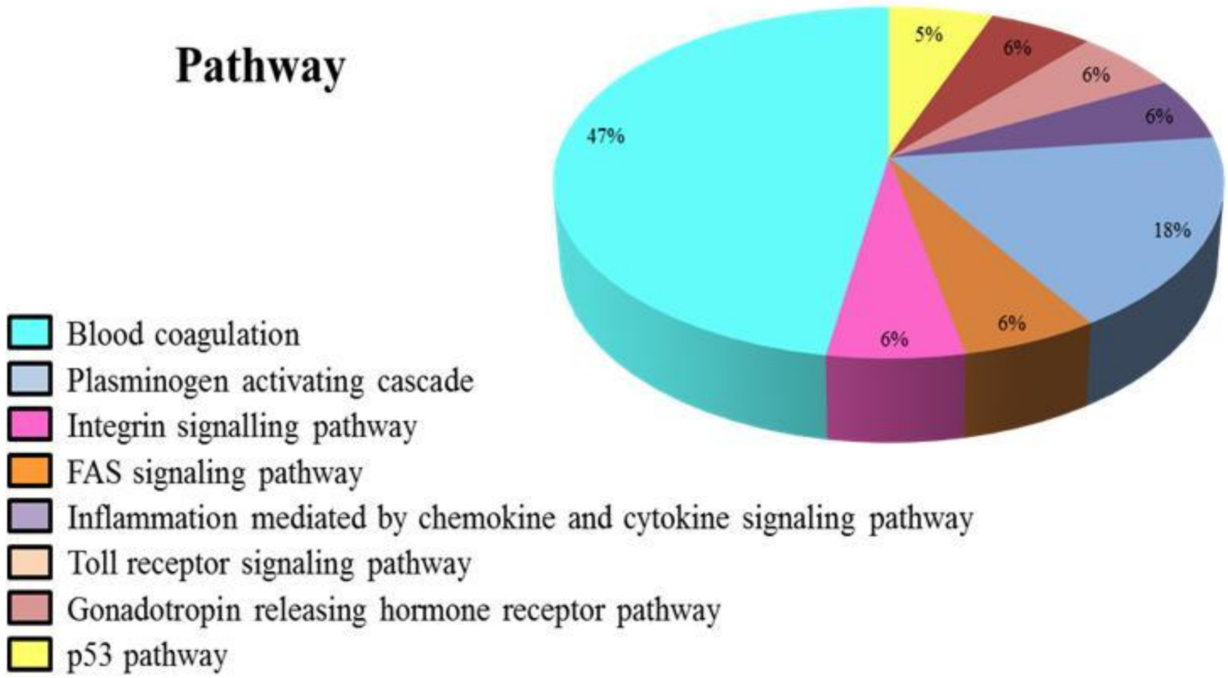

Figure S3A. Functional clustering, biological functions and physiological pathways associated with the differentially abundant proteins identified in low parasitemic vivax malaria patients.

Molecular Functions

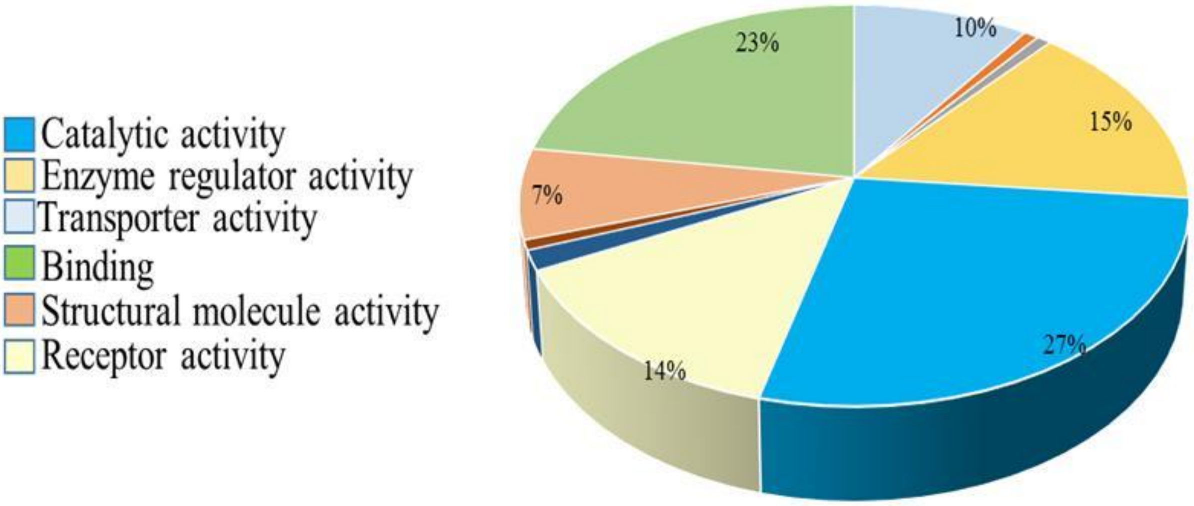

Biological Process

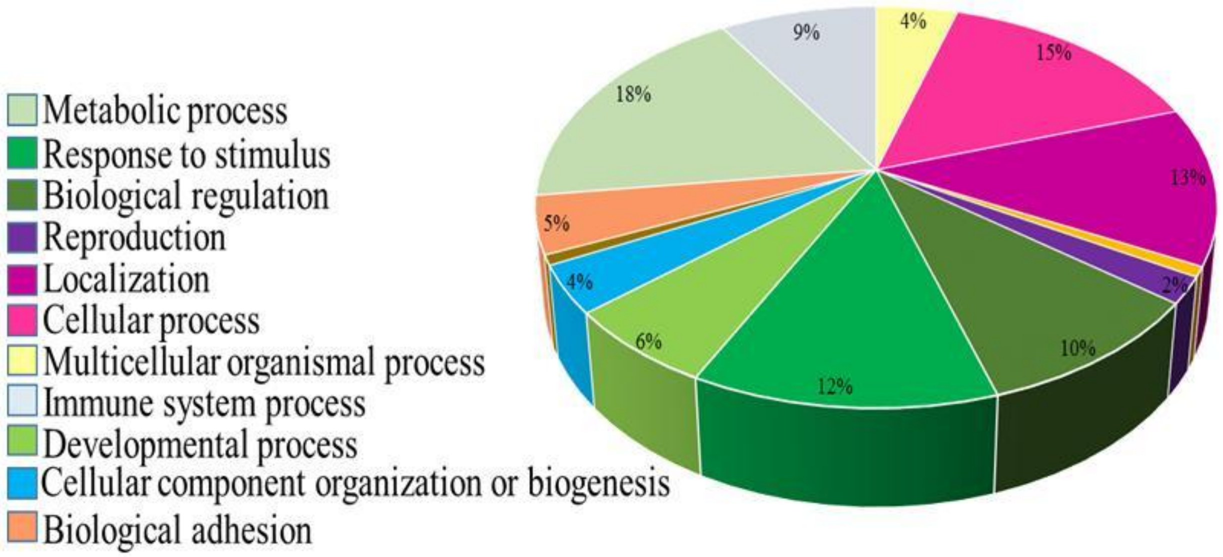

Protein Class

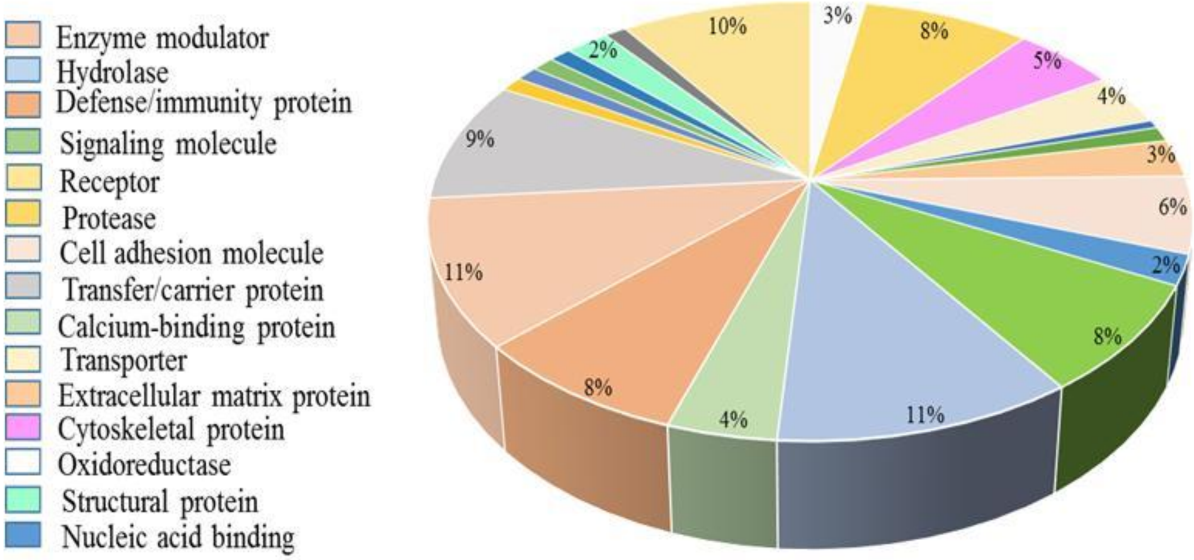

Cellular Components

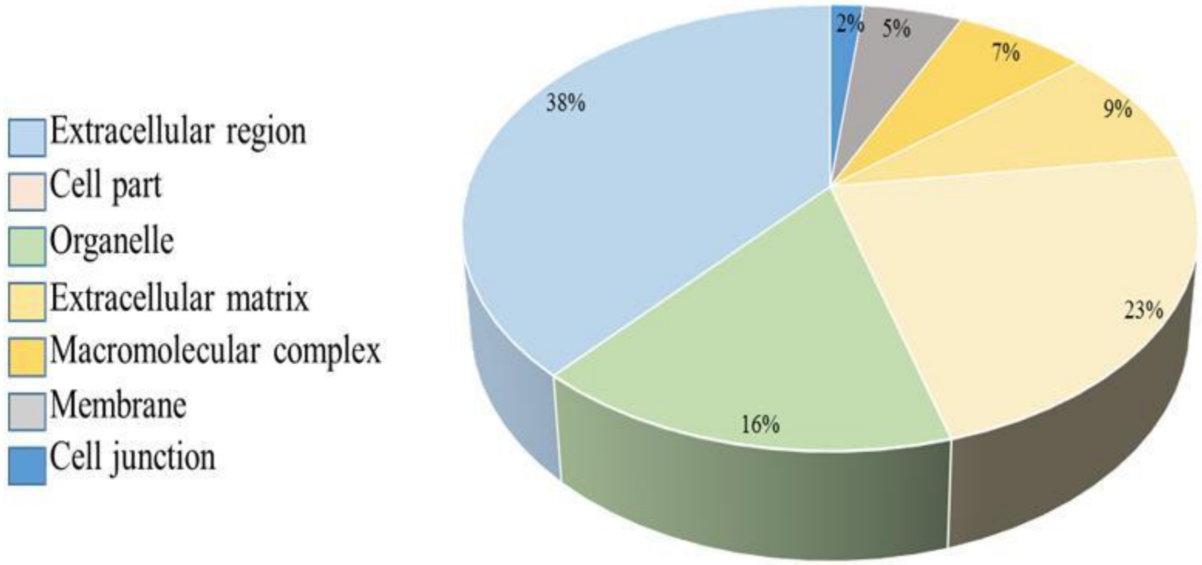

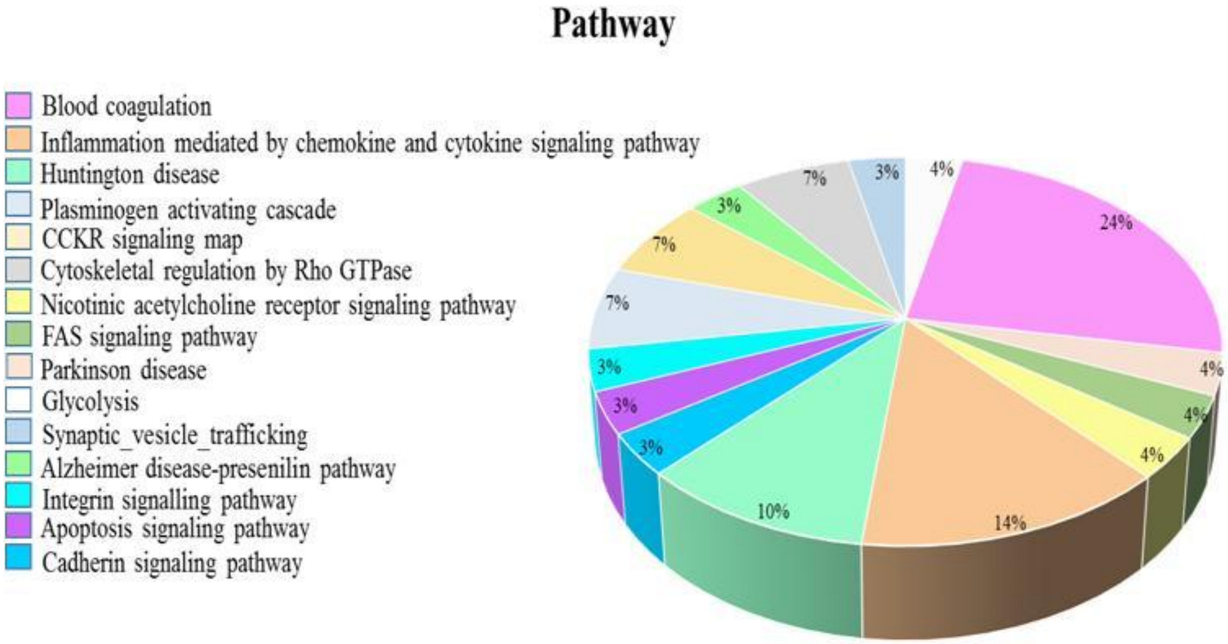

Figure S3B. Functional clustering, biological functions and physiological pathways associated with the differentially abundant proteins identified in moderately high parasitemic vivax malaria patients.

Acute phase response signaling

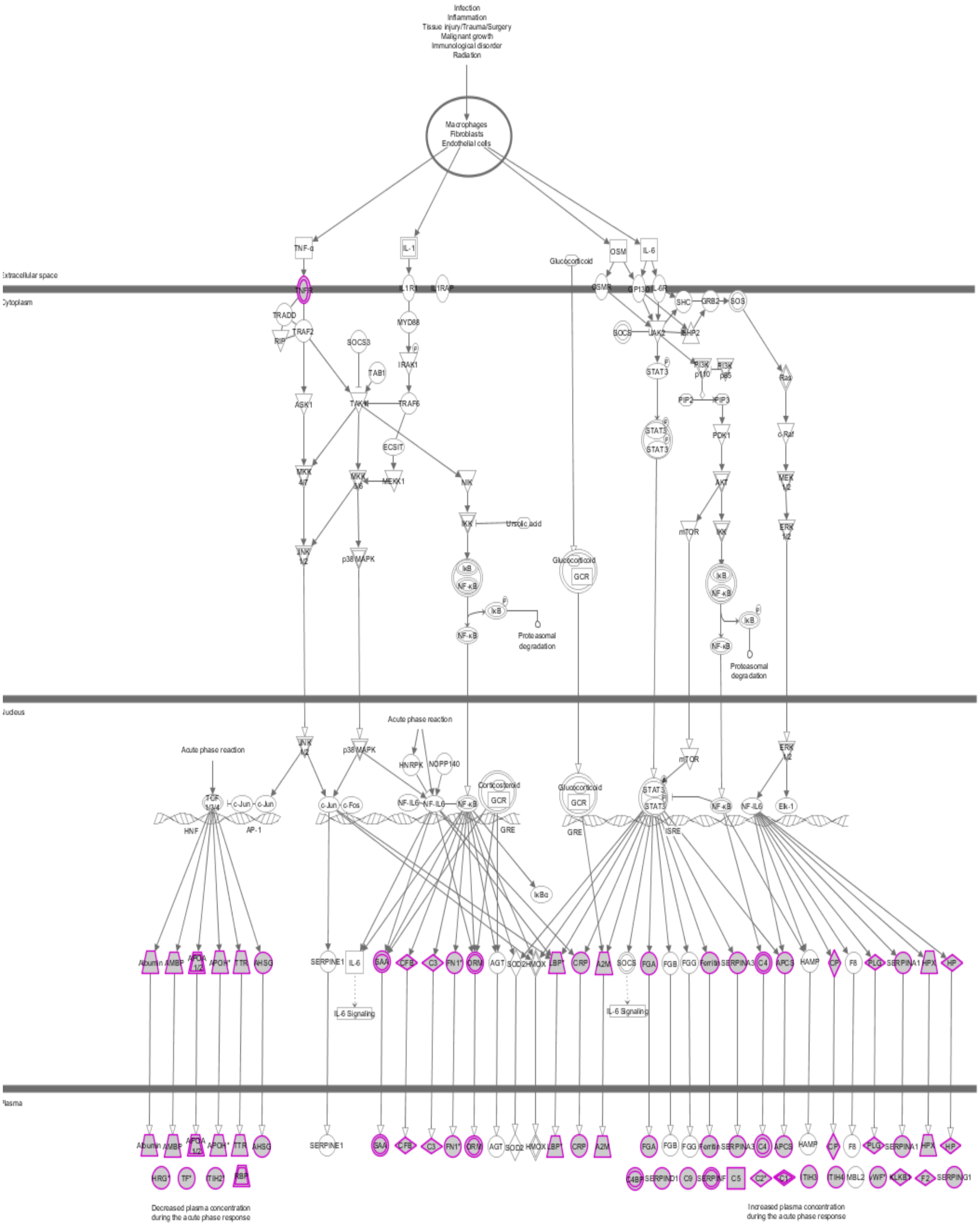

LXR/RXR Activation

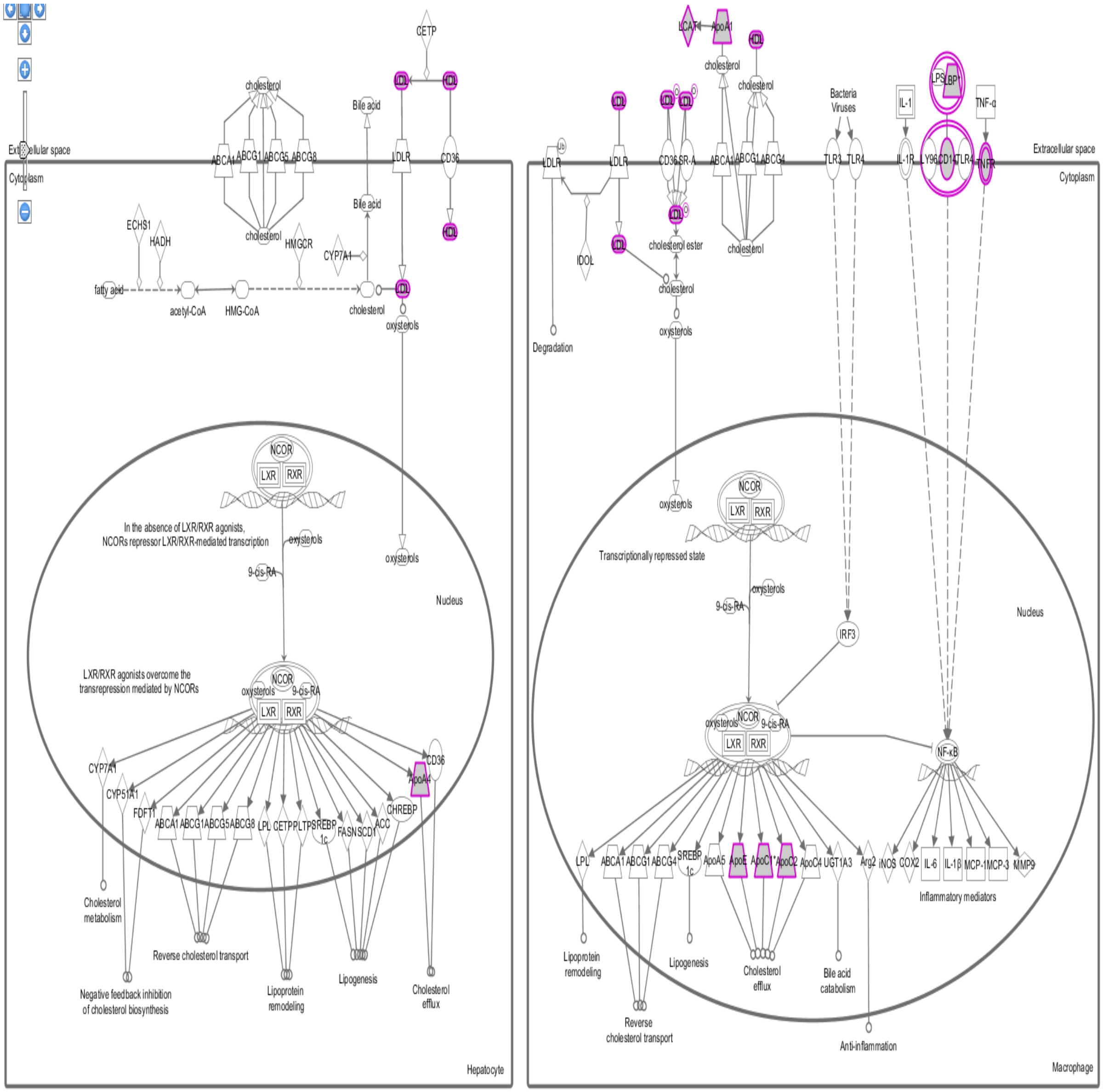

Clathrin-mediated endocytosis signaling

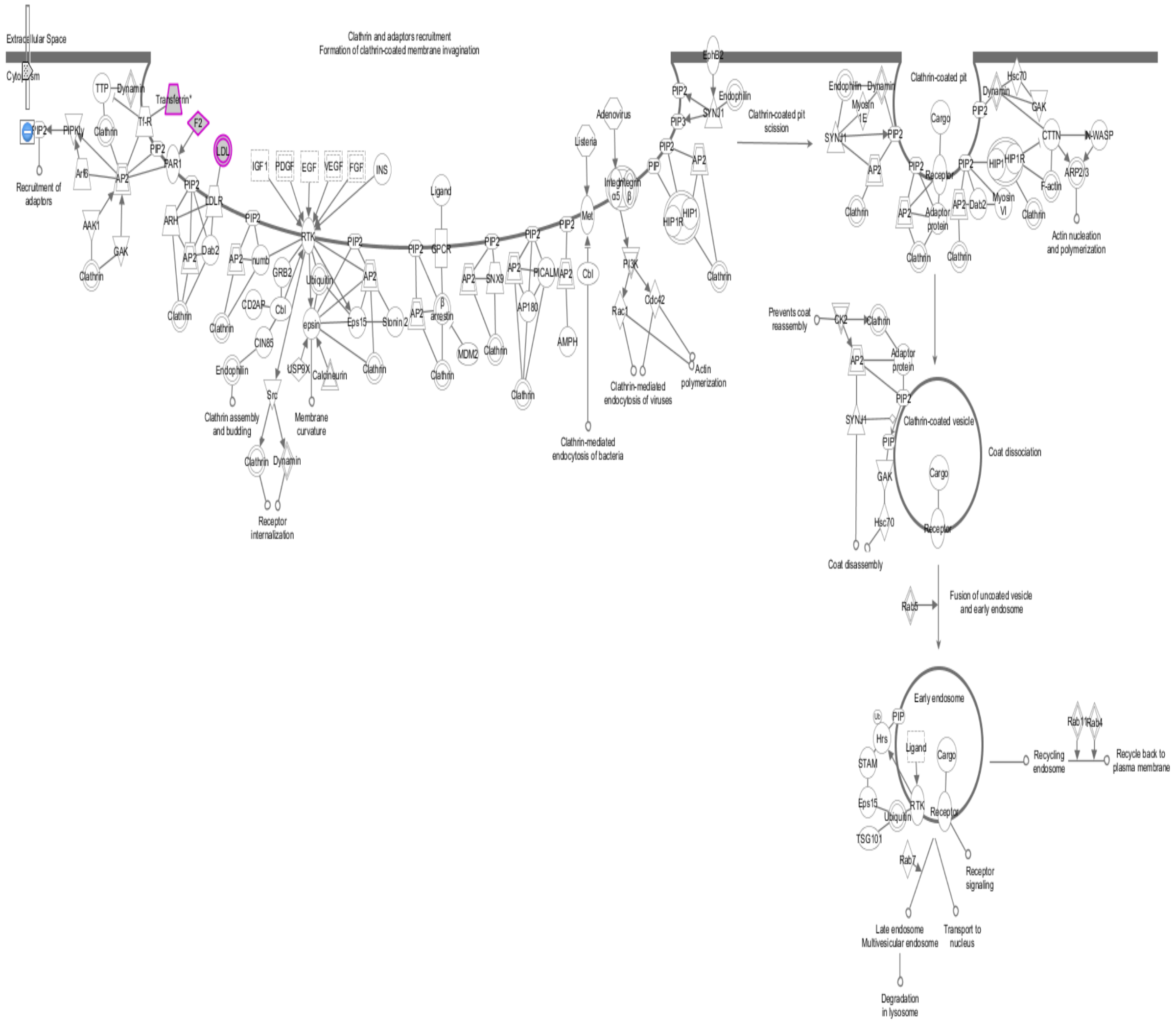

Acute cytoskeleton signaling

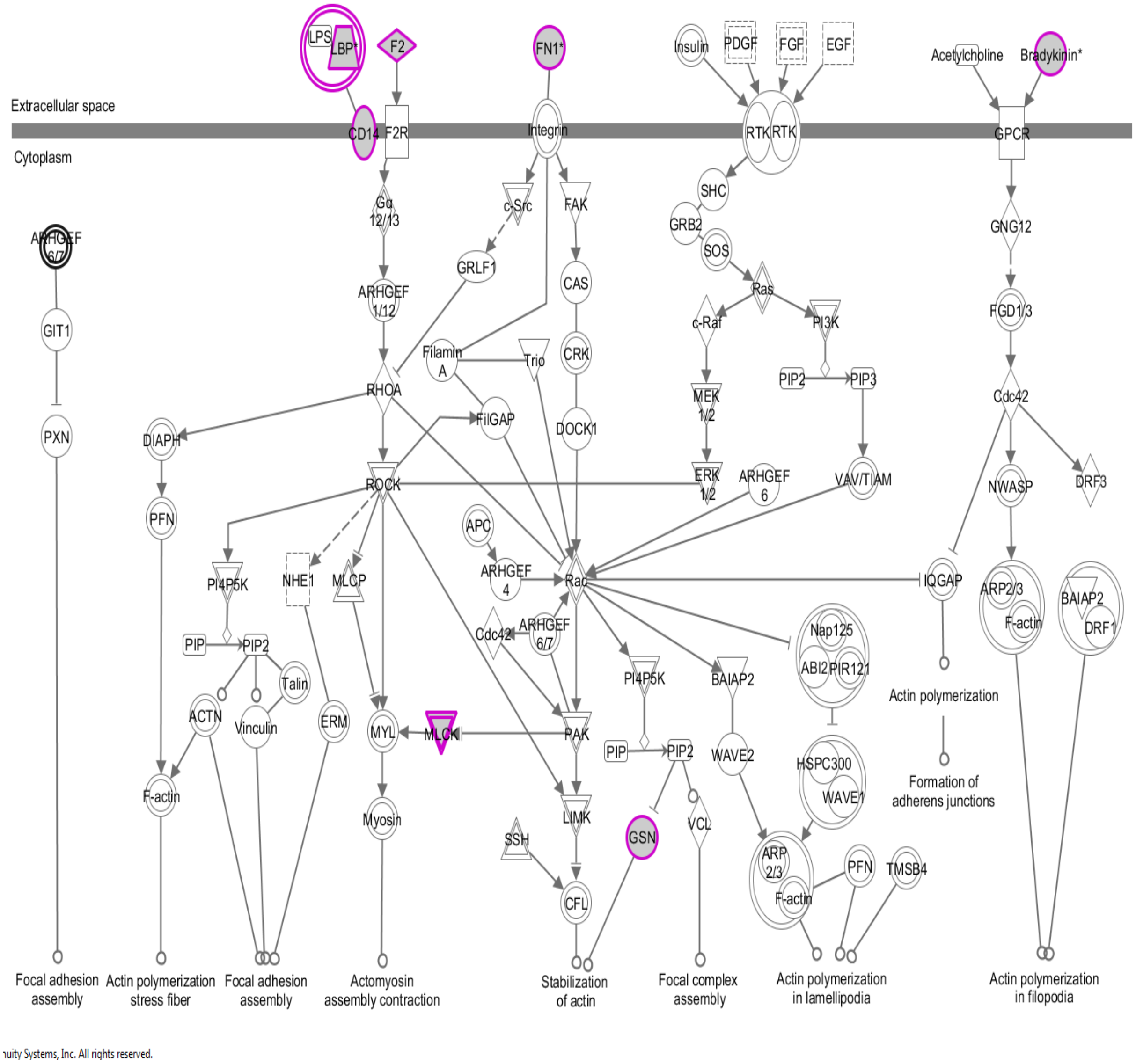

B Cell development

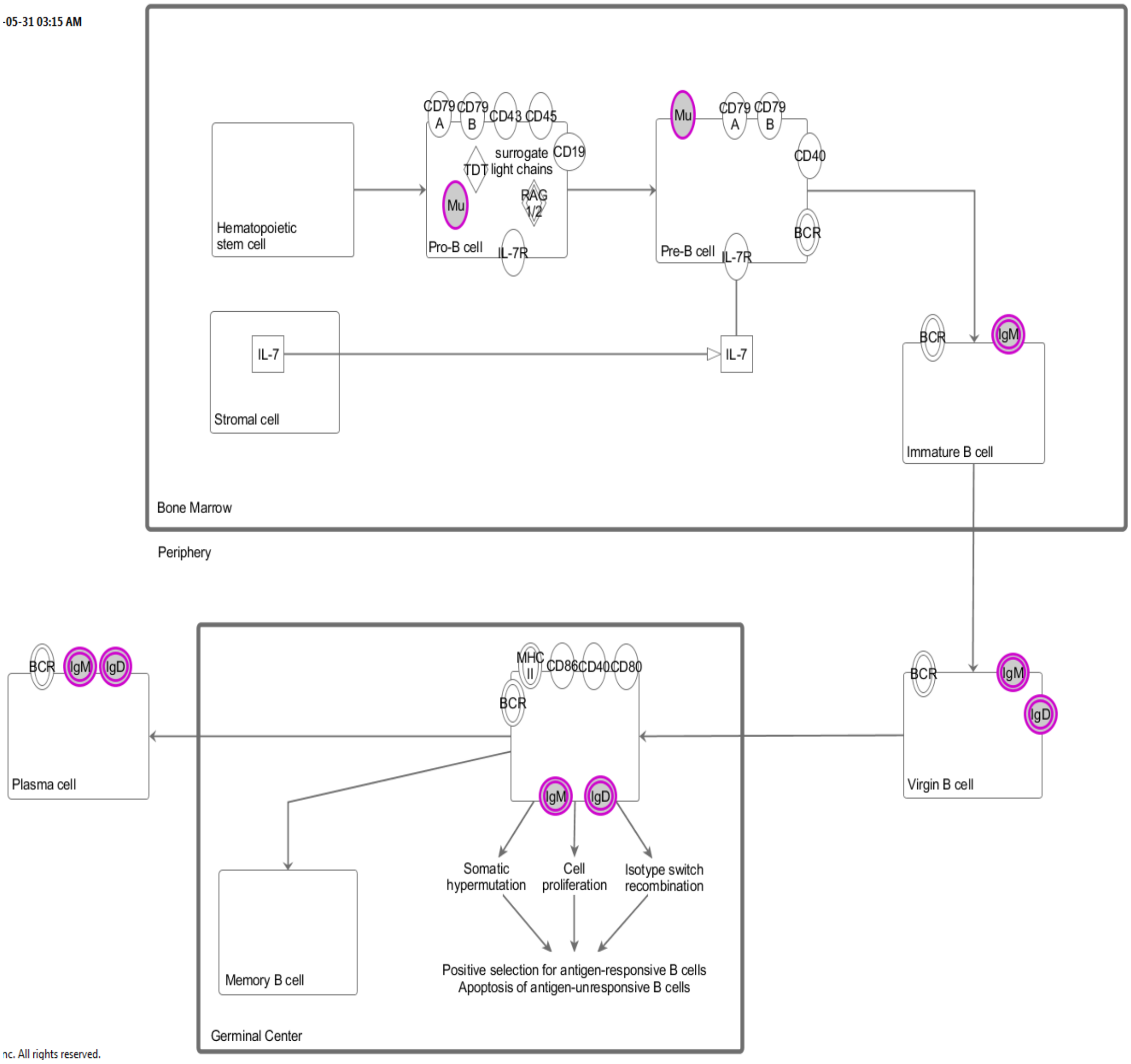

LPS/IL-1 mediated inhibition of RXR function

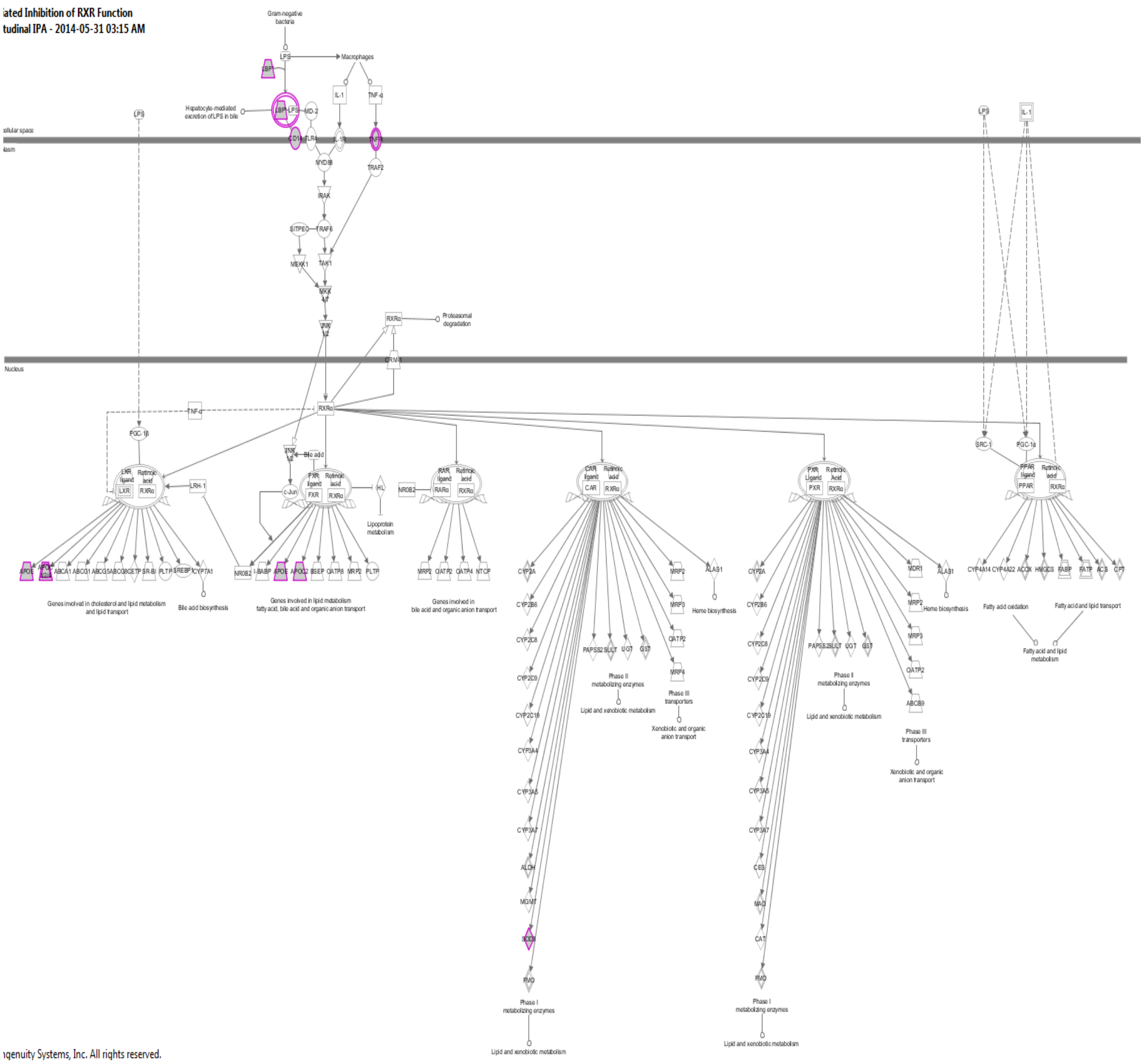

IL-6 signaling

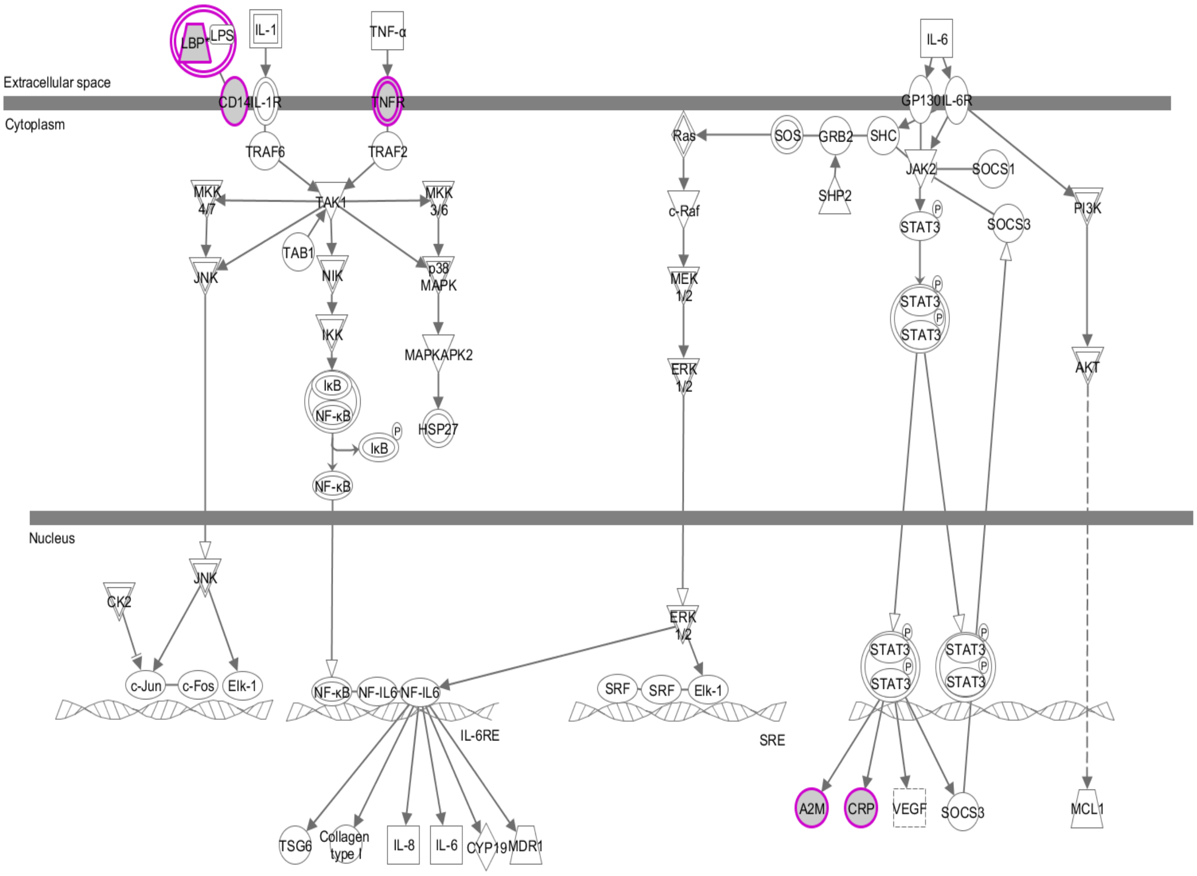

enuity Systems, Inc. All rights reserved.

Neuroprotective role of THOP1 in Alzheimer's disease

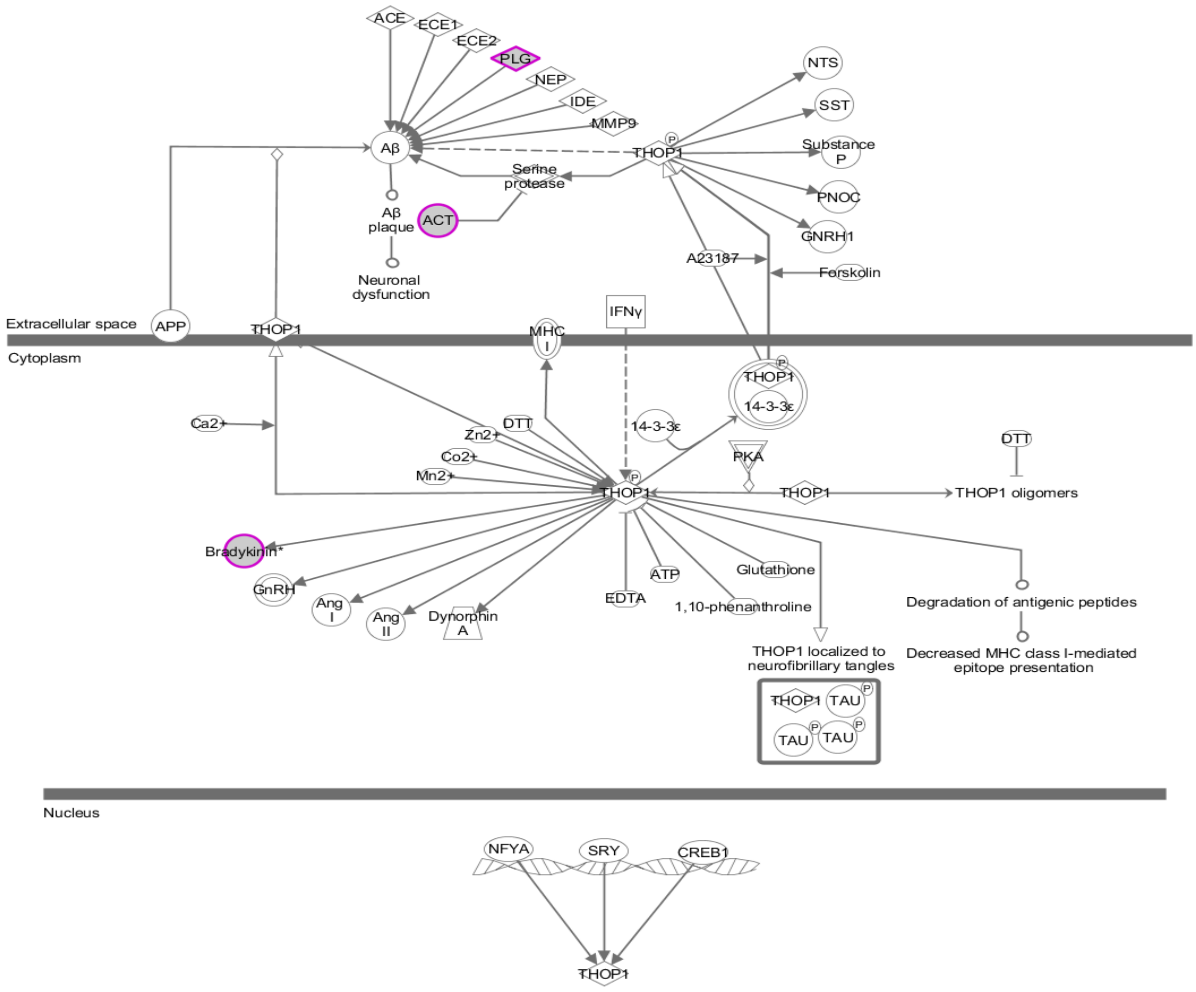

Allograft rejection signaling

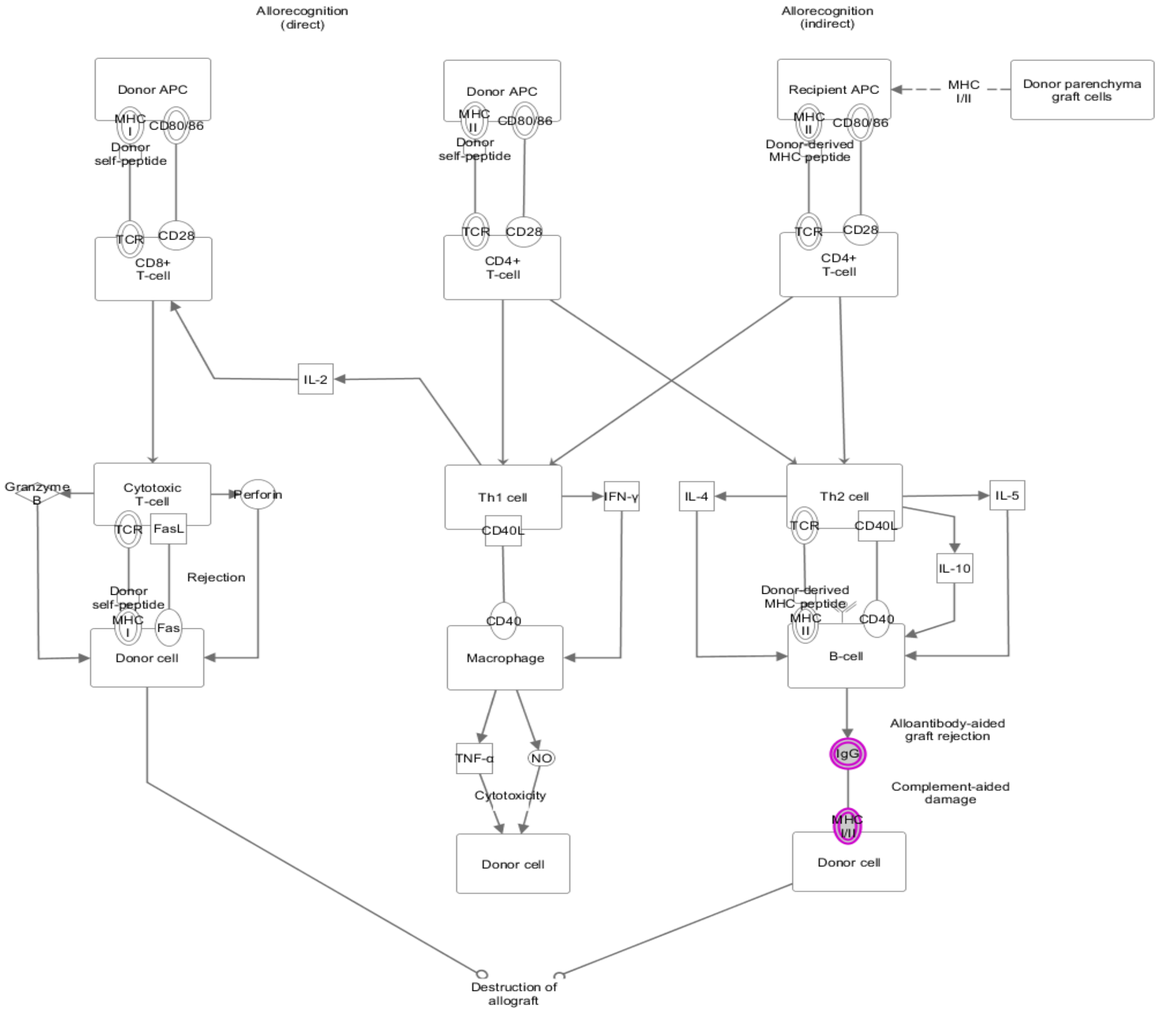

Autoimmune thyroid disease signaling

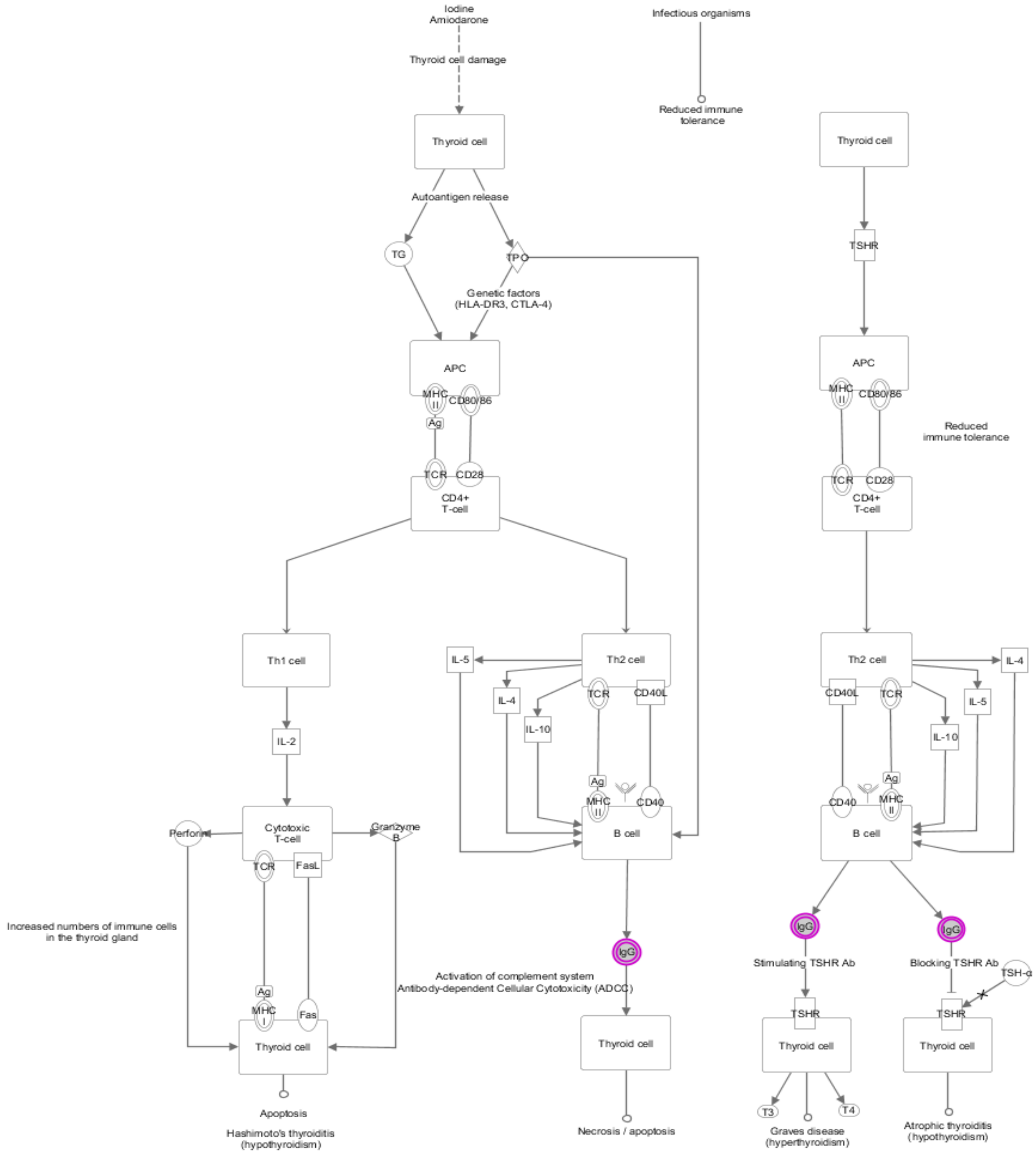

Role of macrophages, fibroblast and endothelial cells in Rheumatoid Arthritis

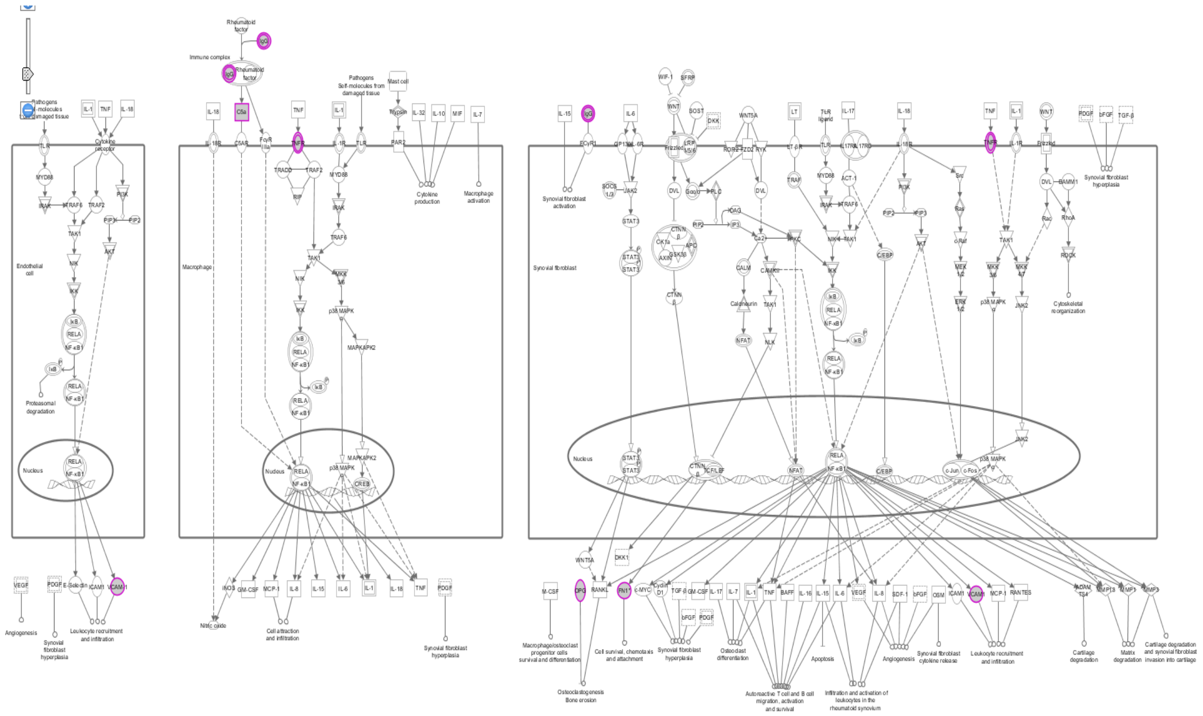

Growth hormone signaling

5 AM

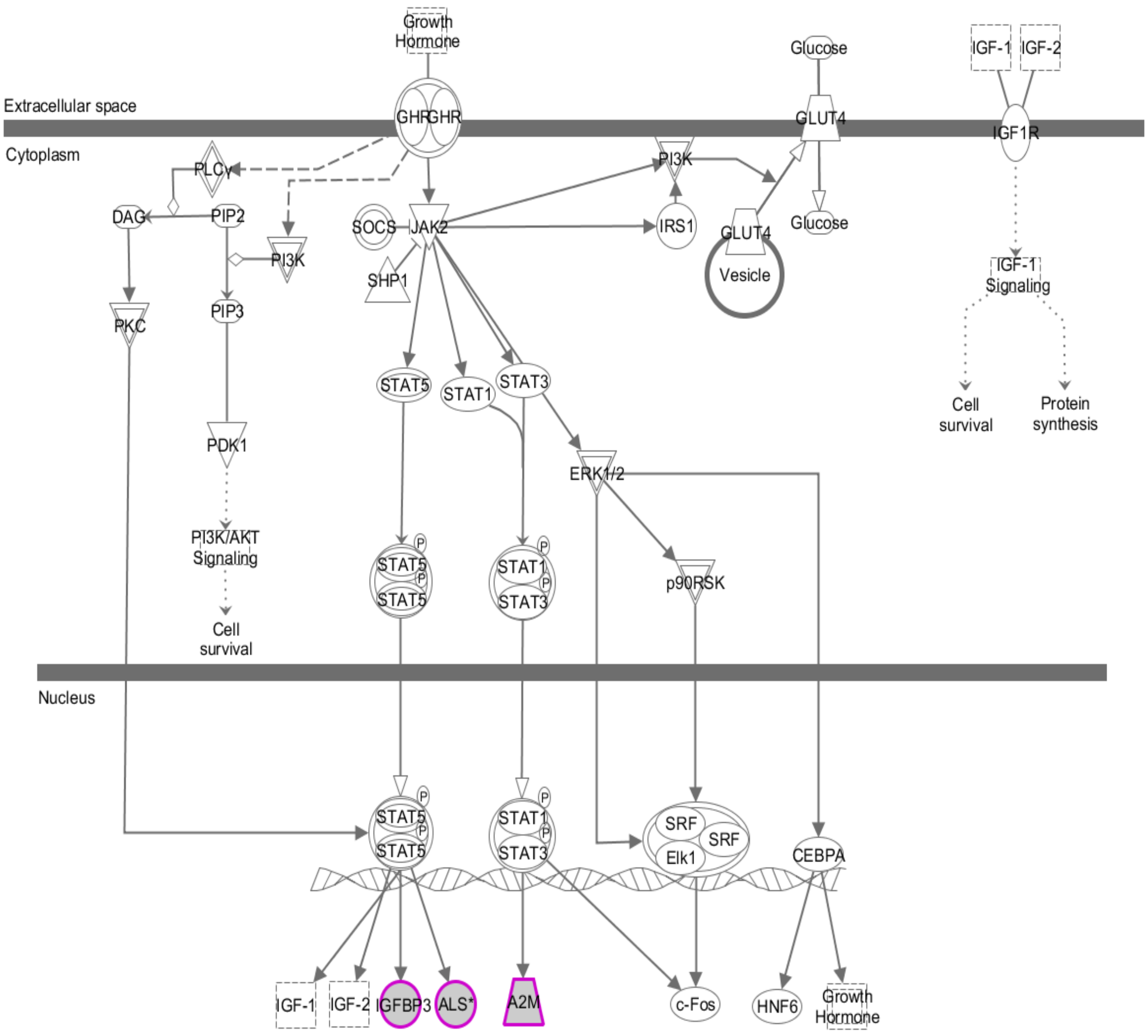

reserved.

Biotin-carboxyl carrier protein assembly

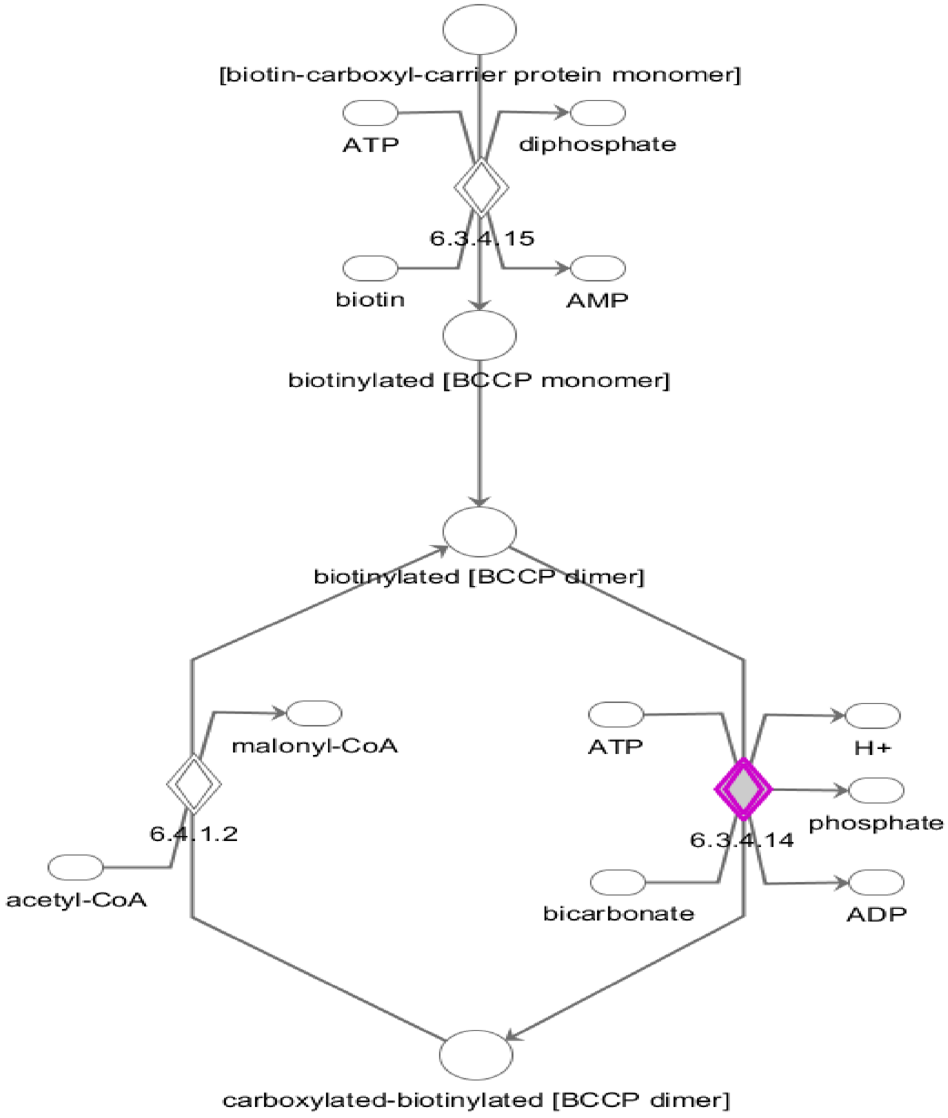

TR/RXR Activation

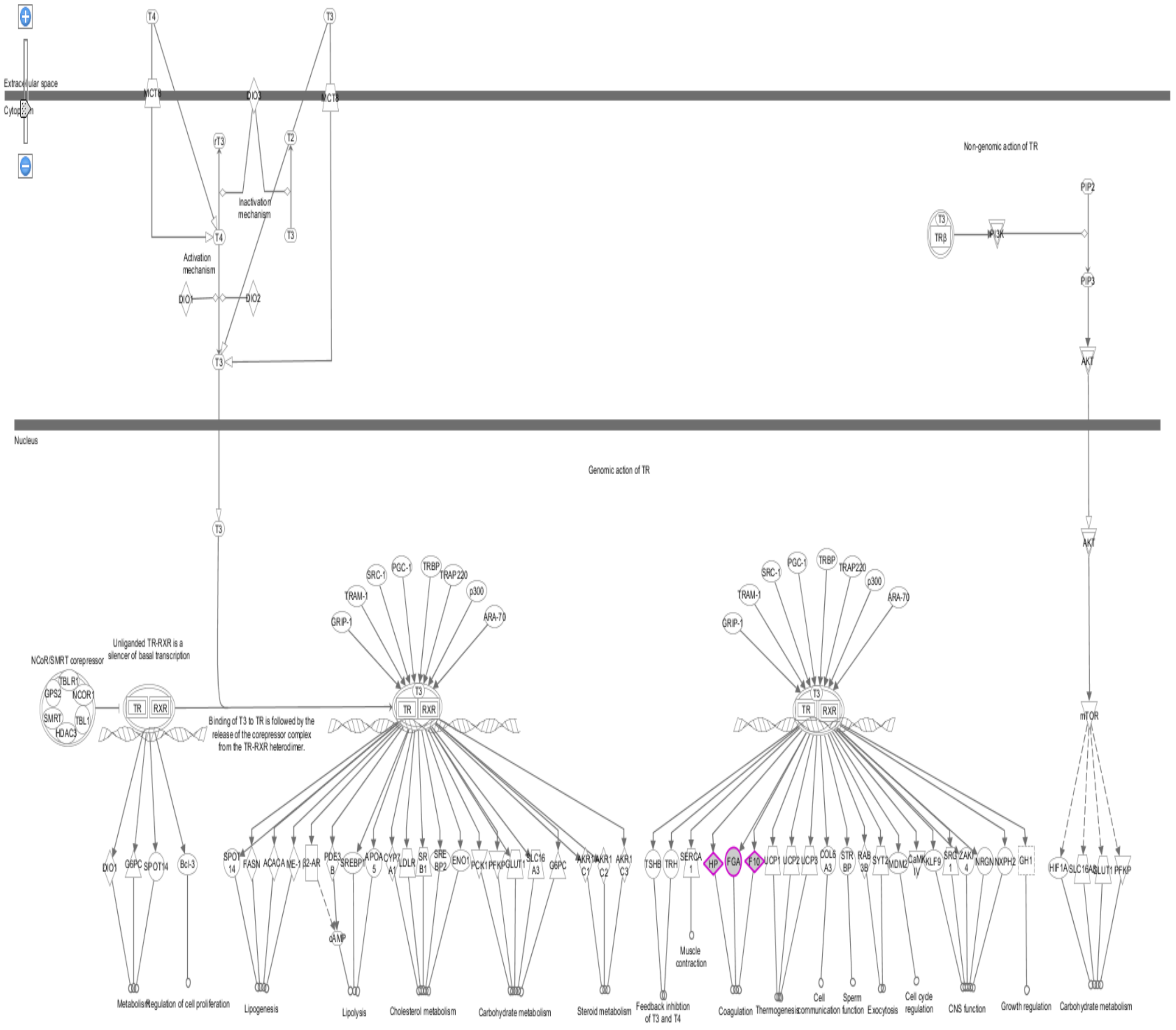

Role of pattern recognition receptors in recognition of bacteria and viruses

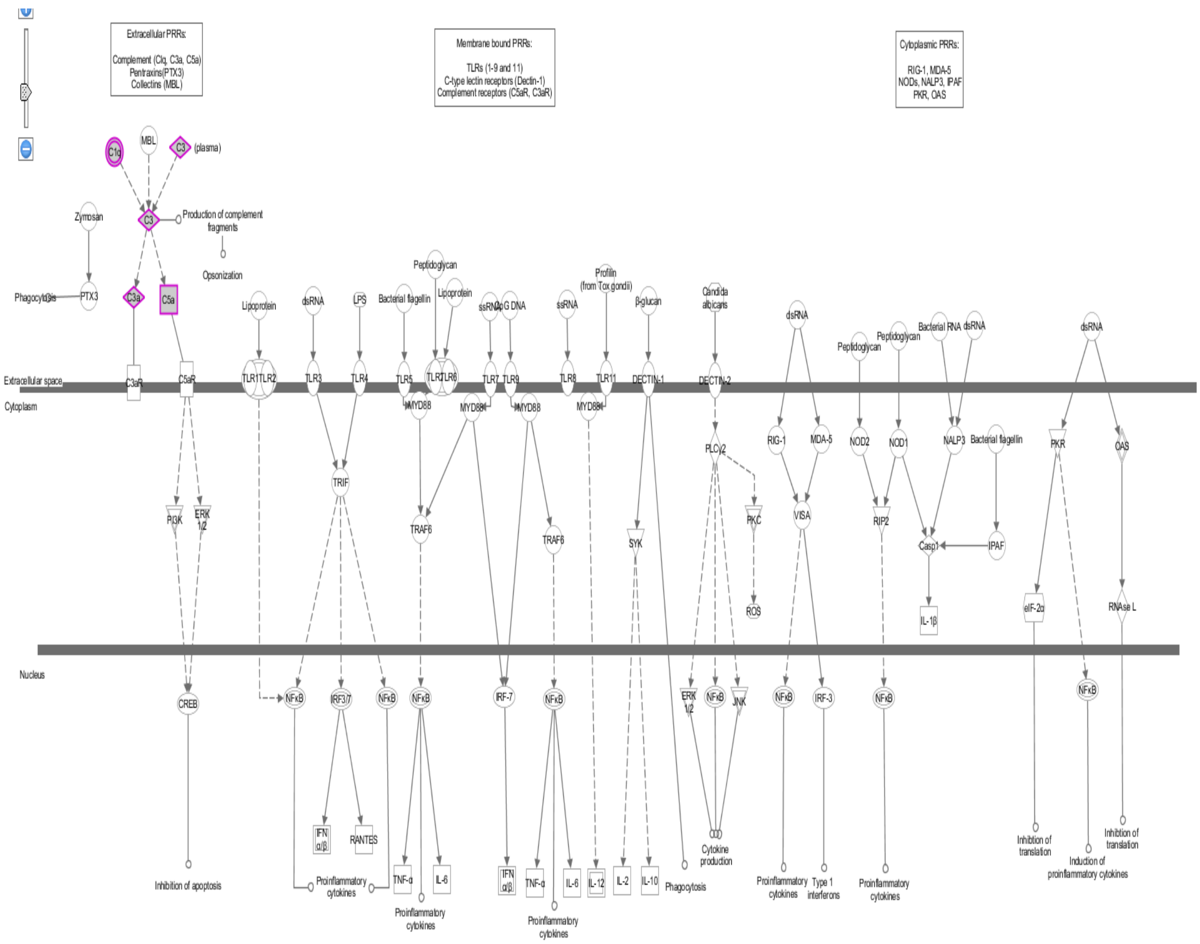

Superoxide radicals degradation

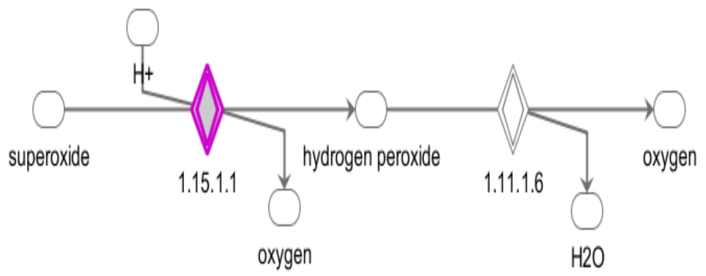

Figure S4. Different physiological pathways associated with the differentially abundant proteins identified the longitudinal cohort of vivax malaria patients. The images [networks, functional analyses, etc.] were generated through the use of QIAGEN's Ingenuity Pathway Analysis (IPA®, QIAGEN Redwood City, www.qiagen.com/ingenuity).

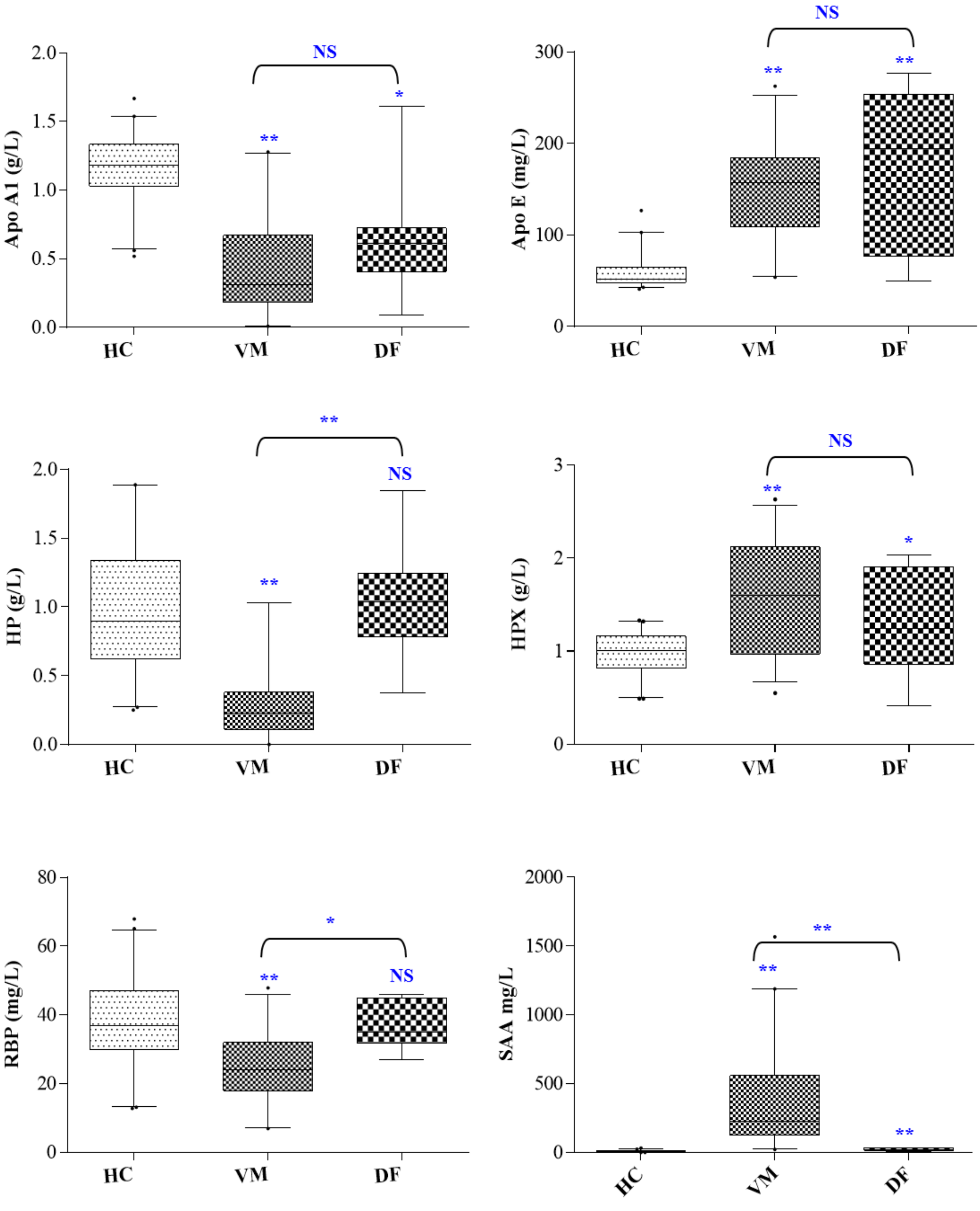

Figure S5. ELISA-based measurement of serum levels of some selected differentially abundant proteins in healthy controls (HC), vivax malaria (VM) and dengue fever (DF) patients. ** Indicates $p < 0.001$, * indicates $0.001 < p < 0.05$ and NS indicates $p > 0.05$ based on a Mann-Whitney test.

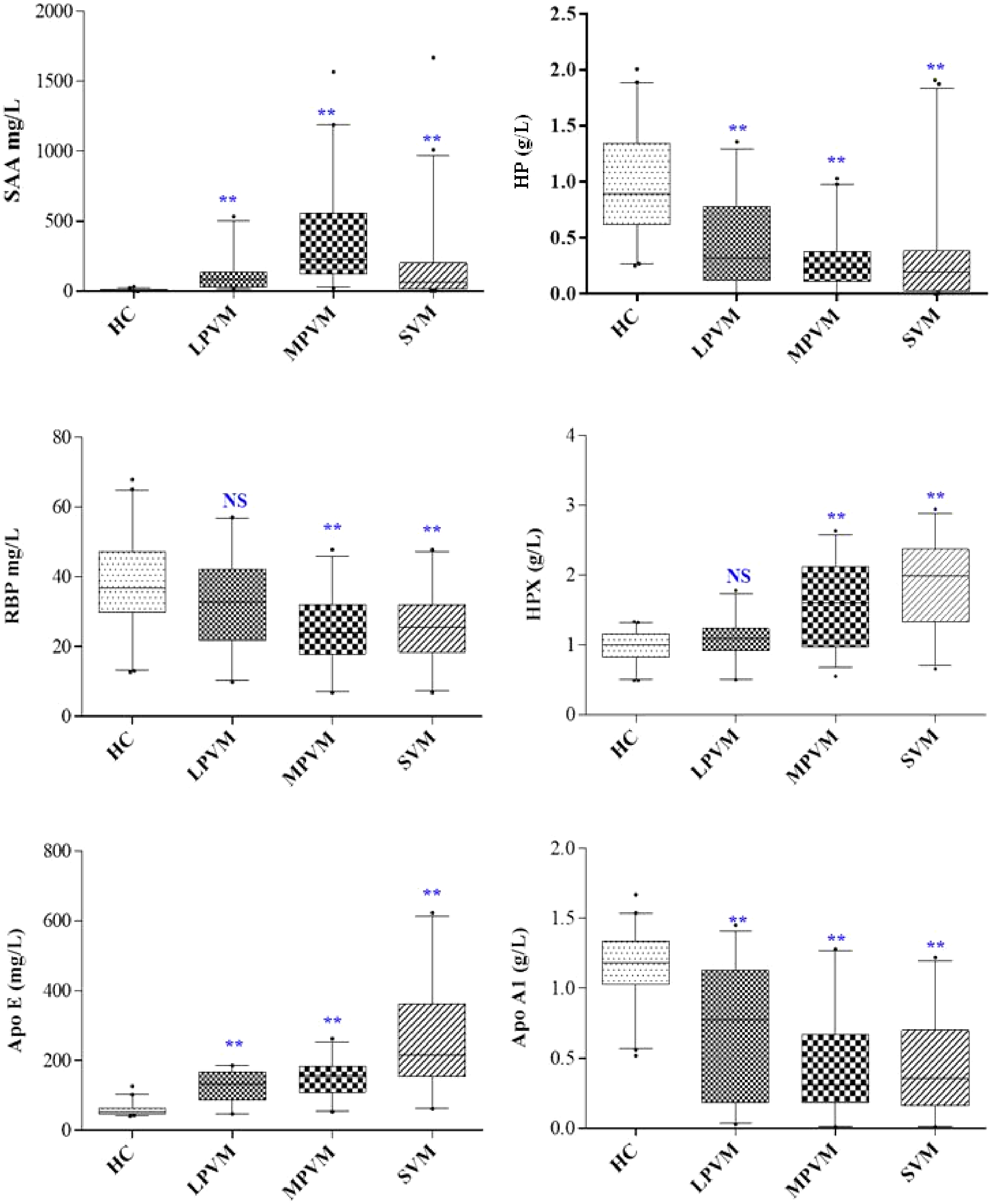

Figure S6. ELISA-based measurement of serum levels of some selected differentially abundant proteins in low and moderately high parasitemic non-severe vivax malaria and severe vivax malaria patients (measurements of these proteins in severe vivax malaria have been re-analyzed from a recently published article from our research group [39]. ** Indicates $p < 0.001$, * indicates $0.001 < p < 0.05$ and NS indicates $p > 0.05$ based on a Mann-Whitney test.

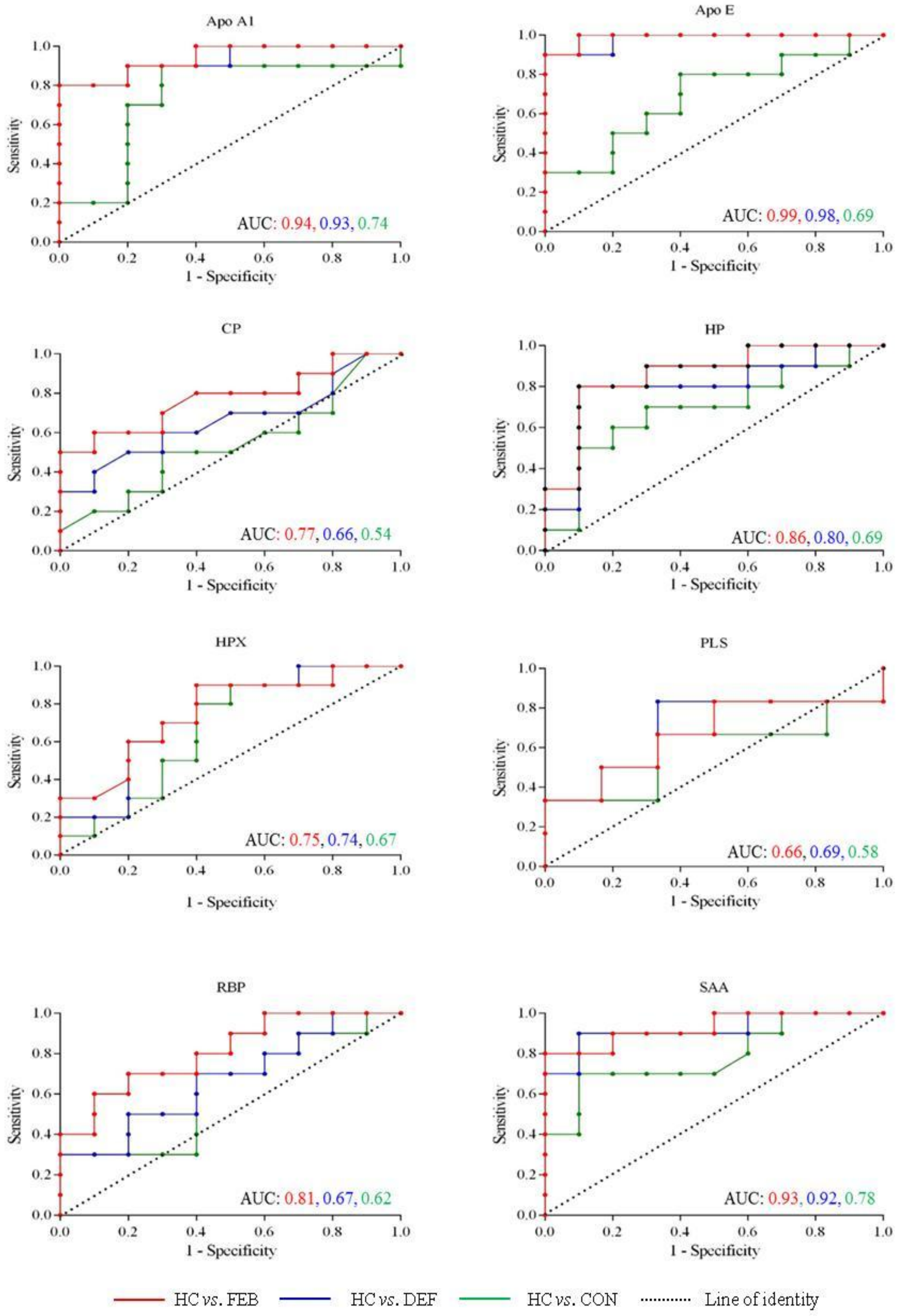

Figure S7. Receiver operating characteristic (ROC) curves depicting the accuracy of a few differentially abundant serum proteins for prediction of FEB, DEF and CON stages of vivax malaria**.**

B. Supplementary Tables

Table S1A. Demographic and clinical details of healthy control subjects and low and moderately high parasitemic vivax malaria and dengue fever patients.

Table S1B. Summary of Kruskal–Wallis test (multiple comparison test) results performed on different clinical parameters in LPVM, MPVM and HC study cohorts.

Table S2A. Demographic and clinical details of the longitudinal cohort of vivax malaria patients and healthy controls.

Table S2B. Summary of Kruskal–Wallis test (multiple comparison test) results performed on different clinical parameters in HC and a longitudinal cohort of vivax malaria patients.

Table S3. Summary for statistically significant ($p < 0.05$) differentially abundant protein spots in low parasitemic vivax malaria patients compared to the healthy control subjects and moderately-high parasitemic vivax malaria patients visualized in 2D-DIGE gels.

Table S4A. Differentially abundant proteins in LPVM compared to HC identified in 2D-DIGE in combination with MALDI-TOF/TOF analysis.

Table S4B. Differentially abundant proteins in MPVM compared to LPVM identified in 2D-DIGE in combination with MALDI-TOF/TOF analysis.

Table S5A. Complete details of protein identification and quantitative iTRAQ data for low and moderately-high parasitemic vivax malaria patients (compared to HC) obtained from Q-TOF analysis.

Table S5B. Differentially abundant proteins in low and moderately-high parasitemic vivax malaria patients (compared to HC) identified by iTRAQ-based quantitative proteomics analysis.

Table S6A. Differentially abundant serum proteins in FEB stage of the infection compared to HC subjects identified in 2D-DIGE combined with MALDI-TOF/TOF.

Table S6B. Differentially abundant serum proteins in DEF stage of the infection compared to HC subjects identified in 2D-DIGE combined with MALDI-TOF/TOF.

Table S6C. Differentially abundant serum proteins in CON stage of the infection compared to HC subjects identified in 2D-DIGE combined with MALDI-TOF/TOF.

Table S7A. Complete details of protein identification and quantitative iTRAQ data for the longitudinal cohort of vivax malaria patients (compared to HC) obtained from Q-TOF analysis.

Table S7B. Complete details of protein identification and quantitative iTRAQ data for the longitudinal cohort of vivax malaria patients (compared to HC) obtained from Q-Exactive analysis.

Table S8A. Differentially abundant proteins in the longitudinal cohort of vivax malaria patients (compared to HC) identified by iTRAQ-based quantitative proteomics analysis using Q-TOF mass spectrometer.

Table S8B. Differentially abundant proteins in the longitudinal cohort of vivax malaria patients (compared to HC) identified by iTRAQ-based quantitative proteomics analysis using Q-Exactive mass spectrometer.

Table S9. Details of the pathways and networks associated with the differentially abundant serum proteins identified in low and moderately-high parasitemic vivax malaria patients.

Table S10. Details of the pathways and networks associated with the differentially abundant serum proteins identified in a longitudinal cohort of vivax malaria patients.

Table S11A. ELISA-based measurement of serum concentrations of a few selected differentially abundant proteins in low and moderately-high parasitemic vivax malaria patients and healthy control subjects.

Table S11B. Summary of Kruskal–Wallis test (multiple comparison test) results performed on serum concentrations of differentially abundant proteins (ELISA-based measurement) in LPVM, MPVM and HC study cohorts.

Table S11C. Probability values (Mann-Whitney test) showing significance of mean differences for differentially abundant proteins in LPVM, MPVM and HC measured by ELISA.

Table S12. Statistical summary of ROC curve analysis for evaluating performance of different serum proteins (measured by ELISA) for prediction of low and moderately-high parasitemic vivax malaria patients.

Table S13A. ELISA-based measurement of serum concentrations of a few selected differentially abundant proteins in a longitudinal cohort of vivax malaria patients.

Table S13B. Summary of Kruskal–Wallis test (multiple comparison test) results performed on serum concentrations of differentially abundant proteins (ELISA-based measurement) in HC and a longitudinal cohort of vivax malaria patients.

Table S13C. Probability values (Mann-Whitney test) showing significance of mean differences for differentially abundant proteins in a longitudinal cohort of vivax malaria patients measured by ELISA.

Table S14. Statistical summary of ROC curve analysis for evaluating performance of different serum proteins for prediction of different stages of disease progression (FEB, DEF and CON stages).

Table S1A. Demographic and clinical details of healthy control subjects and low and moderately high parasitemic vivax malaria and dengue fever patients

Parameters #	Low parasitemic vivax patients (LPVM; n = 23)	Moderate parasitemic vivax patients (MPVM; n = 40)	Dengue fever patients (DF; n = 7)	Healthy controls (HC; n = 27)
Age (Year)	29 (19-58)	33 (21-58)	33 (17-62)	20 (18-27)
Parasitemia (No./µL of blood)	160 (80-200)	2560 (2048-9028)	NA	NA
Hematological parameters				
Hb (g/dL)	12.8 (10.7-57.5)	11.8 (8.6-14.8)	9.8 (402-12.8)	13.1 (11.2-14.8)
ESR (mm/hr)	18 (3-55)	33.5 (5-110)	35.8 (18-105)	7 (0.3-42)
RBC/µL (Millions)	4.55 (3.94-6.9)	4.41 (3.32-5.96)	NA	4.95 (3.93-6.5)
Platelets/µL (Thousands)	185 (46-360)	134 (31-400)	50 (35-150)	348 (240-586)
Neutrophils (%)	72 (57-90)	64.5 (40-78)	NA	66 (48-77)
Lymphocytes (%)	26 (7-39)	31 (19-58)	NA	29 (17-49)
Eosinphils (%)	1 (1)	1.5 (0-6)	NA	3 (1-6)
Basophils (%)	0 (0)	0 (0-2)	NA	0 (0-2)
Monocytes (%)	2 (1-4)	2 (1-8)	NA	1 (0-5)
Biochemical parameters				
Total bilirubin (mg%)	0.69 (0.25-2.73)	0.96 (0.18-4.79)	1.5 (0.6-5.1)	0.62 (0.25-2.08)
AST (IU/L)	39.9 (24.4-90)	32.34 (9.6-55.1)	68 (17-113)	21.22 (12.38-35.36)
ALT (IU/L)	26.9 (10.2-179.6)	31.82 (14.4-106.1)	89 (22-250)	21.22 (12.38-35.36)
ALP (IU/L)	95.3 (63.8-151.5)	72.405 (42.33-131.6)	128 (105-308)	73.25 (17.21-108)

Data is represented as median (interquartile-range); NA- Not available

Table S1B. Summary of Kruskal–Wallis test (multiple comparison test) results performed on different clinical parameters in LPVM, MPVM and HC study cohorts

**Protein**	**One-way ANOVA**			**Multiple comparison test (Adjusted *P* value)**		
	***P* value**	**Number of groups**	**Kruskal-Wallis statistic**	**LPVM vs. Healthy**	**MPVM vs. Healthy**	**MPVM vs. LPVM**
Hemoglobin	< 0.0001	3	27.73	0.5451	< 0.0001	0.0058
Platelets	< 0.0001	3	61.61	< 0.0001	< 0.0001	0.2964
AST	< 0.0001	3	35.25	< 0.0001	< 0.0001	0.4021
ALT	0.0031	3	11.52	0.135	0.0026	> 0.9999
AP	0.0072	3	9.88	0.0077	> 0.9999	0.0285
Total Bilirubin	0.0011	3	13.7	0.2534	0.0007	0.4435

Table S2A. Demographic and clinical details of the longitudinal cohort of vivax malaria patients and healthy controls

Parameters #	Healthy controls	Febrile stage	Defervescence stage	Convalescence stage
Age (Year)	30 (56-77)	20 (32-65)	20 (32-65)	20 (32-65)
Hematological parameters				
Hb (g/dL)	11.7 (9.3-14.9)	12 (8.8-15.6)	11.8 (8.9-15)	12.6 (10-15.2)
ESR (mm/hr)	16 (8-27)	18 (12-26)	20 (12-26)	16 (12-18)
WBC	7600 (6000-9600)	5700 (4300-9200)	5600 (4200-8700)	5800 (4700-7800)
Neutrophils (%)	64 (60-69)	61 (48-75)	61 (50-72)	58 (54-66)
Monocytes (%)	2 (1-3)	5 (0-7)	5 (2-6)	5 (2-7)
Eosinphils (%)	2 (2-9)	3 (2-5)	3 (2-6)	3 (2-6)
Lymphocytes (%)	31 (26-35)	31 (18-44)	32 (22-42)	34 (24-40)
Biochemical parameters				
Urea	25 (9-38)	25 (16-59)	22 (17-65)	18 (16-43)
Creatinine	1 (0.4-1.4)	0.9 (0.7-1.8)	0.9 (0.6-1.9)	0.9 (0.6-1.2)
Uric acid	5.9 (2.2-7.3)	4.8 (3.7-6.2)	4.7 (3.9-6)	4.3 (3.8-5.2)
AST (IU/L)	20 (11-56)	46 (20-224)	45 (18-216)	32 (17-136)
ALT (IU/L)	21 (12-53)	31 (18-291)	29 (15-237)	22 (16-89)
Total protein	6.9 (5.9-9)	5.8 (4.9-6.7)	5.6 (5-6.6)	5.7 (5.1-6.8)
Albumin	4 (3.7-4.9)	3.7 (3.2-4.1)	3.6 (3.2-4.1)	3.6 (2.9-4.2)
Globulin	2.8 (2-4.1)	2 (1.4-3.7)	1.9 (1.5-2.5)	2.1 (1.5-2.6)

Data is represented as median (interquartile-range)

Table S2B. Summary of Kruskal–Wallis test (multiple comparison test) results performed on different clinical parameters in HC and a longitudinal cohort of vivax malaria patients

**Protein**	**One-way ANOVA**			**Multiple comparison test (Adjusted *P* value)**		
	P value	Number of groups	Kruskal-Wallis statistic	FEB vs. HC	DEF vs. HC	CON vs. HC
Hb	0.2222	4	4.392	0.5917	> 0.9999	0.1314
ESR	0.0102	4	11.3	0.0646	0.1009	> 0.9999
WBC	< 0.0001	4	27.42	0.0001	0.0001	0.0002
Neutrophils	0.0057	4	12.57	0.0805	0.1208	0.0023
Monocytes	0.0001	4	20.27	0.0033	0.0016	0.0008
Eosinophil	0.001	4	16.18	0.3374	0.0137	0.0006
Lymphocytes	0.1757	4	4.947	0.9427	> 0.9999	0.0801
Urea	0.0652	4	7.219	> 0.9999	> 0.9999	0.0572
Creatinine	0.1052	4	6.135	> 0.9999	> 0.9999	0.0459
Uric acid	0.002	4	14.77	0.0766	0.0498	0.0008
AST	< 0.0001	4	24.85	< 0.0001	0.0003	0.0812
ALT	0.0162	4	10.3	0.013	0.0647	> 0.9999

Table S3. Summary for statistically significant (p < 0.05) differentially abundant protein spots in low parasitemic vivax malaria patients compared to the healthy control subjects and moderately-high parasitemic vivax malaria patients visualized in 2D-DIGE gels

	**Appearance**	**LPVM *vs.* HC No. of Spots (Fold-change)**	**MPVM *vs.* LPVM No. of Spots (Fold-change)**
Up-regulated	24(24)	5 (2.3 to12.05)	0
	21(24)	3 (5.1 to 2.15)	2 (2.1 to 2.9)
	18(24)	7 (3.97 to 2.5)	0
Down-regulated	24(24)	1(-1.83)	4 (-3.3 to - 2.79)
	21(24)	1(-5.19)	11 (-4.82 to -2.37)
	18(24)	1(-11.59)	2 (-3.57 to - 3.67)

Table S4A. Differentially abundant proteins in LPVM compared to HC identified in 2D-DIGE in combination with MALDI-TOF/TOF analysis

S. No	UniProtKB accession number	Protein name	No. of spot(s)	Fold-change (LPVM/HC)
1	P04217	Alpha-1-B glycoprotein	3	(1.89 - 5.1)
2	P02748	Complement component C9	1	3.5
3	Q14624	Inter-alpha-trypsin inhibitor heavy chain H4	1	2.95
4	P00450	Ceruloplasmin	1	2.33
5	P00738	Haptoglobin	1	-1.83
6	P0DJI8	Serum amyloid A protein	1	12.05
7	P01871	Ig mu chain C region	1	2.48
8	P01011	Alpha-1-antichymotrypsin	1	3.48
9	P01009	Alpha-1-antitrypsin	1	2.29
10	P02766	Transthyretin	1	2.53
11	P02649	Apolipoprotein E	1	3.18

Peptide details

S. No	Spot ID	Fold-change	Protein Name	MW (kDa)	Protein score	Total ion score	Matched peptides	Peptide sequence
1	292	5.1	Alpha-1-B glycoprotein	54.24	776	724	13	GVTFLLR<br>LLELTGPK<br>CLAPLEGAR<br>ATWSGAVLAGR<br>LETPDFQLFK<br>SGLSTGWTQLSK<br>HQFLLTGDTQGR<br>CEGPIPDVTFELLR<br>NGVAQEPVHLDSPAIK<br>LELHVDGPPPRPQLR<br>VTLTCVAPLSGVDFQLR<br>IFFHLNAVALGDGGHYTCR<br>TPGAAANLELIFVGPQHAGNYR
2	301	3.97	Alpha-1-BN glycoprotein	54.3	900	821	16	GVTFLLR<br>IFFHLNAVALGDGGHYTCR<br>LELHVDGPPPRPQLR

								LETPDFQLFK<br>LLELTGPK<br>SGLSTGWTQLSK<br>*LHDNQNGWSGDSAPVELILSDETLPAPE FSPEPESGR*<br>ATWSGAVLAGR<br>CEGPIPDVTFELLR<br>CLAPLEGAR<br>HQFLLTGDTQGR<br>NGVAQEPVHLDSPAIK<br>SLPAPWLSMAPVSWITPGLK<br>SWVPHTFESELSDPVELLVAES<br>TPGAAANLELIFVGPQHAGNYR<br>VTLTCVAPLSGVDFQLR
3	134	3.5	Complement component C9	63.13	73	73	11	MKNAHLK<br>TSNFNAAISLK<br>SIEVFGQFNGKR<br>AIEDYINEFSVR<br>DVVLTTTFVDDIK<br>TEHYEEQIEAFK<br>RPWNVASLIYETK<br>NRDVVLTTTFVDDIK<br>MSPWSEWSQCDPCLR<br>NFRTEHYEEQIEAFK<br>QNLERAIEDYINEFSVR
4	136	2.95	Inter-alpha-trypsin inhibitor heavy chain H4	103.29	230	153	21	YYLQGAK<br>VRPQQLVK<br>YIFHNFMER<br>MNFRPGVLSSR<br>YIFHNFMER<br>VTIGLLFWDGR<br>AGFSWIEVTFK<br>FAHTVVTSRVVNR<br>ITFELVYEELLK<br>ANTVQEATFQMELPK<br>SPEQQETVLDGNLIIR<br>NPLVWVHASPEHVVVTR<br>QLGLPGPPDVPDHAAYHPFR<br>NPLVWVHASPEHVVVTRNR<br>RLDYQEGPPGVEISCWSVEL<br>QGPVNLLSDPEQGVEVTGQYER

								LPEGSVSLIILLTDGDPTVGETNPR<br>*LWAYLTIQQLLEQTVSASDADQQALR*<br>*FSSHVGGTLGQFYQEVLWGSPAASDDGR*<br>*AISGGSIQIENGYFVHYFAPEGLTTMPK*<br>*SFAAGIQALGGTNINDAMLMAVQLLDSSNQEER*
5	146	2.33	Ceruloplasmin	122.13	246	205	18	MYYSAVDPTK<br>GAYPLSIEPIGVR<br>QSEDSTFYLGER<br>DIASGLIGPLIICK<br>ALYLQYTDETFR<br>DLYSGLIGPLIVCR<br>NNEGTYYSPNYNPQSR<br>TTIEKPVWLGFLGPIIK<br>TYYIAAVEVEWDYSPQR<br>LISVDTEHSNIYLQNGPDR<br>KAEEEHLGILGPQLHADVGDK<br>HYYIGIIETTWDYASDHGEK<br>GPEEEHLGILGPVIWAEVGDTIR<br>SVPPSASHVAPTETFTYEWTVPK<br>EFYLFPTVFDENESLLLEDNIR<br>ERGPEEEHLGILGPVIWAEVGDTIR<br>KLEFALLFLVFDENESWYLDDNIK<br>DVDKEFYLFPTVFDENESLLLEDNIR
6	487	-1.83	Haptoglobin	45.17	239	192	11	DYAEVGR<br>GSFPWQAK<br>ILGGHLDAK<br>VGYVSGWGR<br>VTSIQDWVQK<br>SCAVAEYGVYVK<br>YVMLPVADQDQCIR<br>VVLHPNYSQVDIGLIK<br>SPVGVQPILNEHTFCAGMSK<br>SCAVAEYGVYVKVTSIQDWVQK<br>*YQEDTCYGDAGSAFAVHDLEEDTWYATGILSFDK*
7	1815	12.05	Serum amyloid A protein	13.52	215	176	6	GPGGVWAAEAISDAR<br>SFFSFLGEAFDGAR<br>RGPGGVWAAEAISDAR<br>DPNHFRPAGLPEKY<br>EANYIGSDKYFHAR

									FFGHGAEDSLADQAANEWGR
8	135	1.89	Alpha-1B-glycoprotein	54.3	900	821	16		GVTFLLR<br>LLELTGPK<br>CLAPLEGAR<br>ATWSGAVLAGR<br>LETPDFQLFK<br>SGLSTGWTQLSK<br>HQFLLTGDTQGR<br>CEGPIPDVTFELLR<br>NGVAQEPVHLDSPAIK<br>LELHVDGPPPRPQLR<br>VTLTCVAPLSGVDFQLR<br>IFFHLNAVALGDGGHYTCR<br>SLPAPWLSMAPVSWITPGLK<br>TPGAAANLELIFVGPQHAGNYR<br>SWVPHTFESELSDPVELLVAES<br>*LHDNQNGWSGDSAPVELILSDETLPAPE FSPEPESGR*
9	393	2.48	Ig mu chain C region	49.53	153	133	8		QIQVSWLR<br>LICQATGFSPR<br>YAATSQVLLPSK<br>YVTSAPMPEPQAPGR<br>VFAIPPSFASIFLTK<br>ESDWLSQSMFTCR<br>FTCTVTHTDLPSPLK<br>GVALHRPDVYLLPPAR
10	314	3.48	Alpha-1-antichymotrypsin	47.62	189	84	18		LINDYVK<br>FTEDAKR<br>ADLSGITGAR<br>EQLSLLDR<br>KLINDYVK<br>EIGELYLPK<br>NLAVSQVVHK<br>WRDSLEFR<br>ITLLSALVETR<br>MEEVEAMLLPETLK<br>WEMPFDPQDTHQSR<br>LYGSEAFATDFQDSAAAK<br>AVLDVFEEGTEASAATAVK<br>AKWEMPFDPQDTHQSR<br>DLDSQTMMVLVNYIFFK

									GTHVDLGLASANVDFAFSLYK
									DYNLNDILLQLGIEEAFTSK
									FNRPFLMIIVPTDTQNIFFMSK
11	34	2.29	Alpha-1-antitrypsin	46.7	505	384	20		SVLGQLGITK
									LSSWVLLMK
									FLENEDRR
									WERPFEVK
									KLSSWVLLMK
									LGMFNIQHCK
									GKWERPFEVK
									RLGMFNIQHCK
									DTVFALVNYIFFK
									ITPNLAEFAFSLYR
									TDTSHHDQDHPTFNK
									LQHLENELTHDIITK
									FNKPFVFLMIEQNTK
									DTEEEDFHVDQVTTVK
									LYHSEAFTVNFGDTEEAK
									ELDRDTVFALVNYIFFK
									LYHSEAFTVNFGDTEEAKK
									GTEAAGAMFLEAIPMSIPPEVK
									TLNQPDSQLQLTTGNGLFLSEGLK
									LQHLENELTHDIITKFLENEDR
12	1252	2.53	Transthyretin	15.9	88	76	3		GSPAINVAVHVFR
									ALGISPFHEHAEVVFTANDSGPR
									TSESGELHGLTTEEEFVEGIYK
13	826	3.18	Apolipoprotein E	36.13	1100	955	21		DADDLQK
									AQAWGER
									EQVAEVR
									FWDYLR
									WELALGR
									ELQAAQAR
									QWAGLVEK
									LAVYQAGAR
									LGPLVEQGR
									LQAEAFQAR
									LEEQAQQIR
									LGADMEDVCGR
									QQTEWQSGQR

								AKLEEQAQQIR
								AATVGSLAGQPLQER
								SWFEPLVEDMQR
								VEQAVETEPEPELR
								GEVQAMLGQSTEELR
								SELEEQLTPVAEETR
								AYKSELEEQLTPVAEETR
								WVQTLSEQVQEELLSSQVTQELR

Table S4B. Differentially abundant proteins in MPVM compared to LPVM identified in 2D-DIGE in combination with MALDI-TOF/TOF analysis

S. No	UniProtKB accession number	Protein name	No. of spot(s)	Fold-change (MPVM/LPVM)
1	P02790	Hemopexin	1	1.56
2	P01023	Alpha-2-macroglobulin	1	-2.62
3	P02647	Apolipoprotein A-1	2	(-2.37 to - 3.57)
4	P10909	Clusterin	2	(-1.59 to -1.78
5	P0C0L5	Complement C4	1	-2.33
6	P00738	Haptoglobin	6	(-2.33 to -3.67)
7	P02768	Serum albumin	5	(-2.45 to -3.94)
8	P0DJI8	Serum amyloid A	1	1.98

Peptide details

S. No	Spot ID	Fold-change	Protein Name	MW (kDa)	Protein score	Total ion score	Matched peptides	Peptide sequence
1	443	-3.01	Serum albumin	69.32	456	386	30	CCKADDK
								LVNEVTEFAK
								ALVLIAFAQYLQQCPFEDHVK
								AVMDDFAAFVEK
								CCAAADPHECYAK
								DDNPNLPR
								DVFLGMFLYEYAR
								EFNAETFTFHADICTLSEK
								EFNAETFTFHADICTLSEKER
								FKDLGEENFK
								FQNALLVR
								HPDYSVVLLLR
								HPYFYAPELLFFAK
								HPYFYAPELLFFAKR
								KVPQVSTPTLVEVSR
								LCTVATLR
								LKCASLQK
								LVRPEVDVMCTAFHDNEETFLK
								LVRPEVDVMCTAFHDNEETFLKK
								MPCAEDYLSVVLNQLCVLHEK

								QEPERNECFLQHK<br>QNCELFEQLGEYKFQNALLVR<br>RHPDYSVVLLLR<br>RHPYFYAPELLFFAK<br>RMPCAEDYLSVVLNQLCVLHEK<br>RMPCAEDYLSVVLNQLCVLHEK<br>RPCFSALEVDETYVPK<br>TCVADESAENCDKSLHTLFGDK<br>VFDEFKPLVEEPQNLIK<br>YLYEIAR
2	647	-2.79	Haptoglobin	45.17	248	198	12	NYYKLR<br>NPANPVQR<br>VMPICLPSK<br>YQCKNYYK<br>LPECEAVCGKPK<br>TEGDGVYTLNDKK<br>LRTEGDGVYTLNDK<br>YVMLPVADQDQCIR<br>LRTEGDGVYTLNNEK<br>AVGDKLPECEAVCGKPK<br>LPECEADDGCPKPPEIAHGYVEHSVR<br>*AVGDKLPECEADDGCPKPPEIAHGYVEHSVR*
3	435	1.56	Hemopexin	51.64	394	299	17	LHIMAGR<br>LWWLDLK<br>VWVYPPEK<br>RLWWLDLK<br>GGYTLVSGYPK<br>DYFMPCPGR<br>QGHNSVFLIK<br>WDRELISER<br>NFPSPVDAAFR<br>EWFWDLATGTMK<br>YYCFQGNQFLR<br>GECQAEGVLFFQGDR<br>LYLVQGTQVYVFLTK<br>SGAQATWTELPWPHEK<br>LLQDEFPGIPSPLDAAVECHR<br>EVGTPHGIILDSVDAAFICPGSSR<br>*DGWHSWPIAHQWPQGPSAVDAAFSWEEK*
4	1847	1.98	Serum amyloid A	13.52	215	176	6	GPGGVWAAEAISDAR

			protein					SFFSFLGEAFDGAR<br>RGPGGVWAAEAISDAR<br>DPNHFRPAGLPEKY<br>EANYIGSDKYFHAR<br>FFGHGAEDSLADQAANEWGR
5	165	-2.62	Alpha-2-macroglobulin	163.17	318	163	39	YGAATFTR<br>FQVDNNNR<br>QGIPFFGQVR<br>LPPNVVEESAR<br>YDVENCLANK<br>AIGYLNTGYQR<br>VGFYESDVMGR<br>VTAAPQSVCALR<br>NALFCLESAWK<br>HYDGSYSTFGER<br>NQGNTWLTAFVLK<br>LVHVEEPHTETVR<br>ALLAYAFALAGNQDK<br>IAQWQSFQLEGGLK<br>DNSVHWERPQKPK<br>TEHPFTVEEFVLPK<br>SSSNEEVMFLTVQVK<br>ALLAYAFALAGNQDKR<br>AHTSFQISLSVSYTGSR<br>LLIYAVLPTGDVIGDSAK<br>QFSFPLSSEPFQGSYK<br>VSVQLEASPAFLAVPVEK<br>GHFSISIPVKSDIAPVAR<br>FSGQLNSHGCFYQQVK<br>GNRIAQWQSFQLEGGLK<br>AFQPFFVELTMPYSVIR<br>LLLQQVSLPELPGEYSMK<br>VDLSFSPSQSLPASHAHLR<br>AFQPFFVELTMPYSVIR<br>MCPQLQQYEMHGPEGLR<br>AGAFCLSEDAGLGISSTASLR<br>MCPQLQQYEMHGPEGLR<br>LHTEAQIQEEGTVVELTGR<br>QQNAQGGFSSTQDTVVALHALSK

								SLFTDLEAENDVLHCVAFAVPK<br>NALFCLESAWKTAQEGDHGSHVYTK<br>VVSMDENFHPLNELIPLVYIQDPK<br>*YDVENCLANKVDLSFSPSQSLPASHAHLR*<br>*SPCYGYQWVSEEHEEAHHTAYLVFSPSK*
6	341	-2.33	Complement C4	192.6	201	170	19	EDSRAAFR<br>QGSFQGGFR<br>GLCVATPVQLR<br>KADGSYAAWLSR<br>GQIVFMNREPK<br>SFFPENWLWR<br>VHYTVCIWRNGK<br>CCQDGVTRLPMMR<br>AINEKLGQYASPTAK<br>EELVYELNPLDHR<br>EPFLSCCQFAESLR<br>GCGEQTMIYLAPTLAASR<br>LLATLCSAEVCQCAEGKCPR<br>ALEILQEEDLIDEDDIPVR<br>EGAIHREELVYELNPLDHR<br>VTASDPLDTLGSEGALSPGGVASLLR<br>TLEIPGNSDPNMIPDGDFNSYVR<br>STQDTVIALDALSAYWIASHTTEER<br>LQETSNWLLSQQQADGSFQDPCPVLDR
7	598	-3.73	Haptoglobin	45.17	217	150	13	DYAEVGR<br>QLVEIEK<br>GSFPWQAK<br>ILGGHLDAK<br>VGYVSGWGR<br>VMPICLPSK<br>VTSIQDWVQK<br>DIAPTLTLYVGK<br>SCAVAEYGVYVK<br>YVMLPVADQDQCIR<br>VSVNERVMPICLPSK<br>VMPICLPSKDYAEVGR<br>SPVGVQPILNEHTFCAGMSK
8	616	-3.98	Haptoglobin	45.17	239	192	11	DYAEVGR<br>GSFPWQAK<br>ILGGHLDAK

								VGYVSGWGR<br>VTSIQDWVQK<br>SCAVAEYGVYVK<br>YVMLPVADQDQCIR<br>VVLHPNYSQVDIGLIK<br>SPVGVQPILNEHTFCAGMSK<br>SCAVAEYGVYVKVTSIQDWVQK<br>*YQEDTCYGDAGSAFAVHDLEEDTWYATGI LSFDK*
9	638	-3.89	Haptoglobin	45.17	713	649	14	DYAEVGR<br>GSFPWQAK<br>VGYVSGWGR<br>VTSIQDWVQK<br>DIAPTLTLYVGK<br>TEGDGVYTLNDK<br>SCAVAEYGVYVK<br>YVMLPVADQDQCIR<br>VSVNERVMPICLPSK<br>VVLHPNYSQVDIGLIK<br>VMPICLPSKDYAEVGR<br>SPVGVQPILNEHTFCAGMSK<br>YVMLPVADQDQCIRHYEGSTVPEK<br>*YQEDTCYGDAGSAFAVHDLEEDTWYATGI LSFDK*
10	657	-2.45	Serum albumin	69.32	674	619	23	YLYEIAR<br>FQNALLVR<br>QTALVELVK<br>CASLQKFGER<br>ECCEKPLLEK<br>HPDYSVVLLLR<br>AVMDDFAAFVEK<br>RHPDYSVVLLLR<br>CCAAADPHECYAK<br>VPQVSTPTLVEVSR<br>DVFLGMFLYEYAR<br>YICENQDSISSKLK<br>KVPQVSTPTLVEVSR<br>QNCELFEQLGEYK<br>QEPERNECFLQHK<br>RPCFSALEVDETYVPK<br>VPQVSTPTLVEVSRNLGK<br>SLHTLFGDKLCTVATLR

								AAFTECCQAADKAACLLPK<br>VFDEFKPLVEEPQNLIK<br>EFNAETFTFHADICTLSEK<br>MPCAEDYLSVVLNQLCVLHEK<br>RMPCAEDYLSVVLNQLCVLHEK
11	662	-1.78	Clusterin	52.46	179	157	8	IDSLLENDR<br>RPHFFFPK<br>QTCMKFYAR<br>TLLSNLEEAKK<br>NPKFMETVAEK<br>ASSIIDELFQDR<br>QQTHMLDVMQDHFSR<br>EPQDTYHYLPFSLPHR
12	665	-1.59	Clusterin	52.46	210	201	7	FMETVAEK<br>ELDESLQVAER<br>ASSIIDELFQDR<br>EILSVDCSTNNPSQAK<br>LFDSDPITVTVPVEVSR<br>EPQDTYHYLPFSLPHR<br>CREILSVDCSTNNPSQAK
13	718	-3.21	Haptoglobin	45.17	586	511	14	DYAEVGR<br>GSFPWQAK<br>VGYVSGWGR<br>VMPICLPSK<br>VTSIQDWVQK<br>DIAPTLTLYVGK<br>SCAVAEYGVYVK<br>YVMLPVADQDQCIR<br>AVGDKLPECEAVCGKPK<br>VMPICLPSKDYAEVGR<br>SPVGVQPILNEHTFCAGMSK<br>LPECEAVCGKPKNPANPVQR<br>FTDHLKYVMLPVADQDQCIR<br>YQEDTCYGDAGSAFAVH
14	1037	-2.37	Apolipoprotein A-1	30.75	934	719	24	QKVEPLR<br>AELQEGAR<br>AKPALEDLR<br>LSPLGEEMR<br>QKLHELQEK<br>LEALKENGGAR

								QGLLPVLESFK
								DLATVYVDVLK
								VQPYLDDFQK
								WQEEMELYR
								THLAPYSDELR
								ETEGLRQEMSK
								VQPYLDDFQKK
								VSFLSALEEYTK
								DYVSQFEGSALGK
								KWQEEMELYR
								VKDLATVYVDVLK
								VEPLRAELQEGAR
								VSFLSALEEYTKK
								LLDNWDSVTSTFSK
								DSGRDYVSQFEGSALGK
								EQLGPVTQEFWDNLEK
								LREQLGPVTQEFWDNLEK
								EQLGPVTQEFWDNLEKETEGLR
15	626	-3.57	Apolipoprotein A-I	30.75	530	394	19	QKVEPLR
								AELQEGAR
								AKPALEDLR
								LSPLGEEMR
								QGLLPVLESFK
								DLATVYVDVLK
								VQPYLDDFQK
								WQEEMELYR
								THLAPYSDELR
								VQPYLDDFQKK
								DYVSQFEGSALGK
								KWQEEMELYR
								VKDLATVYVDVLK
								LLDNWDSVTSTFSK
								DSGRDYVSQFEGSALGK
								EQLGPVTQEFWDNLEK
								LREQLGPVTQEFWDNLEK
								QLNLKLLDNWDSVTSTFSK
								AKPALEDLRQGLLPVLESFK
16	642	-3.67	Haptoglobin	45.17	248	198	12	NYYKLR
								NPANPVQR
								VMPICLPSK

								YQCKNYYK<br>LPECEAVCGKPK<br>TEGDGVYTLNDKK<br>LRTEGDGVYTLNDK<br>YVMLPVADQDQCIR<br>LRTEGDGVYTLNNEK<br>AVGDKLPECEAVCGKPK<br>LPECEADDGCPKPPEIAHGYVEHSVR<br>*AVGDKLPECEADDGCPKPPEIAHGYVEHSVR*
17	447	-3.3	Serum albumin	69.32	456	386	30	CCKADDK<br>YLYEIAR<br>LCTVATLR<br>DDNPNLPR<br>LKCASLQK<br>FQNALLVR<br>LVNEVTEFAK<br>FKDLGEENFK<br>HPDYSVVLLLR<br>AVMDDFAAFVEK<br>RHPDYSVVLLLR<br>CCAAADPHECYAK<br>DVFLGMFLYEYAR<br>KVPQVSTPTLVEVSR<br>QEPERNECFLQHK<br>HPYFYAPELLFFAK<br>RHPYFYAPELLFFAK<br>HPYFYAPELLFFAKR<br>RPCFSALEVDETYVPK<br>VFDEFKPLVEEPQNLIK<br>EFNAETFTFHADICTLSEK<br>ALVLIAFAQYLQQCPFEDHVK<br>TCVADESAENCDKSLHTLFGDK<br>MPCAEDYLSVVLNQLCVLHEK<br>EFNAETFTFHADICTLSEKER<br>QNCELFEQLGEYKFQNALLVR<br>RMPCAEDYLSVVLNQLCVLHEK<br>LVRPEVDVMCTAFHDNEETFLK<br>RMPCAEDYLSVVLNQLCVLHEK<br>LVRPEVDVMCTAFHDNEETFLKK
18	402	-3.14	Serum albumin	69.32	663	568	26	CCKADDK

									YLYEIAR
									DDNPNLPR
									FQNALLVR
									CASLQKFGER
									HPDYSVVLLLR
									AVMDDFAAFVEK
									RHPDYSVVLLLR
									CCAAADPHECYAK
									DVFLGMFLYEYAR
									KVPQVSTPTLVEVSR
									QNCELFEQLGEYK
									QEPERNECFLQHK
									HPYFYAPELLFFAK
									RPCFSALEVDETYVPK
									NECFLQHKDDNPNLPR
									VFDEFKPLVEEPQNLIK
									VHTECCHGDLLECADDR
									EFNAETFTFHADICTLSEK
									MPCAEDYLSVVLNQLCVLHEK
									EFNAETFTFHADICTLSEKER
									VHTECCHGDLLECADDRADLAK
									LVRPEVDVMCTAFHDNEETFLK
									RMPCAEDYLSVVLNQLCVLHEK
									LVRPEVDVMCTAFHDNEETFLKK
									CCTESLVNRRPCFSALEVDETYVPK
19	432	-3.94	Serum albumin	69.3	674	619	23		YLYEIAR
									FQNALLVR
									QTALVELVK
									CASLQKFGER
									ECCEKPLLEK
									HPDYSVVLLLR
									AVMDDFAAFVEK
									RHPDYSVVLLLR
									CCAAADPHECYAK
									VPQVSTPTLVEVSR
									DVFLGMFLYEYAR
									YICENQDSISSKLK
									KVPQVSTPTLVEVSR
									QNCELFEQLGEYK
									QEPERNECFLQHK

									RPCFSALEVDETYVPK
									VPQVSTPTLVEVSRNLGK
									SLHTLFGDKLCTVATLR
									AAFTECCQAADKAACLLPK
									VFDEFKPLVEEPQNLIK
									EFNAETFTFHADICTLSEK
									MPCAEDYLSVVLNQLCVLHEK
									RMPCAEDYLSVVLNQLCVLHEK

Table S5A. Complete details of protein identification and quantitative iTRAQ data for low and moderately-high parasitemic vivax malaria patients (compared to HC) obtained from Q-TOF analysis

S. No	Num Peps Unique	Score Unique	LPVM/HC	MPVM/HC	Accession number	Protein Name
1	105	1405.88	0.97	0.997	P04114	Apolipoprotein B-100
2	97	1428.41	0.901	0.935	P01024	Complement C3
3	63	944.82	0.789	0.776	P01023	Alpha-2-macroglobulin
4	55	811.8	0.843	0.902	P0C0L5	Complement C4-B
5	37	581.74	1.142	1.501	P01009	Alpha-1-antitrypsin
6	37	551.24	0.634	0.461	P02647	Apolipoprotein A-I
7	32	494.21	0.822	0.849	P02787	Serotransferrin
8	28	453.55	1.166	1.366	P00450	Ceruloplasmin
9	26	404.28	0.473	0.347	P02768	Serum albumin
10	24	361.11	1.385	1.791	P01011	Alpha-1-antichymotrypsin
11	24	295.57	0.609	0.836	P02751	Fibronectin
12	24	345.65	1.115	1.208	Q14624	Inter-alpha-trypsin inhibitor heavy chain H4
13	22	313.87	1.013	0.533	P00738	Haptoglobin
14	22	309.19	0.862	0.929	P01008	Antithrombin-III
15	22	276.09	0.965	1.235	P00751	Complement factor B
16	20	271.89	0.817	0.828	P06727	Apolipoprotein A-IV
17	18	248.29	1.022	1.089	P05155	Plasma protease C1 inhibitor
18	18	234.26	1.05	1.14	P02790	Hemopexin
19	18	228.34	0.76	0.778	P06396	Gelsolin
20	17	198.42	0.745	0.896	P19823	Inter-alpha-trypsin inhibitor heavy chain H2
21	15	221.76	1.331	1.34	P01871	Ig mu chain C region
22	15	207.85	1.035	1.179	P02774	Vitamin D-binding protein
23	14	171.06	0.965	1.163	P25311	Zinc-alpha-2-glycoprotein
24	13	167.29	1.169	1.064	P04196	Histidine-rich glycoprotein
25	12	181.25	1.133	1.079	P01019	Angiotensinogen
26	12	143.17	0.825	0.874	P43652	Afamin
27	12	140.25	1.008	1.129	P02649	Apolipoprotein E
28	12	135.85	1.119	1.308	P01031	Complement C5
29	12	135.46	1.304	1.016	P02671	Fibrinogen alpha chain
30	12	132.01	1.123	1.158	P36955	Pigment epithelium-derived factor
31	11	167.38	0.76	0.738	P02766	Transthyretin
32	11	159.86	1.1	1.262	P01876	Ig alpha-1 chain C region
33	11	148.84	0.847	0.984	P10909	Clusterin
34	11	137.27	0.853	0.933	P19827	Inter-alpha-trypsin inhibitor heavy chain H1

35	10	130.42	1.462	1.699	P02750	Leucine-rich alpha-2-glycoprotein
36	10	168.67	1.172	1.762	P68871	Hemoglobin subunit beta
37	10	146.59	1	1.153	P04217	Alpha-1B-glycoprotein
38	10	136.53	0.935	0.984	P08603	Complement factor H
39	9	136.56	1.354	1.742	P19652	Alpha-1-acid glycoprotein 2
40	9	111.8	1.117	1.093	P08697	Alpha-2-antiplasmin
41	9	88.62	0.735	0.773	P00734	Prothrombin
42	8	124.81	0.897	0.749	P01857	Ig gamma-1 chain C region
43	8	93.77	0.893	0.918	P01042	Kininogen-1
44	7	109.69	1.046	1.522	P69905	Hemoglobin subunit alpha
45	7	103.08	0.724	0.479	P02652	Apolipoprotein A-II
46	7	99.15	1.466	1.376	P0DJI8	Serum amyloid A-1 protein
47	7	96.01	0.924	0.893	P27169	Serum paraoxonase/arylesterase 1
48	7	91.36	0.947	0.765	Q96PD5	N-acetylmuramoyl-L-alanine amidase
49	6	95	1.049	1.123	P04004	Vitronectin
50	6	69.74	1.026	1.027	Q06033	Inter-alpha-trypsin inhibitor heavy chain H3
51	6	69	0.812	0.823	P05546	Heparin cofactor 2
52	6	51.5	1.229	1.126	P06681	Complement C2
53	5	62.97	1.613	8.574	P02741	C-reactive protein
54	5	61.11	0.586	1.638	P18428	Lipopolysaccharide-binding protein
55	5	76.83	1.107	1.431	P02748	Complement component C9
56	5	71.09	1.044	0.635	Q9BVA1	Tubulin beta-2B chain
57	5	66.38	0.81	0.857	P02765	Alpha-2-HS-glycoprotein
58	5	60.72	0.926	1.137	P02743	Serum amyloid P-component
59	5	60.6	0.879	0.742	P13671	Complement component C6
60	5	59.36	0.629	0.743	P51884	Lumican
61	5	57.79	0.916	1.069	P22792	Carboxypeptidase N subunit 2
62	5	51.02	0.931	1.468	P05543	Thyroxine-binding globulin
63	5	46.44	0.945	0.729	P29622	Kallistatin
64	4	49.03	0.983	0.623	P01861	Ig gamma-4 chain C region
65	4	46.82	0.606	1.115	P02675	Fibrinogen beta chain
66	4	67.37	1.064	1.085	B9A064	Immunoglobulin lambda-like polypeptide 5
67	4	62.32	1.094	1.064	P01834	Ig kappa chain C region
68	4	52.61	0.995	0.907	P00747	Plasminogen
69	4	51.85	1.05	1.248	P00736	Complement C1r subcomponent
70	4	51.1	0.759	0.835	P02655	Apolipoprotein C-II
71	4	38.93	0.808	0.999	P08185	Corticosteroid-binding globulin
72	4	37.59	0.977	0.987	P04003	C4b-binding protein alpha chain
73	3	29.11	2.305	0.512	P01880	Ig delta chain C region

74	3	21.56	0.836	0.236	P35858	Insulin-like growth factor-binding protein complex acid labile subunit
75	3	35.14	4.408	1.123	P08670	Vimentin
76	3	32.32	1.448	1.444	P14136	Glial fibrillary acidic protein
77	3	44.45	0.947	0.978	P41222	Prostaglandin-H2 D-isomerase
78	3	44.34	0.95	0.988	P02760	Protein AMBP
79	3	40.31	0.708	0.978	P02749	Beta-2-glycoprotein 1
80	3	36.8	0.815	0.794	P01034	Cystatin-C
81	3	35.96	0.949	1.312	P01620	Ig kappa chain V-III region SIE
82	3	34.19	1.048	0.862	P05156	Complement factor I
83	3	31.94	0.841	0.993	P09871	Complement C1s subcomponent
84	3	31.4	0.86	0.916	P05090	Apolipoprotein D
85	3	30.03	0.908	0.861	P35542	Serum amyloid A-4 protein
86	3	21.18	0.688	1.013	Q96DD0	Leucine-rich repeat-containing protein 39
87	2	15.15	1.457	0.489	P25098	Beta-adrenergic receptor kinase 1
88	2	13.67	1.689	3.766	Q8WZ42	Titin
89	2	26.07	0.797	2.058	P02686	Myelin basic protein
90	2	22.36	1.384	1.691	P20848	Putative alpha-1-antitrypsin-related protein
91	2	12.92	0.915	1.933	Q9H267	Vacuolar protein sorting-associated protein 33B
92	2	26.9	1.681	1.448	P20671	Histone H2A type 1-D
93	2	23.64	0.548	1.319	P02679	Fibrinogen gamma chain
94	2	18.54	1.529	1.148	O75369	Filamin-B
95	2	13.48	1.981	1.268	Q15349	Ribosomal protein S6 kinase alpha-2
96	2	32.15	0.765	1.053	P08571	Monocyte differentiation antigen CD14
97	2	29.1	1.213	0.867	P07358	Complement component C8 beta chain
98	2	28.84	0.812	1.108	P01621	Ig kappa chain V-III region NG9
99	2	27.3	1.072	1.229	P01766	Ig heavy chain V-III region BRO
100	2	26.2	1.027	0.685	P22352	Glutathione peroxidase 3
101	2	25.59	1.154	1.237	Q08380	Galectin-3-binding protein
102	2	25.22	1.266	1.174	P07357	Complement component C8 alpha chain
103	2	24.78	0.863	1.417	P61769	Beta-2-microglobulin
104	2	24.11	0.972	0.764	P01774	Ig heavy chain V-III region POM
105	2	24.05	1.141	1.056	P01591	Immunoglobulin J chain
106	2	23.62	1.033	1.178	P07360	Complement component C8 gamma chain
107	2	23.08	0.874	0.895	O14791	Apolipoprotein L1
108	2	22.71	1.029	0.973	P10643	Complement component C7
109	2	18.25	1.064	1.097	P01781	Ig heavy chain V-III region GAL
110	2	17.8	0.763	1.246	O15020	Spectrin beta chain, non-erythrocytic 2
111	2	15.54	0.833	0.972	P02775	Platelet basic protein
112	2	14.89	0.958	1.177	A8TX70	Collagen alpha-5(VI) chain

113	2	14.65	0.678	0.632	P05060	Secretogranin-1
114	2	13.47	0.688	1.202	Q8NB66	Protein unc-13 homolog C
115	2	13.44	0.688	1.246	Q9Y6D9	Mitotic spindle assembly checkpoint protein MAD1
116	1	15.95	3.057	1.763	P04275	von Willebrand factor
117	1	15.57	0.382	0.222	P34931	Heat shock 70 kDa protein 1-like
118	1	12.78	0.608	0.413	Q14BN4	Sarcolemmal membrane-associated protein
119	1	11.12	2.726	2.055	P59666	Neutrophil defensin 3
120	1	10.43	0.349	0.382	P10720	Platelet factor 4 variant
121	1	9.08	2.043	2.99	P0C1S8	Wee1-like protein kinase 2
122	1	9	3.218	2.167	O15553	Pyrin
123	1	8.84	0.371	0.131	Q14966	Zinc finger protein 638
124	1	8.27	0.602	0.619	P51648	Fatty aldehyde dehydrogenase
125	1	7.98	2.035	2.455	Q9Y3T9	Nucleolar complex protein 2 homolog
126	1	7.84	0.348	0.248	P07202	Thyroid peroxidase
127	1	7.76	2.87	1.891	Q15431	Synaptonemal complex protein 1
128	1	7.68	0.455	0.401	P0CF97	Putative protein FAM200B
129	1	7.68	0.511	0.489	Q9NVW2	E3 ubiquitin-protein ligase RLIM
130	1	7.65	1.534	2.852	O14638	Ectonucleotide pyrophosphatase/phosphodiesterase family member 3
131	1	6.7	1.446	1.908	P55199	RNA polymerase II elongation factor ELL
132	1	6.61	1.74	1.635	Q14008	Cytoskeleton-associated protein 5
133	1	10.95	0.885	0.481	A6NKB5	Pecanex-like protein 2
134	1	10.58	1.367	0.606	P15169	Carboxypeptidase N catalytic chain
135	1	9.98	0.788	0.331	Q04756	Hepatocyte growth factor activator
136	1	9.93	1.041	1.645	Q9UHP3	Ubiquitin carboxyl-terminal hydrolase 25
137	1	9.92	0.701	0.606	P05154	Plasma serine protease inhibitor
138	1	9.8	0.763	0.215	Q5VTE0	Elongation factor 1-alpha 2
139	1	8.71	0.662	1.587	Q562R1	Beta-actin-like protein 2
140	1	8.52	0.675	2.447	Q96IG2	F-box/LRR-repeat protein 20
141	1	8.46	1.253	0.583	A6NNL0	Protein FAM22B
142	1	8.32	0.792	1.661	Q96LX7	Coiled-coil domain-containing protein 17
143	1	8.21	1.058	0.497	Q96JI7	Spatacsin
144	1	8.17	1.04	0.421	Q5VWG9	Transcription initiation factor TFIID subunit 3
145	1	8.02	1.183	2.542	Q8IUA7	ATP-binding cassette sub-family A member 9
146	1	8	1.185	1.692	P80419	Ig heavy chain V-III region GAR
147	1	7.75	1.383	1.549	Q12923	Tyrosine-protein phosphatase non-receptor type 13
148	1	7.23	1.29	1.966	O60494	Cubilin
149	1	6.79	0.681	0.465	O60507	Protein-tyrosine sulfotransferase 1

150	1	6.69	1.272	0.367	Q6KC79	Nipped-B-like protein
151	1	6.59	0.683	0.388	Q14764	Major vault protein
152	1	6.14	1	0.522	Q03426	Mevalonate kinase
153	1	16.89	0.579	0.756	P00748	Coagulation factor XII
154	1	9.02	0.62	0.639	Q5JTH9	RRP12-like protein
155	1	8.72	0.473	1.035	Q5T4S7	E3 ubiquitin-protein ligase UBR4
156	1	8.36	0.452	1.184	Q9UIM3	FK506-binding protein-like
157	1	8.19	0.507	0.755	O15240	Neurosecretory protein VGF
158	1	8.01	0.25	0.636	Q6NS38	Alpha-ketoglutarate-dependent dioxygenase alkB homolog 2
159	1	7.89	0.622	0.826	Q5T197	DC-STAMP domain-containing protein 1
160	1	7.89	1.502	0.971	Q8WWI5	Choline transporter-like protein 1
161	1	7.85	1.858	1.325	Q16566	Calcium/calmodulin-dependent protein kinase type IV
162	1	7.84	2.325	1.156	P43251	Biotinidase
163	1	7.71	0.407	0.884	Q9NRP7	Serine/threonine-protein kinase 36
164	1	7.34	0.2	0.685	O95447	Lebercilin-like protein
165	1	7.33	1.935	1.022	P01266	Thyroglobulin
166	1	6.96	0.574	0.666	Q9P2D8	Protein unc-79 homolog
167	1	6.95	0.321	0.837	P41208	Centrin-2
168	1	6.47	0.604	0.776	Q13601	KRR1 small subunit processome component homolog
169	1	6.14	0.246	1.041	Q9HBA0	Transient receptor potential cation channel subfamily V member 4
170	1	22.42	0.873	1.06	P01593	Ig kappa chain V-I region AG
171	1	21.35	1.382	1.295	P01598	Ig kappa chain V-I region EU
172	1	20.89	1.162	1.149	P01625	Ig kappa chain V-IV region Len
173	1	20.27	1.1	1.035	P02656	Apolipoprotein C-III
174	1	19.59	1.053	0.798	P01611	Ig kappa chain V-I region Wes
175	1	18.56	1.179	1.487	P01717	Ig lambda chain V-IV region Hil
176	1	15.01	0.669	0.747	P04406	Glyceraldehyde-3-phosphate dehydrogenase
177	1	13.45	0.85	1.107	P01715	Ig lambda chain V-IV region Bau
178	1	13.41	0.692	0.655	P03952	Plasma kallikrein
179	1	13.33	1.099	1	P06310	Ig kappa chain V-II region RPMI 6410
180	1	12.36	1.346	1.295	P04207	Ig kappa chain V-III region CLL
181	1	10.87	1.085	1.072	Q6UW60	Proprotein convertase subtilisin/kexin type 4
182	1	10.67	0.988	0.93	P20851	C4b-binding protein beta chain
183	1	10.43	1.093	1.393	Q5R372	Rab GTPase-activating protein 1-like
184	1	10.27	1	1	P05067	Amyloid beta A4 protein
185	1	10.21	1.035	1.413	P26045	Tyrosine-protein phosphatase non-receptor type 3
186	1	10.16	1	1	Q6PEY2	Tubulin alpha-3E chain

187	1	10.16	0.848	0.774	Q9P2D7	Dynein heavy chain 1, axonemal
188	1	10.02	1.288	0.881	P01700	Ig lambda chain V-I region HA
189	1	10	0.982	0.865	Q9UGM5	Fetuin-B
190	1	9.83	0.965	1.075	Q9Y6A9	Signal peptidase complex subunit 1
191	1	9.81	0.901	1.064	P13533	Myosin-6
192	1	9.72	0.735	1.231	O95445	Apolipoprotein M
193	1	9.71	0.837	0.858	Q6PIF6	Unconventional myosin-VIIb
194	1	9.66	0.896	0.812	P48736	Phosphatidylinositol 4,5-bisphosphate 3-kinase catalytic subunit gamma isoform
195	1	9.61	1	1	P13796	Plastin-2
196	1	9.49	1.141	0.699	Q96L42	Potassium voltage-gated channel subfamily H member 8
197	1	9.28	0.745	1.015	Q96KR7	Phosphatase and actin regulator 3
198	1	9	0.65	1.001	O75717	WD repeat and HMG-box DNA-binding protein 1
199	1	8.91	0.991	1.153	Q9H467	CUE domain-containing protein 2
200	1	8.76	1.324	0.983	O60583	Cyclin-T2
201	1	8.74	0.893	0.878	Q5TBE3	Uncharacterized protein C9orf153
202	1	8.73	1.087	1.358	Q96NB2	Sideroflexin-2
203	1	8.67	0.803	0.975	Q9P2R6	Arginine-glutamic acid dipeptide repeats protein
204	1	8.56	1.04	0.935	P28289	Tropomodulin-1
205	1	8.55	0.796	0.751	P78332	RNA-binding protein 6
206	1	8.53	0.943	0.988	Q9Y4B4	Helicase ARIP4
207	1	8.32	1	1.221	Q9NPG3	Ubinuclein-1
208	1	8.25	1.08	1.134	Q86UW6	NEDD4-binding protein 2
209	1	8.23	0.796	0.95	Q12791	Calcium-activated potassium channel subunit alpha-1
210	1	7.99	0.803	0.878	Q9BXG8	Spermatogenic leucine zipper protein 1
211	1	7.94	0.951	0.807	Q5BKU6	Putative uncharacterized protein LOC641298
212	1	7.88	0.888	1.178	Q07617	Sperm-associated antigen 1
213	1	7.82	1.296	0.915	Q9UF47	DnaJ homolog subfamily C member 5B
214	1	7.78	0.998	0.885	B2RTY4	Unconventional myosin-IXa
215	1	7.71	1.376	0.935	P35523	Chloride channel protein 1
216	1	7.7	1.29	0.905	Q7Z2K8	G protein-regulated inducer of neurite outgrowth 1
217	1	7.68	0.934	0.641	Q567V2	Mpv17-like protein 2
218	1	7.68	0.807	0.651	Q92932	Receptor-type tyrosine-protein phosphatase N2
219	1	7.64	1.157	0.651	P16234	Platelet-derived growth factor receptor alpha
220	1	7.64	0.949	1	Q12805	EGF-containing fibulin-like extracellular matrix protein 1
221	1	6.83	0.639	0.717	Q8IYM0	Protein FAM186B
222	1	6.73	0.793	1.029	Q5JR59	Microtubule-associated tumor suppressor candidate 2

223	1	6.71	1	1	Q96PN6	Adenylate cyclase type 10
224	1	6.68	1.121	1.402	Q02952	A-kinase anchor protein 12
225	1	6.44	1.363	0.682	P21359	Neurofibromin
226	1	6.31	1	1	Q9HC10	Otoferlin

Table S5B (I). Differentially abundant proteins in low parasitemic vivax malaria patients (compared to HC) identified by iTRAQ-based quantitative proteomics analysis

S No.	Name of proteins	No. unique peptides	Fold-change (LPVM/HC)
1	Vimentin	3	4.41
2	Pyrin	1	3.22
3	von Willebrand factor	1	3.06
4	Synaptonemal complex protein 1	1	2.87
5	Neutrophil defensin 3	1	2.73
6	Biotinidase	1	2.33
7	Ig delta chain C region	3	2.31
8	Wee1-like protein kinase 2	1	2.04
9	Nucleolar complex protein 2 homolog	1	2.04
10	Ribosomal protein S6 kinase alpha-2	2	1.98
11	Thyroglobulin	1	1.94
12	Calcium/calmodulin-dependent protein kinase type IV	1	1.86
13	Cytoskeleton-associated protein 5	1	1.74
14	Titin	2	1.69
15	Histone H2A type 1-D	2	1.68
16	C-reactive protein	5	1.61
17	Ectonucleotide pyrophosphatase/phosphodiesterase family member 3	1	1.53
18	Filamin-B	2	1.53
19	Choline transporter-like protein 1	1	1.50
20	Serum amyloid A-1 protein	7	1.47
21	Leucine-rich alpha-2-glycoprotein	10	1.46
22	Beta-adrenergic receptor kinase 1	2	1.46
23	Glial fibrillary acidic protein	3	1.45
24	RNA polymerase II elongation factor ELL	1	1.45
25	RRP12-like protein	1	0.62
26	Fibronectin	24	0.61
27	Sarcolemmal membrane-associated protein	1	0.61
28	Fibrinogen beta chain	4	0.61
29	KRR1 small subunit processome component homolog	1	0.60
30	Fatty aldehyde dehydrogenase	1	0.60
31	Lipopolysaccharide-binding protein	5	0.59
32	Coagulation factor XII	1	0.58
33	Protein unc-79 homolog	1	0.57
34	Fibrinogen gamma chain	2	0.55
35	E3 ubiquitin-protein ligase RLIM	1	0.51

36	Neurosecretory protein VGF	1	0.51
37	Serum albumin	26	0.47
38	E3 ubiquitin-protein ligase UBR4	1	0.47
39	Putative protein FAM200B	1	0.46
40	FK506-binding protein-like	1	0.45
41	Serine/threonine-protein kinase 36	1	0.41
42	Heat shock 70 kDa protein 1-like	1	0.38
43	Zinc finger protein 638	1	0.37
44	Platelet factor 4 variant	1	0.35
45	Thyroid peroxidase	1	0.35
46	Centrin-2	1	0.32
47	Alpha-ketoglutarate-dependent dioxygenase alkB homolog 2	1	0.25
48	Transient receptor potential cation channel subfamily V member 4	1	0.25
49	Lebercilin-like protein	1	0.20

Table S5B (II). Differentially abundant proteins in moderately-high parasitemic vivax malaria patients (compared to HC) identified by iTRAQ-based quantitative proteomics analysis

S No.	Name of proteins	No. unique peptides	Fold-change (MPVM/HC)
1	C-reactive protein	5	8.574
2	Titin	2	3.766
3	Wee1-like protein kinase 2	1	2.99
4	Ectonucleotide pyrophosphatase/phosphodiesterase family member 3	1	2.852
5	ATP-binding cassette sub-family A member 9	1	2.542
6	Nucleolar complex protein 2 homolog	1	2.455
7	F-box/LRR-repeat protein 20	1	2.447
8	Pyrin	1	2.167
9	Myelin basic protein	2	2.058
10	Neutrophil defensin 3	1	2.055
11	Cubilin	1	1.966
12	Vacuolar protein sorting-associated protein 33B	2	1.933
13	RNA polymerase II elongation factor ELL	1	1.908
14	Synaptonemal complex protein 1	1	1.891
15	Alpha-1-antichymotrypsin	24	1.791
16	von Willebrand factor	1	1.763
17	Hemoglobin subunit beta	10	1.762
18	Alpha-1-acid glycoprotein 2	9	1.742
19	Leucine-rich alpha-2-glycoprotein	10	1.699
20	Ig heavy chain V-III region GAR	1	1.692
21	Putative alpha-1-antitrypsin-related protein	2	1.691
22	Coiled-coil domain-containing protein 17	1	1.661
23	Ubiquitin carboxyl-terminal hydrolase 25	1	1.645
24	Lipopolysaccharide-binding protein	5	1.638
25	Cytoskeleton-associated protein 5	1	1.635
26	Beta-actin-like protein 2	1	1.587
27	Tyrosine-protein phosphatase non-receptor type 13	1	1.549
28	Hemoglobin subunit alpha	7	1.522
29	Alpha-1-antitrypsin	37	1.501
30	Ig gamma-4 chain C region	4	0.623
31	Fatty aldehyde dehydrogenase	1	0.619
32	Carboxypeptidase N catalytic chain	1	0.606
33	Plasma serine protease inhibitor	1	0.606
34	Protein FAM22B	1	0.583
35	Haptoglobin	22	0.533

36	Mevalonate kinase	1	0.522
37	Ig delta chain C region	3	0.512
38	Spatacsin	1	0.497
39	Beta-adrenergic receptor kinase 1	2	0.489
40	E3 ubiquitin-protein ligase RLIM	1	0.489
41	Pecanex-like protein 2	1	0.481
42	Apolipoprotein A-II	7	0.479
43	Protein-tyrosine sulfotransferase 1	1	0.465
44	Apolipoprotein A-I	37	0.461
45	Transcription initiation factor TFIID subunit 3	1	0.421
46	Sarcolemmal membrane-associated protein	1	0.413
47	Putative protein FAM200B	1	0.401
48	Major vault protein	1	0.388
49	Platelet factor 4 variant	1	0.382
50	Nipped-B-like protein	1	0.367
51	Serum albumin	26	0.347
52	Hepatocyte growth factor activator	1	0.331
53	Thyroid peroxidase	1	0.248
54	Insulin-like growth factor-binding protein complex acid labile subunit	3	0.236
55	Heat shock 70 kDa protein 1-like	1	0.222
56	Elongation factor 1-alpha 2	1	0.215
57	Zinc finger protein 638	1	0.131

Table S6A. Differentially abundant serum proteins in FEB stage of the infection compared to HC subjects identified in 2D-DIGE combined with MALDI-TOF/TOF

S. No.	UniProtKB accession number	Protein name	No. of Spot	Fold-change (FEB/HC)
1	P01011	Alpha-1-antichymotrypsin	1	1.84
2	P01009	Alpha-1-antitrypsin	2	(-2.13 to -6.02)
3	P02647	Apolipoprotein A-I	1	2.44
4	P51530	DNA2-like helicase	1	-3.09
5	O75636	Ficolin 3	1	-2.29
6	P00738	Haptoglobin	7	(-1.93 to -6.36)
7	P02790	Hemopexin	3	(3.54 to 3.74)
8	P02750	Leucine-rich alpha-2-glycoprotein	2	(1.79 to -2.13)
9	P02768	Serum albumin	1	-7.73
10	P0DJI8	Serum amyloid A	1	11.26
11	P02766	Transthyretin	1	-1.92
12	P25311	Zinc-alpha-2-glycoprotein	1	-2.08

Peptide details

S. No.	Gel ID	Fold-change	Protein name	Mw	Protein score	Total ion score	Matched peptides	Peptide sequence
1	1117	11.26	Serum Amyloid A	13.52	212	164	7	GPGGVWAAEAISDAR<br>SFFSFLGEAFDGAR<br>RGPGGVWAAEAISDAR<br>DPNHFRPAGLPEKY<br>EANYIGSDKYFHAR<br>MKLLTGLVFCSLVLGVSSR<br>FFGHGAEDSLADQAANEWGR
2	309	3.54	Hemopexin	5238<br>4.6	280	250	15	LHIMAGR<br>VWVYPPEK<br>VWVYPPEK<br>GGYTLVSGYPK<br>DYFMPCPGR<br>DYFMPCPGR<br>DYFMPCPGR<br>NFPSPVDAAFR<br>NFPSPVDAAFR<br>FDPVRGEVPPR<br>FDPVRGEVPPR<br>YYCFQGNQFLR<br>GECQAEGVLFFQGDR<br>GECQAEGVLFFQGDR<br>EWFWDLATGTMKER

3	301	3.38	Hemopexin	5238<br>4.6	114	96	10	LWWLDLK<br>VWVYPPEK<br>VDGALCMEK<br>GGYTLVSGYPK<br>GGYTLVSGYPK<br>DYFMPCPGR<br>DYFMPCPGR<br>NFPSPVDAAFR<br>NFPSPVDAAFR<br>GECQAEGVLFFQGDR
4	955	2.44	Apolipoprotein A-I	30.75	154	119	15	QKVEPLR<br>QKVEPLR<br>AELQEGAR<br>AELQEGAR<br>LHELQEK<br>AKPALEDLR<br>LSPLGEEMR<br>ATEHLSTLSEK<br>VQPYLDDFQK<br>VQPYLDDFQK<br>VQPYLDDFQK<br>WQEEMELYR<br>THLAPYSDELR<br>DYVSQFEGSALGK<br>DYVSQFEGSALGK
5	465	1.84	alpha-1-antichymotrypsin	47.62	258	182	15	ADLSGITGAR<br>EQLSLLDR<br>EIGELYLPK<br>WRDSLEFR<br>ITLLSALVETR<br>WEMPFDPQDTHQSR<br>MEEVEAMLLPETLKR<br>LYGSEAFATDFQDSAAAK<br>AVLDVFEEGTEASAATAVK<br>AKWEMPFDPQDTHQSR<br>RLYGSEAFATDFQDSAAAK<br>DLDSQTMMVLVNYIFFK<br>GTHVDLGLASANVDFAFSLYK<br>DYNLNDILLQLGIEEAFTSK<br>FNRPFLMIIVPTDTQNIFFMSK
6	296	1.79	Leucine-rich alpha-2-glycoprotein	38.15	637	592	10	GPLQLER<br>DCQVFR<br>VAAGAFQGLR<br>ALGHLDLSGNR<br>DLLLPQPDLR<br>ENQLEVLEVSWLHGLK<br>TLDLGENQLETLPPDLLR<br>NALTGLPPGLFQASATLDTLVLK<br>DGFDISGNPWICDQNLSDLYR<br>LQELHLSSNGLESLSPEFLRPVPQLR
7	1085	-1.92	Transthyretin(Prealbumin)	15.9	88	76	3	GSPAINVAVHVFR<br>ALGISPFHEHAEVVFTANDSGPR<br>TSESGELHGLTTEEEFVEGIYK

8	729	-1.93	Haptoglobin	45.17	586	511	14	DYAEVGR<br>GSFPWQAK<br>VGYVSGWGR<br>VMPICLPSK<br>VTSIQDWVQK<br>DIAPTLTLYVGK<br>SCAVAEYGVYVK<br>YVMLPVADQDQCIR<br>AVGDKLPECEAVCGKPK<br>VMPICLPSKDYAEVGR<br>SPVGVQPILNEHTFCAGMSK<br>LPECEAVCGKPKNPANPVQR<br>FTDHLKYVMLPVADQDQCIR<br>YQEDTCYGDAGSAFAVH
9	626	-2.08	Zinc-alpha-2-glycoprotein	34.46	167	124	15	YYYDGK<br>IDVHWTR<br>DYIEFNK<br>YSKNILDR<br>AGEVQEPELR<br>AGEVQEPELR<br>CLAYDFYPGK<br>WEAEPVYVQR<br>WEAEPVYVQR<br>YSLTYIYTGLSK<br>AYLEEECPATLR<br>AYLEEECPATLR<br>WEAEPVYVQRAK<br>QKWEAEPVYVQR<br>QDPPSVVVTSHQAPGEK
10	684	-2.1	Haptoglobin	45.17	713	649	14	DYAEVGR<br>GSFPWQAK<br>VGYVSGWGR<br>VTSIQDWVQK<br>DIAPTLTLYVGK<br>TEGDGVYTLNDK<br>SCAVAEYGVYVK<br>YVMLPVADQDQCIR<br>VSVNERVMPICLPSK<br>VVLHPNYSQVDIGLIK<br>VMPICLPSKDYAEVGR<br>SPVGVQPILNEHTFCAGMSK<br>YVMLPVADQDQCIRHYEGSTVPEK<br>*YQEDTCYGDAGSAFAVHDLEEDTWYA<br>TGILSFDK*
11	523	-2.13	Alpha-1-antitrypsin	46.7	152	84	14	AVLTIDEK<br>FLENEDR<br>QINDYVEK<br>RSASLHLPK<br>SVLGQLGITK<br>LSSWVLLMK<br>FLENEDRR<br>WERPFEVK<br>GKWERPFEVK<br>DTVFALVNYIFFK<br>LQHLENELTHDIITK<br>DTEEEDFHVDQVTTVK<br>ELDRDTVFALVNYIFFK<br>LYHSEAFTVNFGDTEEAKK

12	531	-2.13	Leucine-rich alpha-2-glycoprotein	38.15	675	621	11	GPLQLER<br>DCQVFR<br>VAAGAFQGLR<br>ALGHLDLSGNR<br>DLLLPQPDLR<br>LARVAAGAFQGLR<br>ENQLEVLEVSWLHGLK<br>TLDLGENQLETLPPDLLR<br>NALTGLPPGLFQASATLDTLVLK<br>DGFDISGNPWICDQNLSDLYR<br>LQELHLSSNGLESLSPEFLRPVPQLR
13	776	-2.29	Ficolin 3 (Collagen/fibrinogen domain-containing protein 3	32.9	515	463	10	VRMMLR<br>YGIDWASGR<br>QDGSVDFFR<br>TFAHYATFR<br>GEPGDPVNLLR<br>RQDGSVDFFR<br>LLGEVDHYQLALGK<br>ALPVFCDMDTEGGGWLVFQR<br>ELLSQGATLSGWYHLCLPEGR<br>*AGFGNQESEFWLGNENLHQLTLQGNW ELR*
14	1039	-2.63	Haptoglobin	45.86	118	98	10	NYYKLR<br>NYYKLR<br>TEGDGVYTLNDK<br>LPECEAVCGKPK<br>LPECEAVCGKPK<br>TEGDGVYTLNNEK<br>TEGDGVYTLNNEK<br>LRTEGDGVYTLNDK<br>YVMLPVADQDQCIR<br>AVGDKLPECEAVCGKPK
15	524	-3.09	DNA2-like helicase	1220<br>47.8	64	15	24	ESNSIEHR<br>ALGMSESLFK<br>NQMAFSLFHR<br>NQMAFSLFHR<br>MNSKIMSLSNK<br>NQMAFSLFHR<br>NQMAFSLFHR<br>NQMAFSLFHR<br>GLNKPQRQAMK<br>DYTLIVGMPGTGK<br>VANAVINLRHFK<br>NQMAFSLFHRISK<br>SAVVQLTVQYRMNSK<br>TGQMYPVPANHLDKR<br>TGQMYPVPANHLDKR<br>LECGSDKVANAVINLR<br>LECGSDKVANAVINLR<br>IVCDGQYLHNFQCK<br>SIGMVEVNTVDKYQGR<br>SIGMVEVNTVDKYQGR<br>SIGMVEVNTVDKYQGR<br>SFWEEAELPAELFQK<br>VHPAIQQFTEQEICR<br>SFWEEAELPAELFQKK

16	726	-3.65	Haptoglobin	45.86	253	158	18	DYAEVGR<br>DYAEVGR<br>NYYKLR<br>QLVEIEK<br>GSFPWQAK<br>GSFPWQAK<br>VGYVSGWGR<br>VGYVSGWGR<br>VMPICLPSK<br>HYEGSTVPEK<br>VTSIQDWVQK<br>DIAPTLTLYVGK<br>SCAVAEYGVYVK<br>SCAVAEYGVYVK<br>YVMLPVADQDQCIR<br>YVMLPVADQDQCIR<br>YVMLPVADQDQCIR<br>VVLHPNYSQVDIGLIK
17	394	-3.74	Hemopexin	52.38	280	250	15	LHIMAGR<br>VWVYPPEK<br>VWVYPPEK<br>GGYTLVSGYPK<br>DYFMPCPGR<br>DYFMPCPGR<br>DYFMPCPGR<br>NFPSPVDAAFR<br>NFPSPVDAAFR<br>FDPVRGEVPPR<br>FDPVRGEVPPR<br>YYCFQGNQFLR<br>GECQAEGVLFFQGDR<br>GECQAEGVLFFQGDR<br>EWFWDLATGTMKER
18	1028	-4.15	Haptoglobin	4586<br>0.8	357	326	13	NPANPVQR<br>TEGDGVYTLNDK<br>TEGDGVYTLNDK<br>LPECEAVCGKPK<br>LPECEAVCGKPK<br>TEGDGVYTLNNEK<br>TEGDGVYTLNNEK<br>NLFLNHSENATAK<br>LRTEGDGVYTLNDK<br>YVMLPVADQDQCIR<br>LRTEGDGVYTLNNEK<br>AVGDKLPECEAVCGKPK<br>AVGDKLPECEAVCGKPK
19	676	-4.32	Haptoglobin	45.17	248	198	12	NYYKLR<br>NPANPVQR<br>VMPICLPSK<br>YQCKNYYK<br>LPECEAVCGKPK<br>TEGDGVYTLNDKK<br>LRTEGDGVYTLNDK<br>YVMLPVADQDQCIR<br>LRTEGDGVYTLNNEK<br>AVGDKLPECEAVCGKPK<br>LPECEADDGCPKPPEIAHGYVEHSVR<br>*AVGDKLPECEADDGCPKPPEIAHGYVE<br>HSVR*

20	349	-6.02	Alpha-1-antitrypsin(Alpha-1-protease inhibitor)	46.7	243	84	24	SASLHLPK<br>RSASLHLPK<br>SVLGQLGITK<br>LSSWVLLMK<br>FLENEDRR<br>LSSWVLLMK<br>LSITGTYDLK<br>KLSSWVLLMK<br>LGMFNIQHCK<br>GKWERPFEVK<br>RLGMFNIQHCK<br>LGMFNIQHCKK<br>RLGMFNIQHCK<br>QINDYVEKGTQGK<br>ITPNLAEFAFSLYR<br>VFSNGADLSGVTEEAPLK<br>FNKPFVFLMIEQNTK<br>ELDRDTVFALVNYIFFK<br>LSITGTYDLKSVLGQLGITK<br>VFSNGADLSGVTEEAPLKLSK<br>KLYHSEAFTVNFGDTEEAK<br>LYHSEAFTVNFGDTEEAKK<br>TLNQPDSQLQLTTGNGLFLSEGLK<br>*TDTSHHDQDHPTFNKITPNLAEFAFSLY R*
21	688	-6.36	Haptoglobin	4586 0.8	154	133	11	DYAEVGR<br>DYAEVGR<br>NYYKLR<br>GSFPWQAK<br>GSFPWQAK<br>VGYVSGWGR<br>VGYVSGWGR<br>VTSIQDWVQK<br>SCAVAEYGVYVK<br>SCAVAEYGVYVK<br>YVMLPVADQDQCIR
22	687	-7.73	Serum albumin	69.3	674	619	23	YLYEIAR<br>FQNALLVR<br>QTALVELVK<br>CASLQKFGER<br>ECCEKPLLEK<br>HPDYSVVLLLR<br>AVMDDFAAFVEK<br>RHPDYSVVLLLR<br>CCAAADPHECYAK<br>VPQVSTPTLVEVSR<br>DVFLGMFLYEYAR<br>YICENQDSISSKLK<br>KVPQVSTPTLVEVSR<br>QNCELFEQLGEYK<br>QEPERNECFLQHK<br>RPCFSALEVDETYVPK<br>VPQVSTPTLVEVSRNLGK<br>SLHTLFGDKLCTVATLR<br>AAFTECCQAADKAACLLPK<br>VFDEFKPLVEEPQNLIK<br>EFNAETFTFHADICTLSEK<br>MPCAEDYLSVVLNQLCVLHEK<br>RMPCAEDYLSVVLNQLCVLHEK

Table S6B. Differentially abundant serum proteins in DEF stage of the infection compared to HC subjects identified in 2D-DIGE combined with MALDI-TOF/TOF

S. No.	UniProtKB accession number	Protein name	No. of Spot	Fold-change (DEF/HC)
1	P01011	Alpha-1-antichymotrypsin	3	(3.01 to 3.16)
2	P01009	Alpha-1-antitrypsin	4	(1.7 to 4.29)
3	P02647	Apolipoprotein A-I	1	-3.04
4	P08603	Complement factor H	2	(2.67 to 2.87)
5	P51530	DNA2-like helicase	1	4.41
6	P00738	Haptoglobin	9	(-1.57 to -3)
7	P02790	Hemopexin	1	2.26
8	P01871	Ig mu chain C region	1	3.56
9	P04004	Vitronectin	1	4.75

Peptide details

S. No.	Gel ID	Fold-change	Protein name	Mw	Protein score	Total ion score	Matched peptides	Peptide sequence
1	501	4.75	Vitronectin(serum spreading factor)	54.27	184	164	7	GRNQNSR<br>QPQFISR<br>AVRPGYPK<br>NGSLFAFR<br>GQYCYELDEK<br>FEDGVLDPDYPR<br>DWHGVPGQVDAAMAGR
2	786	4.41	DNA2-like helicase	12204<br>7.8	58	18	22	ELCILR<br>IDVTVGVK<br>KVVASFPR<br>HLKEMYR<br>ALGMSESLFK<br>MNSKIMSLSNK<br>NQMAFSLFHR<br>NQMAFSLFHR<br>NQMAFSLFHR<br>DYTLIVGMPGTGK<br>VANAVINLRHFK<br>QMLIGTVLHEVFQK<br>LECGSDKVANAVINLR<br>LECGSDKVANAVINLR<br>SIGMVEVNTVDKYQGR<br>SFWEEAELPAELFQK<br>VHPAIQQFTEQEICR<br>VPAPEQVEKGGVSNVTEAK<br>SFWEEAELPAELFQKK<br>SFWEEAELPAELFQKK<br>KNHQNIWLMPASEMEK<br>SSDPATRQMLIGTVLHEVFQK

3	498	4.29	Alpha-1-antitrypsin(Alpha-1-protease inhibitor)	46.7	505	383	20	SVLGQLGITK<br>LSSWVLLMK<br>FLENEDRR<br>WERPFEVK<br>KLSSWVLLMK<br>LGMFNIQHCK<br>GKWERPFEVK<br>RLGMFNIQHCK<br>DTVFALVNYIFFK<br>ITPNLAEFAFSLYR<br>TDTSHHDQDHPTFNK<br>LQHLENELTHDIITK<br>FNKPFVFLMIEQNTK<br>DTEEEDFHVDQVTTVK<br>LYHSEAFTVNFGDTEEAK<br>ELDRDTVFALVNYIFFK<br>LYHSEAFTVNFGDTEEAKK<br>GTEAAGAMFLEAIPMSIPPEVK<br>TLNQPDSQLQLTTGNGLFLSEGLK<br>LQHLENELTHDIITKFLENEDR
4	325	3.56	Ig mu chain C region	49.52	522	484	15	VSVFVPPR<br>DGFFGNPR<br>QIQVSWLR<br>DGFFGNPRSK<br>LICQATGFSPR<br>NVPLPVIAELPPK<br>YVTSAPMPEPQAPGR<br>VFAIPPSFASIFLTK<br>ESDWLSQSMFTCR<br>FTCTVTHTDLPSPLK<br>GVALHRPDVYLLPPAR<br>ESDWLSQSMFTCRVDHR<br>GLTFQQNASSMCVPDQDTAIR<br>YAATSQVLLPSKDVMQGTDEHVVCK<br>*YFAHSILTVSEEEWNTGETYTCVVAHEA LPNR*
5	494	3.32	Alpha-1-antitrypsin(Alpha-1-protease inhibitor)	46.7	243	84	24	SASLHLPK<br>RSASLHLPK<br>SVLGQLGITK<br>LSSWVLLMK<br>FLENEDRR<br>LSSWVLLMK<br>LSITGTYDLK<br>KLSSWVLLMK<br>LGMFNIQHCK<br>GKWERPFEVK<br>RLGMFNIQHCK<br>LGMFNIQHCKK<br>RLGMFNIQHCK<br>QINDYVEKGTQGK<br>ITPNLAEFAFSLYR<br>VFSNGADLSGVTEEAPLK<br>FNKPFVFLMIEQNTK<br>ELDRDTVFALVNYIFFK<br>LSITGTYDLKSVLGQLGITK<br>VFSNGADLSGVTEEAPLKLSK<br>KLYHSEAFTVNFGDTEEAK<br>LYHSEAFTVNFGDTEEAKK<br>TLNQPDSQLQLTTGNGLFLSEGLK<br>TDTSHHDQDHPTFNKITPNLAEFAFSLYR

6	480	3.16	Alpha-1-antichymotrypsin	47.62	224	74	21	LINDYVK<br>ADLSGITGAR<br>EQLSLLDR<br>KLINDYVK<br>EIGELYLPK<br>NLAVSQVVHK<br>WRDSLEFR<br>ITLLSALVETR<br>EQLSLLDRFTEDAK<br>WEMPFDPQDTHQSR<br>MEEVEAMLLPETLKR<br>DSLEFREIGELYLPK<br>LYGSEAFATDFQDSAAAK<br>AVLDVFEEGTEASAATAVK<br>AKWEMPFDPQDTHQSR<br>RLYGSEAFATDFQDSAAAK<br>DLDSQTMMVLVNYIFFK<br>GTHVDLGLASANVDFAFSLYK<br>WVMVPMMSLHHLTIPYFR<br>DYNLNDILLQLGIEEAFTSK<br>FNRPFLMIIVPTDTQNIFFMSK
7	452	3.02	Alpha-1-antichymotrypsin	47.62	258	182	15	ADLSGITGAR<br>EQLSLLDR<br>EIGELYLPK<br>WRDSLEFR<br>ITLLSALVETR<br>WEMPFDPQDTHQSR<br>MEEVEAMLLPETLKR<br>LYGSEAFATDFQDSAAAK<br>AVLDVFEEGTEASAATAVK<br>AKWEMPFDPQDTHQSR<br>RLYGSEAFATDFQDSAAAK<br>DLDSQTMMVLVNYIFFK<br>GTHVDLGLASANVDFAFSLYK<br>DYNLNDILLQLGIEEAFTSK<br>FNRPFLMIIVPTDTQNIFFMSK
8	440	3.01	Alpha-1-antichymotrypsin	47.62	284	130	20	ADLSGITGAR<br>EQLSLLDR<br>KLINDYVK<br>EIGELYLPK<br>NLAVSQVVHK<br>WRDSLEFR<br>ITLLSALVETR<br>EQLSLLDRFTEDAK<br>WEMPFDPQDTHQSR<br>MEEVEAMLLPETLKR<br>LYGSEAFATDFQDSAAAK<br>AVLDVFEEGTEASAATAVK<br>AKWEMPFDPQDTHQSR<br>LYGSEAFATDFQDSAAAKK<br>RLYGSEAFATDFQDSAAAK<br>DLDSQTMMVLVNYIFFK<br>GTHVDLGLASANVDFAFSLYK<br>WVMVPMMSLHHLTIPYFR<br>YNLNDILLQLGIEEAFTSK<br>FNRPFLMIIVPTDTQNIFFMSK

9	164	2.87	Complement factor H precursor	139.0<br>3	701	494	440	CRPGYR<br>NGFYPATR<br>WTGRPTCR<br>NGQWSEPPK<br>IDVHLVPDR<br>IIYKENER<br>WSHPPSCIK<br>EFDHNSNIR<br>RPYFPVAVGK<br>CLHPCVISR<br>SLGNVIMVCR<br>GEWVALNPLR<br>SLGNVIMVCR<br>LSYTCEGGFR<br>HGGLYHENMR<br>TGDEITYQCR<br>CTSTGWIPAPR<br>WQSIPLCVEK<br>DGWSAQPTCIK<br>GEWVALNPLRK<br>CNMGYEYSER<br>SCDIPVFMNAR<br>TGESVEFVCKR<br>SCDIPVFMNAR<br>EIMENYNIALR<br>EGWIHTVCINGR<br>SIDVACHPGYALPK<br>WSSPPQCEGLPCK<br>AGEQVTYTCATYYK<br>EIMENYNIALRWTAK<br>SCDNPYIPNGDYSPLR<br>AQTTVTCMENGWSPTPR<br>EQVQSCGPPPELLNGNVK<br>DTSCVNPPTVQNAYIVSR<br>IPCSQPPQIEHGTINSSR<br>IEGDEEMHCSDDGFWSK<br>AVYTCNEGYQLLGEINYR<br>GDAVCTESGWRPLPSCEEK<br>IVSSAMEPDREYHFGQAVR<br>TKEEYGHSEVVEYYCNPR

10	163	2.67	Complement factor H precursor	143.71	778	572	40	CRPGYR<br>NGFYPATR<br>WTGRPTCR<br>IDVHLVPDR<br>EYHFGQAVR<br>WSHPPSCIK<br>EFDHNSNIR<br>RPYFPVAVGK<br>CLHPCVISR<br>SLGNVIMVCR<br>GEWVALNPLR<br>LSYTCEGGFR<br>HGGLYHENMR<br>TGDEITYQCR<br>CTSTGWIPAPR<br>WQSIPLCVEK<br>KEFDHNSNIR<br>DGWSAQPTCIK<br>GEWVALNPLRK<br>CNMGYEYSER<br>SCDIPVFMNAR<br>CNMGYEYSER<br>SCDIPVFMNAR<br>EIMENYNIALR<br>SSNLIILEEHLK<br>SIDVACHPGYALPK<br>WSSPPQCEGLPCK<br>AGEQVTYTCATYYK<br>EIMENYNIALRWTAK<br>SCDNPYIPNGDYSPLR<br>EIMENYNIALRWTAK<br>AQTTVTCMENGWSPTPR<br>EQVQSCGPPPELLNGNVK<br>DTSCVNPPTVQNAYIVSR<br>IPCSQPPQIEHGTINSSR<br>IEGDEEMHCSDDGFWSK<br>AVYTCNEGYQLLGEINYR<br>GDAVCTESGWRPLPSCEEK<br>IVSSAMEPDREYHFGQAVR<br>TKEEYGHSEVVEYYCNPR
11	345	2.26	Hemopexin precursor	51.64	182	136	14	LHIMAGR<br>LWWLDLK<br>VWVYPPEK<br>RLWWLDLK<br>DYFMPCPGR<br>WDRELISER<br>NFPSPVDAAFR<br>FDPVRGEVPPR<br>YYCFQGNQFLR<br>GECQAEGVLFFQGDR<br>LYLVQGTQVYVFLTK<br>SGAQATWTELPWPHEK<br>LLQDEFPGIPSPLDAAVECHR<br>EVGTPHGIILDSVDAAFICPGSSR

12	491	1.76	Alpha-1-antitrypsin	46.87	381	283	23	FLENEDR<br>QINDYVEK<br>SVLGQLGITK<br>FLENEDRR<br>FLENEDRR<br>WERPFEVK<br>WERPFEVK<br>LSITGTYDLK<br>LGMFNIQHCK<br>GKWERPFEVK<br>GKWERPFEVK<br>ITPNLAEFAFSLYR<br>TDTSHHDQDHPTFNK<br>LQHLENELTHDIITK<br>VFSNGADLSGVTEEAPLK<br>VFSNGADLSGVTEEAPLK<br>DTEEEDFHVDQVTTVK<br>DTEEEDFHVDQVTTVK<br>LYHSEAFTVNFGDTEEAK<br>VFSNGADLSGVTEEAPLKLSK<br>LYHSEAFTVNFGDTEEAKK<br>LYHSEAFTVNFGDTEEAKK<br>TLNQPDSQLQLTTGNGLFLSEGLK
13	502	1.7	Alpha-1-antitrypsin	46878<br>.1	528	402	28	SASLHLPK<br>FLEDVKK<br>AVLTIDEK<br>FLENEDR<br>QINDYVEK<br>SVLGQLGITK<br>LSSWVLLMK<br>FLENEDRR<br>FLENEDRR<br>WERPFEVK<br>WERPFEVK<br>LSITGTYDLK<br>LGMFNIQHCK<br>LGMFNIQHCK<br>LGMFNIQHCK<br>GKWERPFEVK<br>LGMFNIQHCKK<br>TDTSHHDQDHPTFNK<br>TDTSHHDQDHPTFNK<br>LQHLENELTHDIITK<br>VFSNGADLSGVTEEAPLK<br>VFSNGADLSGVTEEAPLK<br>VFSNGADLSGVTEEAPLK<br>DTEEEDFHVDQVTTVK<br>DTEEEDFHVDQVTTVK<br>LYHSEAFTVNFGDTEEAK<br>LYHSEAFTVNFGDTEEAKK<br>TLNQPDSQLQLTTGNGLFLSEGLK

14	716	-1.57	Haptoglobin	45.86	253	158	18	DYAEVGR<br>DYAEVGR<br>NYYKLR<br>QLVEIEK<br>GSFPWQAK<br>GSFPWQAK<br>VGYVSGWGR<br>VGYVSGWGR<br>VMPICLPSK<br>HYEGSTVPEK<br>VTSIQDWVQK<br>DIAPTLTLYVGK<br>SCAVAEYGVYVK<br>SCAVAEYGVYVK<br>YVMLPVADQDQCIR<br>YVMLPVADQDQCIR<br>YVMLPVADQDQCIR<br>VVLHPNYSQVDIGLIK
15	662	-1.6	Haptoglobin	45.17	713	649	14	DYAEVGR<br>GSFPWQAK<br>VGYVSGWGR<br>VTSIQDWVQK<br>DIAPTLTLYVGK<br>TEGDGVYTLNDK<br>SCAVAEYGVYVK<br>YVMLPVADQDQCIR<br>VSVNERVMPICLPSK<br>VVLHPNYSQVDIGLIK<br>VMPICLPSKDYAEVGR<br>SPVGVQPILNEHTFCAGMSK<br>YVMLPVADQDQCIRHYEGSTVPEK<br>*YQEDTCYGDAGSAFAVHDLEEDTWYA<br>TGILSFDK*
16	641	-1.65	Haptoglobin	45.17	239	192	11	DYAEVGR<br>GSFPWQAK<br>ILGGHLDAK<br>VGYVSGWGR<br>VTSIQDWVQK<br>SCAVAEYGVYVK<br>YVMLPVADQDQCIR<br>VVLHPNYSQVDIGLIK<br>SPVGVQPILNEHTFCAGMSK<br>SCAVAEYGVYVKVTSIQDWVQK<br>*YQEDTCYGDAGSAFAVHDLEEDTWYA<br>TGILSFDK*
17	960	-1.7	Haptoglobin	45.86	118	98	10	NYYKLR<br>NYYKLR<br>TEGDGVYTLNDK<br>LPECEAVCGKPK<br>LPECEAVCGKPK<br>TEGDGVYTLNNEK<br>TEGDGVYTLNNEK<br>LRTEGDGVYTLNDK<br>YVMLPVADQDQCIR<br>AVGDKLPECEAVCGKPK

18	967	-1.72	Haptoglobin	45.86	357	326	13	NPANPVQR<br>TEGDGVYTLNDK<br>TEGDGVYTLNDK<br>LPECEAVCGKPK<br>LPECEAVCGKPK<br>TEGDGVYTLNNEK<br>TEGDGVYTLNNEK<br>NLFLNHSENATAK<br>LRTEGDGVYTLNDK<br>YVMLPVADQDQCIR<br>LRTEGDGVYTLNNEK<br>AVGDKLPECEAVCGKPK<br>AVGDKLPECEAVCGKPK
19	632	-1.8	Haptoglobin	34.46	167	124	15	YYYDGK<br>IDVHWTR<br>DYIEFNK<br>YSKNILDR<br>AGEVQEPELR<br>AGEVQEPELR<br>CLAYDFYPGK<br>WEAEPVYVQR<br>WEAEPVYVQR<br>YSLTYIYTGLSK<br>AYLEEECPATLR<br>AYLEEECPATLR<br>WEAEPVYVQRAK<br>QKWEAEPVYVQR<br>QDPPSVVVTSHQAPGEK
20	668	-1.82	Haptoglobin	45.86	237	191	16	DYAEVGR<br>DYAEVGR<br>GSFPWQAK<br>ILGGHLDAK<br>VGYVSGWGR<br>VGYVSGWGR<br>VMPICLPSK<br>HYEGSTVPEK<br>VTSIQDWVQK<br>SCAVAEYGVYVK<br>YVMLPVADQDQCIR<br>YVMLPVADQDQCIR<br>YVMLPVADQDQCIR<br>VMPICLPSKDYAEVGR<br>SPVGVQPILNEHTFCAGMSK<br>SPVGVQPILNEHTFCAGMSK
21	671	-2.32	Haptoglobin	45860<br>.8	203	176	13	DYAEVGR<br>DYAEVGR<br>QLVEIEK<br>GSFPWQAK<br>GSFPWQAK<br>VGYVSGWGR<br>VGYVSGWGR<br>HYEGSTVPEK<br>VTSIQDWVQK<br>SCAVAEYGVYVK<br>SCAVAEYGVYVK<br>YVMLPVADQDQCIR<br>YVMLPVADQDQCIR

22	679	-3	Haptoglobin	45860<br>.8	154	133	11	DYAEVGR<br>DYAEVGR<br>NYYKLR<br>GSFPWQAK<br>GSFPWQAK<br>VGYVSGWGR<br>VGYVSGWGR<br>VTSIQDWVQK<br>SCAVAEYGVYVK<br>SCAVAEYGVYVK<br>YVMLPVADQDQCIR
23	905	-3.04	Apolipoprotein A-I	30.75	486	351	20	QKVEPLR<br>AELQEGAR<br>LHELQEK<br>AKPALEDLR<br>LSPLGEEMR<br>QKLHELQEK<br>QGLLPVLESFK<br>DLATVYVDVLK<br>VQPYLDDFQK<br>WQEEMELYR<br>THLAPYSDELR<br>LSPLGEEMRDR<br>VQPYLDDFQKK<br>VSFLSALEEYTK<br>DYVSQFEGSALGK<br>KWQEEMELYR<br>VEPLRAELQEGAR<br>LLDNWDSVTSTFSK<br>DSGRDYVSQFEGSALGK<br>LREQLGPVTQEFWDNLEK

Table S6C. Differentially abundant serum proteins in CON stage of the infection compared to HC subjects identified in 2D-DIGE combined with MALDI-TOF/TOF

S. No.	UniProtKB accession number	Protein name	No. of Spot	Fold-change (CON/HC)
1	P01011	Alpha-1-antichymotrypsin	1	3.16
2	P01871	Ig mu chain C region	1	3.56
3	P01009	Alpha-1-antitrypsin	1	3.32
4	P00738	Haptoglobin	1	-1.57

Peptide details

S. No.	Gel ID	Fold-change	Protein name	Mw	Protein score	Total ion score	Matched peptides	Peptide sequence
1	93	3.16	Alpha-1-antichymotrypsin	47.62	224	74	21	LINDYVK<br>ADLSGITGAR<br>EQLSLLDR<br>KLINDYVK<br>EIGELYLPK<br>NLAVSQVVHK<br>WRDSLEFR<br>ITLLSALVETR<br>EQLSLLDRFTEDAK<br>WEMPFDPQDTHQSR<br>MEEVEAMLLPETLKR<br>DSLEFREIGELYLPK<br>LYGSEAFATDFQDSAAAK<br>AVLDVFEEGTEASAATAVK<br>AKWEMPFDPQDTHQSR<br>RLYGSEAFATDFQDSAAAK<br>DLDSQTMMVLVNYIFFK<br>GTHVDLGLASANVDFAFSLYK<br>WVMVPMMSLHHLTIPYFR<br>DYNLNDILLQLGIEEAFTSK<br>FNRPFLMIIVPTDTQNIFFMSK
2	179	3.56	Ig mu chain C region	49.52	522	484	15	VSVFVPPR<br>DGFFGNPR<br>QIQVSWLR<br>DGFFGNPRSK<br>LICQATGFSPR<br>NVPLPVIAELPPK<br>YVTSAPMPEPQAPGR<br>VFAIPPSFASIFLTK<br>ESDWLSQSMFTCR<br>FTCTVTHTDLPSPLK<br>GVALHRPDVYLLPPAR<br>ESDWLSQSMFTCRVDHR<br>GLTFQQNASSMCVPDQDTAIR<br>YAATSQVLLPSKDVMQGTDEHVVCK<br>*YFAHSILTVSEEEWNTGETYTCVVA<br>HEALPNR*

3	6	3.32	Alpha-1-antitrypsin(Alpha-1-protease inhibitor)	46.7	243	84	24	SASLHLPK<br>RSASLHLPK<br>SVLGQLGITK<br>LSSWVLLMK<br>FLENEDRR<br>LSSWVLLMK<br>LSITGTYDLK<br>KLSSWVLLMK<br>LGMFNIQHCK<br>GKWERPFEVK<br>RLGMFNIQHCK<br>LGMFNIQHCKK<br>RLGMFNIQHCK<br>QINDYVEKGTQGK<br>ITPNLAEFAFSLYR<br>VFSNGADLSGVTEEAPLK<br>FNKPFVFLMIEQNTK<br>ELDRDTVFALVNYIFFK<br>LSITGTYDLKSVLGQLGITK<br>VFSNGADLSGVTEEAPLKLSK<br>KLYHSEAFTVNFGDTEEAK<br>LYHSEAFTVNFGDTEEAKK<br>TLNQPDSQLQLTTGNGLFLSEGLK<br>TDTSHHDQDHPTFNKITPNLAEFAFSLYR
4	996	-1.57	Haptoglobin	45.86	253	158	18	DYAEVGR<br>DYAEVGR<br>NYYKLR<br>QLVEIEK<br>GSFPWQAK<br>GSFPWQAK<br>VGYVSGWGR<br>VGYVSGWGR<br>VMPICLPSK<br>HYEGSTVPEK<br>VTSIQDWVQK<br>DIAPTLTLYVGK<br>SCAVAEYGVYVK<br>SCAVAEYGVYVK<br>YVMLPVADQDQCIR<br>YVMLPVADQDQCIR<br>YVMLPVADQDQCIR<br>VVLHPNYSQVDIGLIK

Table S7A. Complete details of protein identification and quantitative iTRAQ data for the longitudinal cohort of vivax malaria patients (compared to HC) obtained from Q-TOF analysis

(I) Replicate 1

S. No.	Num Peps Unique	Fold-change FEB/HC	Fold-change DEF/HC	Fold-change CON/HC	Accession number	Protein name
1	98	1.11	0.643	0.638	P04114	Apolipoprotein B-100
2	85	1.073	0.751	0.694	P01024	Complement C3
3	57	1.099	0.773	0.724	P01023	Alpha-2-macroglobulin
4	45	1.151	0.755	0.744	P0C0L4	Complement C4-A
5	36	1.805	1.057	1.078	P01009	Alpha-1-antitrypsin
6	24	1.07	0.694	0.622	P02787	Serotransferrin
7	23	1.882	1.131	1.152	P01011	Alpha-1-antichymotrypsin
8	23	1.026	1.136	0.792	P02768	Serum albumin
9	22	1.35	0.808	0.763	Q14624	Inter-alpha-trypsin inhibitor heavy chain H4
10	20	1.825	1.261	0.806	P00450	Ceruloplasmin
11	19	1.108	0.704	0.997	P02751	Fibronectin
12	18	1.631	1.381	0.909	P02790	Hemopexin
13	17	0.409	0.723	0.763	P00738	Haptoglobin
14	11	0.792	0.69	0.822	P00739	Haptoglobin-related protein
15	17	1.577	0.875	0.908	P00751	Complement factor B
16	16	0.547	0.517	0.714	P02647	Apolipoprotein A-I
17	14	1.465	1.022	0.984	P01871	Ig mu chain C region
18	14	1.416	0.678	0.727	P01008	Antithrombin-III
19	14	0.903	0.669	0.556	P19823	Inter-alpha-trypsin inhibitor heavy chain H2
20	13	1.415	0.861	0.906	P05155	Plasma protease C1 inhibitor
21	11	1.02	0.751	0.546	P04196	Histidine-rich glycoprotein
22	11	1.193	0.782	0.868	P04217	Alpha-1B-glycoprotein
23	10	0.958	0.597	0.724	P19827	Inter-alpha-trypsin inhibitor heavy chain H1
24	9	1.193	0.797	0.847	P01019	Angiotensinogen
25	9	1.309	0.758	0.847	P01876	Ig alpha-1 chain C region
26	8	1.307	0.764	0.838	P01877	Ig alpha-2 chain C region
27	9	2.289	0.953	0.876	P68871	Hemoglobin subunit beta
28	9	1.286	0.773	0.85	P02774	Vitamin D-binding protein
29	8	2.537	1.51	0.994	P02750	Leucine-rich alpha-2-glycoprotein
30	8	1.025	0.613	0.617	P08603	Complement factor H
31	8	1.458	0.615	0.765	P27169	Serum paraoxonase/arylesterase 1
32	7	2.263	1.318	1.421	P02763	Alpha-1-acid glycoprotein 1
33	7	1.952	1.177	1.072	P19652	Alpha-1-acid glycoprotein 2

34	7	2.532	0.839	0.955	P69905	Hemoglobin subunit alpha
35	7	1.38	0.791	0.867	P01031	Complement C5
36	7	1.088	0.695	0.66	P25311	Zinc-alpha-2-glycoprotein
37	7	1.193	0.916	0.903	P36955	Pigment epithelium-derived factor
38	7	1.028	0.78	0.616	P06396	Gelsolin
39	7	0.885	0.548	0.637	P43652	Afamin
40	7	0.88	0.453	0.556	P06727	Apolipoprotein A-IV
41	7	1.264	0.828	0.857	P01042	Kininogen-1
42	6	1.445	0.889	0.798	P04004	Vitronectin
43	6	0.943	0.555	0.509	P05546	Heparin cofactor 2
44	6	1.013	0.69	0.814	P00734	Prothrombin
45	6	3.102	4.945	1.975	P02741	C-reactive protein
46	5	0.999	0.647	0.631	P02766	Transthyretin
47	5	1.064	0.668	0.714	P10909	Clusterin
48	5	1.083	0.763	0.646	P02765	Alpha-2-HS-glycoprotein
49	5	0.991	0.631	0.592	Q96PD5	N-acetylmuramoyl-L-alanine amidase
50	5	0.554	0.537	0.542	P02652	Apolipoprotein A-II
51	5	1.637	1.261	0.927	P02649	Apolipoprotein E
52	4	1.495	1.426	0.954	P01857	Ig gamma-1 chain C region
53	4	1.291	0.873	0.812	P01834	Ig kappa chain C region
54	4	1.157	0.807	0.811	B9A064	Immunoglobulin lambda-like polypeptide 5
55	3	1.224	0.899	0.847	P0CG06	Ig lambda-3 chain C regions
56	4	1.466	0.904	1.226	P08697	Alpha-2-antiplasmin
57	4	1.358	0.758	0.866	P00736	Complement C1r subcomponent
58	2	1.397	0.732	0.866	Q9NZP8	Complement C1r subcomponent-like protein
59	4	1.282	0.926	0.959	P08185	Corticosteroid-binding globulin
60	3	1.594	0.912	1.061	P02748	Complement component C9
61	3	1.988	1.878	1.706	P35858	Insulin-like growth factor-binding protein complex acid labile subunit
62	3	1.225	0.689	0.982	P02760	Protein AMBP
63	3	1.728	0.774	0.754	P05156	Complement factor I
64	3	1.625	1.103	0.96	P04003	C4b-binding protein alpha chain
65	3	1.192	1.367	1.131	P04206	Ig kappa chain V-III region GOL
66	3	1.08	0.856	0.774	P51884	Lumican
67	3	1.133	0.597	0.735	P02743	Serum amyloid P-component
68	3	1.482	1.678	1.14	P01860	Ig gamma-3 chain C region
69	3	1.19	0.863	0.569	P01859	Ig gamma-2 chain C region
70	3	0.833	0.412	0.49	P02749	Beta-2-glycoprotein 1
71	3	1.589	0.595	0.867	P09871	Complement C1s subcomponent
72	3	1.289	0.984	0.656	P06681	Complement C2

73	3	1.937	2.53	1.247	Q06033	Inter-alpha-trypsin inhibitor heavy chain H3
74	3	0.619	0.941	0.637	Q8WZ42	Titin
75	2	1.43	0.783	0.971	P22792	Carboxypeptidase N subunit 2
76	2	1.279	0.884	1.254	P01625	Ig kappa chain V-IV region Len
77	2	1.3	1.089	0.83	P01594	Ig kappa chain V-I region AU
78	2	1.229	1.51	1.042	P00747	Plasminogen
79	2	1.259	1.6	1.614	P01766	Ig heavy chain V-III region BRO
80	2	1.552	0.898	0.953	Q9UNS1	Protein timeless homolog
81	2	0.917	0.593	0.623	P78332	RNA-binding protein 6
82	2	1.213	0.893	1.28	P02656	Apolipoprotein C-III
83	2	0.973	0.636	0.608	Q92797	Symplekin
84	2	1.104	1.229	0.401	P06310	Ig kappa chain V-II region RPMI 6410
85	2	2.639	0.83	1.101	P20929	Nebulin
86	2	1.407	0.997	1.188	P04207	Ig kappa chain V-III region CLL
87	2	1.416	0.517	0.635	P29622	Kallistatin
88	2	1.625	1.272	1.207	P05543	Thyroxine-binding globulin
89	2	1.804	0.968	0.918	P21359	Neurofibromin
90	2	0.71	1.305	0.212	P01774	Ig heavy chain V-III region POM
91	2	1.181	0.958	0.666	P01591	Immunoglobulin J chain
92	2	1.816	1	1	P18428	Lipopolysaccharide-binding protein
93	2	2.301	0.105	1	P04278	Sex hormone-binding globulin
94	2	1.056	1.146	0.919	O15050	TPR and ankyrin repeat-containing protein 1
95	2	2.934	1.884	1.451	Q96DT5	Dynein heavy chain 11, axonemal
96	2	1.197	0.549	0.71	Q96T23	Remodeling and spacing factor 1
97	2	2.072	1.223	1.053	Q96Q15	Serine/threonine-protein kinase SMG1
98	2	1.746	0.625	0.613	Q8N1T3	Unconventional myosin-Ih
99	2	1.974	1.307	1.17	Q9P2R6	Arginine-glutamic acid dipeptide repeats protein
100	2	0.524	0.514	0.403	Q12923	Tyrosine-protein phosphatase non-receptor type 13
101	2	0.828	1.203	1.227	P28289	Tropomodulin-1
102	2	1	1	1	P04275	von Willebrand factor
103	2	4.891	5.035	0.738	Q8N446	Zinc finger protein 843
104	2	1.527	0.682	0.86	P0C1S8	Wee1-like protein kinase 2
105	2	0.951	0.539	0.683	Q9Y646	Carboxypeptidase Q
106	1	0.538	0.467	0.799	Q13790	Apolipoprotein F
107	1	2.476	1	1.756	O14791	Apolipoprotein L1
108	1	1.866	1.397	0.911	P01598	Ig kappa chain V-I region EU
109	1	1.796	0.543	0.358	P07358	Complement component C8 beta chain

110	1	1.016	0.631	0.45	P02671	Fibrinogen alpha chain
111	1	3.766	1.136	0.794	P02655	Apolipoprotein C-II
112	1	1.495	1.385	0.913	Q9H777	Zinc phosphodiesterase ELAC protein 1
113	1	1.184	0.889	1.467	Q9UK55	Protein Z-dependent protease inhibitor
114	1	1	1	1	Q8N6C8	Leukocyte immunoglobulin-like receptor subfamily A member 3
115	1	0.798	0.799	0.531	P10643	Complement component C7
116	1	0.862	0.724	0.615	Q9ULR5	Polyadenylate-binding protein-interacting protein 2B
117	1	1.275	0.728	1.371	O43149	Zinc finger ZZ-type and EF-hand domain-containing protein 1
118	1	1.211	0.712	0.268	P13671	Complement component C6
119	1	0.515	0.763	0.512	P22352	Glutathione peroxidase 3
120	1	1.186	1.371	0.333	P01715	Ig lambda chain V-IV region Bau
121	1	0.229	0.349	0.182	Q8WWI5	Choline transporter-like protein 1
122	1	0.496	0.592	0.848	P01602	Ig kappa chain V-I region HK102
123	1	0.553	0.643	0.43	Q9Y305	Acyl-coenzyme A thioesterase 9, mitochondrial
124	1	1.271	1.058	0.256	Q8N264	Rho GTPase-activating protein 24
125	1	0.44	1	0.347	O75882	Attractin
126	1	0.523	0.588	1.705	P01717	Ig lambda chain V-IV region Hil
127	1	0.426	0.158	0.442	Q13469	Nuclear factor of activated T-cells, cytoplasmic 2
128	1	0.981	0.526	0.622	Q6PIF6	Unconventional myosin-VIIb
129	1	1.389	1.011	0.859	Q92615	La-related protein 4B
130	1	0.739	0.414	1.071	Q5T655	Coiled-coil domain-containing protein 147
131	1	1.86	0.977	1.08	Q6P6B1	Uncharacterized protein C8orf47
132	1	1.338	0.947	0.786	Q8WWT9	Solute carrier family 13 member 3
133	1	1.221	1.227	0.878	P08F94	Fibrocystin
134	1	1.299	1.117	0.851	P52737	Zinc finger protein 136
135	1	3.842	2.565	1.157	Q15678	Tyrosine-protein phosphatase non-receptor type 14
136	1	0.541	1.432	0.099	Q8WXH0	Nesprin-2
137	1	1	1	1	Q96KN2	Beta-Ala-His dipeptidase
138	1	0.932	0.463	0.467	Q9NPG3	Ubinuclein-1
139	1	2.155	2.178	2.61	Q03591	Complement factor H-related protein 1
140	1	0.375	1.216	1.465	A8K5M9	Uncharacterized protein C15orf62, mitochondrial
141	1	1.545	1.595	1.239	Q04912	Macrophage-stimulating protein receptor
142	1	1.513	1	1.871	A6NLA2	Putative glutaredoxin-like protein
143	1	2.006	1.587	0.979	Q9H0H0	Integrator complex subunit 2

144	1	1.241	0.671	0.584	Q9H013	Disintegrin and metalloproteinase domain-containing protein 19
145	1	0.947	1	0.491	P08571	Monocyte differentiation antigen CD14
146	1	3.156	0.608	0.785	Q15911	Zinc finger homeobox protein 3
147	1	1.081	0.734	0.849	P32245	Melanocortin receptor 4
148	1	1.747	1.251	2.449	Q6UWE0	E3 ubiquitin-protein ligase LRSAM1
149	1	0.925	0.223	0.65	P06737	Glycogen phosphorylase, liver form
150	1	1.54	1.023	1.149	Q53GS7	Nucleoporin GLE1
151	1	0.628	0.7	0.338	O60583	Cyclin-T2
152	1	1	1	1	Q9NR48	Unconventional myosin-VI
153	1	0.988	0.692	0.548	O15327	Type II inositol 3,4-bisphosphate 4-phosphatase
154	1	2.255	0.586	0.634	P28845	Corticosteroid 11-beta-dehydrogenase isozyme 1
155	1	3.945	1.246	0.737	P01700	Ig lambda chain V-I region HA
156	1	0.856	0.162	0.285	O75593	Forkhead box protein H1
157	1	0.972	1	2.768	Q9ULT0	Tetratricopeptide repeat protein 7A
158	1	1.526	1.033	1.013	Q5JTH9	RRP12-like protein
159	1	0.673	0.598	0.224	Q13601	KRR1 small subunit processome component homolog
160	1	1.703	0.146	1.419	Q6ZN30	Zinc finger protein basonuclin-2
161	1	1.25	0.653	0.776	Q5FWF5	N-acetyltransferase ESCO1
162	1	1.245	0.612	0.666	Q8N0S6	Centromere protein L
163	1	0.916	0.491	0.524	O75822	Eukaryotic translation initiation factor 3 subunit J
164	1	0.72	0.819	0.578	Q07617	Sperm-associated antigen 1
165	1	0.925	0.624	0.235	P05060	Secretogranin-1
166	1	0.87	0.519	0.272	Q6IPW1	Uncharacterized protein C11orf71
167	1	1.313	0.727	0.742	Q8TEQ0	Sorting nexin-29
168	1	0.609	0.405	1	Q9UI12	V-type proton ATPase subunit H
169	1	1.095	0.414	0.375	E7EW31	Proline-rich basic protein 1
170	1	2.12	1.821	1.537	Q96T37	Putative RNA-binding protein 15
171	1	2.19	0.39	0.676	P20851	C4b-binding protein beta chain
172	1	0.856	0.622	0.656	Q96JI7	Spatacsin
173	1	0.853	0.387	0.548	O95997	Securin
174	1	0.879	0.78	1.063	Q8TEU7	Rap guanine nucleotide exchange factor 6
175	1	1.358	0.841	0.518	Q6UW60	Proprotein convertase subtilisin/kexin type 4
176	1	2.803	1.151	1.989	Q96PN8	Testis-specific serine/threonine-protein kinase 3
177	1	1	1	1	P19878	Neutrophil cytosol factor 2
178	1	1	1	1	Q8WV99	AN1-type zinc finger protein 2B

179	1	2.362	1.941	1.229	Q96M27	Protein PRRC1
180	1	2.104	1.446	1.704	Q8WTP8	Apoptosis-enhancing nuclease
181	1	1	1	1	Q9UL17	T-box transcription factor TBX21
182	1	0.636	1.058	1.12	Q8IYT2	FtsJ methyltransferase domain-containing protein 1
183	1	0.938	0.689	0.775	O75717	WD repeat and HMG-box DNA-binding protein 1
184	1	0.613	0.715	0.358	Q5JWR5	Protein dopey-1
185	1	2.708	0.708	1.235	O75923	Dysferlin
186	1	1.186	0.953	1	Q8NDB2	B-cell scaffold protein with ankyrin repeats
187	1	0.841	0.578	0.407	O00144	Frizzled-9
188	1	0.554	1	1.096	P47224	Guanine nucleotide exchange factor MSS4
189	1	0.779	0.462	0.683	Q92888	Rho guanine nucleotide exchange factor 1
190	1	1	1.37	1	Q16740	Putative ATP-dependent Clp protease proteolytic subunit, mitochondrial
191	1	1	0.358	1	Q7Z408	CUB and sushi domain-containing protein 2
192	1	1.155	1	0.911	Q6SPF0	Atherin
193	1	1.229	0.811	0.269	P31947	14-3-3 protein sigma
194	1	1.775	0.469	0.467	Q9BX40	Protein LSM14 homolog B
195	1	1.69	0.841	0.937	P51580	Thiopurine S-methyltransferase
196	1	0.535	0.299	0.672	P00568	Adenylate kinase isoenzyme 1
197	1	1	0.65	2.342	Q96PM9	Zinc finger protein 385A
198	1	0.93	0.437	0.209	Q9H1B7	Interferon regulatory factor 2-binding protein-like
199	1	1.754	1	2.007	Q68DC2	Ankyrin repeat and SAM domain-containing protein 6
200	1	1	1	1	Q8TE96	ATP-dependent RNA helicase DQX1
201	1	0.87	0.871	0.874	P43403	Tyrosine-protein kinase ZAP-70
202	1	0.784	0.551	0.413	Q4AC94	C2 domain-containing protein 3
203	1	3.167	1.179	1.459	Q5VWG9	Transcription initiation factor TFIID subunit 3
204	1	0.63	0.47	0.292	Q96P48	Arf-GAP with Rho-GAP domain, ANK repeat and PH domain-containing protein 1
205	1	0.498	0.118	0.806	Q9NW13	RNA-binding protein 28
206	1	1	1	1	Q9H2A3	Neurogenin-2
207	1	1.429	1.024	1.01	Q9H0K6	Pseudouridylate synthase 7 homolog-like protein
208	1	3.463	0.826	2.728	Q8IYX0	Zinc finger protein 679
209	1	0.889	0.345	0.568	O95759	TBC1 domain family member 8
210	1	2.136	1	0.726	O43866	CD5 antigen-like
211	1	1.559	0.383	2.116	Q08378	Golgin subfamily A member 3
212	1	1	1	1	Q6NS38	Alpha-ketoglutarate-dependent dioxygenase alkB homolog 2
213	1	0.841	0.471	2.418	Q9Y4C2	Protein FAM115A

214	1	0.675	0.625	0.826	Q96RS6	NudC domain-containing protein 1
215	1	2.318	0.512	0.639	P20020	Plasma membrane calcium-transporting ATPase 1
216	1	0.801	0.628	0.77	Q9H0J4	Glutamine-rich protein 2
217	1	1	1	1	Q12805	EGF-containing fibulin-like extracellular matrix protein 1
218	1	1.39	0.582	0.72	P55199	RNA polymerase II elongation factor ELL
219	1	1.113	1	2.155	P15144	Aminopeptidase N
220	1	1	1	1	Q8N143	B-cell CLL/lymphoma 6 member B protein
221	1	1.923	1.056	1.053	P42701	Interleukin-12 receptor subunit beta-1
222	1	1.208	0.872	0.333	Q8WXS5	Voltage-dependent calcium channel gamma-8 subunit
223	1	2.027	2.832	1.415	Q14872	Metal regulatory transcription factor 1
224	1	2.761	2.118	1.564	O15553	Pyrin
225	1	0.859	0.883	0.449	P21506	Zinc finger protein 10
226	1	2.285	2.126	1.994	P56706	Protein Wnt-7b
227	1	1	1	1	Q8IXV7	Kelch domain-containing protein 8B
228	1	0.554	0.911	1	P35626	Beta-adrenergic receptor kinase 2
229	1	3.896	0.975	3.816	Q9UL01	Dermatan-sulfate epimerase
230	1	0.031	0.927	0.336	Q13976	cGMP-dependent protein kinase 1
231	1	2.579	1.672	0.99	Q6UX07	Dehydrogenase/reductase SDR family member 13
232	1	2.732	1.676	3.215	Q9Y5B9	FACT complex subunit SPT16
233	1	1.332	4.569	3.441	Q8NBV8	Synaptotagmin-8
234	1	1.282	2.046	1.33	Q3KP66	Uncharacterized protein C1orf106
235	1	1.571	0.663	0.877	P52797	Ephrin-A3
236	1	1	1	1	Q9NY43	BarH-like 2 homeobox protein
237	1	0.756	0.299	0.462	Q8N7M3	Putative uncharacterized protein FLJ40852
238	1	0.699	0.936	0.565	Q9UHP3	Ubiquitin carboxyl-terminal hydrolase 25
239	1	2.117	0.631	0.62	Q9UER7	Death domain-associated protein 6
240	1	2.485	1	1	Q9Y4D8	Probable E3 ubiquitin-protein ligase HECTD4
241	1	1.428	1.005	0.59	Q7Z6M2	F-box only protein 33
242	1	2.778	2.725	0.563	P17948	Vascular endothelial growth factor receptor 1
243	1	0.584	0.878	0.534	Q96AJ1	Clusterin-associated protein 1
244	1	4.112	7.101	1.237	Q8IU85	Calcium/calmodulin-dependent protein kinase type 1D
245	1	1	1	1	Q96PN6	Adenylate cyclase type 10
246	1	0.931	0.492	0.561	P98175	RNA-binding protein 10
247	1	11.035	11.77	1	O14727	Apoptotic protease-activating factor 1
248	1	1	1	1	P14373	Zinc finger protein RFP

249	1	2.481	1	1	Q08380	Galectin-3-binding protein
250	1	1	0.236	1	Q8TF27	Arf-GAP with GTPase, ANK repeat and PH domain-containing protein 11
251	1	1.123	1.404	0.649	Q9NZM1	Myoferlin
252	1	1	1	1	Q86WR0	Coiled-coil domain-containing protein 25
253	1	4.8	0.975	0.359	Q96MF6	Coenzyme Q-binding protein COQ10 homolog A, mitochondrial
254	1	0.251	0.995	1	Q6P3W7	SCY1-like protein 2
255	1	3.308	0.912	1.23	Q13023	A-kinase anchor protein 6
256	1	6.139	0.764	1.529	Q96J94	Piwi-like protein 1
257	1	2.888	4.827	1	P56715	Oxygen-regulated protein 1
258	1	1	0.471	1	Q9Y2I7	1-phosphatidylinositol 3-phosphate 5-kinase
259	1	1.716	1.744	1.206	Q86YR5	G-protein-signaling modulator 1
260	1	1.049	6.462	2.293	Q9NQC3	Reticulon-4
261	1	1	0.456	1	O60502	Bifunctional protein NCOAT
262	1	0.833	0.675	0.453	Q6N021	Methylcytosine dioxygenase TET2
263	1	0.776	2.136	0.14	Q9H792	Pseudopodium-enriched atypical kinase 1
264	1	1.176	1	1.924	Q8N819	Probable protein phosphatase 1N
265	1	0.963	0.498	0.141	Q8WUD6	Cholinephosphotransferase 1
266	1	1	1	1	Q9BRZ2	E3 ubiquitin-protein ligase TRIM56
267	1	1.322	1	0.776	Q9BXF3	Cat eye syndrome critical region protein 2
268	1	7.057	1.714	4.056	Q7Z6J6	FERM domain-containing protein 5
269	1	1.303	3.598	3.227	Q9NYV4	Cyclin-dependent kinase 12
270	1	1	1	1	P20908	Collagen alpha-1(V) chain
271	1	1.391	1.057	0.909	Q03001	Dystonin
272	1	0.427	1	0.603	Q8N7M2	Zinc finger protein 283
273	1	0.862	1	1	Q01538	Myelin transcription factor 1
274	1	0.883	0.668	0.429	Q99996	A-kinase anchor protein 9
275	1	1.05	0.708	0.637	Q8N5I2	Arrestin domain-containing protein 1
276	1	1	1	1	P22413	Ectonucleotide pyrophosphatase/phosphodiesterase family member 1
277	1	0.172	0.44	0.732	P27694	Replication protein A 70 kDa DNA-binding subunit
278	1	1.183	0.414	1	Q96NL8	Protein C8orf37
279	1	1	1	1	Q9UNL4	Inhibitor of growth protein 4
280	1	1	1	1	P49815	Tuberin
281	1	1.296	0.911	1.747	Q9Y580	RNA-binding protein 7
282	1	1.921	0.631	0.148	Q8WYN0	Cysteine protease ATG4A
283	1	1	1	1	O95996	Adenomatous polyposis coli protein 2

284	1	1	1	1	Q15345	Leucine-rich repeat-containing protein 41
285	1	1	1	1	P51587	Breast cancer type 2 susceptibility protein
286	1	1	1	1	Q5TB80	Protein QN1 homolog
287	1	1.237	0.802	0.674	Q5JSJ4	Protein DDX26B
288	1	0.55	1.177	0.856	P16471	Prolactin receptor
289	1	1.022	1.996	0.619	Q8WXI2	Connector enhancer of kinase suppressor of ras 2
290	1	1	1	1	A8MRT5	Nuclear pore complex-interacting protein-like 3
291	1	1.783	0.986	0.807	Q9Y4A5	Transformation/transcription domain-associated protein
292	1	1.542	1.095	0.251	A6NEL2	Ankyrin repeat domain-containing protein SOWAHB
293	1	1	1	1	P53778	Mitogen-activated protein kinase 12
294	1	0.236	1	0.916	P21579	Synaptotagmin-1
295	1	1	1	1	P22607	Fibroblast growth factor receptor 3
296	1	0.875	0.605	0.712	Q9ULD2	Microtubule-associated tumor suppressor 1
297	1	0.331	0.508	0.061	P14317	Hematopoietic lineage cell-specific protein
298	1	1	2.149	0.189	Q00537	Cyclin-dependent kinase 17
299	1	1.446	0.865	0.799	Q2LD37	Uncharacterized protein KIAA1109
300	1	0.74	0.729	0.277	Q9UBB4	Ataxin-10
301	1	2.046	3.983	7.22	Q9UPQ7	E3 ubiquitin-protein ligase PDZRN3
302	1	1	1	1	Q9Y6D6	Brefeldin A-inhibited guanine nucleotide-exchange protein 1
303	1	2.078	1.099	0.827	Q7Z6C4	Putative ankyrin repeat domain-containing protein 19-like protein
304	1	0.828	0.901	0.624	Q9UK96	F-box only protein 10
305	1	2.474	4.26	1	Q8IVL1	DIS3-like exonuclease 2
306	1	4.35	3.301	4.093	O75369	Filamin-B
307	1	0.916	0.898	1.047	Q14687	Genetic suppressor element 1
308	1	0.599	0.972	1.289	Q5VYK3	Proteasome-associated protein ECM29 homolog
309	1	5.202	0.988	2.11	P49327	Fatty acid synthase
310	1	1.039	0.185	1	Q14203	Dynactin subunit 1
311	1	1.189	0.492	0.825	Q5TIA1	Meiosis inhibitor protein 1
312	1	1.127	0.812	0.927	Q9Y618	Nuclear receptor corepressor 2
313	1	2.7	1.066	1.94	P16157	Ankyrin-1
314	1	1.411	0.498	0.535	Q5VTT2	Uncharacterized protein C9orf135
315	1	1	1	1	Q6NUM6	tRNA wybutosine-synthesizing protein 1 homolog B
316	1	1	1.737	0.396	Q9HCJ2	Leucine-rich repeat-containing protein 4C
317	1	1.066	0.715	0.798	Q9NZJ4	Sacsin
318	1	1	1	1	P01714	Ig lambda chain V-III region SH

319	1	0.631	1.199	0.409	Q8IY21	Probable ATP-dependent RNA helicase DDX60
320	1	1.809	1	1	O43166	Signal-induced proliferation-associated 1-like protein 1
321	1	0.873	0.301	0.314	P35670	Copper-transporting ATPase 2
322	1	1.278	0.566	0.576	Q12791	Calcium-activated potassium channel subunit alpha-1
323	1	1.587	1	0.789	P06276	Cholinesterase
324	1	1.238	2.481	1	Q9Y252	E3 ubiquitin-protein ligase RNF6
325	1	1	1	1	Q9P2N6	KAT8 regulatory NSL complex subunit 3
326	1	3.853	1.731	5.049	Q9UH90	F-box only protein 40
327	1	1	1	1	Q9Y6V0	Protein piccolo
328	1	1.247	0.489	0.339	Q96JM2	Zinc finger protein 462
329	1	1.058	0.955	1.106	Q9BRH9	Zinc finger protein 251
330	1	1	0.416	1	Q9NPF4	Probable tRNA threonylcarbamoyladenosine biosynthesis protein OSGEP
331	1	1	1	1	P08254	Stromelysin-1
332	1	1.08	0.895	0.711	O95171	Sciellin
333	1	1	5.657	1	Q7Z3S9	Notch homolog 2 N-terminal-like protein
334	1	1	1	1	Q9H9Y2	Ribosome production factor 1
335	1	1	1	1	Q9P2K9	Patched domain-containing protein 2
336	1	1	1	1	Q9UFH2	Dynein heavy chain 17, axonemal
337	1	1.592	0.329	0.425	P68543	UBX domain-containing protein 2A
338	1	0.178	1.025	0.822	P46777	60S ribosomal protein L5
339	1	1	1	1	Q7Z5P9	Mucin-19
340	1	0.099	0.107	0.214	P52757	Beta-chimaerin

(II) Replicate 2

S. No.	No. of unique peptides	Fold-change FEB/HC	Fold-change DEF/HC	Fold-change CON/HC	Accession number	Protein name
1	180	0.938	0.784	1.048	P04114	Apolipoprotein B-100
2	107	1.063	0.984	1.138	P01024	Complement C3
3	72	1.032	1.677	1.32	P01023	Alpha-2-macroglobulin
4	19	0.975	1.47	1.31	P20742	Pregnancy zone protein
5	45	1.042	1.042	1.03	P0C0L5	Complement C4-B
6	42	1.282	1.62	1.253	P00450	Ceruloplasmin
7	40	1.629	1.933	1.358	P01009	Alpha-1-antitrypsin

8	39	1.217	1.321	1.042	P02768	Serum albumin
9	36	0.515	1.065	1.096	P02647	Apolipoprotein A-I
10	31	1.076	1.617	1.252	P02787	Serotransferrin
11	29	1.192	1.937	1.281	P06727	Apolipoprotein A-IV
12	2	3.406	3.461	2.059	P14136	Glial fibrillary acidic protein
13	28	1.466	1.177	1.263	P01008	Antithrombin-III
14	27	2.029	1.263	1.368	P01011	Alpha-1-antichymotrypsin
15	24	1.027	0.808	1.003	Q14624	Inter-alpha-trypsin inhibitor heavy chain H4
16	24	0.622	0.624	0.775	P19823	Inter-alpha-trypsin inhibitor heavy chain H2
17	24	0.803	0.828	0.913	P02751	Fibronectin
18	20	0.374	1.122	1.645	P00738	Haptoglobin
19	12	0.342	0.985	1.52	P00739	Haptoglobin-related protein
20	19	1.176	1.558	1.209	P02774	Vitamin D-binding protein
21	17	1.22	0.906	1.151	P02790	Hemopexin
22	17	1.384	1.899	1.456	Q06033	Inter-alpha-trypsin inhibitor heavy chain H3
23	15	1.1	1.716	1.313	P25311	Zinc-alpha-2-glycoprotein
24	15	1.2	1.715	1.286	P01871	Ig mu chain C region
25	15	0.863	0.986	0.812	P43652	Afamin
26	15	1.1	1.086	1.009	P10909	Clusterin
27	15	0.884	0.627	1.042	P19827	Inter-alpha-trypsin inhibitor heavy chain H1
28	14	1.36	1.263	0.632	P00751	Complement factor B
29	12	1.209	1.411	1.22	P08185	Corticosteroid-binding globulin
30	12	1.253	1.07	1.324	P05155	Plasma protease C1 inhibitor
31	12	1.413	1.616	1.296	P08697	Alpha-2-antiplasmin
32	12	1.141	0.948	0.682	P02649	Apolipoprotein E
33	11	0.949	1.283	1.013	P01019	Angiotensinogen
34	11	1.11	1.551	1.157	P04217	Alpha-1B-glycoprotein
35	10	3.198	1.887	1.839	P68871	Hemoglobin subunit beta
36	9	3.565	2.006	2.208	P02042	Hemoglobin subunit delta
37	10	1.178	1.392	0.678	P01857	Ig gamma-1 chain C region
38	9	0.785	0.918	0.582	P01859	Ig gamma-2 chain C region
39	8	1.123	1.283	0.665	P01860	Ig gamma-3 chain C region
40	6	0.84	1.165	0.678	P01861	Ig gamma-4 chain C region
41	10	2.029	2.815	1.869	P02750	Leucine-rich alpha-2-glycoprotein
42	10	1.361	1.283	1.053	P06681	Complement C2
43	9	0.792	1.329	1.287	P02766	Transthyretin
44	9	0.953	1.202	0.597	P51884	Lumican
45	9	0.686	1.362	0.94	Q96KN2	Beta-Ala-His dipeptidase
46	9	0.87	0.749	0.638	O14791	Apolipoprotein L1

47	9	0.825	0.647	0.905	P01042	Kininogen-1
48	9	1.522	1.858	1.228	P05543	Thyroxine-binding globulin
49	8	1.549	2.263	1.927	P19652	Alpha-1-acid glycoprotein 2
50	8	1.962	2.49	2.013	P02763	Alpha-1-acid glycoprotein 1
51	8	1.141	1.268	1.3	P01876	Ig alpha-1 chain C region
52	7	1.191	1.289	1.308	P01877	Ig alpha-2 chain C region
53	8	0.816	0.649	1.074	P27169	Serum paraoxonase/arylesterase 1
54	8	0.355	0.939	0.914	P02652	Apolipoprotein A-II
55	8	1.042	1.131	0.754	P01031	Complement C5
56	8	1.62	1.722	1.336	Q8WZ42	Titin
57	7	2.992	1.739	1.739	P69905	Hemoglobin subunit alpha
58	7	1.494	1.363	1.45	P09871	Complement C1s subcomponent
59	7	0.604	0.659	0.807	P05546	Heparin cofactor 2
60	6	3.625	1.557	1.72	P0DJI8	Serum amyloid A-1 protein
61	6	1.425	0.841	0.999	P02748	Complement component C9
62	6	1.067	1.142	1.03	P22792	Carboxypeptidase N subunit 2
63	6	0.695	0.643	0.661	P80108	Phosphatidylinositol-glycan-specific phospholipase D
64	6	1.764	1.554	1.26	P06396	Gelsolin
65	5	0.508	0.961	0.667	P02656	Apolipoprotein C-III
66	5	0.825	1.863	0.959	P02655	Apolipoprotein C-II
67	5	0.568	0.651	0.687	P02671	Fibrinogen alpha chain
68	5	0.84	1.437	1.35	P01620	Ig kappa chain V-III region SIE
69	5	0.84	1.494	1.852	P18136	Ig kappa chain V-III region HIC
70	5	2.896	2.055	1.201	P04196	Histidine-rich glycoprotein
71	5	0.441	0.695	0.892	O95445	Apolipoprotein M
72	5	1.266	1.233	1.45	P04275	von Willebrand factor
73	5	1.66	1.746	1.571	P08603	Complement factor H
74	5	0.898	1.013	1.897	O75882	Attractin
75	5	1.226	0.918	1.589	P00734	Prothrombin
76	4	0.839	1.075	0.814	P01834	Ig kappa chain C region
77	4	1.2	1.612	1.076	B9A064	Immunoglobulin lambda-like polypeptide 5
78	3	1.119	1.459	1.029	P0CG06	Ig lambda-3 chain C regions
79	4	1.865	1.291	0.971	P04004	Vitronectin
80	4	0.875	0.788	0.845	P02760	Protein AMBP
81	4	0.671	1	1.188	P05090	Apolipoprotein D
82	4	1.037	1.301	1.04	P01625	Ig kappa chain V-IV region Len
83	4	4.067	2.874	1.558	P02741	C-reactive protein
84	4	1.319	1.628	1.004	Q08380	Galectin-3-binding protein
85	4	1.029	2.071	1.657	P01880	Ig delta chain C region

86	4	2.227	1.619	1.03	P06276	Cholinesterase
87	4	1.849	2.272	2.192	P22352	Glutathione peroxidase 3
88	4	1.064	0.686	0.806	P18428	Lipopolysaccharide-binding protein
89	4	1.42	1.501	1.415	Q6EMK4	Vasorin
90	4	1.75	1.229	1.915	P00915	Carbonic anhydrase 1
91	4	1.362	0.704	0.731	P07358	Complement component C8 beta chain
92	4	1.634	0.448	0.978	P13671	Complement component C6
93	4	0.494	1.013	1.076	Q03001	Dystonin
94	4	1.336	1.252	1.106	Q6TFL3	Coiled-coil domain-containing protein 171
95	3	0.945	1.411	1.215	P02765	Alpha-2-HS-glycoprotein
96	3	1.095	1.211	0.773	P01593	Ig kappa chain V-I region AG
97	2	1.675	0.971	0.909	P01613	Ig kappa chain V-I region Ni
98	3	0.681	1.296	1.57	P12259	Coagulation factor V
99	3	0.659	0.611	1.49	P36955	Pigment epithelium-derived factor
100	3	0.391	0.973	1.155	P10643	Complement component C7
101	3	2.208	2.192	1.302	P00747	Plasminogen
102	3	1.42	1.364	1.283	P00736	Complement C1r subcomponent
103	3	0.638	0.593	0.648	P02654	Apolipoprotein C-I
104	3	0.947	1.109	0.992	P15169	Carboxypeptidase N catalytic chain
105	3	1.345	1.461	1.39	P81605	Dermcidin
106	3	1.187	1.518	1.335	P04180	Phosphatidylcholine-sterol acyltransferase
107	3	1.139	1.379	1.174	P01624	Ig kappa chain V-III region POM
108	3	1.252	0.974	1.137	P01621	Ig kappa chain V-III region NG9
109	2	1.184	0.469	1.137	P04434	Ig kappa chain V-III region VH
110	3	0.769	0.568	0.562	Q9UGM5	Fetuin-B
111	3	0.786	1.037	0.703	P02743	Serum amyloid P-component
112	3	1.452	3.706	1.414	P08571	Monocyte differentiation antigen CD14
113	3	0.816	1.205	1.015	Q86VB7	Scavenger receptor cysteine-rich type 1 protein M130
114	3	1.224	1.108	1.269	P07225	Vitamin K-dependent protein S
115	3	1.562	2.603	2.302	Q9NZP8	Complement C1r subcomponent-like protein
116	3	1.477	1.171	0.559	P05156	Complement factor I
117	3	1.3	1.105	1.485	P68366	Tubulin alpha-4A chain
118	3	0.602	0.955	1.105	Q15413	Ryanodine receptor 3
119	3	0.669	1.146	1.028	Q9P2D7	Dynein heavy chain 1, axonemal
120	3	0.812	1.061	0.671	Q8N3C0	Activating signal cointegrator 1 complex subunit 3
121	3	1.061	2.186	1.434	Q96DT5	Dynein heavy chain 11, axonemal
122	3	0.999	1.413	1.174	Q6PIF6	Unconventional myosin-VIIb
123	3	0.816	1.146	1.066	Q9UQE7	Structural maintenance of chromosomes protein 3
124	3	0.319	0.799	0.911	Q9Y490	Talin-1

125	3	0.433	0.69	1.098	Q6S8J3	POTE ankyrin domain family member E
126	3	1	1	1	Q96PD5	N-acetylmuramoyl-L-alanine amidase
127	3	1.905	2.294	1.035	Q9NYC9	Dynein heavy chain 9, axonemal
128	3	1.035	1.252	1.106	Q8NB14	Ubiquitin carboxyl-terminal hydrolase 38
129	3	1.035	1.252	1.106	Q9NXL6	SID1 transmembrane family member 1
130	3	0.914	0.933	1.643	Q86UQ4	ATP-binding cassette sub-family A member 13
131	3	0.624	0.421	0.883	Q96JI7	Spatacsin
132	3	0.654	0.835	0.741	Q14644	Ras GTPase-activating protein 3
133	3	1.035	1.252	1.104	Q8IVF2	Protein AHNAK2
134	3	0.438	1.216	1.084	P21817	Ryanodine receptor 1
135	2	1.104	1.161	1.124	P01598	Ig kappa chain V-I region EU
136	2	2.996	1.656	1.571	P02747	Complement C1q subcomponent subunit C
137	2	0.583	0.393	0.746	P04003	C4b-binding protein alpha chain
138	2	2.94	3.218	2.244	P02746	Complement C1q subcomponent subunit B
139	2	0.754	1.892	1.215	P01591	Immunoglobulin J chain
140	2	1.117	0.827	0.735	P35542	Serum amyloid A-4 protein
141	2	0.832	1.711	1.404	P01617	Ig kappa chain V-II region TEW
142	2	0.7	2.437	1.437	P04278	Sex hormone-binding globulin
143	2	1.316	1.515	2.533	P20671	Histone H2A type 1-D
144	2	1.941	1.174	0.747	P07357	Complement component C8 alpha chain
145	2	0.637	0.759	0.787	P01781	Ig heavy chain V-III region GAL
146	2	1.677	1.575	1.396	P19320	Vascular cell adhesion protein 1
147	2	4.966	3.017	1.444	P07360	Complement component C8 gamma chain
148	2	2.004	2.713	1.741	P20848	Putative alpha-1-antitrypsin-related protein
149	2	0.44	1.123	1.245	Q9UFH2	Dynein heavy chain 17, axonemal
150	2	0.938	2.175	1.483	P09172	Dopamine beta-hydroxylase
151	2	1.692	2.001	2.123	Q5VST9	Obscurin
152	2	0.988	1.912	0.641	Q99784	Noelin
153	2	0.383	0.634	1.182	P31151	Protein S100-A7
154	2	0.666	1.759	1.758	Q562R1	Beta-actin-like protein 2
155	2	1.305	1.559	0.881	P48740	Mannan-binding lectin serine protease 1
156	2	1.207	1.306	0.508	Q08554	Desmocollin-1
157	2	0.967	1.539	1.368	Q8TDW7	Protocadherin Fat 3
158	2	0.885	1.386	1.133	P78332	RNA-binding protein 6
159	2	0.719	1	1	P15924	Desmoplakin
160	2	0.482	0.634	0.744	Q8IVL1	Neuron navigator 2
161	2	0.85	3.931	9.687	Q14315	Filamin-C
162	2	1.035	0.591	0.763	Q5KSL6	Diacylglycerol kinase kappa
163	2	1	0.166	0.441	P35858	Insulin-like growth factor-binding protein complex acid labile subunit

164	2	1.77	1.917	1.678	E7ETH6	Zinc finger protein 587B
165	2	0.952	0.696	1.065	Q8NDA2	Hemicentin-2
166	2	1.36	1.142	1.164	O43264	Centromere/kinetochore protein zw10 homolog
167	2	0.819	0.192	1.073	P22105	Tenascin-X
168	2	1.799	1.791	1.937	Q96PN6	Adenylate cyclase type 10
169	2	1.347	1.364	0.872	P55199	RNA polymerase II elongation factor ELL
170	2	4.516	6.251	6.165	P20815	Cytochrome P450 3A5
171	2	1.388	2.318	1.891	Q99996	A-kinase anchor protein 9
172	2	1.086	0.888	0.721	Q8IYB7	DIS3-like exonuclease 2
173	2	0.732	8.568	1.021	Q86XX4	Extracellular matrix protein FRAS1
174	2	0.898	0.995	0.767	Q8IYW2	Tetratricopeptide repeat protein 40
175	2	1.831	2.135	1.51	Q96T23	Remodeling and spacing factor 1
176	2	0.654	0.793	0.397	P55058	Phospholipid transfer protein
177	2	0.451	0.556	0.771	Q6P4A8	Phospholipase B-like 1
178	2	1.031	1.656	1.117	Q16760	Diacylglycerol kinase delta
179	2	1.332	1.545	1.074	P80748	Ig lambda chain V-III region LOI
180	2	1.869	2.272	1.368	Q8IV38	Ankyrin repeat and MYND domain-containing protein 2
181	2	0.844	0.818	1	P33151	Cadherin-5
182	2	1.565	1.303	1.417	P20929	Nebulin
183	2	1.063	0.657	2.024	A2PYH4	Probable ATP-dependent DNA helicase HFM1
184	2	1.306	2.791	1.609	Q8IVF4	Dynein heavy chain 10, axonemal
185	2	1.388	2.318	1.891	P46821	Microtubule-associated protein 1B
186	2	0.851	1.767	0.801	Q92538	Golgi-specific brefeldin A-resistance guanine nucleotide exchange factor 1
187	2	1.102	1.747	1.323	Q5EBL2	Zinc finger protein 628
188	2	1.1	1.651	1.181	Q9UGK3	Signal-transducing adaptor protein 2
189	2	0.59	0.869	1.088	Q496Y0	LON peptidase N-terminal domain and RING finger protein 3
190	2	0.775	1.117	0.898	Q15772	Striated muscle preferentially expressed protein kinase
191	2	1.308	1.536	0.726	O60662	Kelch repeat and BTB domain-containing protein 10
192	2	2.924	1.778	1.8	Q15582	Transforming growth factor-beta-induced protein ig-h3
193	2	2.957	1.564	0.398	Q14571	Inositol 1,4,5-trisphosphate receptor type 2
194	2	0.903	1.686	0.346	P08238	Heat shock protein HSP 90-beta
195	2	3.222	1.57	3.202	Q14C86	GTPase-activating protein and VPS9 domain-containing protein 1
196	2	0.822	1.32	0.378	Q8TEK3	Histone-lysine N-methyltransferase, H3 lysine-79 specific
197	2	1.609	1.252	2.425	Q9ULU8	Calcium-dependent secretion activator 1
198	2	1.298	1.72	1.114	P55884	Eukaryotic translation initiation factor 3 subunit B
199	2	1.01	1.727	1.426	Q9UHV7	Mediator of RNA polymerase II transcription

						subunit 13
200	2	0.405	0.617	0.929	Q5JR59	Microtubule-associated tumor suppressor candidate 2
201	2	0.813	0.835	0.446	Q9UH99	SUN domain-containing protein 2
202	2	1.423	1.663	1.506	Q9C0D5	Protein TANC1
203	2	1.112	1.248	0.824	Q9Y6V0	Protein piccolo
204	1	0.768	1.229	1.443	P01766	Ig heavy chain V-III region BRO
205	1	1.113	3.406	1.137	P01717	Ig lambda chain V-IV region Hil
206	1	0.658	5	2.102	P01774	Ig heavy chain V-III region POM
207	1	2.807	1.091	1.742	P43251	Biotinidase
208	1	4.389	4.155	0.685	Q92820	Gamma-glutamyl hydrolase
209	1	1.727	6.029	1.152	P13473	Lysosome-associated membrane glycoprotein 2
210	1	1.235	0.973	0.515	P23083	Ig heavy chain V-I region V35
211	1	0.523	1	2.278	P61626	Lysozyme C
212	1	1.557	1.479	2.617	Q01459	Di-N-acetylchitobiase
213	1	0.779	0.957	1.009	Q13790	Apolipoprotein F
214	1	3.091	2.011	3.361	Q9UKN8	General transcription factor 3C polypeptide 4
215	1	0.786	1.216	0.771	P04433	Ig kappa chain V-III region VG
216	1	1	1.565	0.258	P01611	Ig kappa chain V-I region Wes
217	1	0.526	1	0.201	P33908	Mannosyl-oligosaccharide 1,2-alpha-mannosidase IA
218	1	0.755	1.144	0.594	P06702	Protein S100-A9
219	1	1	1	1	Q5D862	Filaggrin-2
220	1	1	1	1	P02511	Alpha-crystallin B chain
221	1	3.106	0.99	1	P01602	Ig kappa chain V-I region HK102
222	1	1.195	1.576	1.412	P61769	Beta-2-microglobulin
223	1	1.507	1.748	1.463	O43866	CD5 antigen-like
224	1	4.993	3.658	2.126	Q03591	Complement factor H-related protein 1
225	1	2.687	6.498	4.877	P15814	Immunoglobulin lambda-like polypeptide 1
226	1	0.607	1.213	0.609	Q8WX92	Negative elongation factor B
227	1	2.789	2.028	0.343	P05452	Tetranectin
228	1	2.069	0.773	1.403	P03952	Plasma kallikrein
229	1	0.356	0.329	0.293	Q7Z2Y8	Interferon-induced very large GTPase 1
230	1	1.915	2.924	2.641	P04438	Ig heavy chain V-II region SESS
231	1	1.548	2.283	1.094	P11597	Cholesteryl ester transfer protein
232	1	2.498	1.562	1.695	P32119	Peroxiredoxin-2
233	1	1.519	1.696	0.361	P04430	Ig kappa chain V-I region BAN
234	1	0.692	1.448	1.566	P02745	Complement C1q subcomponent subunit A
235	1	1.155	1.762	1.499	Q9Y6A9	Signal peptidase complex subunit 1
236	1	1	0.495	0.42	P20774	Mimecan

237	1	0.49	0.987	1.218	Q14BN4	Sarcolemmal membrane-associated protein
238	1	1.085	1.681	1.703	P46777	60S ribosomal protein L5
239	1	1.188	1.773	0.697	P40425	Pre-B-cell leukemia transcription factor 2
240	1	1.23	10.382	5.736	Q3KNS1	Patched domain-containing protein 3
241	1	0.725	0.433	1	Q16798	NADP-dependent malic enzyme, mitochondrial
242	1	1.795	3.017	1.246	P07359	Platelet glycoprotein Ib alpha chain
243	1	3.218	1	1	P13598	Intercellular adhesion molecule 2
244	1	0.403	1.242	1.216	Q96G01	Protein bicaudal D homolog 1
245	1	0.231	0.339	0.551	Q9C035	Tripartite motif-containing protein 5
246	1	1	0.206	1.888	Q14997	Proteasome activator complex subunit 4
247	1	2.821	0.6	2.432	P01700	Ig lambda chain V-I region HA
248	1	1.386	1.679	0.947	P31025	Lipocalin-1
249	1	0.927	1.287	1.299	Q9C0A6	SET domain-containing protein 5
250	1	0.75	1.376	1.434	O14810	Complexin-1
251	1	1.507	1.463	1.073	Q5R372	Rab GTPase-activating protein 1-like
252	1	1.349	1.072	1.284	Q6ZN30	Zinc finger protein basonuclin-2
253	1	1.527	1.8	1.134	A6NI56	Coiled-coil domain-containing protein 154
254	1	1	0.138	1.81	P13796	Plastin-2
255	1	0.586	0.773	0.719	Q5TEV5	Putative uncharacterized protein C1orf134
256	1	1	1	1	Q96HU8	GTP-binding protein Di-Ras2
257	1	0.378	1.066	1.838	C9J069	Uncharacterized protein C9orf172
258	1	0.524	1.413	0.873	Q15431	Synaptonemal complex protein 1
259	1	1.005	1.67	1.9	P06316	Ig lambda chain V-I region BL2
260	1	1.394	1.727	1.53	Q9NR50	Translation initiation factor eIF-2B subunit gamma
261	1	1.261	1.893	1.219	Q9UFW8	CGG triplet repeat-binding protein 1
262	1	0.349	0.992	0.148	Q8NCB2	CaM kinase-like vesicle-associated protein
263	1	1	1	1	P06319	Ig lambda chain V-VI region EB4
264	1	0.697	1.195	1.737	P49327	Fatty acid synthase
265	1	0.673	0.476	0.541	B2RTY4	Unconventional myosin-IXa
266	1	1	1	1	P02792	Ferritin light chain
267	1	0.672	1.443	1.11	P02775	Platelet basic protein
268	1	0.94	0.762	0.792	O75593	Forkhead box protein H1
269	1	1.037	1.557	0.831	P08F94	Fibrocystin
270	1	0.361	1.046	1.463	P11226	Mannose-binding protein C
271	1	1	1	1	O75015	Low affinity immunoglobulin gamma Fc region receptor III-B
272	1	1.478	1.821	1.408	O95672	Endothelin-converting enzyme-like 1
273	1	3.681	1.879	1.835	Q9HB29	Interleukin-1 receptor-like 2
274	1	1	1	1	P01034	Cystatin-C
275	1	2.565	2.163	1.994	P20701	Integrin alpha-L

276	1	1	1	0.694	P29622	Kallistatin
277	1	0.825	1.075	0.643	Q14872	Metal regulatory transcription factor 1
278	1	3.031	3.888	2.079	Q8TF20	Zinc finger protein 721
279	1	1.356	0.437	0.564	P05165	Propionyl-CoA carboxylase alpha chain, mitochondrial
280	1	5.889	4.305	1.305	Q68DK2	Zinc finger FYVE domain-containing protein 26
281	1	0.989	1.685	0.822	Q14151	Scaffold attachment factor B2
282	1	2.719	6.845	3.97	P02749	Beta-2-glycoprotein 1
283	1	0.398	1.329	1.472	Q15208	Serine/threonine-protein kinase 38
284	1	2.799	2.922	2.024	O00472	RNA polymerase II elongation factor ELL2
285	1	0.72	0.872	0.624	Q92615	La-related protein 4B
286	1	1.035	0.681	0.5	P20618	Proteasome subunit beta type-1
287	1	1.799	1.027	0.964	Q9NYS0	NF-kappa-B inhibitor-interacting Ras-like protein 1
288	1	0.342	0.268	0.212	Q96RR4	Calcium/calmodulin-dependent protein kinase kinase 2
289	1	0.353	0.878	1.565	P41214	Eukaryotic translation initiation factor 2D
290	1	0.794	0.823	0.711	Q5JTH9	RRP12-like protein
291	1	1	1	1	P02675	Fibrinogen beta chain
292	1	2.01	1.648	1.045	Q13075	Baculoviral IAP repeat-containing protein 1
293	1	1.941	0.316	0.342	P08670	Vimentin
294	1	0.4	1.251	0.536	Q6P587	Acylpyruvase FAHD1, mitochondrial
295	1	1.908	1.937	4.287	Q9NZ08	Endoplasmic reticulum aminopeptidase 1
296	1	1	1	1	Q92618	Zinc finger protein 516
297	1	0.946	1.103	0.6	Q12933	TNF receptor-associated factor 2
298	1	1.224	1.254	1.087	Q9HCJ0	Trinucleotide repeat-containing gene 6C protein
299	1	3.485	2.339	2.774	Q6ZW33	MICAL C-terminal-like protein
300	1	1.681	2.075	1.459	Q13459	Unconventional myosin-IXb
301	1	0.584	1.104	0.202	Q7Z2Z2	Elongation factor Tu GTP-binding domain-containing protein 1
302	1	1.03	0.431	0.432	P54277	PMS1 protein homolog 1
303	1	1	1	1	B1AK53	Espin
304	1	0.566	0.844	0.619	O75691	Small subunit processome component 20 homolog
305	1	0.378	0.657	0.496	O75290	Zinc finger protein 780A
306	1	1.325	2.445	1.616	P00748	Coagulation factor XII
307	1	3.258	1.746	1.742	Q69YQ0	Cytospin-A
308	1	1.041	1.347	0.7	Q15029	116 kDa U5 small nuclear ribonucleoprotein component
309	1	1.705	0.834	2.418	Q8WV99	AN1-type zinc finger protein 2B
310	1	0.632	2.102	2.215	Q9UJC3	Protein Hook homolog 1
311	1	1.116	1.554	1.309	P35556	Fibrillin-2
312	1	0.146	1	0.349	O94874	E3 UFM1-protein ligase 1

313	1	0.334	0.277	0.41	O60928	Inward rectifier potassium channel 13
314	1	0.989	1.495	1	P02679	Fibrinogen gamma chain
315	1	0.342	1.937	0.171	Q0VDD8	Dynein heavy chain 14, axonemal
316	1	5.077	3.401	1.647	Q6P1N9	Putative deoxyribonuclease TATDN1
317	1	1.381	1.636	2.634	Q15349	Ribosomal protein S6 kinase alpha-2
318	1	1.753	2.533	0.324	Q7L591	Docking protein 3
319	1	1.609	1.151	1.824	P15144	Aminopeptidase N
320	1	0.803	0.93	0.818	Q96CU9	FAD-dependent oxidoreductase domain-containing protein 1
321	1	0.751	1.537	0.991	Q5JST6	EF-hand domain-containing family member C2
322	1	0.778	1.022	1.254	Q68DQ2	Beta/gamma crystallin domain-containing protein 3
323	1	0.335	1.986	1.199	Q14126	Desmoglein-2
324	1	0.531	0.713	0.764	Q9NZR2	Low-density lipoprotein receptor-related protein 1B
325	1	1.183	0.918	0.961	P10911	Proto-oncogene DBL
326	1	0.791	3.013	1.087	Q96QS1	Tetraspanin-32
327	1	5.537	1	1.692	P36873	Serine/threonine-protein phosphatase PP1-gamma catalytic subunit
328	1	3.195	4.579	2.608	O75131	Copine-3
329	1	0.875	1.215	1.019	Q9UBC5	Unconventional myosin-Ia
330	1	0.947	1.03	1.233	P28289	Tropomodulin-1
331	1	1.155	0.484	1.154	O95997	Securin
332	1	0.818	1.837	0.648	Q6UXB8	Peptidase inhibitor 16
333	1	0.377	1	0.124	P0DJD1	RANBP2-like and GRIP domain-containing protein 2
334	1	2.872	5.984	0.983	O15146	Muscle, skeletal receptor tyrosine-protein kinase
335	1	3.019	2.148	4.112	Q3BBV0	Neuroblastoma breakpoint family member 1
336	1	0.239	0.137	0.191	P25789	Proteasome subunit alpha type-4
337	1	1	1	1	O42043	HERV-K_1q23.3 provirus ancestral Env polyprotein
338	1	2.235	2.367	1.173	Q8NBP0	Tetratricopeptide repeat protein 13
339	1	1.3	2.357	1.087	A2A2Z9	Ankyrin repeat domain-containing protein 18B
340	1	2.526	3.01	1.559	Q9BRJ2	39S ribosomal protein L45, mitochondrial
341	1	1.929	0.723	1.75	P01767	Ig heavy chain V-III region BUT
342	1	1.015	0.677	0.266	Q3SX61	Putative beta-glucuronidase-like protein MGC129979
343	1	0.312	0.414	0.508	P38398	Breast cancer type 1 susceptibility protein
344	1	0.961	1.327	1.07	Q8NEH6	Meiosis-specific nuclear structural protein 1
345	1	1.407	2.145	1.235	P41208	Centrin-2
346	1	1	0.508	0.152	Q9ULE3	DENN domain-containing protein 2A
347	1	1.427	1.416	0.773	Q9C0C9	Ubiquitin-conjugating enzyme E2 O
348	1	1.223	0.6	0.984	Q02325	Plasminogen-like protein B

349	1	1	0.266	1	Q9Y4K1	Absent in melanoma 1 protein
350	1	0.518	0.427	0.661	P02786	Transferrin receptor protein 1
351	1	0.62	0.227	1	P51530	DNA replication ATP-dependent helicase/nuclease DNA2
352	1	1.154	1.178	0.536	Q9BZE4	Nucleolar GTP-binding protein 1
353	1	0.613	1	0.335	Q96BR9	Zinc finger and BTB domain-containing protein 8A
354	1	1.739	1.097	1.03	P35125	Ubiquitin carboxyl-terminal hydrolase 6
355	1	1.581	2.032	1.517	Q14669	E3 ubiquitin-protein ligase TRIP12
356	1	2.694	2.85	2.977	O95376	E3 ubiquitin-protein ligase ARIH2
357	1	1.191	1.992	1.471	Q07617	Sperm-associated antigen 1
358	1	2.129	3.304	3.456	Q9UHB6	LIM domain and actin-binding protein 1
359	1	1.86	2.249	1.982	O94986	Centrosomal protein of 152 kDa
360	1	1.408	0.828	1.075	Q86YN6	Peroxisome proliferator-activated receptor gamma coactivator 1-beta
361	1	0.921	3.618	1.622	P35443	Thrombospondin-4
362	1	0.175	0.106	1	P05023	Sodium/potassium-transporting ATPase subunit alpha-1
363	1	0.921	1	0.941	P23142	Fibulin-1
364	1	0.49	4.519	2.461	Q5SGD2	Protein phosphatase 1L
365	1	0.247	1.012	1.509	Q6P3S1	DENN domain-containing protein 1B
366	1	1.433	1.739	1.193	A6NLF2	RNA polymerase II transcription factor SIII subunit A3-like-2
367	1	1.842	0.916	2.719	P00742	Coagulation factor X
368	1	3.42	5.453	3.304	P04066	Tissue alpha-L-fucosidase
369	1	1	1	1	Q9P225	Dynein heavy chain 2, axonemal
370	1	0.537	0.285	1.568	Q9UHR4	Brain-specific angiogenesis inhibitor 1-associated protein 2-like protein 1
371	1	1.589	1	1.39	Q9NP08	Homeobox protein HMX1
372	1	0.667	1	0.325	Q92783	Signal transducing adapter molecule 1
373	1	0.25	0.526	0.498	Q9Y5K6	CD2-associated protein
374	1	1.006	1.413	1.363	O75636	Ficolin-3
375	1	1	1	1	Q9H257	Caspase recruitment domain-containing protein 9
376	1	7.25	0.656	2.296	Q9P273	Teneurin-3
377	1	1.07	1	0.323	P08294	Extracellular superoxide dismutase [Cu-Zn]
378	1	2.432	3.231	3.141	P53420	Collagen alpha-4(IV) chain
379	1	1.188	2.275	1	Q8NFB2	Transmembrane protein 185A
380	1	1.081	1.162	0.434	Q96T68	Histone-lysine N-methyltransferase SETDB2
381	1	0.126	0.537	0.754	Q9H1B7	Interferon regulatory factor 2-binding protein-like
382	1	1.314	0.891	1.509	Q9H4H8	Protein FAM83D
383	1	2.205	1.988	0.698	O14559	Rho GTPase-activating protein 33
384	1	0.786	3.74	2.043	P06331	Ig heavy chain V-II region ARH-77
385	1	2.258	8.409	0.318	Q12797	Aspartyl/asparaginyl beta-hydroxylase

386	1	0.782	2.088	0.517	P25054	Adenomatous polyposis coli protein
387	1	1.578	1.565	1.437	Q12768	WASH complex subunit strumpellin
388	1	4.63	2.278	8.76	Q5FWF5	N-acetyltransferase ESCO1
389	1	1.67	2.18	1.387	O15050	TPR and ankyrin repeat-containing protein 1
390	1	11.753	1	1	Q08881	Tyrosine-protein kinase ITK/TSK
391	1	0.736	1	1	Q38SD2	Leucine-rich repeat serine/threonine-protein kinase 1
392	1	0.868	0.646	0.889	Q86UK0	ATP-binding cassette sub-family A member 12
393	1	0.724	0.848	0.444	Q9HCB6	Spondin-1
394	1	0.296	0.811	1.063	Q96L73	Histone-lysine N-methyltransferase, H3 lysine-36 and H4 lysine-20 specific
395	1	0.895	1.335	0.832	A8MQ14	Zinc finger protein 850
396	1	0.967	0.923	0.685	Q8WXD9	Caskin-1
397	1	1	1	1	Q8IUG5	Unconventional myosin-XVIIIb
398	1	1.054	0.874	0.42	O60494	Cubilin
399	1	1.448	0.461	1.042	P35626	Beta-adrenergic receptor kinase 2
400	1	1	1	1	Q86U06	Probable RNA-binding protein 23
401	1	1.82	2.884	2.572	P15056	Serine/threonine-protein kinase B-raf
402	1	1.266	1.828	1.006	O95935	T-box transcription factor TBX18
403	1	1.101	0.27	1.257	Q9NXZ1	Sarcoma antigen 1
404	1	1.753	3.285	4.053	Q9P2R6	Arginine-glutamic acid dipeptide repeats protein
405	1	1.516	2.648	2.022	Q96NY7	Chloride intracellular channel protein 6
406	1	0.93	1.016	0.842	Q8NEC7	Glutathione S-transferase C-terminal domain-containing protein
407	1	1	1	1	Q12802	A-kinase anchor protein 13
408	1	3.371	5.445	3.297	Q49AN0	Cryptochrome-2
409	1	0.361	2.45	0.895	Q9ULH0	Kinase D-interacting substrate of 220 kDa
410	1	1.737	1.367	1.611	Q5SW79	Centrosomal protein of 170 kDa
411	1	2.145	1.786	0.441	P62316	Small nuclear ribonucleoprotein Sm D2
412	1	1.067	1.455	1.139	Q8WV41	Sorting nexin-33

(III) Replicate 3

S. No.	No. of unique peptides	Fold-change FEB/HC	Fold-change DEF/HC	Fold-change CON/HC	Accession number	Protein name
1	145	0.559	0.639	0.548	P04114	Apolipoprotein B-100
2	79	0.553	0.574	0.623	P01024	Complement C3
3	43	0.558	0.877	0.781	P01023	Alpha-2-macroglobulin
4	16	0.671	0.888	0.626	P20742	Pregnancy zone protein
5	41	0.552	0.689	0.667	P0C0L5	Complement C4-B
6	39	0.845	1.152	0.922	P00450	Ceruloplasmin
7	37	0.779	1.529	0.932	P01009	Alpha-1-antitrypsin
8	36	0.333	0.444	0.507	P02647	Apolipoprotein A-I
9	32	0.724	0.751	0.676	P02768	Serum albumin
10	26	0.435	0.696	0.398	P06727	Apolipoprotein A-IV
11	26	0.619	0.953	0.713	P02787	Serotransferrin
12	25	1.053	1.153	0.994	P01011	Alpha-1-antichymotrypsin
13	24	0.778	0.826	0.885	P01008	Antithrombin-III
14	20	0.509	0.514	0.531	Q14624	Inter-alpha-trypsin inhibitor heavy chain H4
15	20	0.474	0.48	0.421	P02751	Fibronectin
16	18	0.459	0.341	0.446	P19823	Inter-alpha-trypsin inhibitor heavy chain H2
17	17	0.45	0.783	0.693	P00738	Haptoglobin
18	16	0.438	0.409	0.533	P19827	Inter-alpha-trypsin inhibitor heavy chain H1
19	16	0.767	1.252	0.907	P08697	Alpha-2-antiplasmin
20	15	0.748	1.383	0.898	Q06033	Inter-alpha-trypsin inhibitor heavy chain H3
21	13	0.657	1.029	0.756	P02774	Vitamin D-binding protein
22	13	0.606	0.922	0.564	P25311	Zinc-alpha-2-glycoprotein
23	13	0.523	0.591	0.47	P43652	Afamin
24	13	0.708	1.238	0.737	P06681	Complement C2
25	13	0.886	0.99	0.714	Q8WZ42	Titin
26	12	0.637	0.717	0.625	P02790	Hemopexin
27	12	0.506	0.565	0.466	P10909	Clusterin
28	12	0.726	0.632	0.629	P00751	Complement factor B
29	11	0.975	1.183	0.875	P02649	Apolipoprotein E
30	10	0.663	1.249	0.88	P01871	Ig mu chain C region
31	9	0.845	1.102	0.872	P01857	Ig gamma-1 chain C region
32	8	0.819	0.973	0.817	P01860	Ig gamma-3 chain C region
33	7	0.626	0.779	0.642	P01859	Ig gamma-2 chain C region
34	6	0.649	0.88	0.862	P01861	Ig gamma-4 chain C region
35	9	0.588	0.754	0.395	P51884	Lumican
36	9	0.523	0.581	0.571	P05155	Plasma protease C1 inhibitor

37	9	0.325	0.331	0.432	P05546	Heparin cofactor 2
38	8	0.52	0.716	0.523	P02766	Transthyretin
39	8	0.637	0.949	0.846	P08185	Corticosteroid-binding globulin
40	8	0.88	1.166	0.992	P19652	Alpha-1-acid glycoprotein 2
41	8	0.82	1.402	1.138	P02763	Alpha-1-acid glycoprotein 1
42	8	0.456	0.561	0.958	O14791	Apolipoprotein L1
43	8	0.791	1.277	0.657	P04275	von Willebrand factor
44	8	0.44	0.786	0.575	P01031	Complement C5
45	7	1.742	2.111	1.158	P68871	Hemoglobin subunit beta
46	6	1.664	1.84	1.093	P02042	Hemoglobin subunit delta
47	7	1.089	2.388	1.494	P02750	Leucine-rich alpha-2-glycoprotein
48	7	0.306	0.299	0.414	P02652	Apolipoprotein A-II
49	7	0.719	1.094	0.727	P01019	Angiotensinogen
50	7	0.84	1.187	0.791	P05543	Thyroxine-binding globulin
51	7	0.525	0.713	0.61	P09871	Complement C1s subcomponent
52	7	1.392	1.605	1.026	P18428	Lipopolysaccharide-binding protein
53	6	0.633	1.055	0.641	P01876	Ig alpha-1 chain C region
54	6	0.616	1.011	0.69	P01877	Ig alpha-2 chain C region
55	6	0.669	0.953	0.793	P02671	Fibrinogen alpha chain
56	6	0.421	0.584	0.52	P01042	Kininogen-1
57	6	0.707	0.966	0.779	P00734	Prothrombin
58	6	0.363	0.398	0.323	Q96KN2	Beta-Ala-His dipeptidase
59	5	1.366	1.516	0.859	P0DJI8	Serum amyloid A-1 protein
60	5	0.484	0.658	0.459	P02656	Apolipoprotein C-III
61	5	0.684	0.891	0.694	P01834	Ig kappa chain C region
62	5	0.509	0.787	0.468	P02655	Apolipoprotein C-II
63	5	0.337	0.46	0.479	P27169	Serum paraoxonase/arylesterase 1
64	5	0.992	0.915	0.813	Q08380	Galectin-3-binding protein
65	5	0.365	0.808	0.719	P10643	Complement component C7
66	4	1.559	1.772	0.969	P69905	Hemoglobin subunit alpha
67	4	0.709	1.193	0.923	P22792	Carboxypeptidase N subunit 2
68	4	1.795	10.989	1.929	P02741	C-reactive protein
69	4	0.308	0.416	0.352	P05090	Apolipoprotein D
70	4	0.777	1.184	1.367	P08571	Monocyte differentiation antigen CD14
71	4	0.745	0.971	0.403	P06396	Gelsolin
72	4	0.761	0.549	0.622	P02748	Complement component C9
73	4	0.692	0.868	0.625	P04206	Ig kappa chain V-III region GOL
74	4	0.66	1.247	0.749	P04217	Alpha-1B-glycoprotein
75	4	0.642	1.03	0.752	P22352	Glutathione peroxidase 3

76	4	1.008	0.738	0.552	P04003	C4b-binding protein alpha chain
77	4	0.402	0.521	0.688	P07225	Vitamin K-dependent protein S
78	4	0.196	0.766	0.291	P80108	Phosphatidylinositol-glycan-specific phospholipase D
79	4	0.687	0.775	0.676	P50851	Lipopolysaccharide-responsive and beige-like anchor protein
80	3	0.725	1.048	0.798	P0CG06	Ig lambda-3 chain C regions
81	2	0.674	1.009	0.722	B9A064	Immunoglobulin lambda-like polypeptide 5
82	3	0.719	0.865	0.468	P02760	Protein AMBP
83	3	0.921	0.721	0.602	P04004	Vitronectin
84	3	0.773	1.51	0.758	P01594	Ig kappa chain V-I region AU
85	2	0.947	0.943	0.774	P01613	Ig kappa chain V-I region Ni
86	3	0.88	0.846	0.782	P00736	Complement C1r subcomponent
87	3	0.374	0.397	0.336	P02654	Apolipoprotein C-I
88	3	1.177	0.843	0.682	P12259	Coagulation factor V
89	3	1.157	0.758	0.761	O95445	Apolipoprotein M
90	3	0.609	0.786	0.506	P06276	Cholinesterase
91	3	0.982	1.43	0.763	P08603	Complement factor H
92	3	1.496	2.091	0.808	P00915	Carbonic anhydrase 1
93	3	0.524	1.126	0.751	Q6S8J3	POTE ankyrin domain family member E
94	3	0.836	1.124	0.63	Q562R1	Beta-actin-like protein 2
95	3	0.471	0.916	0.702	Q9P2D7	Dynein heavy chain 1, axonemal
96	3	0.699	0.956	0.644	P01880	Ig delta chain C region
97	3	0.722	0.258	0.787	P02743	Serum amyloid P-component
98	3	0.622	1.355	0.467	P00747	Plasminogen
99	3	0.985	1.602	1.241	Q5VST9	Obscurin
100	3	0.584	0.347	0.329	Q14515	SPARC-like protein 1
101	3	2.986	1.617	1.478	P13796	Plastin-2
102	3	0.928	1.096	0.617	Q9NZP8	Complement C1r subcomponent-like protein
103	3	0.749	1.084	0.646	P55058	Phospholipid transfer protein
104	3	0.651	1.53	0.922	Q8WXI7	Mucin-16
105	3	0.736	0.703	0.714	P01619	Ig kappa chain V-III region B6
106	3	0.687	0.628	0.714	Q9UPN3	Microtubule-actin cross-linking factor 1, isoforms 1/2/3/5
107	3	0.858	1.338	0.798	Q96RW7	Hemicentin-1
108	3	0.669	0.703	0.714	Q8TEP8	Centrosomal protein of 192 kDa
109	3	0.687	0.628	0.714	O75592	Probable E3 ubiquitin-protein ligase MYCBP2
110	3	0.847	0.515	0.233	O95613	Pericentrin

111	3	0.733	0.694	0.721	Q13085	Acetyl-CoA carboxylase 1
112	2	0.567	0.479	0.426	P31151	Protein S100-A7
113	2	0.83	1.477	0.816	P01598	Ig kappa chain V-I region EU
114	2	0.443	1.324	1.079	P15169	Carboxypeptidase N catalytic chain
115	2	0.736	0.778	0.872	P01625	Ig kappa chain V-IV region Len
116	2	0.828	1.88	0.989	P43251	Biotinidase
117	2	0.539	0.77	0.798	Q13790	Apolipoprotein F
118	2	0.441	0.622	0.671	P01591	Immunoglobulin J chain
119	2	0.893	0.496	0.546	P31025	Lipocalin-1
120	2	0.869	0.725	0.616	P06702	Protein S100-A9
121	2	0.747	1.177	0.859	P01617	Ig kappa chain V-II region TEW
122	2	0.712	0.5	0.626	O00533	Neural cell adhesion molecule L1-like protein
123	2	0.298	0.466	0.635	P13671	Complement component C6
124	2	1.197	2.435	1.383	Q15582	Transforming growth factor-beta-induced protein ig-h3
125	2	0.259	0.484	0.256	Q14980	Nuclear mitotic apparatus protein 1
126	2	0.665	0.822	0.432	P32119	Peroxiredoxin-2
127	2	0.374	0.35	0.601	Q03001	Dystonin
128	2	0.453	0.78	0.579	Q8NB14	Ubiquitin carboxyl-terminal hydrolase 38
129	2	1.123	1.605	1.026	P01624	Ig kappa chain V-III region POM
130	2	0.969	0.716	1.283	P23142	Fibulin-1
131	2	2.13	1.259	1.067	P33151	Cadherin-5
132	2	2.075	1.07	0.541	Q5D862	Filaggrin-2
133	2	0.391	0.632	0.96	Q15413	Ryanodine receptor 3
134	2	0.774	1.003	0.765	P04180	Phosphatidylcholine-sterol acyltransferase
135	2	0.715	1.067	1.564	Q709C8	Vacuolar protein sorting-associated protein 13C
136	2	0.681	0.982	0.636	Q14997	Proteasome activator complex subunit 4
137	2	0.534	0.554	0.617	Q5KSL6	Diacylglycerol kinase kappa
138	2	0.391	0.632	0.833	Q14204	Cytoplasmic dynein 1 heavy chain 1
139	2	0.707	1.12	1.159	Q12768	WASH complex subunit strumpellin
140	2	1.506	6.114	1.378	P02792	Ferritin light chain
141	2	0.718	0.986	0.748	P78332	RNA-binding protein 6
142	2	0.681	0.537	0.511	B2RTY4	Unconventional myosin-IXa
143	2	0.371	0.581	0.473	O43795	Unconventional myosin-Ib
144	2	6.13	6.453	3.482	P19320	Vascular cell adhesion protein 1
145	2	2.094	0.88	1.543	Q14151	Scaffold attachment factor B2

146	2	1.112	4.665	1.466	P81605	Dermcidin
147	2	2.926	3.737	3.861	Q8IWC1	MAP7 domain-containing protein 3
148	2	0.953	0.408	1.725	Q69YQ0	Cytospin-A
149	2	0.655	1.149	0.862	Q8NDA2	Hemicentin-2
150	2	0.649	0.713	0.462	P55199	RNA polymerase II elongation factor ELL
151	2	0.83	0.828	1.186	O75882	Attractin
152	2	0.706	0.664	0.717	Q9UQE7	Structural maintenance of chromosomes protein 3
153	2	1.022	1.634	0.979	Q12955	Ankyrin-3
154	2	0.706	0.778	0.717	Q5SWA1	Protein phosphatase 1 regulatory subunit 15B
155	2	0.335	0.344	0.252	P20851	C4b-binding protein beta chain
156	2	1.924	1.667	1.356	Q5VYJ5	MAM and LDL-receptor class A domain-containing protein C10orf112
157	2	0.366	0.294	0.321	P05362	Intercellular adhesion molecule 1
158	2	0.489	0.319	0.485	Q5M9N0	Coiled-coil domain-containing protein 158
159	2	1.299	0.365	0.693	P20929	Nebulin
160	2	0.384	0.768	1.356	Q8NF91	Nesprin-1
161	2	0.814	1.759	1.295	Q96PN6	Adenylate cyclase type 10
162	2	0.782	1.09	0.696	Q9UHB7	AF4/FMR2 family member 4
163	2	0.82	1.035	0.862	Q8NB78	Lysine-specific histone demethylase 1B
164	2	0.878	1.299	0.713	Q15678	Tyrosine-protein phosphatase non-receptor type 14
165	2	0.634	0.728	0.585	Q8NCW0	Kremen protein 2
166	2	0.45	1.257	0.886	Q5JST6	EF-hand domain-containing family member C2
167	2	0.749	1.133	0.535	Q14644	Ras GTPase-activating protein 3
168	2	0.312	0.608	1.174	Q9UHB6	LIM domain and actin-binding protein 1
169	2	0.952	1.241	1.298	Q96QV1	Hedgehog-interacting protein
170	2	0.708	0.986	0.646	P46100	Transcriptional regulator ATRX
171	2	0.972	0.619	0.413	Q12772	Sterol regulatory element-binding protein 2
172	2	2.16	1.197	1.578	Q9NQS7	Inner centromere protein
173	2	2.554	1.971	6.122	Q9NRC6	Spectrin beta chain, non-erythrocytic 5
174	2	0.714	1.286	0.798	Q12802	A-kinase anchor protein 13
175	2	1.915	1.035	0.862	Q15746	Myosin light chain kinase, smooth muscle
176	2	2.052	4.196	1.903	Q9ULD5	Zinc finger protein 777

177	2	0.807	0.717	0.997	Q8WWZ3	Ectodysplasin-A receptor-associated adapter protein
178	2	0.166	2.858	1.441	Q9H254	Spectrin beta chain, non-erythrocytic 4
179	2	1.1	1.489	0.868	O42043	HERV-K_1q23.3 provirus ancestral Env polyprotein
180	2	1.173	1.391	1.281	Q9BSQ5	Malcavernin
181	2	0.871	1.941	2.312	Q4KWH8	1-phosphatidylinositol 4,5-bisphosphate phosphodiesterase eta-1
182	2	0.804	0.663	0.267	Q5W041	Armadillo repeat-containing protein 3
183	2	0.376	0.382	0.417	Q9UBJ2	ATP-binding cassette sub-family D member 2
184	2	0.998	1.247	0.59	O75448	Mediator of RNA polymerase II transcription subunit 24
185	2	0.639	0.673	0.436	Q09666	Neuroblast differentiation-associated protein AHNAK
186	2	0.579	0.499	0.792	Q86YR7	Probable guanine nucleotide exchange factor MCF2L2
187	2	0.489	1.232	0.485	Q8IVF2	Protein AHNAK2
188	2	0.58	0.214	0.398	O75140	DEP domain-containing protein 5
189	2	1.844	1.106	1.027	Q92610	Zinc finger protein 592
190	2	0.322	0.485	0.367	P07814	Bifunctional glutamate/proline--tRNA ligase
191	2	0.56	0.909	0.725	P35579	Myosin-9
192	1	0.557	0.65	0.969	P01766	Ig heavy chain V-III region BRO
193	1	1.522	0.643	1.146	P01611	Ig kappa chain V-I region Wes
194	1	2.854	2.387	4.365	P02747	Complement C1q subcomponent subunit C
195	1	1.305	0.654	0.906	P36955	Pigment epithelium-derived factor
196	1	1.359	1.235	1.037	P01774	Ig heavy chain V-III region POM
197	1	1.042	1.556	1.045	P01717	Ig lambda chain V-IV region Hil
198	1	0.462	0.881	0.615	P02765	Alpha-2-HS-glycoprotein
199	1	1.638	1.277	1.218	P04278	Sex hormone-binding globulin
200	1	0.706	0.977	0.706	Q8N6C8	Leukocyte immunoglobulin-like receptor subfamily A member 3
201	1	0.637	0.894	0.676	P35542	Serum amyloid A-4 protein
202	1	0.818	1.091	1.067	P23083	Ig heavy chain V-I region V35
203	1	0.26	0.815	0.401	Q03591	Complement factor H-related protein 1
204	1	0.212	1.483	0.499	P04430	Ig kappa chain V-I region BAN
205	1	1.909	0.646	3.069	Q9UGM5	Fetuin-B
206	1	0.662	0.856	0.713	P04433	Ig kappa chain V-III region VG
207	1	0.74	0.673	0.719	P07359	Platelet glycoprotein Ib alpha chain

208	1	0.59	1.227	0.977	O43866	CD5 antigen-like
209	1	0.44	0.174	1	P02746	Complement C1q subcomponent subunit B
210	1	0.843	1.176	0.302	Q6UXB8	Peptidase inhibitor 16
211	1	0.901	1.363	0.804	P61769	Beta-2-microglobulin
212	1	1.554	0.353	0.592	P33908	Mannosyl-oligosaccharide 1,2-alpha-mannosidase IA
213	1	1.848	1.862	1.414	P07360	Complement component C8 gamma chain
214	1	0.655	0.446	0.781	Q96L93	Kinesin-like protein KIF16B
215	1	0.857	0.982	1.151	P01781	Ig heavy chain V-III region GAL
216	1	0.578	0.957	0.671	Q9Y6A9	Signal peptidase complex subunit 1
217	1	0.941	0.989	0.629	P02745	Complement C1q subcomponent subunit A
218	1	0.622	0.481	0.584	P48740	Mannan-binding lectin serine protease 1
219	1	0.712	0.639	0.776	Q15029	116 kDa U5 small nuclear ribonucleoprotein component
220	1	3	0.791	0.335	Q7Z2Y8	Interferon-induced very large GTPase 1
221	1	1	1	1	P23634	Plasma membrane calcium-transporting ATPase 4
222	1	0.497	0.625	0.568	Q96G01	Protein bicaudal D homolog 1
223	1	1.078	0.511	0.863	Q9C0A6	SET domain-containing protein 5
224	1	0.353	0.394	0.477	O75691	Small subunit processome component 20 homolog
225	1	0.52	1.128	0.878	A6NI56	Coiled-coil domain-containing protein 154
226	1	0.271	0.104	0.391	P03952	Plasma kallikrein
227	1	1.478	0.689	0.593	P10599	Thioredoxin
228	1	0.678	1.214	1.22	P20701	Integrin alpha-L
229	1	0.63	0.687	0.647	O14810	Complexin-1
230	1	1.604	1.735	0.654	O95672	Endothelin-converting enzyme-like 1
231	1	0.292	1.228	0.696	Q16378	Proline-rich protein 4
232	1	0.54	1.041	0.277	P04438	Ig heavy chain V-II region SESS
233	1	0.635	1.061	0.958	Q8NEH6	Meiosis-specific nuclear structural protein 1
234	1	0.589	0.702	0.632	Q9HCJ0	Trinucleotide repeat-containing gene 6C protein
235	1	0.231	0.471	0.273	Q9NPH3	Interleukin-1 receptor accessory protein
236	1	0.539	0.771	0.722	Q8TDS5	Oxoeicosanoid receptor 1
237	1	0.759	0.709	0.997	O75636	Ficolin-3
238	1	0.332	1	1	Q6YHK3	CD109 antigen

239	1	0.759	0.686	1.507	Q08999	Retinoblastoma-like protein 2
240	1	0.774	1.32	0.934	Q14872	Metal regulatory transcription factor 1
241	1	0.754	1.618	1.037	Q12923	Tyrosine-protein phosphatase non-receptor type 13
242	1	0.685	0.675	0.434	P12273	Prolactin-inducible protein
243	1	0.817	2.164	1.42	Q16798	NADP-dependent malic enzyme, mitochondrial
244	1	0.664	1.951	0.863	P08F94	Fibrocystin
245	1	1.88	0.629	1.278	P05156	Complement factor I
246	1	0.459	0.804	0.549	O94874	E3 UFM1-protein ligase 1
247	1	0.514	0.647	0.415	P01780	Ig heavy chain V-III region JON
248	1	0.337	0.196	0.217	P09172	Dopamine beta-hydroxylase
249	1	1.733	2.354	0.943	Q9P273	Teneurin-3
250	1	0.447	0.348	0.557	Q9HB29	Interleukin-1 receptor-like 2
251	1	2.364	0.977	0.625	P48357	Leptin receptor
252	1	0.462	0.813	0.706	Q13243	Serine/arginine-rich splicing factor 5
253	1	0.714	0.866	0.515	Q5T5Y3	Calmodulin-regulated spectrin-associated protein 1
254	1	0.835	0.821	0.608	P40425	Pre-B-cell leukemia transcription factor 2
255	1	1	1	1	Q6XZB0	Lipase member I
256	1	0.615	2.245	0.625	P04434	Ig kappa chain V-III region VH
257	1	0.628	0.799	0.216	P09486	SPARC
258	1	0.551	0.869	0.757	Q8NFM4	Adenylate cyclase type 4
259	1	1.025	0.564	0.707	Q5JTH9	RRP12-like protein
260	1	0.371	0.77	0.782	E7ETH6	Zinc finger protein 587B
261	1	0.343	0.506	0.106	Q08554	Desmocollin-1
262	1	0.391	0.499	0.394	Q3L8U1	Chromodomain-helicase-DNA-binding protein 9
263	1	0.315	0.392	0.27	P61626	Lysozyme C
264	1	1.367	0.734	0.543	P54277	PMS1 protein homolog 1
265	1	0.218	0.126	0.213	P59796	Glutathione peroxidase 6
266	1	0.959	1.1	1.371	O94986	Centrosomal protein of 152 kDa
267	1	6.067	10.8	4.87	Q9NQS3	Poliovirus receptor-related protein 3
268	1	0.308	1.705	1	Q9UI33	Sodium channel protein type 11 subunit alpha
269	1	1	0.23	0.267	Q6EMK4	Vasorin
270	1	1.388	0.363	1	P07357	Complement component C8 alpha chain
271	1	2.488	1.775	3.861	P04196	Histidine-rich glycoprotein
272	1	0.886	1.409	0.696	Q96NY7	Chloride intracellular channel protein 6

273	1	0.334	1.165	0.065	Q02246	Contactin-2
274	1	0.659	0.649	0.559	Q8TE58	A disintegrin and metalloproteinase with thrombospondin motifs 15
275	1	0.148	1.356	0.405	Q9ULE3	DENN domain-containing protein 2A
276	1	1	0.588	0.772	O15020	Spectrin beta chain, non-erythrocytic 2
277	1	0.187	0.673	0.194	Q14789	Golgin subfamily B member 1
278	1	0.229	0.195	0.386	Q9Y490	Talin-1
279	1	0.534	0.37	0.294	Q8NG76	Olfactory receptor 2T33
280	1	0.544	0.538	0.634	O14980	Exportin-1
281	1	0.294	0.134	0.188	O95340	Bifunctional 3'-phosphoadenosine 5'-phosphosulfate synthase 2
282	1	1	1	1	P50224	Sulfotransferase 1A3/1A4
283	1	0.556	0.604	0.672	P57789	Potassium channel subfamily K member 10
284	1	0.462	0.208	0.132	Q8NDM7	WD repeat-containing protein 96
285	1	1	1	1	Q9ULD4	Bromodomain and PHD finger-containing protein 3
286	1	1	1	1	P14866	Heterogeneous nuclear ribonucleoprotein L
287	1	0.44	1.618	0.653	Q9P225	Dynein heavy chain 2, axonemal
288	1	0.539	0.913	1.716	O60494	Cubilin
289	1	1.562	0.888	0.903	O60330	Protocadherin gamma-A12
290	1	2.793	1.352	2.68	P04920	Anion exchange protein 2
291	1	0.236	0.711	1.025	P0CG48	Polyubiquitin-C
292	1	1.089	1	0.722	Q07157	Tight junction protein ZO-1
293	1	0.576	0.822	0.576	Q2LD37	Uncharacterized protein KIAA1109
294	1	0.625	0.958	0.248	A2RUB6	Coiled-coil domain-containing protein 66
295	1	1.097	1.703	0.872	P26232	Catenin alpha-2
296	1	0.646	0.055	0.041	O60568	Procollagen-lysine,2-oxoglutarate 5-dioxygenase 3
297	1	0.475	1.003	0.626	Q16363	Laminin subunit alpha-4
298	1	1.261	1.703	1.087	P86790	Vacuolar fusion protein CCZ1 homolog B
299	1	0.398	0.963	0.566	Q9HCH0	Nck-associated protein 5-like
300	1	1	0.393	1.582	Q9P0L2	Serine/threonine-protein kinase MARK1
301	1	1	1	1	Q9Y2U8	Inner nuclear membrane protein Man1
302	1	0.642	0.878	0.331	Q9BVP2	Guanine nucleotide-binding protein-like 3
303	1	0.339	0.545	0.27	Q14517	Protocadherin Fat 1
304	1	0.163	0.563	1	Q86T65	Disheveled-associated activator of morphogenesis 2

305	1	0.716	0.782	0.147	Q16827	Receptor-type tyrosine-protein phosphatase O
306	1	0.578	0.654	1	Q9NYQ8	Protocadherin Fat 2
307	1	1.236	1.315	0.781	Q8NGT7	Olfactory receptor 2A12
308	1	0.438	0.549	0.582	O95477	ATP-binding cassette sub-family A member 1
309	1	1	1	1	Q0VF96	Cingulin-like protein 1
310	1	1	0.685	1	Q8IWN7	Retinitis pigmentosa 1-like 1 protein
311	1	0.557	0.438	0.559	Q8N196	Homeobox protein SIX5
312	1	1	0.238	1	Q8NBP0	Tetratricopeptide repeat protein 13
313	1	1.112	0.769	0.326	P46940	Ras GTPase-activating-like protein IQGAP1
314	1	4.522	0.687	1.582	Q6R2W3	SCAN domain-containing protein 3
315	1	0.894	0.365	0.675	Q9UKZ4	Teneurin-1
316	1	0.985	1.509	0.281	Q13206	Probable ATP-dependent RNA helicase DDX10
317	1	1	1	0.937	Q9UBV2	Protein sel-1 homolog 1

(IV) Replicate 4

S. No.	No. of unique peptides	Fold-change FEB/HC	Fold-change DEF/HC	Fold-change CON/HC	Accession number	Protein name
1	141	0.723	0.61	0.574	P04114	Apolipoprotein B-100
2	89	0.778	0.854	0.656	P01024	Complement C3
3	49	1.232	1	1.041	P01023	Alpha-2-macroglobulin
4	11	1.055	0.846	0.943	P20742	Pregnancy zone protein
5	43	2.055	1.459	1.786	P01009	Alpha-1-antitrypsin
6	41	1.474	1.205	1.249	P00450	Ceruloplasmin
7	40	0.891	0.673	1.439	P0C0L5	Complement C4-B
8	34	0.566	0.426	0.406	P02647	Apolipoprotein A-I
9	34	1.14	0.765	0.651	P02768	Serum albumin
10	26	1.318	0.945	0.844	P02787	Serotransferrin
11	26	1.459	1.843	1.257	P01011	Alpha-1-antichymotrypsin
12	24	0.673	0.551	0.497	P06727	Apolipoprotein A-IV
13	24	0.806	0.995	0.665	P01008	Antithrombin-III
14	24	0.84	0.724	0.582	Q14624	Inter-alpha-trypsin inhibitor heavy chain H4
15	22	0.638	0.441	0.438	P02751	Fibronectin
20	17	0.489	0.447	0.31	P19823	Inter-alpha-trypsin inhibitor heavy chain H2
21	16	1.622	1.362	1.308	P02774	Vitamin D-binding protein
22	16	1.63	1.178	0.957	P08697	Alpha-2-antiplasmin
23	16	0.682	0.568	1.045	P00738	Haptoglobin
24	15	1.283	1.311	1.344	Q06033	Inter-alpha-trypsin inhibitor heavy chain H3
26	13	1.127	1	1.013	P25311	Zinc-alpha-2-glycoprotein
27	12	0.891	0.835	0.587	P02790	Hemopexin
28	12	0.623	0.502	0.415	P19827	Inter-alpha-trypsin inhibitor heavy chain H1
29	12	0.833	0.587	0.515	P10909	Clusterin
30	12	1.588	1.488	2.221	P01871	Ig mu chain C region
32	11	1.083	0.719	0.674	P05155	Plasma protease C1 inhibitor
33	11	1.105	0.881	0.65	P00751	Complement factor B
34	10	2.266	1.676	1.496	P01857	Ig gamma-1 chain C region
35	8	1.086	0.999	1.137	P01859	Ig gamma-2 chain C region
36	8	1.443	1.53	1.426	P01860	Ig gamma-3 chain C region
37	6	1.741	1.498	1.289	P01861	Ig gamma-4 chain C region
38	10	0.755	0.841	0.596	P43652	Afamin
39	10	1.332	0.906	1.287	P04275	von Willebrand factor
40	10	1.607	1.096	1.001	P02649	Apolipoprotein E
41	10	2.544	1.503	1.87	Q8WZ42	Titin

42	9	0.986	0.843	0.756	P02766	Transthyretin
43	9	1.348	0.984	1.036	P08185	Corticosteroid-binding globulin
44	9	2.228	1.679	1.765	P02763	Alpha-1-acid glycoprotein 1
45	8	2.007	1.82	1.901	P19652	Alpha-1-acid glycoprotein 2
46	9	1.309	1.142	1.534	P01876	Ig alpha-1 chain C region
47	7	1.261	1.123	1.465	P01877	Ig alpha-2 chain C region
48	9	1.113	0.933	0.953	P06681	Complement C2
49	9	1.269	1.041	1.058	P04217	Alpha-1B-glycoprotein
50	8	6.165	2.195	2.488	P68871	Hemoglobin subunit beta
51	7	5.575	2.104	2.365	P02042	Hemoglobin subunit delta
52	2	11.135	2.439	1.221	P02100	Hemoglobin subunit epsilon
53	8	1.586	1.075	1.472	P01019	Angiotensinogen
54	8	0.701	0.513	0.548	P02652	Apolipoprotein A-II
55	8	1.496	0.797	1.015	O14791	Apolipoprotein L1
56	7	3.509	1.964	1.924	P02750	Leucine-rich alpha-2-glycoprotein
57	7	5.202	1.592	1.734	P69905	Hemoglobin subunit alpha
58	7	1.006	0.757	0.522	P51884	Lumican
59	7	1.474	1.226	1.486	P05543	Thyroxine-binding globulin
60	7	1.493	0.807	0.557	Q96KN2	Beta-Ala-His dipeptidase
61	7	0.368	0.447	0.332	P05546	Heparin cofactor 2
62	7	1.323	1.282	1.586	P18428	Lipopolysaccharide-binding protein
63	6	2.59	1.495	1.952	P0DJI8	Serum amyloid A-1 protein
64	6	0.692	0.647	0.441	P01042	Kininogen-1
65	6	0.57	0.766	0.796	P02671	Fibrinogen alpha chain
66	6	1.447	0.912	1.241	Q08380	Galectin-3-binding protein
67	6	4.51	1.818	2.167	P00915	Carbonic anhydrase 1
68	6	0.918	0.74	0.759	P09871	Complement C1s subcomponent
69	6	1.263	1.604	1.438	P19320	Vascular cell adhesion protein 1
70	5	1.49	1.262	1.309	P01834	Ig kappa chain C region
71	5	1.211	1.15	1.217	P02655	Apolipoprotein C-II
72	5	0.709	0.571	0.726	P01031	Complement C5
73	5	0.771	0.428	0.702	P10643	Complement component C7
74	5	0.904	0.991	0.949	P07225	Vitamin K-dependent protein S
75	4	0.91	0.801	1.064	P02656	Apolipoprotein C-III
76	5	1.556	1.194	1.417	B9A064	Immunoglobulin lambda-like polypeptide 5
77	4	1.62	1.317	1.533	P0CG06	Ig lambda-3 chain C regions
78	4	0.695	0.612	0.728	P27169	Serum paraoxonase/arylesterase 1
79	4	0.951	0.632	0.629	P22792	Carboxypeptidase N subunit 2
80	4	1.378	1.134	1.566	P04206	Ig kappa chain V-III region GOL

81	4	0.856	0.766	0.631	P02760	Protein AMBP
82	4	0.706	0.616	0.579	P05090	Apolipoprotein D
83	4	1.392	0.945	0.639	P12259	Coagulation factor V
84	4	3.638	3.942	1.71	P02741	C-reactive protein
85	4	1.32	1.844	2.241	Q15582	Transforming growth factor-beta-induced protein ig-h3
86	4	1.094	1.117	1.248	P06276	Cholinesterase
87	4	1.784	1.342	0.786	P06396	Gelsolin
88	4	1.757	1.08	0.746	P32119	Peroxiredoxin-2
89	4	0.973	0.908	0.961	P02748	Complement component C9
90	4	2.028	5.173	1.015	P02792	Ferritin light chain
91	4	1.254	0.835	0.483	P00747	Plasminogen
92	4	1.105	1.217	1.821	P08603	Complement factor H
93	4	4.685	1.838	0.932	P04196	Histidine-rich glycoprotein
94	4	0.722	0.444	0.616	O95445	Apolipoprotein M
95	4	1.481	1.373	2.509	P13796	Plastin-2
96	4	1.618	1.051	0.708	O43795	Unconventional myosin-Ib
97	4	3.141	1.723	0.758	Q09666	Neuroblast differentiation-associated protein AHNAK
98	4	1.819	0.67	0.435	P20929	Nebulin
99	3	1.327	1.671	1.793	P01593	Ig kappa chain V-I region AG
100	2	1.195	3.097	2.025	P01613	Ig kappa chain V-I region Ni
101	3	1.072	0.98	1.653	P08571	Monocyte differentiation antigen CD14
102	3	1.146	0.806	0.909	P00736	Complement C1r subcomponent
103	3	1.581	1.057	0.841	P04004	Vitronectin
104	3	1.344	1.136	1.575	P22352	Glutathione peroxidase 3
105	3	1.543	1.021	0.554	P15169	Carboxypeptidase N catalytic chain
106	3	1.57	0.664	0.81	P02743	Serum amyloid P-component
107	3	0.88	0.851	0.485	Q6S8J3	POTE ankyrin domain family member E
108	2	1.165	0.666	0.881	Q562R1	Beta-actin-like protein 2
109	3	1.756	1.583	1.886	P01619	Ig kappa chain V-III region B6
110	3	1.602	1.314	0.891	Q9BZZ2	Sialoadhesin
111	3	1.309	1.034	0.837	O75882	Attractin
112	3	0.759	0.666	0.802	O75369	Filamin-B
113	3	2.145	1.365	1.562	P04180	Phosphatidylcholine-sterol acyltransferase
114	3	1.756	1.533	1.541	Q9P2D7	Dynein heavy chain 1, axonemal
115	3	0.621	0.386	0.544	Q03001	Dystonin
116	3	1.964	1.581	1.87	P50851	Lipopolysaccharide-responsive and beige-like anchor protein
117	3	0.523	0.887	0.652	P13671	Complement component C6
118	3	1.835	1.448	1.01	Q9UHP3	Ubiquitin carboxyl-terminal hydrolase 25

119	3	2.053	1.675	1.886	Q9UPN3	Microtubule-actin cross-linking factor 1, isoforms 1/2/3/5
120	3	1.241	0.676	0.904	Q6TFL3	Coiled-coil domain-containing protein 171
121	3	1.222	1.147	1.137	Q0VDD8	Dynein heavy chain 14, axonemal
122	3	0.675	0.551	0.466	Q15413	Ryanodine receptor 3
123	3	0.962	0.692	0.843	Q6KC79	Nipped-B-like protein
124	3	1.816	1.583	1.886	Q9HCR9	Dual 3',5'-cyclic-AMP and -GMP phosphodiesterase 11A
125	3	2.511	1.603	1.744	Q709C8	Vacuolar protein sorting-associated protein 13C
126	3	1.574	0.384	0.162	P00918	Carbonic anhydrase 2
127	3	1.04	0.559	0.827	Q8WXI7	Mucin-16
128	3	1.127	0.909	0.825	P15144	Aminopeptidase N
129	3	1.254	1.14	0.999	Q8NF91	Nesprin-1
130	3	0.732	0.852	0.635	P32189	Glycerol kinase
131	2	1.879	1.581	1.701	P01625	Ig kappa chain V-IV region Len
132	2	1.124	1.323	0.59	P31151	Protein S100-A7
133	2	1.298	0.935	0.748	P02765	Alpha-2-HS-glycoprotein
134	2	1.396	1.882	1.583	P01598	Ig kappa chain V-I region EU
135	2	0.45	0.351	0.374	P02654	Apolipoprotein C-I
136	2	1.672	0.871	2.039	O75015	Low affinity immunoglobulin gamma Fc region receptor III-B
137	2	1.757	1.188	2.648	P08637	Low affinity immunoglobulin gamma Fc region receptor III-A
138	2	1.537	0.918	1.266	Q13790	Apolipoprotein F
139	2	1.403	1.507	1.395	P01614	Ig kappa chain V-II region Cum
140	2	1.853	1.058	0.781	P43251	Biotinidase
141	2	1.695	1.818	1.417	Q9NZP8	Complement C1r subcomponent-like protein
142	2	2.586	0.684	1.945	P07359	Platelet glycoprotein Ib alpha chain
143	2	1.014	0.215	0.712	Q6UXB8	Peptidase inhibitor 16
144	2	1.035	1.213	1.412	P01591	Immunoglobulin J chain
145	2	5.326	0.898	0.732	O00533	Neural cell adhesion molecule L1-like protein
146	2	0.971	0.946	1.081	P07357	Complement component C8 alpha chain
147	2	1.669	1.458	1.641	O43866	CD5 antigen-like
148	2	1.351	0.867	0.921	P04003	C4b-binding protein alpha chain
149	2	3	1.591	0.453	O75556	Mammaglobin-B
150	2	1.229	1.613	1.586	P01624	Ig kappa chain V-III region POM
151	2	0.776	0.759	0.604	P05362	Intercellular adhesion molecule 1
152	2	1.272	0.836	0.924	O43423	Acidic leucine-rich nuclear phosphoprotein 32 family member C
153	2	1.274	0.512	1.454	P33151	Cadherin-5
154	2	1.435	0.973	0.51	Q8NB14	Ubiquitin carboxyl-terminal hydrolase 38
155	2	1.319	1.271	1.065	Q6ZTK2	Putative uncharacterized protein LOC400499

156	2	2.059	1.612	0.976	P81605	Dermcidin
157	2	1.162	1.939	0.641	P01880	Ig delta chain C region
158	2	0.672	0.377	0.338	P80108	Phosphatidylinositol-glycan-specific phospholipase D
159	2	1.52	1.241	1.144	P78332	RNA-binding protein 6
160	2	1.491	1.103	0.836	A6NKG5	Retrotransposon-like protein 1
161	2	1.816	1.581	1.87	Q5VTR2	E3 ubiquitin-protein ligase BRE1A
162	2	0.493	0.774	0.775	O14617	AP-3 complex subunit delta-1
163	2	2.918	1.538	2.99	Q9P2P6	StAR-related lipid transfer protein 9
164	2	1.602	1.182	0.836	P46100	Transcriptional regulator ATRX
165	2	0.603	0.358	0.409	Q9HAV4	Exportin-5
166	2	0.896	5.548	1.943	P35542	Serum amyloid A-4 protein
167	2	1.879	1.583	1.886	Q92782	Zinc finger protein neuro-d4
168	2	1.319	0.903	0.836	Q14644	Ras GTPase-activating protein 3
169	2	1.435	0.973	0.51	Q08378	Golgin subfamily A member 3
170	2	1.879	1.583	1.886	O15078	Centrosomal protein of 290 kDa
171	2	1.964	1.628	1.924	Q5SWA1	Protein phosphatase 1 regulatory subunit 15B
172	2	2.778	1.371	0.829	P00734	Prothrombin
173	2	1.319	0.903	1.065	P24821	Tenascin
174	2	1.783	1.206	1.274	P10266	HERV-K_5q33.3 provirus ancestral Pol protein
175	2	2.104	2.398	1.265	P15924	Desmoplakin
176	2	1.879	1.583	1.886	O14782	Kinesin-like protein KIF3C
177	2	1.332	1.061	1.226	Q5KSL6	Diacylglycerol kinase kappa
178	2	2.053	1.583	1.886	O75113	NEDD4-binding protein 1
179	2	1.804	1.014	1.51	P07384	Calpain-1 catalytic subunit
180	2	2.312	1.427	1.464	P35658	Nuclear pore complex protein Nup214
181	2	1.096	0.683	0.336	O43909	Exostosin-like 3
182	2	0.804	0.694	1.524	Q9NU22	Midasin
183	2	0.493	0.93	0.986	Q8NDA2	Hemicentin-2
184	2	1.07	0.821	1.225	O75445	Usherin
185	2	1.042	1.279	0.856	Q9C0G6	Dynein heavy chain 6, axonemal
186	2	0.986	0.837	0.872	Q12768	WASH complex subunit strumpellin
187	2	1.142	1.613	1.586	Q15051	IQ calmodulin-binding motif-containing protein 1
188	2	0.803	0.689	1.385	P07358	Complement component C8 beta chain
189	2	1.34	0.772	0.821	Q5JST6	EF-hand domain-containing family member C2
190	2	1.208	1.226	0.961	Q8TF20	Zinc finger protein 721
191	2	1.262	0.932	0.642	Q5SW79	Centrosomal protein of 170 kDa
192	2	0.388	1.333	1	Q9BXX0	EMILIN-2
193	2	0.815	0.773	0.841	Q9ULT8	E3 ubiquitin-protein ligase HECTD1

194	2	2.969	0.886	0.644	Q86TI2	Dipeptidyl peptidase 9
195	2	3.509	0.683	0.741	P14923	Junction plakoglobin
196	2	1.193	0.957	0.556	Q9BX63	Fanconi anemia group J protein
197	2	1.058	0.6	0.451	Q9UPW8	Protein unc-13 homolog A
198	2	1.183	0.427	0.567	P23470	Receptor-type tyrosine-protein phosphatase gamma
199	2	0.922	0.934	0.828	P49327	Fatty acid synthase
200	2	0.834	0.634	0.291	Q9H7F0	Probable cation-transporting ATPase 13A3
201	2	0.9	0.932	1.49	Q96RW7	Hemicentin-1
202	2	1.541	0.903	0.868	Q9NYQ6	Cadherin EGF LAG seven-pass G-type receptor 1
203	2	1.094	1.344	1.114	Q8IVF2	Protein AHNAK2
204	2	1.416	1.734	1.143	P11678	Eosinophil peroxidase
205	2	1.917	0.629	1.444	Q12955	Ankyrin-3
206	2	1.081	1.535	0.886	P49792	E3 SUMO-protein ligase RanBP2
207	2	0.906	1.282	0.851	Q9Y6R4	Mitogen-activated protein kinase kinase kinase 4
208	2	1.157	0.88	1.451	Q9Y458	T-box transcription factor TBX22
209	1	1.311	1.142	1.613	P01766	Ig heavy chain V-III region BRO
210	1	1.923	0.898	1.487	P01774	Ig heavy chain V-III region POM
211	1	0.845	2.083	2.526	P04406	Glyceraldehyde-3-phosphate dehydrogenase
212	1	3.274	1.873	1.913	P01717	Ig lambda chain V-IV region Hil
213	1	0.342	0.486	1.168	Q92820	Gamma-glutamyl hydrolase
214	1	1.271	0.758	0.318	Q6EMK4	Vasorin
215	1	2.058	0.682	0.829	P02746	Complement C1q subcomponent subunit B
216	1	0.496	1.968	1.546	P04278	Sex hormone-binding globulin
217	1	0.792	0.916	0.986	P80748	Ig lambda chain V-III region LOI
218	1	2.193	0.972	0.462	Q9UGM5	Fetuin-B
219	1	1.659	1.17	1.389	P04433	Ig kappa chain V-III region VG
220	1	1.471	1.109	1.307	P01781	Ig heavy chain V-III region GAL
221	1	1.261	1.073	1.411	P61769	Beta-2-microglobulin
222	1	1	1	1	Q8N6C8	Leukocyte immunoglobulin-like receptor subfamily A member 3
223	1	1.222	0.954	0.808	Q9UIW2	Plexin-A1
224	1	1.752	1.515	0.562	P09172	Dopamine beta-hydroxylase
225	1	2.043	3.456	1.921	P36955	Pigment epithelium-derived factor
226	1	4.469	1.966	3.082	Q15848	Adiponectin
227	1	5.476	3.608	1.862	P06702	Protein S100-A9
228	1	1.517	1.099	2.333	P04438	Ig heavy chain V-II region SESS
229	1	1.02	1.67	2.397	P55056	Apolipoprotein C-IV
230	1	2.36	3.08	3.62	Q9BYE9	Cadherin-related family member 2
231	1	3.173	2.286	1.32	P13598	Intercellular adhesion molecule 2

232	1	0.836	1.131	0.751	P00742	Coagulation factor X
233	1	0.625	0.2	0.991	P05156	Complement factor I
234	1	1.019	0.684	0.522	P04066	Tissue alpha-L-fucosidase
235	1	1	1	1	P01611	Ig kappa chain V-I region Wes
236	1	0.727	0.246	0.838	P20851	C4b-binding protein beta chain
237	1	1	1	1	P04430	Ig kappa chain V-I region BAN
238	1	1.643	1.213	1.395	P23083	Ig heavy chain V-I region V35
239	1	3.368	0.846	4.326	Q14515	SPARC-like protein 1
240	1	0.735	0.499	0.262	Q10588	ADP-ribosyl cyclase 2
241	1	0.635	0.759	1.853	Q9C0A6	SET domain-containing protein 5
242	1	1.131	0.901	0.836	Q9Y6A9	Signal peptidase complex subunit 1
243	1	1.856	0.969	1.44	P01780	Ig heavy chain V-III region JON
244	1	1.996	0.932	0.988	O75691	Small subunit processome component 20 homolog
245	1	0.872	0.567	0.549	Q9HB29	Interleukin-1 receptor-like 2
246	1	1.197	0.947	1.602	P01767	Ig heavy chain V-III region BUT
247	1	1.589	1.331	1.966	Q96JQ0	Protocadherin-16
248	1	0.996	0.886	0.611	E7ETH6	Zinc finger protein 587B
249	1	1.408	1.059	0.807	Q9Y2H2	Phosphatidylinositide phosphatase SAC2
250	1	1	1	1	P01714	Ig lambda chain V-III region SH
251	1	3.382	0.936	1.26	Q8IYR2	SET and MYND domain-containing protein 4
252	1	1.289	1.166	1.669	A6NI56	Coiled-coil domain-containing protein 154
253	1	0.73	2.127	2.302	P04434	Ig kappa chain V-III region VH
254	1	0.623	0.374	0.468	P31025	Lipocalin-1
255	1	1.159	0.908	1.291	Q9Y6L7	Tolloid-like protein 2
256	1	0.807	0.401	0.535	P05452	Tetranectin
257	1	1.15	0.605	0.851	P02745	Complement C1q subcomponent subunit A
258	1	0.676	0.598	1.304	P02747	Complement C1q subcomponent subunit C
259	1	4.887	1.943	4.714	P16471	Prolactin receptor
260	1	1.735	2.49	1.481	Q4KWH8	1-phosphatidylinositol 4,5-bisphosphate phosphodiesterase eta-1
261	1	1.564	0.909	0.468	P15814	Immunoglobulin lambda-like polypeptide 1
262	1	1.061	0.765	1.08	P12525	Protein L-Myc-2
263	1	12.808	2.617	2.892	Q9P273	Teneurin-3
264	1	0.97	2.293	1.101	Q01459	Di-N-acetylchitobiase
265	1	0.889	1.099	1.515	P14314	Glucosidase 2 subunit beta
266	1	4.216	3.059	1.255	Q3V6T2	Girdin
267	1	7.77	2.015	5.352	Q4L235	Acyl-CoA synthetase family member 4
268	1	2.352	1.675	1.875	Q12923	Tyrosine-protein phosphatase non-receptor type 13
269	1	1.198	0.671	0.163	O00763	Acetyl-CoA carboxylase 2

270	1	1.674	1.03	1.334	Q7Z3Z2	Protein RD3
271	1	1	1	1	P18206	Vinculin
272	1	1.343	0.577	3.742	Q5TCS8	Adenylate kinase domain-containing protein 1
273	1	0.564	0.772	0.957	Q9Y2U8	Inner nuclear membrane protein Man1
274	1	0.342	1	2.466	P28838	Cytosol aminopeptidase
275	1	1.007	0.534	0.608	Q6ZS17	Protein FAM65A
276	1	0.98	0.566	1.742	Q12912	Lymphoid-restricted membrane protein
277	1	1	1	1	P0DJD1	RANBP2-like and GRIP domain-containing protein 2
278	1	1.283	0.482	0.405	Q96BY6	Dedicator of cytokinesis protein 10
279	1	1.61	1	0.672	P46940	Ras GTPase-activating-like protein IQGAP1
280	1	0.921	1.223	1.53	P35443	Thrombospondin-4
281	1	0.272	1.054	0.614	Q9HBE1	POZ-, AT hook-, and zinc finger-containing protein 1
282	1	1	1	1	Q70Z35	Phosphatidylinositol 3,4,5-trisphosphate-dependent Rac exchanger 2 protein
283	1	0.736	1.305	1.514	Q96DR7	Rho guanine nucleotide exchange factor 26
284	1	1.21	0.622	0.541	Q9H254	Spectrin beta chain, non-erythrocytic 4
285	1	0.81	0.699	0.612	P20807	Calpain-3
286	1	2.946	1.247	1.032	Q9NQS7	Inner centromere protein
287	1	1.73	0.969	0.427	Q5VT25	Serine/threonine-protein kinase MRCK alpha
288	1	6.453	1.218	2.278	A6NM62	Leucine-rich repeat-containing protein 53
289	1	0.602	0.693	0.807	Q8TD57	Dynein heavy chain 3, axonemal
290	1	1.171	0.636	0.955	P25789	Proteasome subunit alpha type-4
291	1	7.961	3.42	3.42	P35626	Beta-adrenergic receptor kinase 2
292	1	0.144	0.13	1	P00505	Aspartate aminotransferase, mitochondrial
293	1	1.142	1	0.341	Q86UW6	NEDD4-binding protein 2
294	1	1	1	1	O60841	Eukaryotic translation initiation factor 5B
295	1	1	1	1	Q9UPP2	IQ motif and SEC7 domain-containing protein 3
296	1	2.551	0.58	1	Q96PU9	Outer dense fiber protein 3
297	1	2.402	2.511	0.359	O95935	T-box transcription factor TBX18
298	1	1.189	1.172	0.533	Q96L73	Histone-lysine N-methyltransferase, H3 lysine-36 and H4 lysine-20 specific
299	1	3.123	1	1.752	P12110	Collagen alpha-2(VI) chain
300	1	3.191	3.301	3.425	Q01118	Sodium channel protein type 7 subunit alpha
301	1	1.837	2.483	1.77	Q96Q42	Alsin
302	1	1	1	1	Q9UHR4	Brain-specific angiogenesis inhibitor 1-associated protein 2-like protein 1
303	1	1	1	1	P01612	Ig kappa chain V-I region Mev
304	1	1.875	0.932	1.007	Q8WYA0	Intraflagellar transport protein 81 homolog
305	1	2.256	5.22	2.256	P86790	Vacuolar fusion protein CCZ1 homolog B
306	1	2.736	3.204	1	P53675	Clathrin heavy chain 2

Table S7B. Complete details of protein identification and quantitative iTRAQ data for the longitudinal cohort of vivax malaria patients (compared to HC) obtained from Q-Exactive analysis

S. No.	No. of unique peptides	Fold-change FEB/HC	Fold-change DEF/HC	Fold-change CON/HC	Accession number	Protein name
1	133	0.984	0.549	0.622	C0JYY2	Apolipoprotein B
2	117	1.019	0.614	0.656	P01024	Complement C3
3	76	1.046	0.653	0.66	P01023	Alpha-2-macroglobulin
4	47	1.095	0.501	0.715	B7ZLE5	FN1 protein
5	45	0.818	1.096	0.706	P02768	Serum albumin
6	35	1.762	0.868	0.939	P01009	Alpha-1-antitrypsin
7	35	1.291	0.715	0.8	B2RMS9	Inter-alpha (Globulin) inhibitor H4
8	33	1.42	0.721	0.835	B4E1Z4	Complement factor B
9	31	1.172	0.655	0.774	P01031	Complement C5 OS
10	30	0.976	0.549	0.637	Q5T985	Inter-alpha-trypsin inhibitor heavy chain H2
11	25	1.7	1.552	0.991	P02790	Hemopexin
12	23	1.002	0.597	0.669	P00747	Plasminogen
13	23	0.934	0.548	0.648	A8K5T0	cDNA FLJ75416, highly similar to Homo sapiens complement factor H (CFH), mRNA
14	23	0.554	0.421	0.657	P06727	Apolipoprotein A-IV
15	22	0.966	0.499	0.586	P06396	Gelsolin
16	21	0.953	0.567	0.628	P00734	Prothrombin
17	20	0.952	0.522	0.627	P43652	Afamin
18	19	2.199	0.933	1.18	P01011	Alpha-1-antichymotrypsin
19	17	1.269	0.677	0.716	P01008	Antithrombin-III
20	17	0.401	0.422	0.424	P02647	Apolipoprotein A-I
21	16	1.507	0.774	0.943	Q06033	Inter-alpha-trypsin inhibitor heavy chain H3
22	16	1.053	0.609	0.672	P25311	Zinc-alpha-2-glycoprotein
23	15	1.313	0.708	0.82	B4E1D8	cDNA FLJ51597, highly similar to C4b-binding protein alpha chain
24	13	1.717	1.657	1.506	P02649	Apolipoprotein E
25	13	1.637	0.82	1.013	P02748	Complement component C9
26	13	1.394	0.723	0.837	B4E1H2	Plasma protease C1 inhibitor
27	13	1.026	0.719	0.824	O43866	CD5 antigen-like
28	13	1.059	0.589	0.602	B0AZL7	cDNA, FLJ79457, highly similar to Insulin-like growth factor-binding proteincomplex acid labile chain
29	13	0.945	0.558	0.605	B4E1B2	cDNA FLJ53691, highly similar to Serotransferrin
30	13	0.848	0.475	0.573	B2R8I2	cDNA, FLJ93914, highly similar to Homo sapiens histidine-rich glycoprotein (HRG), mRNA

31	12	1.229	0.674	0.784	G3XAM2	Complement factor I light chain
32	12	0.405	0.623	0.656	P00738	Haptoglobin
33	12	1.133	0.524	0.705	P36955	Pigment epithelium-derived factor
34	11	1.876	1.618	0.797	P04004	Vitronectin
35	11	1.235	0.678	0.807	B4E1B3	cDNA FLJ53950, highly similar to Angiotensinogen
36	11	0.843	0.553	0.596	P05546	Heparin cofactor 2
37	11	0.94	0.537	0.6	P27169	Serum paraoxonase/arylesterase 1
38	11	1.011	0.536	0.692	P03952	Plasma kallikrein
39	11	1.074	0.5	0.686	P10909	Clusterin
40	11	0.916	0.482	0.577	B2R815	cDNA, FLJ93695, highly similar to Homo sapiens serpin peptidase inhibitor, clade A (alpha-1 antiproteinase, antitrypsin)
41	11	0.805	0.421	0.56	D9IWP9	Beta-2-glycoprotein I
42	10	1.155	0.725	0.791	P08185	Corticosteroid-binding globulin
43	10	1.405	0.676	0.817	P08697	Alpha-2-antiplasmin
44	10	0.891	0.528	0.583	P02765	Alpha-2-HS-glycoprotein
45	10	0.839	0.464	0.582	A8K050	cDNA FLJ75376, highly similar to Homo sapiens peptidoglycan recognition protein L
46	9	1.473	0.702	0.841	F8WCZ6	Complement C1s subcomponent
47	9	1.286	0.646	0.795	P22792	Carboxypeptidase N subunit 2
48	9	1.028	0.619	0.721	P04217	Alpha-1B-glycoprotein
49	9	1.204	0.616	0.766	P13671	Complement component C6
50	9	1.099	0.604	0.701	P02760	Protein AMBP
51	9	0.977	0.502	0.612	P51884	Lumican
52	8	1.553	0.795	0.999	P05543	Thyroxine-binding globulin
53	8	1.305	0.633	0.852	A8K5J8	cDNA FLJ75066, highly similar to Homo sapiens complement component 1
54	8	0.974	0.563	0.726	Q16519	Protein S (Fragment)
55	8	0.841	0.486	0.58	P02766	Transthyretin
56	7	2.233	1.068	1.184	P02763	Alpha-1-acid glycoprotein 1
57	7	1.229	0.711	0.826	Q6PIL8	IGK@ protein
58	7	1.323	0.682	0.704	B4DVE1	cDNA FLJ53478, highly similar to Galectin-3-binding protein
59	7	1.178	0.599	0.674	P07357	Complement component C8 alpha chain
60	7	0.811	0.452	0.52	P05090	Apolipoprotein D
61	6	1.715	0.822	0.971	P19652	Alpha-1-acid glycoprotein 2
62	6	0.568	0.783	0.598	P00739	Haptoglobin-related protein
63	6	1.105	0.757	0.879	P01591	Immunoglobulin J chain
64	6	1.194	0.703	0.702	A8K3E4	cDNA FLJ78367, highly similar to Homo sapiens fibrinogen, A alpha polypeptide (FGA)
65	6	0.85	0.566	0.777	Q6ZW64	cDNA FLJ41552 fis, clone COLON2004478, highly similar to Protein

						Tro alpha1 H
66	6	0.51	0.514	0.783	P02652	Apolipoprotein A-II
67	5	8.291	3.503	4.69	P02741	C-reactive protein
68	5	1.761	0.736	0.937	L8E853	von Willebrand factor
69	5	1.707	0.549	0.653	P02743	Serum amyloid P-component
70	5	0.823	0.445	0.639	Q5VY30	Plasma retinol-binding protein(1-182)
71	4	1.66	1.362	0.884	P01860	Ig gamma-3 chain C region
72	4	2.223	1.077	1.191	Q8TCF0	LBP protein
73	4	1.615	0.999	1.151	P01880	Ig delta chain C region
74	4	1.531	0.977	0.898	A2MYD4	V2-7 protein
75	4	5.59	0.852	0.866	Q14474	A-gamma-hemoglobin gene from Greek HPFH mutant,
76	4	1.328	0.801	0.891	B4DDD5	cDNA FLJ58762, highly similar to Phospholipid transfer protein
77	4	1.275	0.751	0.912	A8K5A4	cDNA FLJ76826, highly similar to Homo sapiens ceruloplasmin
78	4	1.371	0.747	0.893	P15169	Carboxypeptidase N catalytic chain
79	4	1.208	0.709	0.709	B7Z7C9	cDNA FLJ50907, highly similar to Biotinidase
80	4	1.186	0.705	0.807	P00748	Coagulation factor XII
81	4	1.178	0.668	0.81	B4DNT5	cDNA FLJ60316, highly similar to Apolipoprotein-L1
82	4	1.332	0.64	0.679	P07360	Complement component C8 gamma chain
83	4	1.289	0.637	0.738	B7Z550	Complement component 8, beta polypeptide
84	4	0.972	0.587	0.674	P20851	C4b-binding protein beta chain
85	4	0.948	0.582	0.646	P05160	Coagulation factor XIII B chain
86	4	0.822	0.577	0.594	P07996	Thrombospondin-1
87	4	0.929	0.551	0.637	D3JV41	Thrombocidin-2 antimicrobial variant
88	4	1.046	0.507	0.584	Q7Z379	Putative uncharacterized protein DKFZp686K04218
89	3	1.976	1	1.131	C8C504	Beta-globin
90	3	1.537	0.941	1.086	P01625	Ig kappa chain V-IV region Len
91	3	1.211	0.803	0.982	P01871	Ig mu chain C region
92	3	4.061	0.725	0.99	E5RH81	Carbonic anhydrase 1
93	3	0.823	0.713	0.756	B2R950	cDNA, FLJ94213, highly similar to Homo sapiens pregnancy-zone protein (PZP)
94	3	1.242	0.712	0.833	P22352	Glutathione peroxidase 3
95	3	1.467	0.702	0.876	B2R888	Monocyte differentiation antigen CD14
96	3	1.225	0.702	0.8	Q6PIK1	IGL@ protein OS
97	3	1.04	0.698	0.843	Q14520	Hyaluronan-binding protein 2
98	3	1.217	0.676	0.802	D3DPF9	Titin, isoform CRA_b
99	3	1.219	0.665	0.798	E7EQ48	Proteoglycan 4
100	3	1.089	0.662	0.711	F8WDX4	Complement factor H

101	3	0.927	0.651	0.647	P0C0L4	Complement C4-A
102	3	1.12	0.642	0.767	P00742	Coagulation factor X
103	3	1.125	0.629	0.787	B1AKG0	Complement factor H-related protein 1
104	3	0.843	0.623	0.743	P02655	Apolipoprotein C-II
105	3	1.126	0.581	0.695	B4DQI1	Complement C2b fragment
106	3	0.652	0.472	0.597	P02656	Apolipoprotein C-III
107	3	0.605	0.427	0.501	P02776	Platelet factor 4
108	3	0.775	0.378	0.502	P80108	Phosphatidylinositol-glycan-specific phospholipase D
109	3	0.49	0.355	0.43	Q5SRP5	Apolipoprotein M
110	3				Q5DT20	Hornerin
111	2	2.06	1.928	1.494	A2MYC8	V5-2 protein (Fragment)
112	2	1.205	1.769	1.14	Q6J1Z9	Hemoglobin alpha 1
113	2	2.65	1.668	0.592	B2R9V7	Superoxide dismutase [Cu-Zn]
114	2	1.67	1.059	1.12	A2MYD0	V1-17 protein (Fragment)
115	2	1.29	0.993	1.053	P01703	Ig lambda chain V-I region NEWM
116	2	2.404	0.993	1.212	Q6FHW3	DF protein
117	2	1.171	0.927	0.724	P0C0L5	Complement C4-B
118	2	1.408	0.849	1.095	A2NB45	Cold agglutinin FS-1 L-chain (Fragment)
119	2	1.333	0.818	0.857	Q9UL88	Myosin-reactive immunoglobulin heavy chain variable region (Fragment)
120	2	2.771	0.807	1.107	Q4TZM4	Hemoglobin beta chain
121	2	1.578	0.804	0.904	Q9UK55	Protein Z-dependent protease inhibitor
122	2	1.277	0.796	0.784	P01766	Ig heavy chain V-III region BRO
123	2	2.622	0.79	1.082	P02042	Hemoglobin subunit delta
124	2	1.336	0.784	0.77	H0Y8L3	Transforming growth factor-beta-induced protein ig-h3
125	2	1.279	0.774	0.898	Q96JD0	Amyloid lambda 6 light chain variable region SAR
126	2	1.617	0.735	1.319	D6RF35	Vitamin D-binding protein
127	2	1.156	0.731	0.691	D1CS68	TLR7
128	2	1.389	0.719	0.803	A0N5G1	Rheumatoid factor C6 light chain
129	2	1.61	0.717	0.905	Q16610	Extracellular matrix protein 1
130	2	1.133	0.715	0.773	Q9ULB6	Imunoglobulin heavy chain
131	2	1.205	0.705	0.857	S6BGD6	IgG L chain
132	2	1.376	0.702	0.89	I3L1U9	Actin, cytoplasmic 2, N-terminally processed
133	2	1.396	0.698	0.788	Q6UY50	NL3
134	2	1.166	0.694	0.804	A0N5G5	Rheumatoid factor D5 light chain
135	2	1.911	0.669	1.743	Q5NV83	V3-3 protein
136	2	1.073	0.662	0.553	O00300	Tumor necrosis factor receptor superfamily member 11B
137	2	1.283	0.657	0.812	P26927	Hepatocyte growth factor-like protein

138	2	0.858	0.6	0.613	I3L145	Sex hormone-binding globulin
139	2	0.959	0.586	0.683	B4DI57	cDNA FLJ54111, highly similar to Serotransferrin
140	2	1.07	0.571	0.567	B4DZ36	cDNA FLJ58441, highly similar to Attractin
141	2	1.281	0.567	1.17	A0M8Q6	Ig lambda-7 chain C region
142	2	1.172	0.563	0.756	Q8NBH6	cDNA PSEC0266 fis, clone NT2RP3003649, highly similar to Homo sapiens fibulin-1D
143	2	0.972	0.549	0.601	Q96IY4	Carboxypeptidase B2
144	2	1.011	0.546	0.609	A6XND1	Insulin-like growth factor binding protein 3 isoform b
145	2	0.889	0.542	0.664	B4E1C4	cDNA FLJ51179, highly similar to Vitamin K-dependent protein C (EC 3.4.21.69)
146	2	1.053	0.538	0.62	B2R582	cDNA, FLJ92374, highly similar to Homo sapiens C-type lectin domain family 3, member B (CLEC3B)
147	2	1.325	0.51	0.788	B4E1C2	Kininogen 1, isoform CRA_b
148	2	0.721	0.465	0.552	P02774	Vitamin D-binding protein
149	2	0.897	0.463	0.549	D6REX5	Selenoprotein P
150	2	0.965	0.414	0.628	B4E180	cDNA FLJ61201, highly similar to Beta-Ala-His dipeptidase (EC 3.4.13.20)
151	2	0.706	0.407	0.551	K7ERI9	Truncated apolipoprotein C-I
152	2				P81605	Dermcidin
153	2	1.846	1.679	0.699	P0DJI9	Serum amyloid A-2 protein
154	1	1.919	1.784	1.547	D3DQX7	Serum amyloid A protein
155	1	3.653	1.562	1.866	Q9UJX1	Alanine-glyoxylate aminotransferase homolog
156	1	1.54	1.394	1.178	Q5NV91	V2-19 protein
157	1	1.247	1.301	1.07	H0YMW5	Proprotein convertase subtilisin/kexin type 6
158	1	1.689	1.231	1.055	B7Z2I6	cDNA FLJ57106, highly similar to Transferrin receptor protein 1
159	1	0.882	1.226	0.667	S6BGE0	IgG H chain
160	1	1.803	1.206	0.794	B4DKK0	cDNA FLJ52511, highly similar to Abhydrolase domain-containing protein 14B
161	1	1.55	1.195	1.456	Q05BW3	NES protein
162	1	1.057	1.192	0.784	A2MYD6	V1-2 protein
163	1	1.889	1.148	1.236	P02750	Leucine-rich alpha-2-glycoprotein
164	1	2.43	1.116	1.241	Q9UPS8	Ankyrin repeat domain-containing protein 26
165	1	1.648	1.108	0.759	Q9H5S1	cDNA: FLJ23121 fis, clone LNG07996
166	1	1.567	1.093	1.238	P04220	Ig mu heavy chain disease protein
167	1	1.483	1.091	0.864	Q50KD6	Taste receptor type 2
168	1	1.81	1.06	1.015	O95327	Putative uncharacterized protein
169	1	0.533	1.029	0.591	C9J8U1	Cytospin-A
170	1	1.049	1.014	1.158	Q86TT1	Full-length cDNA clone CS0DD006YL02 of Neuroblastoma of Homo sapiens

171	1	1.389	1.007	1.009	F5GXS5	Apolipoprotein F
172	1	3.443	0.995	0.918	P69905	Hemoglobin subunit alpha
173	1	1.499	0.992	1.18	A2NB44	Cold agglutinin FS-2 H-chain
174	1	1.768	0.986	1.105	Q9NP74	Palmdelphin OS
175	1	1.31	0.976	1.055	B2RCH7	cDNA, FLJ96082, highly similar to Homo sapiens cervical cancer 1 protooncogene (HCCR1)
176	1	0.985	0.968	0.593	P21817	Ryanodine receptor 1
177	1	2.139	0.966	1.25	Q9UL83	Myosin-reactive immunoglobulin light chain variable region
178	1	1.783	0.96	1.144	Q5NV84	V1-3 protein
179	1	1.405	0.952	0.996	Q96SA9	Anti-streptococcal/anti-myosin immunoglobulin kappa light chain variable region
180	1	1.515	0.945	0.619	B3KUS6	Serine/threonine-protein kinase MAK
181	1	1.973	0.942	0.89	Q3ZCU5	DLG4 protein
182	1	1.299	0.929	0.786	A2NUT2	Lambda-chain (AA -20 to 215)
183	1	1.571	0.924	0.973	P01814	Ig heavy chain V-II region OU
184	1	2.007	0.921	0.291	Q8N7X4	Melanoma-associated antigen B6
185	1	1.352	0.919	0.899	Q9H804	cDNA FLJ14022 fis, clone HEMBA1003538, weakly similar to COMPLEMENT C1R COMPONENT (EC 3.4.21.41)
186	1	1.147	0.907	0.721	Q5NV75	V2-15 protein (Fragment)
187	1	0.915	0.894	0.902	L0R8D2	Alternative protein RCOR1
188	1	1.34	0.894	1.066	P16157	Ankyrin-1
189	1	1.758	0.884	1.045	A2J1N5	Rheumatoid factor RF-ET6
190	1	1.204	0.871	0.817	Q6GMX6	IGH@ protein
191	1	1.261	0.869	0.982	Q59GK2	N-deacetylase/N-sulfotransferase (Heparan glucosaminyl) 1 variant
192	1	1.078	0.859	0.877	H0YBJ8	Protein LYRIC
193	1	1.577	0.853	0.89	P12273	Prolactin-inducible protein
194	1	1.194	0.846	0.979	E5FY30	Glycoprotein Ib
195	1	1.52	0.845	0.821	M0R126	Uncharacterized protein
196	1	0.862	0.845	0.696	Q76B58	BMP/retinoic acid-inducible neural-specific protein 3
197	1	1.352	0.845	0.785	H0YFN7	Protein RIC1 homolog
198	1	2.179	0.842	1.833	Q9HCC1	Single chain Fv
199	1	1.374	0.842	0.923	Q8N355	IGL@ protein
200	1	1.557	0.841	1.045	Q9UL85	Myosin-reactive immunoglobulin kappa chain variable region
201	1	1.35	0.836	0.776	Q65ZC9	Single-chain Fv
202	1	1.118	0.835	0.971	Q8TE73	Dynein heavy chain 5, axonemal
203	1	1.249	0.835	0.749	B2R6W1	cDNA, FLJ93143, highly similar to Homo sapiens complement component 7 (C7)

204	1	1.263	0.83	0.915	A2IPI6	HRV Fab 027-VL
205	1	1.402	0.828	0.867	Q02224	Centromere-associated protein E
206	1	0.997	0.827	0.962	A8KA24	cDNA FLJ78648, highly similar to Homo sapiens vacuolar protein sorting 54 (yeast) (VPS54), transcript variant 2
207	1	2.082	0.824	1.07	H2B4M3	LILRA3 protein
208	1	1.531	0.817	0.97	Q0ZCH6	Immunglobulin heavy chain variable region
209	1	1.316	0.816	0.789	Q52LL2	Protocadherin gamma subfamily A, 10
210	1	1.955	0.816	0.865	U3KQ89	Protein FAM35A
211	1	1.322	0.801	0.873	P01621	Ig kappa chain V-III region NG9
212	1	1.524	0.799	1.092	B2R8A2	cDNA, FLJ93804, highly similar to Homo sapiens gp25L2 protein (HSGP25L2G),
213	1	1.262	0.798	0.873	P06331	Ig heavy chain V-II region ARH-77
214	1	1.497	0.796	0.804	A2KBB9	Anti-(ED-B) scFV
215	1	1.232	0.788	0.679	Q92782	Zinc finger protein neuro-d4
216	1	1.214	0.786	0.809	P01603	Ig kappa chain V-I region Ka
217	1	1.172	0.783	0.86	Q13789	Apolipoprotein B
218	1	1.147	0.782	0.685	A2NYQ9	Anti-folate binding protein
219	1	1.43	0.776	0.83	P0CW18	Serine protease 56
220	1	1.296	0.776	0.87	Q9P084	HSPC298
221	1	1.3	0.775	0.868	P01762	Ig heavy chain V-III region TRO
222	1	3.402	0.774	3.263	C9JDS9	Tubulin alpha-4A chain
223	1	1.036	0.773	0.644	Q68E01	Integrator complex subunit 3
224	1	1.224	0.772	0.863	H0YLF3	Beta-2-microglobulin form pI 5.3
225	1	1.597	0.772	0.833	B4DQA0	cDNA FLJ57560, highly similar to L-selectin
226	1	1.866	0.766	0.613	S4R3K6	Lebercilin
227	1	1.285	0.763	0.735	Q5NV92	V5-6 protein
228	1	1.244	0.763	0.946	Q9UL70	Myosin-reactive immunoglobulin light chain variable region
229	1	1.263	0.762	0.822	H0Y7Y8	AT-rich interactive domain-containing protein 4B
230	1	1.632	0.757	0.677	Q9UL90	Myosin-reactive immunoglobulin heavy chain variable region
231	1	1.229	0.757	0.72	P01714	Ig lambda chain V-III region SH
232	1	1.117	0.752	0.712	B3KX47	cDNA FLJ44733 fis, clone BRACE3026290, highly similar to Lethal(2) giant larvae protein homolog 2
233	1	1.159	0.749	0.851	B4DYS8	cDNA FLJ55913, highly similar to Cohesin subunit SA-3
234	1	1.083	0.741	0.864	Q5NV65	V1-5 protein
235	1	1.481	0.74	1.073	Q9NZP8	Complement C1r subcomponent-like protein
236	1	1.166	0.739	0.849	C9J610	Uncharacterized protein C20orf26
237	1	1.494	0.736	0.789	A8K449	cDNA FLJ76787, highly similar to Homo sapiens chloride channel Ka (CLCNKA)

238	1	1.288	0.736	0.847	D6RA08	Complement C1q subcomponent subunit B
239	1	1.179	0.734	0.796	K9LC28	MHC class I antigen
240	1	1.124	0.733	0.67	Q5TB80	Centrosomal protein of 162 kDa
241	1	1.483	0.728	0.78	Q6ZP26	CDNA FLJ26672 fis, clone MPG03403, highly similar to Ligatin
242	1	1.462	0.727	0.732	Q68CK4	Leucine-rich alpha-2-glycoprotein
243	1	1.252	0.723	0.882	Q68BL8	Olfactomedin-like protein 2B
244	1	1.794	0.723	0.709	P35542	Serum amyloid A-4 protein
245	1	1.416	0.723	0.913	A2J1N0	Rheumatoid factor RF-IP14
246	1	1.402	0.713	1.132	A2JA19	Anti-mucin1 light chain variable region
247	1	1.569	0.712	0.911	A6NJ16	Putative V-set and immunoglobulin domain-containing-like protein IGHV4OR15-8
248	1	2.331	0.712	0.791	B4DF70	cDNA FLJ60461, highly similar to Peroxiredoxin-2 (EC 1.11.1.15)
249	1	1.139	0.71	0.708	Q96P70	Importin-9
250	1	1.344	0.709	0.897	P11226	Mannose-binding protein C
251	1	1.428	0.704	1.021	Q05CH0	SOX11 protein
252	1	1.233	0.701	0.842	A2N0T4	VH6DJ protein
253	1	1.163	0.697	0.578	O95714	E3 ubiquitin-protein ligase HERC2
254	1	1.56	0.695	0.595	Q9Y5Y7	Lymphatic vessel endothelial hyaluronic acid receptor 1
255	1	0.651	0.694	0.525	P51805	Plexin-A3
256	1	1.437	0.692	1.232	A8MSZ8	Obscurin-like protein 1
257	1	1.285	0.692	1.013	B7Z539	cDNA FLJ56954, highly similar to Inter-alpha-trypsin inhibitor heavy chain H1
258	1	1.204	0.691	0.687	A2J1M2	Rheumatoid factor RF-IP9
259	1	1.376	0.69	0.736	Q562M3	Actin-like protein
260	1	1.022	0.689	0.728	A8K2P0	cDNA FLJ75914, highly similar to Homo sapiens leucine rich repeat containing 17
261	1	1.297	0.687	0.772	F5H793	L-lactate dehydrogenase B chain
262	1	1.066	0.686	0.807	P04053	DNA nucleotidylexotransferase
263	1	1.531	0.685	0.888	Q5W0A0	Protein FAM194B
264	1	1.094	0.682	0.617	Q8IVF2	Protein AHNAK2
265	1	1.126	0.68	0.742	B4E344	cDNA FLJ54406, highly similar to Complement C4-B
266	1	1.143	0.674	0.634	A5YAK2	Apolipoprotein C-IV
267	1	1.391	0.67	0.824	P01613	Ig kappa chain V-I region Ni
268	1	1.052	0.667	1.04	Q59GU3	Voltage-dependent L-type calcium channel alpha-1C subunit variant
269	1	1.189	0.667	0.819	H3BTL3	Uncharacterized protein C15orf41
270	1	1.012	0.665	0.743	Q04756	Hepatocyte growth factor activator
271	1	1.121	0.663	0.746	B4DPP8	cDNA FLJ53075, highly similar to Kininogen-1
272	1	1.932	0.651	0.702	Q75MW2	Protein ZNF767

273	1	1.268	0.643	0.858	B3KWB5	cDNA FLJ42722 fis, clone BRAMY4000277, highly similar to Alpha-1B-glycoprotein
274	1	1.126	0.641	0.641	B3KXB6	cDNA FLJ45101 fis, clone BRAWH3032298, highly similar to Tenascin-N
275	1	1.157	0.637	0.737	E7EUY0	DNA-dependent protein kinase catalytic subunit
276	1	1.279	0.637	0.898	Q9UEJ6	Cadherin
277	1	1.42	0.634	1.079	Q5NV89	V1-7 protein
278	1	1.142	0.629	0.644	H7C0U4	EF-hand domain-containing family member B
279	1	0.838	0.628	0.681	P31151	Protein S100-A7
280	1	1.148	0.626	0.807	I6L976	Chromosome 6 open reading frame 57
281	1	1.231	0.621	0.838	B7Z8T3	Fetuin-B
282	1	1.054	0.621	0.564	P78492	Inter-alpha-trypsin inhibitor
283	1	1.326	0.62	0.725	F8WEX7	Cholinesterase
284	1	0.996	0.62	0.604	P0DJI8	Serum amyloid A-1 protein
285	1	0.898	0.619	0.722	H0YHI9	Myelin regulatory factor-like protein
286	1	1.145	0.619	0.771	P02747	Complement C1q subcomponent subunit C
287	1	1.532	0.618	0.936	A8K477	cDNA FLJ78571, highly similar to Homo sapiens sulfhydryl oxidase mRNA
288	1	1.241	0.613	0.614	Q9BRX8	Redox-regulatory protein FAM213A
289	1	1.228	0.611	0.698	A0NA61	Codes for truncated alpha mRNA of alpha heavy chain disease patient LTE
290	1	0.98	0.611	0.793	B9EK43	Cyclic nucleotide gated channel beta 3
291	1	1.017	0.608	0.646	B2R5G8	Serum amyloid A protein
292	1	1.011	0.605	0.577	B7Z768	cDNA FLJ52138, highly similar to NADH dehydrogenase (ubiquinone) 1 alpha subcomplex subunit 8 (EC 1.6.5.3)
293	1	1.209	0.604	0.536	A8K6N7	cDNA FLJ78671
294	1	1.035	0.603	0.601	G3V264	Plasma serine protease inhibitor
295	1	1.164	0.602	0.637	H7C0B6	Villin-1
296	1	2.211	0.599	1.225	B1AKC9	Ephrin type-B receptor 2
297	1	1.19	0.599	0.706	P01608	Ig kappa chain V-I region Roy
298	1	1.5	0.598	0.775	C1PHC3	CD44 molecule
299	1	1.287	0.596	0.802	A8K2T4	cDNA FLJ78207, highly similar to Human complement protein component C7 mRNA
300	1	1.252	0.589	0.548	Q59HB3	Apolipoprotein B variant
301	1	1.076	0.589	0.611	P01620	Ig kappa chain V-III region SIE
302	1	1.056	0.587	0.58	Q9NV70	Exocyst complex component 1
303	1	1.219	0.584	0.551	P01611	Ig kappa chain V-I region Wes
304	1	1.041	0.579	0.72	Q5GIA7	Antigen MU-RMS-40.16A
305	1	1.074	0.578	0.678	E9PLD0	Ras-related protein Rab-1B

306	1	0.875	0.577	0.955	B4DNL9	cDNA FLJ55853, highly similar to Protein FAM45A
307	1	0.978	0.577	0.665	B3KX29	cDNA FLJ44554 fis, clone UTERU3007419, highly similar to Calcium-dependent secretion activator 2
308	1	0.5	0.576	0.696	H0YE32	AMP deaminase 2
309	1	0.99	0.572	0.635	B7Z1F8	cDNA FLJ53025, highly similar to Complement C4-B
310	1	1.321	0.565	0.63	Q59FV9	PTK7 protein tyrosine kinase 7 isoform a variant
311	1	0.639	0.563	0.497	H0YMC0	Uncharacterized protein C15orf57
312	1	2.077	0.562	0.754	H0YJC6	Processed lymphoid-restricted membrane protein
313	1	1.402	0.553	0.921	P01619	Ig kappa chain V-III region B6
314	1	0.794	0.553	0.707	B4DS03	cDNA FLJ51060, weakly similar to Sorting nexin-6
315	1	1.069	0.552	0.639	D3DX33	HCG1999854, isoform CRA_a
316	1	1.111	0.552	0.791	C9J7L2	Replication initiator 1
317	1	0.883	0.539	0.565	O43825	Beta-1,3-galactosyltransferase 2
318	1	1.392	0.53	0.839	Q15485	Ficolin-2
319	1	0.897	0.529	0.523	B4DKF7	cDNA FLJ59244, highly similar to Homo sapiens cyclin M1 (CNNM1)
320	1	1.02	0.526	0.674	Q19UI2	Truncated coagulation factor IX
321	1	1.134	0.521	0.797	Q5SVK8	Dynamin-binding protein
322	1	0.623	0.513	0.521	H7BZG9	Glutathione S-transferase theta-1
323	1	0.997	0.513	0.63	B7Z549	cDNA FLJ56821, highly similar to Inter-alpha-trypsin inhibitor heavy chain H1
324	1	0.974	0.506	0.873	Q66K41	Zinc finger protein 385C
325	1	0.884	0.501	0.571	Q6N095	Putative uncharacterized protein DKFZp686K03196
326	1	0.804	0.501	0.589	A0A5E4	Uncharacterized protein
327	1	0.833	0.491	0.658	B2RAK1	cDNA, FLJ94965, highly similar to Homo sapiens leucyl/cystinyl aminopeptidase (LNPEP)
328	1	0.845	0.479	0.577	Q70EL4	Ubiquitin carboxyl-terminal hydrolase 43
329	1	0.724	0.475	0.677	B4DYE2	cDNA FLJ61467, highly similar to Chromosome-associated kinesin KIF4A
330	1	0.83	0.465	0.747	S4R394	Uncharacterized protein
331	1	1.058	0.46	0.579	Q4KMQ8	C-terminal binding protein 1
332	1	0.571	0.456	0.554	Q96HN9	Putative uncharacterized protein
333	1	0.952	0.456	0.731	D3DV75	Adenosine deaminase, RNA-specific, isoform CRA
334	1	1.105	0.445	0.843	B1ANW7	Nebulin-related-anchoring protein
335	1	0.778	0.435	0.658	C9J6N9	Ubiquitin fusion degradation protein 1 homolog
336	1	1.2	0.428	0.547	F1T0K4	DmX-like protein 1

337	1	0.376	0.375	0.374	U3TJK0	ATP-binding cassette, sub-family B (MDR/TAP), member 6
338	1	1.274	0.266	0.912	H7BYX6	Neural cell adhesion molecule 1
339	1	0.281	0.266	NI	Q0D2N8	SARM1 protein
340	1	1.563	0.238	0.846	E9PFZ2	Ceruloplasmin
341	1	1.471	NI	NI	H7C501	Protein KRBA1
342	1	1.218	NI	1.265	F8VZA8	Methyltransferase-like protein 25

Table S8A. Differentially abundant proteins in the longitudinal cohort of vivax malaria patients (compared to HC) identified by iTRAQ-based quantitative proteomics analysis using Q-TOF mass spectrometer

(I) Differentially abundant proteins in FEB stage

Sl No.	Uniprot_ID	Gene Name	Protein names	Fold-change (FEB/HC)	Adjusted *p*-value
1	P02647	APOA1	Apolipoprotein A-I	0.42	0.0011
2	P68871	HBB	Hemoglobin subunit beta	2.70	0.0028
3	P02654	APOC1	Apolipoprotein C-I	0.45	0.0029
4	P02741	CRP	C-reactive protein	3.91	0.0030
5	P02750	LRG1	Leucine-rich alpha-2-glycoprotein	1.83	0.0046
6	P05546	SERPIND1	Heparin cofactor 2	0.44	0.0048
7	P02042	HBD	Hemoglobin subunit delta	3.77	0.0053
8	P69905	HBA1	Hemoglobin subunit alpha	2.63	0.0061
9	P19823	ITIH2	Inter-alpha-trypsin inhibitor heavy chain H2	0.49	0.0078
10	P0DJI8	SAA1	Serum amyloid A-1 protein	2.49	0.0084
11	P02008	HBZ	Hemoglobin subunit zeta	4.83	0.0109
12	P00915	CA1	Carbonic anhydrase 1	2.61	0.0136
13	P19827	ITIH1	Inter-alpha-trypsin inhibitor heavy chain H1	0.65	0.0214
14	P00738	HP	Haptoglobin	0.47	0.0244
15	P02751	FN1	Fibronectin	0.72	0.0299
16	P04114	APOB	Apolipoprotein B-100	0.77	0.0299
17	P02656	APOC3	Apolipoprotein C-III	0.68	0.0314
18	P43652	AFM	Afamin	0.67	0.0327
19	P01011	SERPINA3	Alpha-1-antichymotrypsin	1.42	0.0364
20	Q14624	ITIH4	Inter-alpha-trypsin inhibitor heavy chain H4	0.72	0.0430
21	P43251	BTD	Biotinidase	1.30	0.0453
22	P02652	APOA2	Apolipoprotein A-II	0.41	0.0468
23	P80108	GPLD1	Phosphatidylinositol-glycan-specific phospholipase D	0.63	0.0503
24	P02649	APOE	Apolipoprotein E	1.24	0.0505
25	P02790	HPX	Hemopexin	1.22	0.051

(II) Differentially abundant proteins in DEF stage

Sl No.	Uniprot_ID	Gene	Protein names	Fold-change (DEF/HC)	Adjusted *p*-value
1	P02654	APOC1	Apolipoprotein C-I	0.40	3.3504E-^{05}
2	P19827	ITIH1	Inter-alpha-trypsin inhibitor heavy chain H1	0.45	0.0001
3	P05546	SERPIND1	Heparin cofactor 2	0.39	0.0014
4	P04114	APOB	Apolipoprotein B-100	0.63	0.0016
5	P19823	ITIH2	Inter-alpha-trypsin inhibitor heavy chain H2	0.41	0.0031
6	P10909	CLU	Clusterin	0.68	0.0032
7	P01009	SERPINA1	Alpha-1-antitrypsin	1.51	0.0040
8	P02751	FN1	Fibronectin	0.58	0.0052
9	P02741	CRP	C-reactive protein [Cleaved into: C-reactive protein	6.69	0.0056
10	P01042	KNG1	Kininogen-1	0.63	0.0060
11	P27169	PON1	Serum paraoxonase/arylesterase 1	0.54	0.0072
12	P02647	APOA1	Apolipoprotein A-I	0.48	0.0076
13	P0C0L4	C4A	Complement C4-A	0.73	0.0137
14	Q9H9E3	COG4	Conserved oligomeric Golgi complex subunit 4	0.55	0.0180
15	P02008	HBZ	Hemoglobin subunit zeta	1.88	0.0228
16	P0DJI8	SAA1	Serum amyloid A-1 protein	1.60	0.0232
17	P02743	APCS	Serum amyloid P-component	0.62	0.0246
18	P69905	HBA1;	Hemoglobin subunit alpha	1.49	0.0247
19	P01024	C3	Complement C3	0.66	0.0315
20	P02750	LRG1	Leucine-rich alpha-2-glycoprotein	1.63	0.0369
21	P01610	IGKV1-17	Immunoglobulin kappa variable 1-17	0.78	0.0369
22	P01031	C5	Complement C5	0.67	0.0385
23	P02763	ORM1	Alpha-1-acid glycoprotein 1	1.49	0.0406
24	P02656	APOC3	Apolipoprotein C-III	0.68	0.0412
25	Q9NX58	LYAR	Cell growth-regulating nucleolar protein	2.10	0.0439
26	P02649	APOE	Apolipoprotein E	1.16	0.0455
27	P00738	HP	Haptoglobin	0.68	0.0489
28	Q14624	ITIH4	Inter-alpha-trypsin inhibitor heavy chain H4	0.63	0.0556

(III) Differentially abundant proteins in CON stage

Sl No.	Uniprot_ID	Gene names	Protein names	Fold-change (CON/HC)	Adjusted *p*-value
1	P02654	APOC1	Apolipoprotein C-I	0.46	0.0002
2	P02741	CRP PTX1	C-reactive protein [Cleaved into: C-reactive protein	2.45	0.0010
3	P01610	IGKV1-17	Immunoglobulin kappa variable 1-17	0.58	0.0014
4	P80108	GPLD1 PIGPLD1	Phosphatidylinositol-glycan-specific phospholipase D	0.44	0.0065

(IV) Distinctive and overlapping differentially abundant proteins in FEB, DEF and CON stages of vivax malaria

**Proteins differentially abundant only in FEB stage**	**Proteins differentially abundant only in DEF stage**	**Proteins differentially abundant in FEB and DEF stages**	**Proteins differentially abundant in FEB and CON stages**	**Proteins differentially abundant in DEF and CON stages**	**Proteins differentially abundant in FEB, DEF and CON stages**
Hemoglobin subunit delta (P02042)	Cell growth-regulating nucleolar protein (Q9NX58)	Hemoglobin subunit zeta (P02008)	Phosphatidylinositol-glycan-specific phospholipase D (P80108)	Immunoglobulin kappa variable 1-17 (P01610)	Apolipoprotein C-I (P02654)
Hemoglobin subunit beta (P68871)	Alpha-1-antitrypsin (P01009)	Hemoglobin subunit alpha (P69905)			C-reactive protein (P02741)
Carbonic anhydrase 1 (P00915)	Alpha-1-acid glycoprotein 1 (P02763)	Serum amyloid A-1 protein (P0DJI8)			
Alpha-1-antichymotrypsin (P01011)	Complement C4-A (P0C0L4)	Leucine-rich alpha-2-glycoprotein (P02750)			
Biotinidase (P43251)	Clusterin (P10909)	Apolipoprotein E (P02649)			
Hemopexin (P02790)	Complement C5 (P01031)	Apolipoprotein B-100 (P04114)			
Afamin (P43652)	Complement C3 (P01024)	Inter-alpha-trypsin inhibitor heavy chain H4 (Q14624)			
Apolipoprotein A-II (P02652)	Kininogen-1 (P01042)	Fibronectin (P02751)			
	Serum amyloid P-component (P02743)	Apolipoprotein C-III (P02656)			
	Conserved oligomeric Golgi complex subunit 4 (Q9H9E3)	Inter-alpha-trypsin inhibitor heavy chain H1 (P19827)			
	Serum paraoxonase/arylesterase 1 (P27169)	Inter-alpha-trypsin inhibitor heavy chain H2 (P19823)			
		Haptoglobin (P00738)			
		Heparin cofactor 2 (P05546)			
		Apolipoprotein A-I (P02647)			

Table S8B. Differentially abundant proteins in the longitudinal cohort of vivax malaria patients (compared to HC) identified by iTRAQ-based quantitative proteomics analysis using Q-Exactive mass spectrometer

S. No.	No. of unique peptides	Fold-change FEB/HC	Fold-change DEF/HC	Fold-change CON/HC	Accession number	Protein name
1	133	0.984	0.549	0.622	C0JYY2	Apolipoprotein B
2	117	1.019	0.614	0.656	P01024	Complement C3
3	76	1.046	0.653	0.66	P01023	Alpha-2-macroglobulin
4	47	1.095	0.501	0.715	B7ZLE5	FN1 protein
5	45	0.818	1.096	0.706	P02768	Serum albumin
6	35	1.762	0.868	0.939	P01009	Alpha-1-antitrypsin
7	35	1.291	0.715	0.8	B2RMS9	Inter-alpha (Globulin) inhibitor H4
8	33	1.42	0.721	0.835	B4E1Z4	Complement factor B
9	31	1.172	0.655	0.774	P01031	Complement C5 OS
10	30	0.976	0.549	0.637	Q5T985	Inter-alpha-trypsin inhibitor heavy chain H2
11	25	1.7	1.552	0.991	P02790	Hemopexin
12	23	1.002	0.597	0.669	P00747	Plasminogen
13	23	0.934	0.548	0.648	A8K5T0	cDNA FLJ75416, highly similar to Homo sapiens complement factor H (CFH), mRNA
14	23	0.554	0.421	0.657	P06727	Apolipoprotein A-IV
15	22	0.966	0.499	0.586	P06396	Gelsolin
16	21	0.953	0.567	0.628	P00734	Prothrombin
17	20	0.952	0.522	0.627	P43652	Afamin
18	19	2.199	0.933	1.18	P01011	Alpha-1-antichymotrypsin
19	17	1.269	0.677	0.716	P01008	Antithrombin-III
20	17	0.401	0.422	0.424	P02647	Apolipoprotein A-I
21	16	1.507	0.774	0.943	Q06033	Inter-alpha-trypsin inhibitor heavy chain H3
22	16	1.053	0.609	0.672	P25311	Zinc-alpha-2-glycoprotein
23	15	1.313	0.708	0.82	B4E1D8	cDNA FLJ51597, highly similar to C4b-binding protein alpha chain
24	13	1.717	1.657	1.506	P02649	Apolipoprotein E
25	13	1.637	0.82	1.013	P02748	Complement component C9
26	13	1.394	0.723	0.837	B4E1H2	Plasma protease C1 inhibitor
27	13	1.026	0.719	0.824	O43866	CD5 antigen-like
28	13	1.059	0.589	0.602	B0AZL7	cDNA, FLJ79457, highly similar to Insulin-like growth factor-binding proteincomplex acid labile chain
29	13	0.945	0.558	0.605	B4E1B2	cDNA FLJ53691, highly similar to Serotransferrin
30	13	0.848	0.475	0.573	B2R8I2	cDNA, FLJ93914, highly similar to Homo sapiens histidine-rich glycoprotein (HRG), mRNA

31	12	1.229	0.674	0.784	G3XAM2	Complement factor I light chain
32	12	0.405	0.623	0.656	P00738	Haptoglobin
33	12	1.133	0.524	0.705	P36955	Pigment epithelium-derived factor
34	11	1.876	1.618	0.797	P04004	Vitronectin
35	11	1.235	0.678	0.807	B4E1B3	cDNA FLJ53950, highly similar to Angiotensinogen
36	11	0.843	0.553	0.596	P05546	Heparin cofactor 2
37	11	0.94	0.537	0.6	P27169	Serum paraoxonase/arylesterase 1
38	11	1.011	0.536	0.692	P03952	Plasma kallikrein
39	11	1.074	0.5	0.686	P10909	Clusterin
40	11	0.916	0.482	0.577	B2R815	cDNA, FLJ93695, highly similar to Homo sapiens serpin peptidase inhibitor, clade A (alpha-1 antiproteinase, antitrypsin)
41	11	0.805	0.421	0.56	D9IWP9	Beta-2-glycoprotein I
42	10	1.155	0.725	0.791	P08185	Corticosteroid-binding globulin
43	10	1.405	0.676	0.817	P08697	Alpha-2-antiplasmin
44	10	0.891	0.528	0.583	P02765	Alpha-2-HS-glycoprotein
45	10	0.839	0.464	0.582	A8K050	cDNA FLJ75376, highly similar to Homo sapiens peptidoglycan recognition protein L
46	9	1.473	0.702	0.841	F8WCZ6	Complement C1s subcomponent
47	9	1.286	0.646	0.795	P22792	Carboxypeptidase N subunit 2
48	9	1.028	0.619	0.721	P04217	Alpha-1B-glycoprotein
49	9	1.204	0.616	0.766	P13671	Complement component C6
50	9	1.099	0.604	0.701	P02760	Protein AMBP
51	9	0.977	0.502	0.612	P51884	Lumican
52	8	1.553	0.795	0.999	P05543	Thyroxine-binding globulin
53	8	1.305	0.633	0.852	A8K5J8	cDNA FLJ75066, highly similar to Homo sapiens complement component 1
54	8	0.974	0.563	0.726	Q16519	Protein S (Fragment)
55	8	0.841	0.486	0.58	P02766	Transthyretin
56	7	2.233	1.068	1.184	P02763	Alpha-1-acid glycoprotein 1
57	7	1.229	0.711	0.826	Q6PIL8	IGK@ protein
58	7	1.323	0.682	0.704	B4DVE1	cDNA FLJ53478, highly similar to Galectin-3-binding protein
59	7	1.178	0.599	0.674	P07357	Complement component C8 alpha chain
60	7	0.811	0.452	0.52	P05090	Apolipoprotein D
61	6	1.715	0.822	0.971	P19652	Alpha-1-acid glycoprotein 2
62	6	0.568	0.783	0.598	P00739	Haptoglobin-related protein
63	6	1.105	0.757	0.879	P01591	Immunoglobulin J chain
64	6	1.194	0.703	0.702	A8K3E4	cDNA FLJ78367, highly similar to Homo sapiens fibrinogen, A alpha polypeptide (FGA)
65	6	0.85	0.566	0.777	Q6ZW64	cDNA FLJ41552 fis, clone COLON2004478, highly similar to Protein Tro alpha1 H

66	6	0.51	0.514	0.783	P02652	Apolipoprotein A-II
67	5	8.291	3.503	4.69	P02741	C-reactive protein
68	5	1.761	0.736	0.937	L8E853	von Willebrand factor
69	5	1.707	0.549	0.653	P02743	Serum amyloid P-component
70	5	0.823	0.445	0.639	Q5VY30	Plasma retinol-binding protein(1-182)
71	4	1.66	1.362	0.884	P01860	Ig gamma-3 chain C region
72	4	2.223	1.077	1.191	Q8TCF0	LBP protein
73	4	1.615	0.999	1.151	P01880	Ig delta chain C region
74	4	1.531	0.977	0.898	A2MYD4	V2-7 protein
75	4	5.59	0.852	0.866	Q14474	A-gamma-hemoglobin gene from Greek HPFH mutant,
76	4	1.328	0.801	0.891	B4DDD5	cDNA FLJ58762, highly similar to Phospholipid transfer protein
77	4	1.275	0.751	0.912	A8K5A4	cDNA FLJ76826, highly similar to Homo sapiens ceruloplasmin
78	4	1.371	0.747	0.893	P15169	Carboxypeptidase N catalytic chain
79	4	1.208	0.709	0.709	B7Z7C9	cDNA FLJ50907, highly similar to Biotinidase
80	4	1.186	0.705	0.807	P00748	Coagulation factor XII
81	4	1.178	0.668	0.81	B4DNT5	cDNA FLJ60316, highly similar to Apolipoprotein-L1
82	4	1.332	0.64	0.679	P07360	Complement component C8 gamma chain
83	4	1.289	0.637	0.738	B7Z550	Complement component 8, beta polypeptide
84	4	0.972	0.587	0.674	P20851	C4b-binding protein beta chain
85	4	0.948	0.582	0.646	P05160	Coagulation factor XIII B chain
86	4	0.822	0.577	0.594	P07996	Thrombospondin-1
87	4	0.929	0.551	0.637	D3JV41	Thrombocidin-2 antimicrobial variant
88	4	1.046	0.507	0.584	Q7Z379	Putative uncharacterized protein DKFZp686K04218
89	3	1.976	1	1.131	C8C504	Beta-globin
90	3	1.537	0.941	1.086	P01625	Ig kappa chain V-IV region Len
91	3	1.211	0.803	0.982	P01871	Ig mu chain C region
92	3	4.061	0.725	0.99	E5RH81	Carbonic anhydrase 1
93	3	0.823	0.713	0.756	B2R950	cDNA, FLJ94213, highly similar to Homo sapiens pregnancy-zone protein (PZP)
94	3	1.242	0.712	0.833	P22352	Glutathione peroxidase 3
95	3	1.467	0.702	0.876	B2R888	Monocyte differentiation antigen CD14
96	3	1.225	0.702	0.8	Q6PIK1	IGL@ protein OS
97	3	1.04	0.698	0.843	Q14520	Hyaluronan-binding protein 2
98	3	1.217	0.676	0.802	D3DPF9	Titin, isoform CRA_b
99	3	1.219	0.665	0.798	E7EQ48	Proteoglycan 4
100	3	1.089	0.662	0.711	F8WDX4	Complement factor H
101	3	0.927	0.651	0.647	P0C0L4	Complement C4-A

102	3	1.12	0.642	0.767	P00742	Coagulation factor X
103	3	1.125	0.629	0.787	B1AKG0	Complement factor H-related protein 1
104	3	0.843	0.623	0.743	P02655	Apolipoprotein C-II
105	3	1.126	0.581	0.695	B4DQI1	Complement C2b fragment
106	3	0.652	0.472	0.597	P02656	Apolipoprotein C-III
107	3	0.605	0.427	0.501	P02776	Platelet factor 4
108	3	0.775	0.378	0.502	P80108	Phosphatidylinositol-glycan-specific phospholipase D
109	3	0.49	0.355	0.43	Q5SRP5	Apolipoprotein M
110	2	2.06	1.928	1.494	A2MYC8	V5-2 protein (Fragment)
111	2	1.205	1.769	1.14	Q6J1Z9	Hemoglobin alpha 1
112	2	2.65	1.668	0.592	B2R9V7	Superoxide dismutase [Cu-Zn]
113	2	1.67	1.059	1.12	A2MYD0	V1-17 protein (Fragment)
114	2	1.29	0.993	1.053	P01703	Ig lambda chain V-I region NEWM
115	2	2.404	0.993	1.212	Q6FHW3	DF protein
116	2	1.171	0.927	0.724	P0C0L5	Complement C4-B
117	2	1.408	0.849	1.095	A2NB45	Cold agglutinin FS-1 L-chain (Fragment)
118	2	1.333	0.818	0.857	Q9UL88	Myosin-reactive immunoglobulin heavy chain variable region (Fragment)
119	2	2.771	0.807	1.107	Q4TZM4	Hemoglobin beta chain
120	2	1.578	0.804	0.904	Q9UK55	Protein Z-dependent protease inhibitor
121	2	1.277	0.796	0.784	P01766	Ig heavy chain V-III region BRO
122	2	2.622	0.79	1.082	P02042	Hemoglobin subunit delta
123	2	1.336	0.784	0.77	H0Y8L3	Transforming growth factor-beta-induced protein ig-h3
124	2	1.279	0.774	0.898	Q96JD0	Amyloid lambda 6 light chain variable region SAR
125	2	1.617	0.735	1.319	D6RF35	Vitamin D-binding protein
126	2	1.156	0.731	0.691	D1CS68	TLR7
127	2	1.389	0.719	0.803	A0N5G1	Rheumatoid factor C6 light chain
128	2	1.61	0.717	0.905	Q16610	Extracellular matrix protein 1
129	2	1.133	0.715	0.773	Q9ULB6	Imunoglobulin heavy chain
130	2	1.205	0.705	0.857	S6BGD6	IgG L chain
131	2	1.376	0.702	0.89	I3L1U9	Actin, cytoplasmic 2, N-terminally processed
132	2	1.396	0.698	0.788	Q6UY50	NL3
133	2	1.166	0.694	0.804	A0N5G5	Rheumatoid factor D5 light chain
134	2	1.911	0.669	1.743	Q5NV83	V3-3 protein
135	2	1.073	0.662	0.553	O00300	Tumor necrosis factor receptor superfamily member 11B
136	2	1.283	0.657	0.812	P26927	Hepatocyte growth factor-like protein
137	2	0.858	0.6	0.613	I3L145	Sex hormone-binding globulin
138	2	0.959	0.586	0.683	B4DI57	cDNA FLJ54111, highly similar to Serotransferrin

139	2	1.07	0.571	0.567	B4DZ36	cDNA FLJ58441, highly similar to Attractin
140	2	1.281	0.567	1.17	A0M8Q6	Ig lambda-7 chain C region
141	2	1.172	0.563	0.756	Q8NBH6	cDNA PSEC0266 fis, clone NT2RP3003649, highly similar to Homo sapiens fibulin-1D
142	2	0.972	0.549	0.601	Q96IY4	Carboxypeptidase B2
143	2	1.011	0.546	0.609	A6XND1	Insulin-like growth factor binding protein 3 isoform b
144	2	0.889	0.542	0.664	B4E1C4	cDNA FLJ51179, highly similar to Vitamin K-dependent protein C (EC 3.4.21.69)
145	2	1.053	0.538	0.62	B2R582	cDNA, FLJ92374, highly similar to Homo sapiens C-type lectin domain family 3, member B (CLEC3B)
146	2	1.325	0.51	0.788	B4E1C2	Kininogen 1, isoform CRA_b
147	2	0.721	0.465	0.552	P02774	Vitamin D-binding protein
148	2	0.897	0.463	0.549	D6REX5	Selenoprotein P
149	2	0.965	0.414	0.628	B4E180	cDNA FLJ61201, highly similar to Beta-Ala-His dipeptidase (EC 3.4.13.20)
150	2	0.706	0.407	0.551	K7ERI9	Truncated apolipoprotein C-I
151	2	1.846	1.679	0.699	P0DJI9	Serum amyloid A-2 protein

Table S9A. Details of the pathways and networks associated with the differentially abundant serum proteins identified in low and moderately-high parasitemic vivax malaria patients

Biocarta									
Category	Term	Count	%	PValue	Genes	List Total	Fold Enrichment	Benjamini	FDR
BIOCARTA	h_intrinsicPathway:Intrinsic Prothrombin Activation Pathway	4	4.60	7.01E-04	P02671, P03952, P00748, P00734	17	19.89	0.021	0.596
**KEGG PATHWAY**									
Category	Term	Count	%	PValue	Genes	List Total	Fold Enrichment	Benjamini	FDR
KEGG_PATHWAY	hsa04610:Complement and coagulation cascades	12	13.79	1.92E-13	P02679, P02671, P07358, P05154, P04003, P02675, P02748, P01009, P03952, P00748, P01023, P00734	34	26.01	0.00	0.00
**PANTHER PATHWAY**									
Category	Term	Count	%	PValue	Genes	List Total	Fold Enrichment	Benjamini	FDR
PANTHER_PATHWAY	P00011:Blood coagulation	8	9.20	2.80E-08	P02679, P02671, P02675, P01009, P03952, P00748, P01023, P00734	20	20.78	0.00	0.00
PANTHER_PATHWAY	P00050:Plasminogen activating cascade	3	3.45	0.009	P02679, P02671, P02675	20	19.48	0.08	6.41

Table S9B. Details of the pathways and networks associated with the differentially abundant serum proteins identified in moderately high parasitemic vivax malaria patients

Biocarta									
Category	Term	Count	%	P-value	Genes	List Total	Fold Enrichment	Benjamini	FDR
BIOCARTA	h_compPathway:Complement Pathway	7	7.95	2.55E-08	P01031, P07357, P00751, P02748, P09871, P00736, P06681	20	29.59	6.36E-07	2.07E-05
BIOCARTA	h_classicPathway:Classical Complement Pathway	6	6.82	1.71E-07	P01031, P07357, P02748, P09871, P00736, P06681	20	35.93	2.14E-06	1.39E-04
BIOCARTA	h_alternativePathway:Alternative Complement Pathway	5	5.68	4.37E-06	P01031, P07357, P00751, P02748, P06681	20	35.93	3.64E-05	0.003549
BIOCARTA	h_lectinPathway:Lectin Induced Complement Pathway	4	4.55	4.00E-04	P01031, P07357, P02748, P06681	20	23.95	0.0025	0.32
**PANTHER Pathway**									
Category	Term	Count	%	P-value	Genes	List Total	Fold Enrichment	Benjamini	FDR
PANTHER_PATHWAY	P00011:Blood coagulation	8	9.09	3.79E-09	P02679, P36955, P02675, P01009, P04275, P03952, P00748, P01023	16	25.972727	4.93E-08	2.51E-06
PANTHER_PATHWAY	P00050:Plasminogen activating cascade	3	3.41	0.005595	P02679, P36955, P02675	16	24.349432	0.035811	3.646378
**KEGG Pathway**									
Category	Term	Count	%	P-value	Genes	List Total	Fold Enrichment	Benjamini	FDR
KEGG_PATHWAY	hsa04610:Complement and coagulation cascades	16	18.18	2.92E-20	P02679, P01031, P07357, P04003, P02675, P02748, P09871, P00751, P05154, P01009, P04275, P03952, P00748, P01023, P06681, P00736	35	33.689441	9.04E-19	2.50E-17
KEGG_PATHWAY	hsa05322:Systemic lupus erythematosus	7	7.95	4.05E-05	P01031, P07357, P02748, P09871, P20671, P00736, P06681	35	10.27	0.00	0.03
KEGG_PATHWAY	hsa05020:Prion diseases	3	3.41	0.022496	P01031, P07357, P02748	35	12.45	0.21	17.76

Table S10. Details of the pathways and networks associated with the differentially abundant serum proteins identified in a longitudinal cohort of vivax malaria patients

Pathways	List Total	Count	Expected	+/-	*p*-value
Blood coagulation	45	8	0.17	+	2.21E-09
Plasminogen activating cascade	17	3	0.07	+	7.62E-03
Transport	2564	18	9.88	+	1.00E+00
Single-multicellular organism process	1798	13	6.93	+	1.00E+00
Cell cycle	1399	3	5.39	-	1.00E+00
Nitrogen compound metabolic process	308	1	1.19	-	1.00E+00
Cellular defense response	387	3	1.49	+	1.00E+00
Nervous system development	1008	2	3.88	-	1.00E+00
Ectoderm development	909	3	3.5	-	1.00E+00
Cellular amino acid biosynthetic process	125	1	0.48	+	1.00E+00
Response to stress	439	4	1.69	+	1.00E+00
Pattern specification process	244	1	0.94	+	1.00E+00
Cellular component morphogenesis	646	5	2.49	+	1.00E+00
Cytokinesis	187	1	0.72	+	1.00E+00
Carbohydrate transport	94	0	0.36	-	1.00E+00
Regulation of biological process	2175	7	8.38	-	1.00E+00
Vitamin catabolic process	1	0	0	-	1.00E+00
Vitamin biosynthetic process	39	0	0.15	-	1.00E+00
Biological regulation	3252	18	12.53	+	1.00E+00
Response to pheromone	16	1	0.06	+	1.00E+00
Sensory perception of pain	16	0	0.06	-	1.00E+00
Peroxisomal transport	33	0	0.13	-	1.00E+00
Segment specification	172	0	0.66	-	1.00E+00
Hemopoiesis	155	1	0.6	+	1.00E+00
Defense response to bacterium	30	0	0.12	-	1.00E+00
Synaptic vesicle exocytosis	100	0	0.39	-	1.00E+00
Cytoskeleton organization	16	0	0.06	-	1.00E+00
Protein complex biogenesis	79	0	0.3	-	1.00E+00
Sensory perception of chemical stimulus	13	0	0.05	-	1.00E+00
Rrna metabolic process	127	0	0.49	-	1.00E+00
Sensory perception of sound	78	1	0.3	+	1.00E+00
Purine nucleobase metabolic process	103	0	0.4	-	1.00E+00
Cellular amino acid catabolic process	69	0	0.27	-	1.00E+00
Primary metabolic process	7177	39	27.65	+	1.00E+00
Muscle contraction	280	2	1.08	+	1.00E+00
Visual perception	303	4	1.17	+	1.00E+00
Sensory perception	442	5	1.7	+	1.00E+00

Regulation of vasoconstriction	51	0	0.2	-	1.00E+00
Neuronal action potential propagation	21	0	0.08	-	1.00E+00
Receptor-mediated endocytosis	193	0	0.74	-	1.00E+00
Endocytosis	397	0	1.53	-	1.00E+00
Anatomical structure morphogenesis	691	5	2.66	+	1.00E+00
Regulation of phosphate metabolic process	67	1	0.26	+	1.00E+00
Coenzyme metabolic process	101	0	0.39	-	1.00E+00
Mammary gland development	27	0	0.1	-	1.00E+00
Angiogenesis	246	1	0.95	+	1.00E+00
Death	699	4	2.69	+	1.00E+00
Mitochondrion organization	21	0	0.08	-	1.00E+00
Chromatin remodeling	3	0	0.01	-	1.00E+00
Protein lipidation	23	0	0.09	-	1.00E+00
Cell-matrix adhesion	115	3	0.44	+	1.00E+00
Biosynthetic process	143	0	0.55	-	1.00E+00
Catabolic process	157	1	0.6	+	1.00E+00
Cellular component movement	493	2	1.9	+	1.00E+00
Regulation of nucleobase-containing compound metabolic process	1648	1	6.35	-	1.00E+00
Tricarboxylic acid cycle	23	0	0.09	-	1.00E+00
Pentose-phosphate shunt	8	0	0.03	-	1.00E+00
Glycolysis	25	0	0.1	-	1.00E+00
Protein transport	1339	1	5.16	-	1.00E+00
RNA splicing	272	1	1.05	-	1.00E+00
Gluconeogenesis	32	0	0.12	-	1.00E+00
Exocytosis	275	0	1.06	-	1.00E+00
Intracellular protein transport	1322	1	5.09	-	1.00E+00
Generation of precursor metabolites and energy	280	0	1.08	-	1.00E+00
Cellular glucose homeostasis	33	0	0.13	-	1.00E+00
Regulation of cellular amino acid metabolic process	10	0	0.04	-	1.00E+00
Cellular amino acid metabolic process	368	2	1.42	+	1.00E+00
Cell communication	3221	19	12.41	+	1.00E+00
Chromatin organization	257	0	0.99	-	1.00E+00
Protein glycosylation	177	0	0.68	-	1.00E+00
Cell death	697	4	2.69	+	1.00E+00
Induction of apoptosis	228	2	0.88	+	1.00E+00
Ferredoxin metabolic process	11	0	0.04	-	1.00E+00
Apoptotic process	697	4	2.69	+	1.00E+00
System process	1481	12	5.71	+	1.00E+00
DNA repair	191	0	0.74	-	1.00E+00
Response to toxic substance	61	0	0.24	-	1.00E+00
Natural killer cell activation	103	0	0.4	-	1.00E+00

Vitamin transport	81	2	0.31	+	1.00E+00
Cellular calcium ion homeostasis	49	0	0.19	-	1.00E+00
Embryo development	187	0	0.72	-	1.00E+00
Skeletal system development	334	4	1.29	+	1.00E+00
Cell growth	12	0	0.05	-	1.00E+00
Protein methylation	12	0	0.05	-	1.00E+00
Cellular component organization	1065	5	4.1	+	1.00E+00
Oxidative phosphorylation	57	0	0.22	-	1.00E+00
Mrna 3'-end processing	94	0	0.36	-	1.00E+00
Protein acetylation	36	0	0.14	-	1.00E+00
DNA recombination	87	0	0.34	-	1.00E+00
Protein ADP-ribosylation	10	0	0.04	-	1.00E+00
Phagocytosis	30	0	0.12	-	1.00E+00
Bile acid metabolic process	1	0	0	-	1.00E+00
Macrophage activation	289	3	1.11	+	1.00E+00
Pinocytosis	9	0	0.03	-	1.00E+00
Cellular process	5952	33	22.93	+	1.00E+00
Cholesterol metabolic process	108	0	0.42	-	1.00E+00
Localization	2636	18	10.16	+	1.00E+00
Steroid metabolic process	219	0	0.84	-	1.00E+00
Response to abiotic stimulus	21	0	0.08	-	1.00E+00
Mrna splicing, via spliceosome	368	1	1.42	-	1.00E+00
Amino acid transport	83	0	0.32	-	1.00E+00
Sex determination	13	0	0.05	-	1.00E+00
Mesoderm development	959	7	3.69	+	1.00E+00
Endoderm development	23	0	0.09	-	1.00E+00
DNA catabolic process	8	0	0.03	-	1.00E+00
Protein phosphorylation	550	1	2.12	-	1.00E+00
Monosaccharide metabolic process	135	0	0.52	-	1.00E+00
Female gamete generation	144	0	0.55	-	1.00E+00
Cellular protein modification process	1204	3	4.64	-	1.00E+00
Regulation of carbohydrate metabolic process	21	0	0.08	-	1.00E+00
Protein complex assembly	79	0	0.3	-	1.00E+00
Vesicle-mediated transport	928	1	3.58	-	1.00E+00
Cyclic nucleotide metabolic process	99	0	0.38	-	1.00E+00
Nitrogen utilization	1	0	0	-	1.00E+00
Nuclear transport	121	0	0.47	-	1.00E+00
Locomotion	18	0	0.07	-	1.00E+00
System development	1645	7	6.34	+	1.00E+00
DNA replication	221	1	0.85	+	1.00E+00
Extracellular transport	125	0	0.48	-	1.00E+00

Metabolic process	8613	40	33.18	+	1.00E+00
Meiosis	100	0	0.39	-	1.00E+00
Protein folding	194	0	0.75	-	1.00E+00
Spermatogenesis	223	1	0.86	+	1.00E+00
Disaccharide metabolic process	6	0	0.02	-	1.00E+00
Cellular component organization or biogenesis	1137	5	4.38	+	1.00E+00
DNA metabolic process	434	1	1.67	-	1.00E+00
Growth	17	0	0.07	-	1.00E+00
Muscle organ development	390	2	1.5	+	1.00E+00
Antigen processing and presentation of peptide or polysaccharide antigen via MHC class II	51	0	0.2	-	1.00E+00
Response to biotic stimulus	1	0	0	-	1.00E+00
RNA splicing, via transesterification reactions	272	1	1.05	-	1.00E+00
Fertilization	82	1	0.32	+	1.00E+00
Phospholipid metabolic process	190	0	0.73	-	1.00E+00
Gamete generation	534	4	2.06	+	1.00E+00
Glycogen metabolic process	90	0	0.35	-	1.00E+00
Polysaccharide metabolic process	228	0	0.88	-	1.00E+00
Neuromuscular synaptic transmission	15	0	0.06	-	1.00E+00
Carbohydrate metabolic process	650	1	2.5	-	1.00E+00
Neuron-neuron synaptic transmission	90	0	0.35	-	1.00E+00
B cell mediated immunity	234	2	0.9	+	1.00E+00
Neurological system process	1212	6	4.67	+	1.00E+00
Antigen processing and presentation	81	1	0.31	+	1.00E+00
Heart development	240	0	0.92	-	1.00E+00
Mitochondrial transport	21	0	0.08	-	1.00E+00
Dorsal/ventral axis specification	39	0	0.15	-	1.00E+00
Digestive tract mesoderm development	54	0	0.21	-	1.00E+00
Nucleobase-containing compound transport	110	0	0.42	-	1.00E+00
Organelle organization	318	0	1.23	-	1.00E+00
Acyl-coa metabolic process	30	0	0.12	-	1.00E+00
Sensory perception of taste	11	0	0.04	-	1.00E+00
Polyphosphate catabolic process	3	0	0.01	-	1.00E+00
Unsaturated fatty acid biosynthetic process	9	0	0.03	-	1.00E+00
Neurotransmitter secretion	190	0	0.73	-	1.00E+00
Fatty acid beta-oxidation	35	0	0.13	-	1.00E+00
Reproduction	616	4	2.37	+	1.00E+00
Synaptic transmission	415	0	1.6	-	1.00E+00
Phosphate-containing compound metabolic process	520	1	2	-	1.00E+00
Cell-cell signaling	835	2	3.22	-	1.00E+00
Fatty acid biosynthetic process	41	1	0.16	+	1.00E+00
Fatty acid metabolic process	188	1	0.72	+	1.00E+00

Sulfur compound metabolic process	84	0	0.32	-	1.00E+00
Mitosis	528	1	2.03	-	1.00E+00
Negative regulation of apoptotic process	186	2	0.72	+	1.00E+00
Trna metabolic process	65	0	0.25	-	1.00E+00
Anterior/posterior axis specification	44	1	0.17	+	1.00E+00
Mrna processing	438	1	1.69	-	1.00E+00
Homeostatic process	136	0	0.52	-	1.00E+00
**Molecular function**	**List Total**	**Count**	**Expected**	**+/-**	***p*- value**
Peptidase activity	747	28	2.88	+	3.47E-18
Peptidase inhibitor activity	193	14	0.74	+	5.09E-12
Lipid transporter activity	92	10	0.35	+	5.87E-10
Hydrolase activity	2332	33	8.98	+	1.36E-09
Serine-type peptidase activity	314	14	1.21	+	3.22E-09
Serine-type endopeptidase inhibitor activity	83	9	0.32	+	7.55E-09
Enzyme inhibitor activity	340	14	1.31	+	9.05E-09
Metallopeptidase activity	198	9	0.76	+	1.32E-05
Enzyme regulator activity	1091	16	4.2	+	5.99E-04
Nucleic acid binding	3315	2	12.77	-	1.95E-02
Protein binding	2855	24	11	+	2.22E-02
DNA binding	2032	0	7.83	-	4.26E-02
Receptor binding	1017	12	3.92	+	8.25E-02
Catalytic activity	5529	35	21.3	+	1.25E-01
Cytokine activity	188	5	0.72	+	1.32E-01
Nucleic acid binding transcription factor activity	1762	0	6.79	-	1.33E-01
Sequence-specific DNA binding transcription factor activity	1754	0	6.76	-	1.38E-01
Transferase activity	1614	0	6.22	-	2.47E-01
Transporter activity	1145	11	4.41	+	7.09E-01
Cation transmembrane transporter activity	368	0	1.42	-	1.00E+00
G-protein coupled receptor activity	284	0	1.09	-	1.00E+00
Glucosidase activity	12	0	0.05	-	1.00E+00
Transaminase activity	26	0	0.1	-	1.00E+00
Galactosidase activity	8	0	0.03	-	1.00E+00
Lipid binding	26	0	0.1	-	1.00E+00
DNA ligase activity	2	0	0.01	-	1.00E+00
Cytokine receptor activity	66	1	0.25	+	1.00E+00
Extracellular matrix structural constituent	91	1	0.35	+	1.00E+00
Structural constituent of cytoskeleton	851	4	3.28	+	1.00E+00
Exoribonuclease activity	32	0	0.12	-	1.00E+00
Atpase activity, coupled to transmembrane movement of substances	34	0	0.13	-	1.00E+00

Neurotrophin receptor binding	10	0	0.04	-	1.00E+00
Tumor necrosis factor receptor binding	10	0	0.04	-	1.00E+00
Growth factor activity	171	0	0.66	-	1.00E+00
Phosphoric diester hydrolase activity	40	0	0.15	-	1.00E+00
Transferase activity, transferring acyl groups	179	0	0.69	-	1.00E+00
Exodeoxyribonuclease activity	6	0	0.02	-	1.00E+00
Phosphoprotein phosphatase activity	179	2	0.69	+	1.00E+00
Endoribonuclease activity	50	0	0.19	-	1.00E+00
Endodeoxyribonuclease activity	19	0	0.07	-	1.00E+00
Ligand-gated ion channel activity	97	0	0.37	-	1.00E+00
Ligand-activated sequence-specific DNA binding RNA polymerase II transcription factor activity	48	0	0.18	-	1.00E+00
Calcium-dependent phospholipid binding	124	2	0.48	+	1.00E+00
Non-membrane spanning protein tyrosine kinase activity	96	0	0.37	-	1.00E+00
Transmembrane receptor protein tyrosine kinase activity	17	0	0.07	-	1.00E+00
Hydrogen ion transmembrane transporter activity	45	0	0.17	-	1.00E+00
Nuclease activity	196	0	0.76	-	1.00E+00
Receptor activity	1576	13	6.07	+	1.00E+00
Glutamate receptor activity	23	0	0.09	-	1.00E+00
Transmembrane receptor protein serine/threonine kinase activity	22	0	0.08	-	1.00E+00
Protein kinase activity	414	0	1.59	-	1.00E+00
Lipase activity	87	1	0.34	+	1.00E+00
Oxidoreductase activity	634	0	2.44	-	1.00E+00
Acetylcholine receptor activity	46	0	0.18	-	1.00E+00
Cysteine-type endopeptidase inhibitor activity	21	1	0.08	+	1.00E+00
Interleukin-3 receptor binding	1	0	0	-	1.00E+00
GABA receptor activity	46	0	0.18	-	1.00E+00
Proton-transporting ATP synthase activity, rotational mechanism	20	0	0.08	-	1.00E+00
DNA polymerase processivity factor activity	1	0	0	-	1.00E+00
Ligase activity	500	1	1.93	-	1.00E+00
Transmembrane transporter activity	1076	5	4.15	+	1.00E+00
Binding	5933	27	22.86	+	1.00E+00
Deaminase activity	46	0	0.18	-	1.00E+00
SNAP receptor activity	24	0	0.09	-	1.00E+00
Centromeric DNA binding	17	0	0.07	-	1.00E+00
Enzyme activator activity	146	1	0.56	+	1.00E+00
Cytokine receptor binding	64	0	0.25	-	1.00E+00
Transmembrane receptor protein kinase activity	48	0	0.18	-	1.00E+00
Antigen binding	4	0	0.02	-	1.00E+00
Guanyl-nucleotide exchange factor activity	166	0	0.64	-	1.00E+00
Small gtpase regulator activity	400	1	1.54	-	1.00E+00

Calmodulin binding	262	3	1.01	+	1.00E+00
Intramolecular transferase activity	8	0	0.03	-	1.00E+00
Cysteine-type peptidase activity	149	2	0.57	+	1.00E+00
Ubiquitin-protein ligase activity	279	1	1.07	-	1.00E+00
Phosphorylase activity	12	0	0.05	-	1.00E+00
Kinase activity	814	0	3.14	-	1.00E+00
Pyrophosphatase activity	271	2	1.04	+	1.00E+00
Metalloendopeptidase inhibitor activity	4	0	0.02	-	1.00E+00
Actin binding	194	3	0.75	+	1.00E+00
Microtubule motor activity	86	0	0.33	-	1.00E+00
Motor activity	145	1	0.56	+	1.00E+00
Calcium ion binding	445	4	1.71	+	1.00E+00
Racemase and epimerase activity	58	0	0.22	-	1.00E+00
Isomerase activity	169	1	0.65	+	1.00E+00
Intermediate filament binding	10	0	0.04	-	1.00E+00
Deacetylase activity	27	1	0.1	+	1.00E+00
Phosphatase inhibitor activity	36	0	0.14	-	1.00E+00
Phosphatase activator activity	1	0	0	-	1.00E+00
Kinase inhibitor activity	84	0	0.32	-	1.00E+00
RNA-directed DNA polymerase activity	1	0	0	-	1.00E+00
Cation channel activity	147	0	0.57	-	1.00E+00
Kinase activator activity	94	0	0.36	-	1.00E+00
Aspartic-type endopeptidase activity	22	0	0.08	-	1.00E+00
Phosphatase regulator activity	71	0	0.27	-	1.00E+00
Kinase regulator activity	261	0	1.01	-	1.00E+00
Microtubule binding	64	0	0.25	-	1.00E+00
Nucleotide phosphatase activity	34	0	0.13	-	1.00E+00
Carbohydrate phosphatase activity	13	0	0.05	-	1.00E+00
Amino acid kinase activity	5	0	0.02	-	1.00E+00
Nucleotide kinase activity	58	0	0.22	-	1.00E+00
Phospholipase activity	70	0	0.27	-	1.00E+00
RNA methyltransferase activity	19	0	0.07	-	1.00E+00
Carbohydrate kinase activity	34	0	0.13	-	1.00E+00
Anion channel activity	35	0	0.13	-	1.00E+00
Protein disulfide isomerase activity	16	0	0.06	-	1.00E+00
Helicase activity	146	0	0.56	-	1.00E+00
Guanylate cyclase activity	26	0	0.1	-	1.00E+00
Hydro-lyase activity	56	0	0.22	-	1.00E+00
Carboxy-lyase activity	26	1	0.1	+	1.00E+00
Hydrolase activity, hydrolyzing N-glycosyl compounds	41	0	0.16	-	1.00E+00
Aminoacyl-trna ligase activity	37	0	0.14	-	1.00E+00

Chemokine activity	50	1	0.19	+	1.00E+00
Voltage-gated potassium channel activity	60	0	0.23	-	1.00E+00
Methyltransferase activity	122	0	0.47	-	1.00E+00
Voltage-gated sodium channel activity	37	0	0.14	-	1.00E+00
Phosphatase activity	300	2	1.16	+	1.00E+00
Amino acid transmembrane transporter activity	109	0	0.42	-	1.00E+00
Voltage-gated calcium channel activity	38	0	0.15	-	1.00E+00
Voltage-gated ion channel activity	133	0	0.51	-	1.00E+00
Gap junction channel activity	21	0	0.08	-	1.00E+00
Translation release factor activity	7	0	0.03	-	1.00E+00
Transcription factor binding transcription factor activity	192	0	0.74	-	1.00E+00
Translation elongation factor activity	44	0	0.17	-	1.00E+00
Protein binding transcription factor activity	192	0	0.74	-	1.00E+00
Translation initiation factor activity	102	0	0.39	-	1.00E+00
Lyase activity	209	1	0.81	+	1.00E+00
Sequence-specific DNA binding RNA polymerase II transcription factor activity	67	0	0.26	-	1.00E+00
Adenylate cyclase activity	67	0	0.26	-	1.00E+00
Hydrolase activity, acting on ester bonds	678	3	2.61	+	1.00E+00
Transketolase activity	5	0	0.02	-	1.00E+00
Translation regulator activity	132	0	0.51	-	1.00E+00
Peroxidase activity	27	0	0.1	-	1.00E+00
Structural molecule activity	1261	5	4.86	+	1.00E+00
DNA-directed RNA polymerase activity	43	0	0.17	-	1.00E+00
Structural constituent of ribosome	204	0	0.79	-	1.00E+00
DNA primase activity	1	0	0	-	1.00E+00
Tumor necrosis factor-activated receptor activity	22	1	0.08	+	1.00E+00
Single-stranded DNA binding	74	0	0.29	-	1.00E+00
Nucleotidyltransferase activity	78	0	0.3	-	1.00E+00
Double-stranded DNA binding	29	0	0.11	-	1.00E+00
Poly(A) RNA binding	86	0	0.33	-	1.00E+00
Gtpase activity	254	1	0.98	+	1.00E+00
Mrna binding	313	1	1.21	-	1.00E+00
Hydrolase activity, hydrolyzing O-glycosyl compounds	26	0	0.1	-	1.00E+00
DNA-directed DNA polymerase activity	29	0	0.11	-	1.00E+00
Transforming growth factor beta-activated receptor activity	14	0	0.05	-	1.00E+00
RNA helicase activity	89	0	0.34	-	1.00E+00
Neuropeptide hormone activity	25	0	0.1	-	1.00E+00
RNA binding	639	2	2.46	-	1.00E+00
DNA replication origin binding	44	0	0.17	-	1.00E+00
DNA-methyltransferase activity	18	0	0.07	-	1.00E+00
Damaged DNA binding	20	0	0.08	-	1.00E+00

Chromatin binding	194	0	0.75	-	1.00E+00
Carbohydrate transmembrane transporter activity	60	0	0.23	-	1.00E+00
Acetyltransferase activity	102	0	0.39	-	1.00E+00
Ion channel activity	354	0	1.36	-	1.00E+00
Translation factor activity, nucleic acid binding	137	0	0.53	-	1.00E+00
DNA topoisomerase activity	6	0	0.02	-	1.00E+00
Antioxidant activity	30	0	0.12	-	1.00E+00
DNA photolyase activity	2	0	0.01	-	1.00E+00
Hormone activity	108	0	0.42	-	1.00E+00
Unclassified	10605	30	40.86	-	1.00E+00
Nucleotide binding	37	0	0.14	-	1.00E+00
Cytoskeletal protein binding	271	3	1.04	+	1.00E+00
Transcription cofactor activity	192	0	0.74	-	1.00E+00
Structural constituent of myelin sheath	21	0	0.08	-	1.00E+00
DNA helicase activity	74	0	0.29	-	1.00E+00
Amylase activity	6	0	0.02	-	1.00E+00
Transferase activity, transferring glycosyl groups	229	0	0.88	-	1.00E+00
Cyclic nucleotide-gated ion channel activity	6	0	0.02	-	1.00E+00
**Cellular Component**	**List Total**	**Count**	**Expected**	**+/-**	***p*- value**
Extracellular region	533	10	2.05	+	2.06E-03
Extracellular matrix	376	6	1.45	+	1.73E-01
Unclassified	19442	67	74.9	-	4.12E-01
SNARE complex	22	0	0.08	-	1.00E+00
Cytosol	25	0	0.1	-	1.00E+00
Chromosome	17	0	0.07	-	1.00E+00
Vesicle coat	41	0	0.16	-	1.00E+00
Cytoskeleton	786	4	3.03	+	1.00E+00
Plasma membrane	250	2	0.96	+	1.00E+00
Apical part of cell	2	0	0.01	-	1.00E+00
Cell junction	66	1	0.25	+	1.00E+00
Cytoplasmic membrane-bounded vesicle	7	0	0.03	-	1.00E+00
Intermediate filament cytoskeleton	73	0	0.28	-	1.00E+00
Integral to membrane	30	0	0.12	-	1.00E+00
Membrane	398	2	1.53	+	1.00E+00
Endoplasmic reticulum	2	0	0.01	-	1.00E+00
Intracellular	1434	6	5.52	+	1.00E+00
Cell projection	19	0	0.07	-	1.00E+00
MHC protein complex	42	1	0.16	+	1.00E+00
Synapse	5	0	0.02	-	1.00E+00
Tubulin complex	23	0	0.09	-	1.00E+00

Microtubule	248	0	0.96	-	1.00E+00
Ribosome	11	0	0.04	-	1.00E+00
Ribonucleoprotein complex	134	0	0.52	-	1.00E+00
Protein complex	514	1	1.98	-	1.00E+00
Peroxisome	2	0	0.01	-	1.00E+00
Mitochondrial inner membrane	64	0	0.25	-	1.00E+00
Vacuole	4	0	0.02	-	1.00E+00
Microvillus	1	0	0	-	1.00E+00
Protein-DNA complex	3	0	0.01	-	1.00E+00
Proton-transporting ATP synthase complex	20	0	0.08	-	1.00E+00
Dendrite	3	0	0.01	-	1.00E+00
Macromolecular complex	648	1	2.5	-	1.00E+00
Nuclear chromosome	12	0	0.05	-	1.00E+00
Neuron projection	12	0	0.05	-	1.00E+00
Cell part	1592	6	6.13	-	1.00E+00
Heterotrimeric G-protein complex	27	0	0.1	-	1.00E+00
Immunoglobulin complex	4	0	0.02	-	1.00E+00
Mitochondrion	77	0	0.3	-	1.00E+00
Cytoplasm	329	1	1.27	-	1.00E+00
Organelle	1051	4	4.05	-	1.00E+00
Lysosome	2	0	0.01	-	1.00E+00
Cilium	4	0	0.02	-	1.00E+00
Tight junction	3	0	0.01	-	1.00E+00
Nucleus	71	0	0.27	-	1.00E+00
Actin cytoskeleton	388	4	1.49	+	1.00E+00
**PANTHER Protein Class**	**List Total**	**Count**	**Expected**	**+/-**	***p*- value**
Complement component	81	13	0.31	+	2.26E-15
Serine protease inhibitor	119	13	0.46	+	2.99E-13
Protease inhibitor	193	14	0.74	+	5.80E-12
Defense/immunity protein	572	20	2.2	+	9.15E-12
Transfer/carrier protein	439	17	1.69	+	1.87E-10
Apolipoprotein	91	10	0.35	+	6.02E-10
Protease	597	14	2.3	+	1.25E-05
Metalloprotease	198	9	0.76	+	1.51E-05
Serine protease	278	10	1.07	+	2.39E-05
Cell adhesion molecule	593	11	2.28	+	3.23E-03
Enzyme modulator	1439	16	5.54	+	1.98E-02
Unclassified	9728	21	37.48	-	2.88E-02
Hydrolase	1654	17	6.37	+	3.02E-02
Signaling molecule	1048	13	4.04	+	3.32E-02

Cytokine	178	5	0.69	+	1.18E-01
Transcription factor	1787	0	6.88	-	1.37E-01
Extracellular matrix protein	466	7	1.8	+	3.95E-01
Nucleic acid binding	2524	2	9.72	-	4.20E-01
Transferase	1313	0	5.06	-	9.76E-01
Mitochondrial carrier protein	62	0	0.24	-	1.00E+00
Microtubule family cytoskeletal protein	250	0	0.96	-	1.00E+00
Microtubule binding motor protein	86	0	0.33	-	1.00E+00
Methyltransferase	122	0	0.47	-	1.00E+00
Metalloprotease inhibitor	4	0	0.02	-	1.00E+00
Membrane-bound signaling molecule	224	1	0.86	+	1.00E+00
Membrane trafficking regulatory protein	133	0	0.51	-	1.00E+00
Membrane traffic protein	386	0	1.49	-	1.00E+00
Major histocompatibility complex antigen	45	1	0.17	+	1.00E+00
Mrna splicing factor	271	1	1.04	-	1.00E+00
Mrna processing factor	316	1	1.22	-	1.00E+00
Mrna polyadenylation factor	89	0	0.34	-	1.00E+00
Lyase	183	1	0.71	+	1.00E+00
Lipase	95	1	0.37	+	1.00E+00
Ligase	501	1	1.93	-	1.00E+00
Ligand-gated ion channel	109	0	0.42	-	1.00E+00
Kinase modulator	292	0	1.12	-	1.00E+00
Kinase inhibitor	115	0	0.44	-	1.00E+00
Kinase activator	94	0	0.36	-	1.00E+00
Kinase	545	0	2.1	-	1.00E+00
Isomerase	168	1	0.65	+	1.00E+00
Ionotropic glutamate receptor	23	0	0.09	-	1.00E+00
Ion channel	336	0	1.29	-	1.00E+00
Intracellular calcium-sensing protein	262	3	1.01	+	1.00E+00
Exoribonuclease	32	0	0.12	-	1.00E+00
Intermediate filament binding protein	10	0	0.04	-	1.00E+00
Exodeoxyribonuclease	9	0	0.03	-	1.00E+00
Esterase	100	0	0.39	-	1.00E+00
Epimerase/racemase	58	0	0.22	-	1.00E+00
Endoribonuclease	49	0	0.19	-	1.00E+00
Endodeoxyribonuclease	20	0	0.08	-	1.00E+00
Dehydrogenase	257	0	0.99	-	1.00E+00
Dehydratase	40	0	0.15	-	1.00E+00
Intermediate filament	83	0	0.32	-	1.00E+00
Interleukin superfamily	36	0	0.14	-	1.00E+00
Interferon superfamily	18	0	0.07	-	1.00E+00

Immunoglobulin superfamily cell adhesion molecule	98	1	0.38	+	1.00E+00
Immunoglobulin receptor superfamily	180	2	0.69	+	1.00E+00
Immunoglobulin	4	0	0.02	-	1.00E+00
Hydroxylase	17	0	0.07	-	1.00E+00
Decarboxylase	26	1	0.1	+	1.00E+00
Hydratase	19	0	0.07	-	1.00E+00
Deaminase	46	0	0.18	-	1.00E+00
Deacetylase	27	1	0.1	+	1.00E+00
Damaged DNA-binding protein	21	0	0.08	-	1.00E+00
Cytoskeletal protein	865	4	3.33	+	1.00E+00
Cytokine receptor	278	3	1.07	+	1.00E+00
Cysteine protease inhibitor	21	1	0.08	+	1.00E+00
Cysteine protease	125	1	0.48	+	1.00E+00
Cyclic nucleotide-gated ion channel	18	0	0.07	-	1.00E+00
Homeobox transcription factor	227	0	0.87	-	1.00E+00
Histone	57	0	0.22	-	1.00E+00
Heterotrimeric G-protein	43	0	0.17	-	1.00E+00
Helix-turn-helix transcription factor	227	0	0.87	-	1.00E+00
Helicase	146	0	0.56	-	1.00E+00
Guanylate cyclase	18	0	0.07	-	1.00E+00
Guanyl-nucleotide exchange factor	166	0	0.64	-	1.00E+00
Growth factor	174	0	0.67	-	1.00E+00
Glycosyltransferase	223	0	0.86	-	1.00E+00
Cyclase	55	0	0.21	-	1.00E+00
Glycosidase	42	0	0.16	-	1.00E+00
Chromatin/chromatin-binding protein	190	0	0.73	-	1.00E+00
Chemokine	50	1	0.19	+	1.00E+00
Chaperonin	28	0	0.11	-	1.00E+00
Chaperone	221	0	0.85	-	1.00E+00
Centromere DNA-binding protein	17	0	0.07	-	1.00E+00
Cell junction protein	171	1	0.66	+	1.00E+00
Glucosidase	12	0	0.05	-	1.00E+00
Gap junction	21	0	0.08	-	1.00E+00
Galactosidase	6	0	0.02	-	1.00E+00
Extracellular matrix structural protein	93	1	0.36	+	1.00E+00
Extracellular matrix linker protein	28	0	0.11	-	1.00E+00
Extracellular matrix glycoprotein	133	1	0.51	+	1.00E+00
Cation transporter	221	0	0.85	-	1.00E+00
Carbohydrate transporter	60	0	0.23	-	1.00E+00
Carbohydrate phosphatase	13	0	0.05	-	1.00E+00
Carbohydrate kinase	35	0	0.13	-	1.00E+00

Calsequestrin	2	0	0.01	-	1.00E+00
Calmodulin	262	3	1.01	+	1.00E+00
Calcium-binding protein	384	4	1.48	+	1.00E+00
Calcium channel	38	0	0.15	-	1.00E+00
Cadherin	109	0	0.42	-	1.00E+00
Basic leucine zipper transcription factor	18	0	0.07	-	1.00E+00
Basic helix-loop-helix transcription factor	93	0	0.36	-	1.00E+00
Aspartic protease	24	0	0.09	-	1.00E+00
Antibacterial response protein	118	2	0.45	+	1.00E+00
Annexin	124	2	0.48	+	1.00E+00
Zinc finger transcription factor	682	0	2.63	-	1.00E+00
Voltage-gated sodium channel	37	0	0.14	-	1.00E+00
Anion channel	35	0	0.13	-	1.00E+00
Voltage-gated potassium channel	85	0	0.33	-	1.00E+00
Amylase	6	0	0.02	-	1.00E+00
Voltage-gated ion channel	136	0	0.52	-	1.00E+00
Aminoacyl-trna synthetase	37	0	0.14	-	1.00E+00
Voltage-gated calcium channel	38	0	0.15	-	1.00E+00
Amino acid transporter	109	0	0.42	-	1.00E+00
Amino acid kinase	5	0	0.02	-	1.00E+00
Aldolase	1	0	0	-	1.00E+00
Adenylate cyclase	51	0	0.2	-	1.00E+00
Acyltransferase	104	0	0.4	-	1.00E+00
Actin family cytoskeletal protein	424	4	1.63	+	1.00E+00
Actin binding motor protein	59	1	0.23	+	1.00E+00
Viral protein	9	0	0.03	-	1.00E+00
Viral coat protein	4	0	0.02	-	1.00E+00
Vesicle coat protein	42	0	0.16	-	1.00E+00
Ubiquitin-protein ligase	305	1	1.18	-	1.00E+00
Tyrosine protein kinase receptor	17	0	0.07	-	1.00E+00
Actin and actin related protein	31	0	0.12	-	1.00E+00
Type II cytokine receptor	13	0	0.05	-	1.00E+00
Acetyltransferase	102	0	0.39	-	1.00E+00
Type I cytokine receptor	59	0	0.23	-	1.00E+00
Acetylcholine receptor	46	0	0.18	-	1.00E+00
Replication origin binding protein	25	0	0.1	-	1.00E+00
Tumor necrosis factor receptor	23	1	0.09	+	1.00E+00
Reductase	211	0	0.81	-	1.00E+00
TGF-beta receptor	14	0	0.05	-	1.00E+00
Receptor	1597	13	6.15	+	1.00E+00
SNARE protein	41	0	0.16	-	1.00E+00

Pyrophosphatase	19	0	0.07	-	1.00E+00
RNA methyltransferase	19	0	0.07	-	1.00E+00
Protein phosphatase	180	2	0.69	+	1.00E+00
RNA helicase	89	0	0.34	-	1.00E+00
Protein kinase receptor	46	0	0.18	-	1.00E+00
RNA binding protein	983	2	3.79	-	1.00E+00
Protein kinase	404	0	1.56	-	1.00E+00
Tumor necrosis factor family member	10	0	0.04	-	1.00E+00
Tubulin	23	0	0.09	-	1.00E+00
Transporter	1069	5	4.12	+	1.00E+00
Transmembrane receptor regulatory/adaptor protein	80	0	0.31	-	1.00E+00
Translation release factor	7	0	0.03	-	1.00E+00
Translation initiation factor	78	0	0.3	-	1.00E+00
Translation factor	113	0	0.44	-	1.00E+00
KRAB box transcription factor	410	0	1.58	-	1.00E+00
Translation elongation factor	44	0	0.17	-	1.00E+00
Transketolase	5	0	0.02	-	1.00E+00
Hsp90 family chaperone	4	0	0.02	-	1.00E+00
Hsp70 family chaperone	17	0	0.07	-	1.00E+00
Primase	1	0	0	-	1.00E+00
Potassium channel	85	0	0.33	-	1.00E+00
Phosphorylase	12	0	0.05	-	1.00E+00
Phospholipase	65	0	0.25	-	1.00E+00
HMG box transcription factor	42	0	0.16	-	1.00E+00
GABA receptor	46	0	0.18	-	1.00E+00
Phosphodiesterase	36	0	0.14	-	1.00E+00
Phosphatase modulator	71	0	0.27	-	1.00E+00
G-protein modulator	487	2	1.88	+	1.00E+00
Phosphatase inhibitor	36	0	0.14	-	1.00E+00
G-protein coupled receptor	433	0	1.67	-	1.00E+00
G-protein	219	0	0.84	-	1.00E+00
Phosphatase activator	1	0	0	-	1.00E+00
Phosphatase	306	2	1.18	+	1.00E+00
Peroxidase	24	0	0.09	-	1.00E+00
Transcription cofactor	195	0	0.75	-	1.00E+00
Transaminase	26	0	0.1	-	1.00E+00
Tight junction	36	0	0.14	-	1.00E+00
DNA-directed RNA polymerase	48	0	0.18	-	1.00E+00
Surfactant	44	0	0.17	-	1.00E+00
Structural protein	226	0	0.87	-	1.00E+00
DNA-directed DNA polymerase	29	0	0.11	-	1.00E+00

Peptide hormone	109	0	0.42	-	1.00E+00
DNA topoisomerase	6	0	0.02	-	1.00E+00
Storage protein	23	0	0.09	-	1.00E+00
DNA strand-pairing protein	7	0	0.03	-	1.00E+00
Oxygenase	77	0	0.3	-	1.00E+00
DNA polymerase processivity factor	1	0	0	-	1.00E+00
Oxidoreductase	639	0	2.46	-	1.00E+00
DNA photolyase	2	0	0.01	-	1.00E+00
Oxidase	144	0	0.55	-	1.00E+00
DNA methyltransferase	18	0	0.07	-	1.00E+00
Nucleotidyltransferase	81	0	0.31	-	1.00E+00
DNA ligase	3	0	0.01	-	1.00E+00
DNA helicase	74	0	0.29	-	1.00E+00
Nucleotide phosphatase	34	0	0.13	-	1.00E+00
Nucleotide kinase	58	0	0.22	-	1.00E+00
DNA glycosylase	10	0	0.04	-	1.00E+00
Nuclease	200	0	0.77	-	1.00E+00
Sodium channel	37	0	0.14	-	1.00E+00
Small gtpase	135	0	0.52	-	1.00E+00
Serine/threonine protein kinase receptor	26	0	0.1	-	1.00E+00
Ribosomal protein	210	0	0.81	-	1.00E+00
DNA binding protein	861	0	3.32	-	1.00E+00
Ribonucleoprotein	123	0	0.47	-	1.00E+00
Nuclear hormone receptor	48	0	0.18	-	1.00E+00
Reverse transcriptase	1	0	0	-	1.00E+00
Non-receptor tyrosine protein kinase	96	0	0.37	-	1.00E+00
CREB transcription factor	18	0	0.07	-	1.00E+00
Non-receptor serine/threonine protein kinase	292	0	1.12	-	1.00E+00
Non-motor microtubule binding protein	64	0	0.25	-	1.00E+00
Non-motor actin binding protein	196	3	0.76	+	1.00E+00
ATP-binding cassette (ABC) transporter	46	0	0.18	-	1.00E+00
ATP synthase	45	0	0.17	-	1.00E+00
Neurotrophic factor	9	0	0.03	-	1.00E+00
Neuropeptide	25	0	0.1	-	1.00E+00
Myelin protein	21	0	0.08	-	1.00E+00
Mutase	12	0	0.05	-	1.00E+00

Table S10B. Details of the pathways and network associated with the differentially abundant serum proteins identified in a longitudinal cohort of vivax malaria patients defined by IPA analysis

**Pathways obtained from IPA functional analysis in longitudinal study**				
**S. No.**	**Ingenuity canonical pathway**	**-log (p-value)**	**Ratio**	**Molecules**
1	Acute phase response signalling	59.159	0.26	47
2	LXR/RXR Activation	53.53	0.288	40
3	Complement system	33.43	0.571	20
4	Coagulation system	22.059	0.395	15
5	Atherosclerosis signalling	19.841	0.144	20
6	IL-12 Signalling and production in macrophages	17.623	0.121	19
7	Clathrin-mediated endocytosis signalling	16.162	0.101	20
8	Production of Nitric Oxide and reactive oxygen species in macrophages	15.219	0.09	19
9	Intrinsic prothrombin activation pathway	12.048	0.243	9
10	Extrinsic prothrombin activation pathway	10.594	0.318	7
11	Systemic lupus erythematosus signalling	7.363	0.051	13
12	Hematopoiesis from pluripotent stem cells	5.406	0.095	6
13	Primary Immunodeficiency signalling	5.356	0.094	6
14	FXR/RXR Activation	5.133	0.064	7
15	Hepatic fibrosis/ Hepatic stellate cell activation	3.791	0.045	7
16	Actin cytoskeleton signalling	2.679	0.029	7
17	B Cell develpoment	2.633	0.083	3
18	LPS/IL-1 mediated inhibition of RXR function	2.601	0.029	7
19	IL-6 Signalling	2.549	0.04	5
20	Neuroprotective role of THOP1 in Alzheimer,s disease	2.354	0.055	3
21	Allograft rejection signalling	2.297	0.041	4
22	Autoimmune thyroid disease signalling	2.157	0.048	3
23	Communication between Innate and Adaptive immune cells	2.156	0.036	4
24	Role of macrophages, fibroblast and endothelial cells in Rheumatoid arthritis	1.858	0.02	7
25	Granulocyte adhesion and diapedesis	1.821	0.027	5
26	Dendritic cell maturation	1.782	0.024	5
27	Agranulocyte adhesion and diapedesis	1.717	0.026	5
28	Growth hormone signalling	1.705	0.038	3
29	Biotin-carboxyl carrier protein assembly	1.484	0.067	1
30	TR/RXR Activation	1.471	0.028	3

31	Role of pattern recognition receptors in recognition of bacteria and viruses	1.339	0.028	3
32	Superoxide radicals degradation	1.312	0.125	1

Table S11A. ELISA-based measurement of serum concentrations of a few selected differentially abundant proteins in low and moderately-high parasitemic vivax malaria patients and healthy control subjects

Protein Name		Median	25% Percentile	75% Percentile
SAA	HC	4.16	8.93	12.23
	LPVM	28.34	82.63	224.90
	MPVM	123.89	134.80	556.90
HP	HC	0.90	0.62	1.34
	LPVM	0.32	0.12	0.78
	MPVM	0.23	0.11	0.38
HPX	HC	1.00	0.82	1.16
	LPVM	1.09	0.91	1.23
	MPVM	1.60	0.97	2.12
Apo A1	HC	1.18	1.03	1.34
	LPVM	0.78	0.18	1.13
	MPVM	0.31	0.18	0.67
Apo E	HC	52.06	48.05	64.52
	LPVM	133.29	87.03	167.30
	MPVM	157.30	109.45	184.35

Table S11B. Summary of Kruskal–Wallis test (multiple comparison test) results performed on serum concentrations of differentially abundant proteins (ELISA-based measurement) in LPVM, MPVM and HC study cohorts

**Protein**	**One-way ANOVA**			**Multiple comparison test (Adjusted *P* Value)**		
	***P* value**	**Number of groups**	**Kruskal-Wallis statistic**	**MPVM *vs.* LP VM**	**HC *vs.* LP VM**	**HC *vs.* MPVM**
SAA	< 0.0001	3	75.25	0.077	< 0.0001	< 0.0001
HP	< 0.0001	3	40.33	0.5243	0.0002	< 0.0001
HPX	< 0.0001	3	24.08	0.0134	0.5779	< 0.0001
Apo A1	< 0.0001	3	42.73	0.3114	0.0003	< 0.0001
Apo E	< 0.0001	3	53.45	0.6833	< 0.0001	< 0.0001

Table S11C. Probability values (Mann-Whitney test) showing significance of mean differences for differentially abundant proteins in LPVM, MPVM and HC measured by ELISA

Protein Name	HC/LPVM	HC/MPVM	LPVM/MPVM
SAA	7.79E-15	4.46E-22	2.47E-04
HP	2.29E-05	3.35E-17	0.10520477
HPX	0.0900134	2.181742E-6	9.28E-04
ApoE	1.69E-08	1.84E-14	0.11605768
Apo A1	3.95E-05	2.33430E-12	0.087508425

Table S12. Statistical summary of ROC curve analysis for evaluating performance of different serum proteins for prediction of low and moderately-high parasitemic vivax malaria patients

Protein Name	AUC (LPVM/HC)	AUC (MPVM/HC)	AUC (LPVM/MPVM)
SAA(mg/L)	0.93 (95% CI: 0.87 to 0.9909)	0.97 (95% CI: 0.9336 to 1.00)	0.69 (95% CI: 0.5546 to 0.8211)
Apo E (mg/L)	0.89 (95% CI: 0.8051 to 0.9841)	0.94 (95% CI: 0.8947 to 0.9886)	0.62 (95% CI: 0.4798 to 0.7621)
HPX (g/L)	0.63 (95% CI:0.4817 to 0.7769)	0.80 (95% CI: 0.6903 to 0.9052)	0.75 (95% CI: 0.6248 to 0.8713)
Apo A1(g/L)	0.80 (95% CI: 0.6846 to 0.9176)	0.91 (95% CI: 0.8511 to 0.9777)	0.63 (95% CI: 0.4805 to 0.7815)
HP (g/L)	0.81 (95% CI:0.6934 to 0.9251)	0.92 (95% CI: 0.8631 to 0.9799)	0.62 (95% CI: 0.4739 to 0.7747)

Table S13A. ELISA-based measurement of serum concentrations of a few selected differentially abundant proteins in a longitudinal cohort of vivax malaria patients

Longitudinal				
**Protein Name**		**Median**	**25% Percentile**	**75% Percentile**
HPX	HC	0.73	0.875	1.7325
	FEB	0.84	1.3975	2.0825
	DEF	0.98	1.385	1.9325
	CON	0.91	1.285	1.7775
Apo A1	HC	1.31	0.9875	1.5
	FEB	0.425	0.275	0.6375
	DEF	0.575	0.49	0.6775
	CON	0.95	0.855	1.0925
Apo E	HC	79.77	60.765	96.0675
	FEB	195.7	165.392	230.825
	DEF	191.44	157.7	221.063
	CON	93.96	79.6075	112.628
RBP	HC	35.5	28.3625	53.2775
	FEB	22.425	17.65	32.3475
	DEF	30.115	19.0975	43.56
	CON	31.28	20.4175	44.1225
SAA	HC	2.42	1.0175	3.7825
	FEB	16.85	6.82	28.8
	DEF	12.61	4.53	22.9475
	CON	5.645	2.105	9.0425
HP	HC	0.97	0.7625	1.33
	FEB	0.29	0.1125	0.53
	DEF	0.485	0.2825	0.715
	CON	0.705	0.5575	1.0925
CP	HC	0.295	0.22	0.3675
	FEB	0.515	0.2925	0.7525
	DEF	0.365	0.225	0.56
	CON	0.305	0.2125	0.405
PLS	HC	2.02	1.705	2.41
	FEB	2.28	1.78	3.4325
	DEF	2.5	1.8375	3.46
	CON	2.2	1.625	3.44

Table S13B. Summary of Kruskal–Wallis test (multiple comparison test) results performed on serum concentrations of differentially abundant proteins (ELISA-based measurement) in HC and a longitudinal cohort of vivax malaria patients

**Protein Name**	**One-way ANOVA**			**Multiple comparison test (Adjusted *P* Value)**		
	***P* value**	**Number of groups**	**Kruskal-Wallis statistic**	**FEB *vs.* HC**	**DEF *vs.* HC**	**CON *vs.* HC**
HPX	0.1503	4	5.313	0.0965	0.2211	0.7414
Apo A1	0.0001	4	20.8	0.0001	0.0053	0.6626
Apo E	< 0.0001	4	26.69	< 0.0001	0.0004	> 0.9999
RBP	0.1399	4	5.479	0.0653	0.5056	> 0.9999
SAA	0.0012	4	15.93	0.001	0.0036	0.1907
HP	0.0076	4	11.95	0.0037	0.0739	0.9184
CP	0.1071	4	6.095	0.0812	0.7059	> 0.9999
PLS	0.7509	4	1.209	> 0.9999	0.9515	> 0.9999

Table S13C. Probability values (Mann-Whitney test) showing significance of mean differences for differentially abundant proteins in a longitudinal cohort of vivax malaria patients measured by ELISA

Protein Name	HC *vs.* FEB	HC *vs.* DEF	HC *vs.* CON
HPX	0.049731645	0.07525601	0.21006083
Apo A1	3.25E-04	4.44E-04	0.07525601
Apo E	2.1650176E-5	4.33E-05	0.16549395
RBP	0.018543376	0.21756262	0.39304814
SAA	4.87E-04	7.25E-04	0.03016952
HP	0.005196042	0.02323064	0.16549395
CP	0.03737903	0.23905042	0.78134406
PLS	0.3939394	0.30952382	0.6991342

Table S14. Statistical summary of ROC curve analysis for evaluating performance of different serum proteins for prediction of different stages of disease progression (FEB, DEF and CON stages)

Protein Name	AUC (FEB/HC)	AUC (DEF/HC)	AUC (CON/HC)
SAA(mg/L)	0.93 (95% CI: 0.87 to 0.1092)	0.92 (95% CI: 0.7920 to 1.048)	0.78 (95% CI: 0.5779 to 0.9921)
Apo E (mg/L)	0.99 (95% CI: 0.9583 to 1.022)	0.98 (95% CI: 0.9295 to 1.031)	0.69 (95% CI:0.4521 to 0.9279)
HPX (g/L)	0.75 (95% CI:0.5380 to 0.9720)	0.74 (95% CI: 0.5136 to 0.9664)	0.67 (95% CI: 0.4227 to 0.9173)
Apo A1(g/L)	0.94 (95% CI: 0.8397 to 1.040)	0.93 (95% CI: 0.8162 to 1.044)	0.74 (95% CI: 0.4988 to 0.9812)
HP (g/L)	0.86 (95% CI: 0.6894 to 1.031)	0.80 (95% CI: 0.5881 to 1.012)	0.69 (95% CI: 0.4478 to 0.9322)
RBP (mg/L)	0.81 (95% CI: 0.6213 to 0.9987)	0.67 (95% CI: 0.4298 to 0.9102)	0.62 (95% CI: 0.3654 to 0.8746)
CP (mg/L)	0.77 (95% CI: 0.5631 to 0.9869)	0.66 (95% CI: 0.4130 to 0.9070)	0.54 (95% CI: 0.2791 to 0.8009)
HPX (mg/L)	0.66 (95% CI: 0.3384 to 0.9949)	0.69 (95% CI: 0.3691 to 1.020)	0.58 (95% CI: 0.2336 to 0.9331)
